# Etiology of hospital mortality in children living in low- and middle-income countries: a systematic review and meta-analysis

**DOI:** 10.3389/fped.2024.1397232

**Published:** 2024-06-07

**Authors:** Teresa B. Kortz, Rishi P. Mediratta, Audrey M. Smith, Katie R. Nielsen, Asya Agulnik, Stephanie Gordon Rivera, Hailey Reeves, Nicole F. O’Brien, Jan Hau Lee, Qalab Abbas, Jonah E. Attebery, Tigist Bacha, Emaan G. Bhutta, Carter J. Biewen, Jhon Camacho-Cruz, Alvaro Coronado Muñoz, Mary L. deAlmeida, Larko Domeryo Owusu, Yudy Fonseca, Shubhada Hooli, Hunter Wynkoop, Mara Leimanis-Laurens, Deogratius Nicholaus Mally, Amanda M. McCarthy, Andrew Mutekanga, Carol Pineda, Kenneth E. Remy, Sara C. Sanders, Erica Tabor, Adriana Teixeira Rodrigues, Justin Qi Yuee Wang, Niranjan Kissoon, Yemisi Takwoingi, Matthew O. Wiens, Adnan Bhutta

**Affiliations:** ^1^ Department of Pediatrics, University of California, San Francisco, San Francisco, CA, United States; ^2^ Institute for Global Health Sciences, University of California, San Francisco, San Francisco, CA, United States; ^3^ Department of Pediatrics, Stanford University School of Medicine, Stanford, CA, United States; ^4^ Department of Medicine, Miller School of Medicine, Miami, FL, United States; ^5^ Department of Pediatrics and Department of Global Health, University of Washington, Seattle, WA, United States; ^6^ Department of Global Pediatric Medicine, St. Jude Children’s Research Hospital, Memphis, TN, United States; ^7^ Faculty of Medicine & Dentistry, University of Alberta, Edmonton, AB, Canada; ^8^ Department of Pediatrics, Ohio State University/Nationwide Children’s Hospital, Columbus, OH, United States; ^9^ Children's Intensive Care Unit, Department of Paediatric Subspecialties, KK Women's and Children's Hospital, Singapore, Singapore; ^10^ Paediatrics Academic Clinical Programme, Duke-NUS Medical School, Singapore, Singapore; ^11^ Department of Pediatrics and Child Health, Section of Pediatric Critical Care Medicine, Aga Khan University, Karachi, Pakistan; ^12^ Department of Pediatrics, University of Colorado, Aurora, CO, United States; ^13^ Barrow Global Health, Barrow Neurological Institute, Phoenix, AZ, United States; ^14^ Department of Pediatric and Child Health, Saint Paul Hospital Medical College, Addis Ababa, Ethiopia; ^15^ Mailman School of Public Health, Columbia University, New York, NY, United States; ^16^ Department of Pediatrics, Universidad Nacional de Colombia, Fundación Universitaria de Ciencias de la Salud (FUCS), Sociedad de Cirugía de Bogota-Hospital San José, Fundación Universitaria Sanitas, Clínica Reina Sofia Pediátrica y Mujer Colsanitas, Red Colaborativa Pediátrica de Latinoamérica (LARed Network), Bogotá D.C., Colombia; ^17^ Pediatric Critical Care Division, Department of Pediatrics, Children’s Hospital at Montefiore, New York, NY, United States; ^18^ Department of Pediatrics, Emory University, Atlanta, GA, United States; ^19^ Pediatric Emergency Unit, Child Health Directorate, Komfo Anokye Teaching Hospital, Kumasi, Ghana; ^20^ Department of Pediatrics, University of Maryland Medical Center, Baltimore, MD, United States; ^21^ Department of Pediatrics, Baylor College of Medicine, Houston, TX, United States; ^22^ Department of Pediatrics and Human Development, Michigan State University, East Lansing and Helen DeVos Children’s Hospital, Grand Rapids, MI, United States; ^23^ Pediatric Intensive Care Unit, Pediatrics and Child Health, Muhimbili University of Health and Allied Sciences (MUHAS), Dar es Salaam, Tanzania; ^24^ Department of Pediatrics, University of Texas M.D. Anderson Cancer Center, Houston, TX, United States; ^25^ Department of Medicine, Mbarara University of Science and Technology, Mbarara, Uganda; ^26^ Department of Pediatrics, Baystate Medical Center, University of Massachusetts Chan Medical School, Springfield, MA, United States; ^27^ Department of Pediatrics, Rainbow Babies and Children’s Hospital, and Department of Internal Medicine, University Hospitals of Cleveland, Cleveland, OH, United States; ^28^ Department of Pediatrics, Connecticut Children’s and University of Connecticut, Hartford, CT, United States; ^29^ Department of Biology, Pennsylvania State University, University Park, PA, United States; ^30^ Department of Pediatrics, Federal University of Minas Gerais, Belo Horizonte, Brazil; ^31^ Paediatric Intensive Care Unit, Royal Brompton Hospital, London, United Kingdom; ^32^ Department of Pediatrics and Emergency Medicine, University of British Columbia, Vancouver, BC, Canada; ^33^ Institute of Applied Health Research, University of Birmingham, Edgbaston and NIHR Birmingham Biomedical Research Centre, University Hospitals Birmingham NHS Foundation Trust and University of Birmingham, Birmingham, United Kingdom; ^34^ Department of Anesthesiology, Pharmacology and Therapeutics, University of British Columbia, Vancouver, BC, Canada; ^35^ Walimu, Kampala, Uganda; ^36^ Department of Pediatrics, Indiana University School of Medicine and Riley Children’s Health, Indianapolis, IN, United States

**Keywords:** global health, resource-limited settings, low- and middle-income countries, hospital death, hospital admission, acute illness, critical illness

## Abstract

In 2019, 80% of the 7.4 million global child deaths occurred in low- and middle-income countries (LMICs). Global and regional estimates of cause of hospital death and admission in LMIC children are needed to guide global and local priority setting and resource allocation but are currently lacking. The study objective was to estimate global and regional prevalence for common causes of pediatric hospital mortality and admission in LMICs. We performed a systematic review and meta-analysis to identify LMIC observational studies published January 1, 2005-February 26, 2021. Eligible studies included: a general pediatric admission population, a cause of admission or death, and total admissions. We excluded studies with data before 2,000 or without a full text. Two authors independently screened and extracted data. We performed methodological assessment using domains adapted from the Quality in Prognosis Studies tool. Data were pooled using random-effects models where possible. We reported prevalence as a proportion of cause of death or admission per 1,000 admissions with 95% confidence intervals (95% CI). Our search identified 29,637 texts. After duplicate removal and screening, we analyzed 253 studies representing 21.8 million pediatric hospitalizations in 59 LMICs. All-cause pediatric hospital mortality was 4.1% [95% CI 3.4%–4.7%]. The most common causes of mortality (deaths/1,000 admissions) were infectious [12 (95% CI 9–14)]; respiratory [9 (95% CI 5–13)]; and gastrointestinal [9 (95% CI 6–11)]. Common causes of admission (cases/1,000 admissions) were respiratory [255 (95% CI 231–280)]; infectious [214 (95% CI 193–234)]; and gastrointestinal [166 (95% CI 143–190)]. We observed regional variation in estimates. Pediatric hospital mortality remains high in LMICs. Global child health efforts must include measures to reduce hospital mortality including basic emergency and critical care services tailored to the local disease burden. Resources are urgently needed to promote equity in child health research, support researchers, and collect high-quality data in LMICs to further guide priority setting and resource allocation.

## 1 Introduction

As of 2019, 73 countries had not achieved the United Nations Sustainable Development Goal (SDG) 3.2 (1) to “end preventable” child mortality (2). In that same year, 7.4 million infants, children, and adolescents died globally from primarily treatable causes (3). More than 80% of these deaths occurred in low- and middle-income countries (LMICs), representing a devastating global inequity (4).

Pediatric hospital mortality is also higher in LMICs compared to high-income countries (5–7). Most hospital deaths could be avoided with reliable, timely high-quality emergency and critical care services (8–11), which are limited due to underfinanced health systems and insufficient equipment, trained personnel, and medications (12). Recognizing this, the World Health Organization (WHO) recommended strengthening emergency and critical care globally (13, 14). A better understanding of reasons children are admitted to and die in LMIC hospitals is necessary to set a prioritized agenda and advocate for resources to target the greatest drivers of morbidity and mortality in hospitalized children.

Estimates of cause specific pediatric LMIC hospital mortality and admission by region and globally are unknown. Global child mortality data from the Global Burden of Disease (GBD) studies, WHO, and United Nations Children's Fund (UNICEF) include rates and causes of death (4, 15–17); however, these population-level estimates do not provide facility-level data (18). Additionally, these estimates may not be accurate due to imputation methods (19). Many studies have reported the epidemiology of acute pediatric illness in a single hospital or country, but fail to provide regional or global estimates (20–22). To address these gaps, we conducted a systematic review and meta-analysis to determine common causes of pediatric hospital mortality and admission in LMICs.

## 2 Materials and methods

### 2.1 Data sources and search strategy

We followed published guidelines for systematic reviews of observational studies (CoCoPop framework), PRISMA and GATHER reporting standards (Supplementary Tables S1, S2) (23–25). The study was organized by the Pediatric Acute Lung Injury and Sepsis Investigator (PALISI) Global Health subgroup, reviewed by the PALISI Scientific Committee, and registered with PROSPERO (#230228). The multinational Working Group (WG) was comprised of subject matter and methodology experts. We identified eligible studies by searching MEDLINE, EMBASE, CINAHL, and LILACS using MeSH terms and keywords (Supplementary Table S3). Searches were performed by an academic librarian (SG) on November 6, 2019, with a gap analysis on March 1, 2021 (Supplementary Tables S4, S5). The protocol was published (26), and amendments are detailed in the (Supplementary Appendix S1).

### 2.2 Study selection

#### 2.2.1 Condition

The primary condition was the reported, principal cause of pediatric hospital mortality by organ system. We selected an organ system-based categorization for causes of death/admission to inform general resource requirements (Supplementary Table S6). For example, the “Respiratory System” includes common conditions (pneumonia, bronchiolitis, asthma) that require respiratory support (oxygen, mechanical ventilation). Multi-organ infections (malaria) were categorized as “Non-Organ Specific Infectious Diseases”. When more than one cause was listed (malaria with anemia), members of the WG determined the principal cause and attributed cases to only the principal cause.

#### 2.2.2 Context

Studies were eligible for inclusion if published between January 1, 2005 and February 26, 2021, and data were collected after 2000. We chose these criteria to reflect recent trends in pediatric hospitalization and mortality. We determined LMIC status using the GBD 2017 Socio-Demographic Index (SDI), a composite indicator of a country's development status that correlates with health outcomes (27). We included countries within the low-, lower-middle, and middle-SDI quintiles in the search terms. Studies that presented aggregated data representing multiple countries were included if country-specific data could be extracted. We excluded publications not representative of the LMIC setting (medical mission, foreign military hospital).

#### 2.2.3 Population

We included children admitted to a hospital (non-birth admission) in an LMIC aged 0 days-18 years. To focus on a general pediatric admission population instead of a neonatal population, we excluded studies conducted in newborn nurseries or neonatal intensive care units, any study where >50% of the sample were neonates (3, 15), and neonatal specific conditions (neonatal tetanus, birth asphyxia). Conditions not specific to neonates (tetanus, pneumonia) were categorized as described above.

Included studies reported total number of children admitted to the hospital. We excluded studies that sampled only specific patient populations and studies with exclusion criteria that resulted in a different case mix than a general pediatric hospital admission population. The WG evaluated publications from the same data source and, if the populations overlapped, we retained the most recent or relevant study. Full texts in Spanish, English, and French were included (Supplementary Figure S1).

### 2.3 Data extraction

We used Covidence (Veritas Health Innovation, Melbourne, Australia) for screening, text upload, and conflict resolution (28). Duplicates were removed, titles/abstracts and full texts were independently screened by two WG members, and conflicts were resolved by a third member. Two WG members extracted data using a case report form (Supplementary Table S7) in REDCap (29), and conflicts were adjudicated by a third member. We made no assumptions about missing or ambiguous data. We extracted data from studies reporting multiple sites as separate records if participant-level data were available for each site; studies with data aggregated across sites were extracted as one record. WG members independently assessed the risk of bias based on relevant domains adapted from the Quality in Prognosis Studies (QUIPS) tool (30): (1) study participation (generalizability to underlying population); (2) study attrition; and (3) factor measurement (cause of admission/death) (Supplementary Table S8). We resolved risk of bias assessment conflicts by consensus.

### 2.4 Data analysis

The primary outcome was cause-specific proportions for hospital mortality, reported per 1,000 pediatric hospital admissions. Secondary outcomes included case fatality rates (CFRs), calculated as the number of deaths per 1,000 pediatric hospital admissions with a given system-based illness, and cause-specific proportions for hospital admission, reported per 1,000 pediatric hospital admissions. Included studies provided raw data for the denominator and numerator to estimate proportions and 95% confidence intervals (CI). We summarized data according to study- and outcome-level characteristics. To generate a global estimate, data from more than one GBD region were required, while for regional estimates, more than one study per system was required, else data were labeled as “Not reported.” We excluded “other” diagnoses, which were a heterogeneous group of conditions that differed between studies and precluded comparisons across regions.

We anticipated heterogeneity and performed meta-analyses of causes of death, CFRs, and causes of admission using random-effects models with the Freeman-Tukey double arcsine transformation and fixed-effect models when data were limited (31). We assessed statistical heterogeneity using the variance estimates from the random-effects models. We did not use the I^2^ statistic because the mean-variance relationship of proportions can lead to misleadingly high values (32). We performed sensitivity analyses to confirm that the transformation method did not affect the main results.

We performed subgroup analyses by GBD super-region by using each covariate for stratification in the relevant meta-analysis. Additional subgroup analyses were not possible due to poor reporting of potential sources of heterogeneity. We reported summary estimates overall and according to GBD super-regions: Central Europe, Eastern Europe, and Central Asia (CE); Latin America and Caribbean (LA); North Africa and Middle East (NA); South Asia (SA); Southeast Asia, East Asia, and Oceania (SEA); and Sub-Saharan Africa (SSA) (33). A *p*-value < 0.05 was considered statistically significant. All analyses were performed using the STATA (version 17) metan command.

## 3 Results

### 3.1 Characteristics of included studies

**Figure 1:**
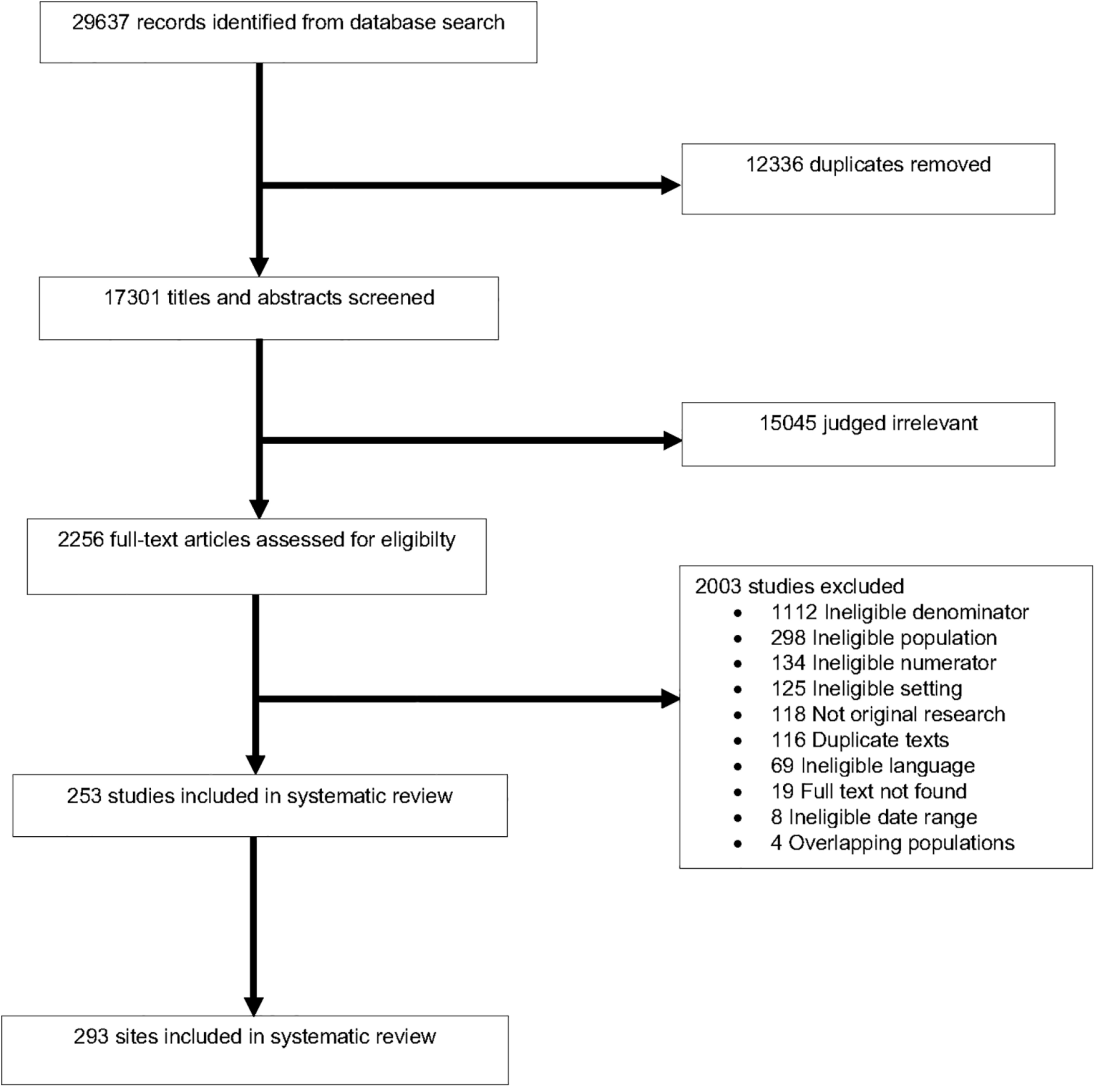
Study selection process. Preferred Reporting Items for Systematic Review and Meta-Analysis Protocols (PRISMA) flowchart for title and abstract screening and text selection from the final search (conducted March 1, 2021).

We identified 29,637 texts, removed 12,336 duplicates, screened 17,301 abstracts, and assessed 2,256 full texts for inclusion (Figure 1, Supplementary Appendix S2). Of the 253 publications included, the majority were cohort studies published after 2010. The highest number of studies came from Nigeria (*N* = 45, 15%), followed by Kenya (*N* = 27, 9%, Supplementary Figure S2). Included studies represented a total of 21,762,798 pediatric hospital admissions from 293 hospitals in 59 LMICs and six GBD super-regions (Supplementary Table S9). SSA had the highest number of sites of any super-region (*N* = 187, 64%**)**. Twenty-six percent (*N* = 76) of sites were urban and 15% (*N* = 44) were rural; 59% (*N* = 173) of sites did not report urban or rural status. Among all study sites, 9% (*N* = 25) were conducted in children's hospitals while 46% (*N* = 135) were not; 45% (*N* = 133) did not report this information. A pediatric or general intensive care unit was present in 12% (*N* = 35) of study hospitals, and not present or not reported in 82% (*N* = 243). We observed heterogeneity in outcome estimates between studies and regions (see estimates of τ^2^ in the Supplementary tables).

### 3.2 Risk of bias

Over 60% of included studies had a low risk of bias in the three domains evaluated (Supplementary Figure S3). A high risk of bias was found in 8% (*N* = 21) of studies for “Study Participation”; 5% (*N* = 14) of studies for “Study Attrition”; and 13% (*N* = 33) of studies for “Measurement Bias”.

### 3.3 Hospital mortality

**Figure 2:**
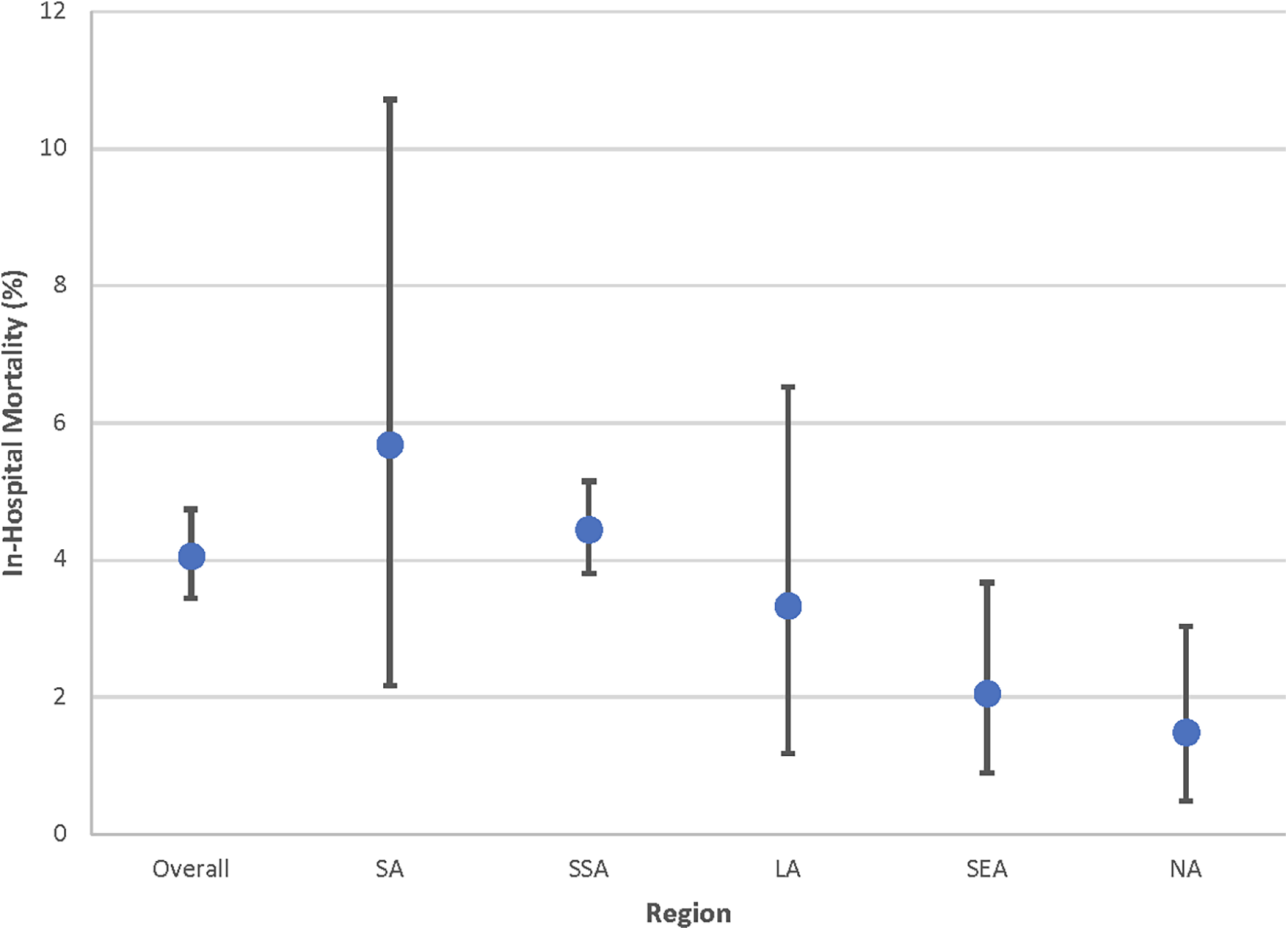
All-cause pediatric hospital mortality by Global Burden of Disease (GBD) super region. Point estimates and 95% confidence intervals shown. Estimates for Central Europe, Eastern Europe, and Central Asia (CE) are not included given limited data. LA, Latin America and Caribbean; NA, North Africa and Middle East; SA, South Asia; SEA, Southeast Asia, East Asia, and Oceania; SSA, Sub-Saharan Africa.

All-cause pediatric hospital mortality was 4.1% (95% CI 3.4%–4.7%), and summary estimates varied by region: SA had the highest all-cause hospital mortality [5.7% (95% CI 2.2–10.7%)] and NA had the lowest [1.5% (95% CI 0.5%–3.0%), Figure 2].

**Figure 3:**
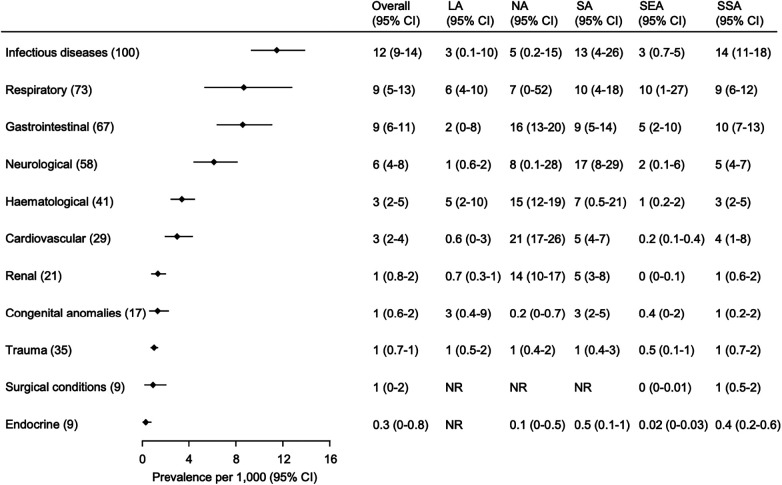
Common causes of hospital mortality in children by organ system and GBD super region. Organ systems are ordered according to the overall rate (number of children with a cause of death/1,000 children admitted) and the number in parenthesis next to each organ system represents the number of studies included in the analysis. Overall and GBD super region mortality rates are presented as pooled estimates from random-effects models with 95% confidence intervals (CI). Estimates for Central Europe, Eastern Europe, and Central Asia (CE) are not included given limited data. The hematological category includes oncological conditions. The numbers in () next to each category in the left column are the number of studies included in the overall analysis shown on the right. The categories are sorted according to the overall proportion across the super regions. The hematological category includes oncological conditions. LA, Latin America and Caribbean; NA, North Africa and Middle East; SA, South Asia; SEA, Southeast Asia, East Asia, and Oceania; SSA, Sub-Saharan Africa; CI, Confidence Interval; NR, not reported.

The most common causes of hospital death were non-organ specific infectious diseases, respiratory conditions, and gastrointestinal conditions (deaths/1,000 admission: 12 [95% CI 9–14]; 9 [95% CI 5–13], and 9 [95% CI 6–11], respectively) (Figure 3 and Supplementary Figure S4). The highest proportion of deaths due to non-organ specific infectious diseases occurred in SSA [14 deaths/1,000 admissions (95% CI 11–18)] and SA [13 deaths/1,000 admissions (95% CI 4–26)]. The highest proportion of deaths due to respiratory conditions also occurred in SA [10 deaths/1,000 admissions (95% CI 4–18)] and SSA [9 deaths/1,000 admissions (95% CI 6–12)]. The highest proportion of deaths due to gastrointestinal conditions occurred in NA [16 deaths/1,000 admissions (95% CI 13–20)].

### 3.4 Case fatality rate (CFR)

**Figure 4:**
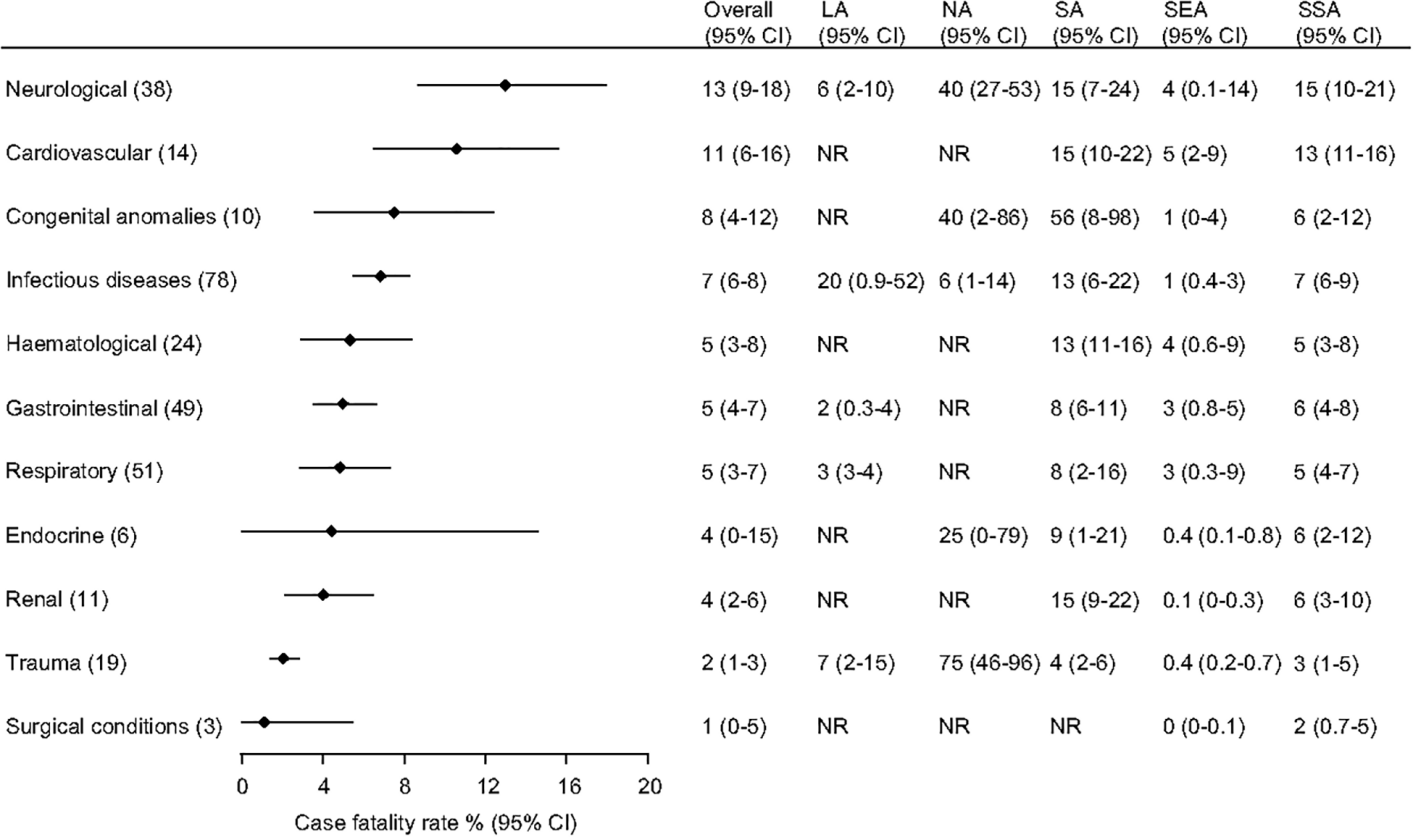
Case fatality rates in children admitted to hospital by organ system and Global Burden of Disease (GBD) super region. Organ systems are ordered according to the overall fatality (number of children with a specific cause of death/1,000 children admitted with that cause) and the number in parenthesis next to each organ system represents the number of studies included in the analysis. Overall and GBD super region case fatality rates are presented as pooled estimates from random-effects models with 95% confidence intervals (CI). Estimates for Central Europe, Eastern Europe, and Central Asia (CE) are not included given limited data. The hematological category includes oncological conditions. The numbers in () next to each category in the left column are the number of studies included in the overall analysis shown on the right. The categories are sorted according to the overall proportion across the super regions. The hematological category includes oncological conditions. LA, Latin America and Caribbean; NA, North Africa and Middle East; SA, South Asia; SEA, Southeast Asia, East Asia, and Oceania; SSA, Sub-Saharan Africa; CI, Confidence Interval; NR, not reported.

The highest overall CFRs occurred in neurological, cardiovascular, and congenital anomalies-related conditions (13% [95% CI 9%–18%], 11% [95% CI 6%–16%], and 8% [95% CI 4%–12%], respectively) (Figure 4 and Supplementary Figure S5). Neurological conditions had the highest CFR in NA [40% (95% CI 27%–53%)], while cardiovascular conditions and congenital anomalies had the highest CFR in SA (15% [95% CI 10%–22%] and 56% (95% CI 8%–98%), respectively).

### 3.5 Hospital admission

**Figure 5:**
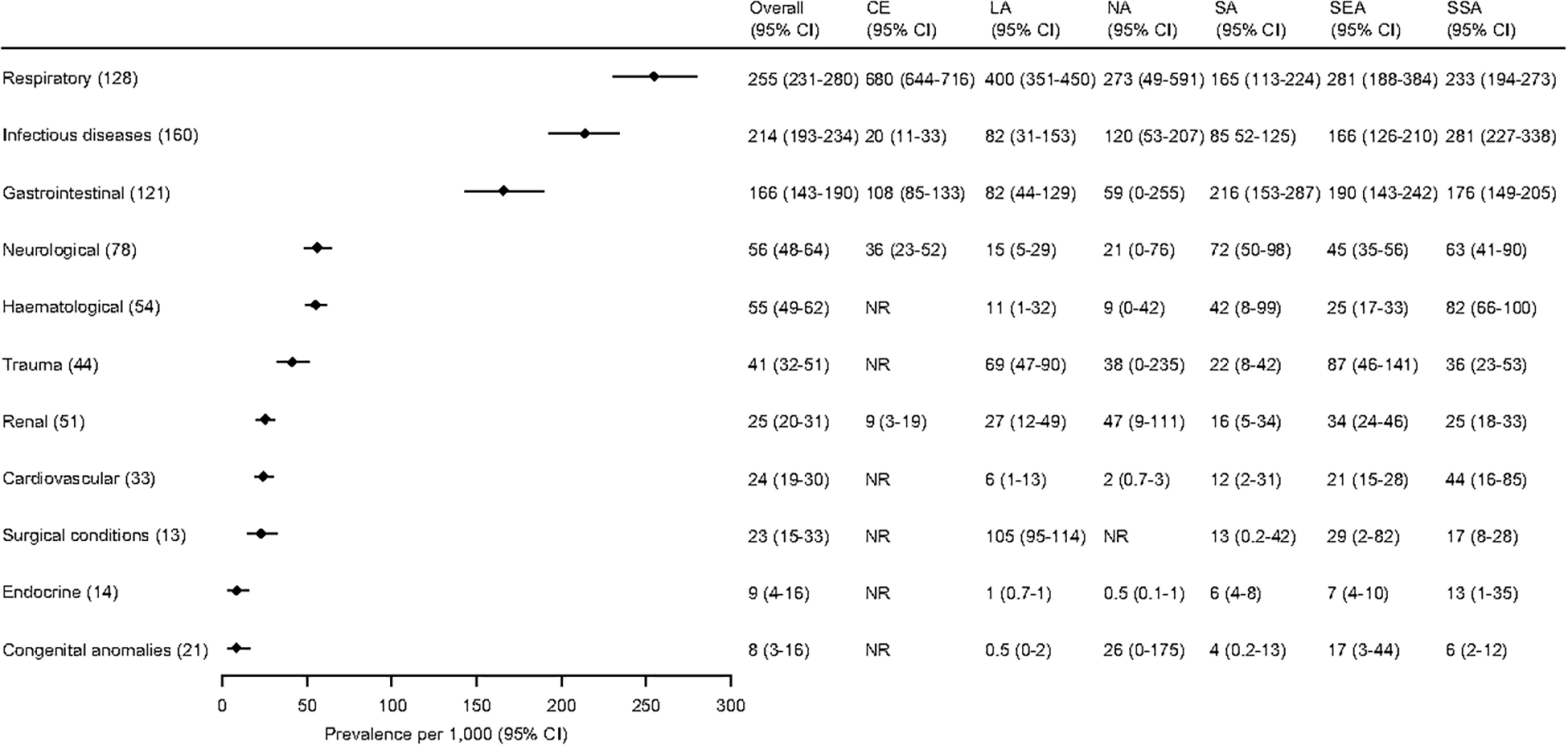
Common causes of hospital admission in children by organ system and Global Burden of Disease (GBD) super region. Organ systems are ordered according to the overall proportion of children with the cause of admission and the number in parenthesis next to each organ system represent the number of studies included in the analysis. Overall and GBD super region proportions are presented as pooled estimates from random-effects models with 95% confidence intervals (CI). Estimates for Central Europe, Eastern Europe, and Central Asia (CE) are based on a single study of 638 children. The hematological category includes oncological conditions. The numbers in () next to each category in the left column are the number of studies included in the overall analysis shown on the right. The categories are sorted according to the overall proportion across the super regions. The hematological category includes oncological conditions. CE, Central Europe, Eastern Europe, and Central Asia; LA, Latin America and Caribbean; NA, North Africa and Middle East; SA, South Asia; SEA, Southeast Asia, East Asia, and Oceania; SSA, Sub-Saharan Africa; CI, Confidence Interval; NR, not reported.

The most common causes of pediatric admissions were respiratory conditions, non-organ specific infectious diseases, and gastrointestinal conditions (cases/1,000 admissions: 255 [95% CI 231–280]; 214 [95% CI 193–234]; 166 [95% CI 143–190], respectively) (Figure 5 and Supplementary Figure S6). CE had the highest proportion of admissions due to respiratory conditions [680 cases/1,000 admissions (95% CI 644–716)], while SSA had the highest proportion due to non-organ specific infectious diseases [281 cases/1,000 admissions (95% CI 227–338)], and SA had the highest proportion due to gastrointestinal conditions [216 cases/1,000 admissions (95% CI 153–287)].

## 4 Discussion

This is the first systematic review to comprehensively identify the most common causes of pediatric hospital mortality in LMICs. All-cause pediatric hospital mortality was 4%, consistent with data from East and West Africa and India (6%–12%) but in contrast to 0.8% and 0.05% in the United States and Scotland, respectively (34–39). Similar to data published by the WHO, UNICEF, and GBD, we observed differences in cause and burden of mortality across regions, and found that the major causes of pediatric acute illness (hospital admission and death) were due to non-organ specific infectious diseases, respiratory conditions, and gastrointestinal conditions (3, 4, 15–17). The conditions with the highest CFRs, however, differed from the most common causes of hospital admission and death. Neurological and cardiovascular conditions, while less common in pediatric compared to adult populations, can cause high mortality (40, 41); it is, therefore, not surprising that these conditions account for fewer cases of hospital admission and have the highest rates of mortality when they do occur (CFR). To have the greatest impact on pediatric hospital mortality, however, LMIC hospitals should focus on management of the most frequent causes of hospital admission and death as opposed to the conditions with the highest CFRs. For the greatest regional impact on outcomes, LA and SEA hospitals should focus on respiratory conditions, while SSA hospitals should focus on non-organ specific infectious diseases. Primary prevention through vaccination; water, sanitation, and hygiene programs; improved nutrition; and mosquito control efforts are effective at reducing pediatric hospital admission directly and mortality indirectly (42–48). However, public health interventions alone are insufficient; child health efforts must also include improvements in hospital care. Simple, cost-effective strategies exist to manage common causes of pediatric admission (9, 45, 49, 50); yet, hospital mortality remains high. Most hospital deaths and long-term morbidity can be avoided with adequate emergency and critical care resources (8, 9, 11, 37). In Sierra Leone, improving emergency care processes, staffing, and resource availability decreased paediatric hospital mortality from 12%–6% (37). The basic tools necessary to deliver basic emergency and critical care and to identify and treat hospitalized patients at high risk of mortality are effective, low-cost, and low-tech (8, 37). For example, most infectious disease-related deaths are due to sepsis, and sepsis bundles have been shown to reduce mortality (51–53). Respiratory conditions can progress to respiratory failure and death; effective management includes pulse oximetry, oxygen therapy, and non-invasive and invasive ventilation (5, 54). Gastrointestinal illnesses can progress to severe dehydration and death, which can be treated with oral rehydration solution and/or intravenous fluids (IVF) (50). A recent survey of 238 hospitals in 60 countries, however, identified inconsistent availability of key resources required to care for acutely ill children including sepsis bundle resources, basic respiratory support, and dextrose-containing IVF (12). There is an urgent need for basic pediatric emergency and critical care resources in LMIC hospitals to manage common causes of admission and reduce preventable mortality (Supplementary Table S10) (8, 12).

While our analysis demonstrated global trends and regional differences in hospital mortality, admission, and CFR, country socio-economic development also impacts child outcomes (55), and is associated with resource availability and access to care (12, 56). In a global study of paediatric severe sepsis and septic shock, Tan, *et al*. found that the pooled CFR was 32% in “less developed” compared with 19% in more developed countries (7). Likewise, McAllister, *et al*., found that the CFR for children hospitalized with pneumonia was higher in low- compared to middle-income countries (6). Collectively, these findings suggest that regional differences in disease burden, resource availability, and access to high-quality hospital care can impact child mortality. More research is needed to better understand the interplay and likely synergy between burden, resources, and access; interventions that address all three are likely to have the greatest impact on child health outcomes in LMICs.

There are notable strengths of this study. The results further and independently support WHO, UNICEF, and GBD findings for the top causes of global childhood mortality and, unlike prior, large-scale global studies, represent an exclusively hospitalized pediatric population of 21.8 million (3, 4, 15–17). The focus on organ systems allows for identification of required hospital resources to manage common conditions and reduce mortality. Furthermore, this systematic review relies on health facility-level data generated in LMICs, as opposed to estimates or imputation methods, a major criticism of previously published global health metrics (19).

There are also limitations. Our analysis was restricted to available, published data and, while the search criteria had no language restrictions, we were unable to evaluate full texts not in English, Spanish or French, which may have introduced a selection bias. Although some subgroups had few studies, which could lead to an inaccurate estimate of the between-study heterogeneity, the meta-analyses across subgroups for overall summary estimates had robust sample sizes. Though the study was designed to focus on a general pediatric population, it is important to note that neonatal subjects were included. This may have resulted in an underestimate of the causes of admission and death; neonates were accounted for in the denominator, but incompletely in the numerator. While this may have influenced summary estimates, excluding all studies with neonates would similarly have resulted in studies biased against other pediatric age groups (under five years). Observed regional differences may have been influenced by available data, study selection, local health systems, and health-seeking behaviors. While most included studies had a low risk of bias, risk due to missing results (arising from reporting biases) could not be assessed. Similar to other observational studies, we were limited to the reported cause of admission/death, often a clinician's diagnosis, which could result in misclassification and highlights the need for universal research methods including standard data elements and diagnostic definitions (57). Included studies represented LMICs disproportionately; some countries (Brazil) were overrepresented, while others (Sudan) contributed no data. Underrepresented countries tended to have fewer resources and political and/or economic instability, which can contribute to higher rates of childhood illness and mortality (3). We also excluded disease-specific studies that did not report total hospital admissions and outbreak studies, which may have resulted in an underestimation for certain diseases. We attempted to recategorize “other” diagnoses; however, many could not be recategorized, which may have resulted in an underestimate of disease burden. Finally, this study was designed to capture hospital admission and death and not pre-hospital or post-discharge death, which are significant contributors to morbidity and mortality in children in LMICs (58, 59). For these reasons, this large-scale systematic review, while the first of its kind, likely underestimates the overall burden of childhood hospital mortality in LMICs.

**Figure 6:**
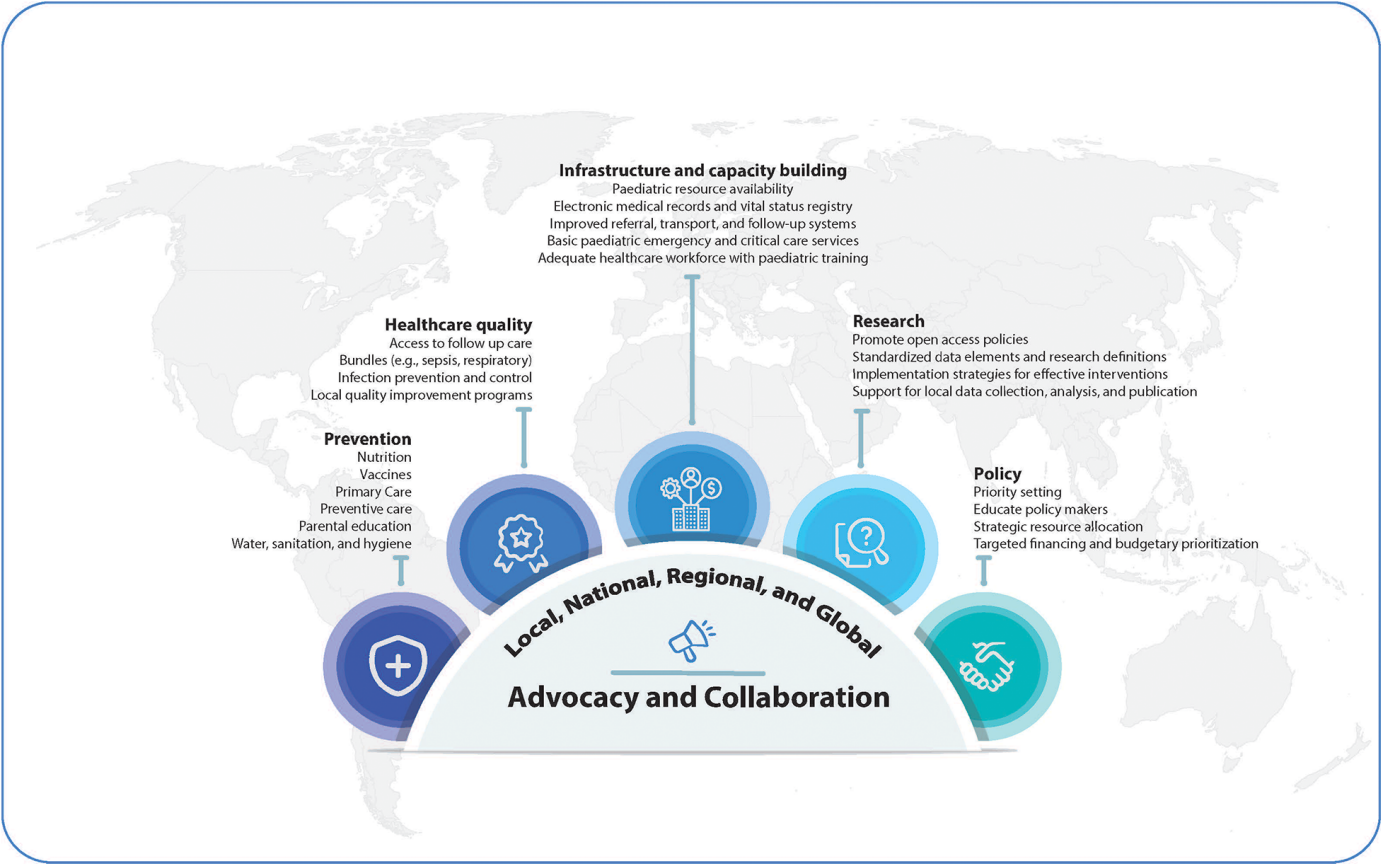
Actions to address preventable child mortality. This figure illustrates the coordinated global effort across multiple domains (e.g., healthcare quality) and levels (e.g., regional) that is required to address preventable child mortality.

Common causes of pediatric hospital mortality in LMICs could be managed with basic, cost-effective emergency and critical care services. A coordinated global effort is required to address preventable child mortality by increasing access to care, deploying targeted interventions, allocating available resources strategically, and including emergency and critical care services in the global child health agenda (Figure 6). To further reduce global child mortality and achieve the SDG target, we need public health measures, health system strengthening, and increased hospital resources tailored to the local burden of disease (Supplementary Table S10). These findings are a call to action for increased, high-quality emergency and critical care resources in LMIC hospitals to prevent avoidable pediatric hospital mortality and effectively care for children with life-threatening conditions.

## Data Availability

The datasets presented in this study can be found in online repositories. The names of the repository and DOI can be found below: https://borealisdata.ca/dataverse/Pedi_SepsisCoLab; DOI: 10.5683/SP3/2UKUKW.
