# Supplementary material for: Etiology of hospital mortality in children living in low- and middle-income countries: a systematic review and meta-analysis
Source: Front Pediatr. 2024 Jun 7;12:1397232. doi: 10.3389/fped.2024.1397232 (PMC11190367; doi:10.3389/fped.2024.1397232)
Supplement: Supplementary file 1 [file Datasheet1.pdf]

Supplementary Material

Table of content

Supplementary Table 1. The Preferred Reporting Items for Systematic Reviews and Meta-Analyses (PRISMA) checklist
Supplementary Table 2. Checklist of information that should be included in new reports of global health estimates (GATHER)
Supplementary Table 3. Detailed search strategy including full search terms by database
Supplementary Table 4. Summary of searched databases and number of texts identified by the search strategy on November 6, 2019
Supplementary Table 5. Summary of searched databases and number of texts identified by the gap search on March 1, 2021
Supplementary Table 6. Organ-System Based Categorization of Causes of Admission and Death
Supplementary Table 7. Simplified case report form
Supplementary Table 8 Risk of bias assessment domains and questions adapted from the Quality in Prognosis Studies (QUIPS) criteria
Supplementary Table 9 Included study characteristics by GBD super region
Supplementary Table 10. Recommended basic pediatric emergency and critical care resources including medications, supplies and workforce by organ system.

Supplementary Figure 1. Systematic review inclusion and exclusion criteria
Supplementary Figure 2. Map of distribution of included studies
Supplementary Figure 3. Risk of bias assessment summary
Supplementary Figure 4. Forest plots for causes of hospital mortality
Supplementary Figure 5. Forest Plots for Case Fatality Rates
Supplementary Figure 6. Forest Plots for Hospital Admission

Appendix 1. Protocol modifications
Appendix 2. List of excluded studies at full-text screening (N=2003)

Supplementary Table 1. The Preferred Reporting Items for Systematic Reviews and Meta-Analyses (PRISMA) checklist

Section and Topic	Item #	Checklist item	Location where item is reported
**TITLE**			
Title	1	Identify the report as a systematic review.	Title
**ABSTRACT**			
Abstract	2	See the PRISMA 2020 for Abstracts checklist.	Done
**INTRODUCTION**			
Rationale	3	Describe the rationale for the review in the context of existing knowledge.	Introduction
Objectives	4	Provide an explicit statement of the objective(s) or question(s) the review addresses.	Introduction
**METHODS**			
Eligibility criteria	5	Specify the inclusion and exclusion criteria for the review and how studies were grouped for the syntheses.	Methods section.<br>CoCoPop framework
Information sources	6	Specify all databases, registers, websites, organisations, reference lists and other sources searched or consulted to identify studies. Specify the date when each source was last searched or consulted.	Supplemental
Search strategy	7	Present the full search strategies for all databases, registers and websites, including any filters and limits used.	Supplemental
Selection process	8	Specify the methods used to decide whether a study met the inclusion criteria of the review, including how many reviewers screened each record and each report retrieved, whether they worked independently, and if applicable, details of automation tools used in the process.	Methods section 'Screening, extraction and quality assessment'<br>Supplemental
Data collection process	9	Specify the methods used to collect data from reports, including how many reviewers collected data from each report, whether they worked independently, any processes for obtaining or confirming data from study investigators, and if applicable, details of automation tools used in the process.	Methods section 'Screening, extraction and quality assessment' Supplemental
Data items	10a	List and define all outcomes for which data were sought. Specify whether all results that were compatible with each outcome domain in each study were sought (e.g. for all measures, time points, analyses), and if not, the methods used to decide which results to collect.	Methods sections 'Condition' and 'Outcomes'<br>Supplemental
	10b	List and define all other variables for which data were sought (e.g. participant and intervention characteristics, funding sources). Describe any assumptions made about any missing or unclear information.	Methods section 'Screening, extraction and quality assessment'<br>Supplemental (case report form)
Study risk of bias assessment	11	Specify the methods used to assess risk of bias in the included studies, including details of the tool(s) used, how many reviewers assessed each study and whether they worked independently, and if applicable, details of automation tools used in the process.	Methods section 'Screening, extraction and quality assessment'<br>Supplemental
Effect measures	12	Specify for each outcome the effect measure(s) (e.g. risk ratio, mean difference) used in the synthesis or presentation of results.	Methods section 'Data analysis'
Synthesis	13a	Describe the processes used to decide which studies were eligible for each synthesis (e.g. tabulating the study	Methods section 'Data

Section and Topic	Item #	Checklist item	Location where item is reported
methods		intervention characteristics and comparing against the planned groups for each synthesis (item #5)).	analysis'
	13b	Describe any methods required to prepare the data for presentation or synthesis, such as handling of missing summary statistics, or data conversions.	Methods section 'Data analysis'
	13c	Describe any methods used to tabulate or visually display results of individual studies and syntheses.	Methods section "data analysis"
	13d	Describe any methods used to synthesize results and provide a rationale for the choice(s). If meta-analysis was performed, describe the model(s), method(s) to identify the presence and extent of statistical heterogeneity, and software package(s) used.	Methods section "data analysis"
	13e	Describe any methods used to explore possible causes of heterogeneity among study results (e.g. subgroup analysis, meta-regression).	Methods section "data analysis"
	13f	Describe any sensitivity analyses conducted to assess robustness of the synthesized results.	Not relevant
Reporting bias assessment	14	Describe any methods used to assess risk of bias due to missing results in a synthesis (arising from reporting biases).	Not relevant
Certainty assessment	15	Describe any methods used to assess certainty (or confidence) in the body of evidence for an outcome.	Not relevant
**RESULTS**			
Study selection	16a	Describe the results of the search and selection process, from the number of records identified in the search to the number of studies included in the review, ideally using a flow diagram.	Results<br>Figure 1<br>Supplemental
	16b	Cite studies that might appear to meet the inclusion criteria, but which were excluded, and explain why they were excluded.	Supplemental
Study characteristics	17	Cite each included study and present its characteristics.	Supplemental
Risk of bias in studies	18	Present assessments of risk of bias for each included study.	Results section 'Risk of bias and heterogeneity'<br>Supplemental
Results of individual studies	19	For all outcomes, present, for each study: (a) summary statistics for each group (where appropriate) and (b) an effect estimate and its precision (e.g. confidence/credible interval), ideally using structured tables or plots.	Figures 2,3,4 and 5<br>Supplemental
Results of syntheses	20a	For each synthesis, briefly summarise the characteristics and risk of bias among contributing studies.	Supplemental
	20b	Present results of all statistical syntheses conducted. If meta-analysis was done, present for each the summary estimate and its precision (e.g. confidence/credible interval) and measures of statistical heterogeneity. If comparing groups, describe the direction of the effect.	Results<br>Supplemental
	20c	Present results of all investigations of possible causes of heterogeneity among study results.	Supplemental
	20d	Present results of all sensitivity analyses conducted to assess the robustness of the synthesized results.	Not relevant
Reporting biases	21	Present assessments of risk of bias due to missing results (arising from reporting biases) for each synthesis assessed.	Not relevant
Certainty of	22	Present assessments of certainty (or confidence) in the body of evidence for each outcome assessed.	Not relevant

Section and Topic	Item #	Checklist item	Location where item is reported
evidence			
**DISCUSSION**			
Discussion	23a	Provide a general interpretation of the results in the context of other evidence.	Discussion
	23b	Discuss any limitations of the evidence included in the review.	Discussion
	23c	Discuss any limitations of the review processes used.	Discussion
	23d	Discuss implications of the results for practice, policy, and future research.	Discussion<br>Figure 6<br>Supplemental
**OTHER INFORMATION**			
Registration and protocol	24a	Provide registration information for the review, including register name and registration number, or state that the review was not registered.	Methods section 'Search strategy and selection criteria'
	24b	Indicate where the review protocol can be accessed, or state that a protocol was not prepared.	Methods section 'Search strategy and selection criteria'
	24c	Describe and explain any amendments to information provided at registration or in the protocol.	Supplemental
Support	25	Describe sources of financial or non-financial support for the review, and the role of the funders or sponsors in the review.	Funding statement
Competing interests	26	Declare any competing interests of review authors.	Declaration of interests
Availability of data, code and other materials	27	Report which of the following are publicly available and where they can be found: template data collection forms; data extracted from included studies; data used for all analyses; analytic code; any other materials used in the review.	Data sharing statement

From: Page MJ, McKenzie JE, Bossuyt PM, Boutron I, Hoffmann TC, Mulrow CD, et al. The PRISMA 2020 statement: an updated guideline for reporting systematic reviews. BMJ 2021;372:n71. doi: 10.1136/bmj.n71
For more information, visit: http://www.prisma-statement.org/

Supplementary Table 2. Checklist of information that should be included in new reports of global health estimates

Item #	Checklist item	Reported on page #
**Objectives and funding**		
1	Define the indicator(s), populations (including age, sex, and geographic entities), and time period(s) for which estimates were made.	Methods sections 'Population' and 'Context'
2	List the funding sources for the work.	Funding statement
**Data Inputs**		
For all data inputs from multiple sources that are synthesized as part of the study:		
3	Describe how the data were identified and how the data were accessed.	Methods, Appendix
4	Specify the inclusion and exclusion criteria. Identify all ad-hoc exclusions.	Methods, Appendix
5	Provide information on all included data sources and their main characteristics. For each data source used, report reference information or contact name/institution, population represented, data collection method, year(s) of data collection, sex and age range, diagnostic criteria or measurement method, and sample size, as relevant.	Supplemental
6	Identify and describe any categories of input data that have potentially important biases (e.g., based on characteristics listed in item 5).	Supplemental
For data inputs that contribute to the analysis but were not synthesized as part of the study:		
7	Describe and give sources for any other data inputs.	Not relevant
For all data inputs:		
8	Provide all data inputs in a file format from which data can be efficiently extracted (e.g., a spreadsheet rather than a PDF), including all relevant meta-data listed in item 5. For any data inputs that cannot be shared because of ethical or legal reasons, such as third-party ownership, provide a contact name or the name of the institution that retains the right to the data.	Data sharing statement
**Data analysis**		
9	Provide a conceptual overview of the data analysis method. A diagram may be helpful.	Data analysis, Supplemental
10	Provide a detailed description of all steps of the analysis, including mathematical formulae. This description should cover, as relevant, data cleaning, data pre-processing, data adjustments and weighting of data sources, and mathematical or statistical model(s).	Data analysis, Supplemental
11	Describe how candidate models were evaluated and how the final model(s) were selected.	Data analysis
12	Provide the results of an evaluation of model performance, if done, as well as the results of any relevant sensitivity analysis.	Not relevant
13	Describe methods for calculating uncertainty of the estimates. State which sources of uncertainty were, and were not, accounted for in the uncertainty analysis.	Data analysis
14	State how analytic or statistical source code used to generate estimates can be accessed.	Data sharing statement
**Results and Discussion**		
15	Provide published estimates in a file format from which data can be efficiently extracted.	Figures, Supplemental, Data sharing
16	Report a quantitative measure of the uncertainty of the estimates (e.g. uncertainty intervals).	Figures, Tables, Supplemental
17	Interpret results in light of existing evidence. If updating a previous set of estimates, describe the reasons for changes in estimates.	Discussion
18	Discuss limitations of the estimates. Include a discussion of any modelling assumptions or data limitations that affect interpretation of the estimates.	Discussion

This checklist should be used in conjunction with the GATHER statement and Explanation and Elaboration document, found on gather-statement.org

Supplementary Table 3. Detailed search strategy including full search terms by database

Database	Search Strategy
**Ovid MEDLINE[#]**	1. *hospitalization/ or *patient admission/ or *patient readmission/<br>2. (admis* or admit* or patient discharge* or readmis* or readmit* or rehospital*).tw,kf. or hospitali*.kf.<br>3. hospital mortality/ or ((tertiary or hospital*) adj5 (mortalit* or utilis* or utiliz*)).tw,kf.<br>4. (*hospital units/ or *hemodialysis units, hospital/ or *intensive care units/ or *intensive care units, pediatric/ or *respiratory care units/ or exp *hospitals/ or *tertiary care centers/) and mortalit*.mp.<br>5. (*critical care/ or early goal-directed therapy/ or (acute care or critical care or intensive care or iicu* or picu* or tertiary care).tw,kf.) and mortalit*.mp.<br>6. (child, hospitalized/ or hospitals, pediatric/) and mortalit*.mp.<br>7. or/1-6<br>8. (Afghanistan or Albania or Algeria or Samoa or Angola or Bangladesh or Belize or Benin or Bhutan or Bolivia or Botswana or Brazil or Burkina Faso or Burundi or Cabo Verde or Cambodia or Cameroon or Central African Republic or Chad or Timor or Cape Verde or Colombia or Comoros or Congo or Costa Rica or "Cote d'Ivoire" or Cuba or Djibouti or Dominica or Dominican Republic or Ecuador or Egypt or El Salvador or Equatorial Guinea or Eritrea or Ethiopia or Fiji or Gabon or Gambia or Ghana or Grenada or Guatemala or Guinea or Guinea-Bissau or Guyana or Haiti or Honduras or India or Indonesia or Iraq or Jamaica or Jordan or Kenya or Kiribati or North Korea or "Democratic People's Republic of Korea" or Kyrgyz Republic or Kyrgyzstan or Laos or "Lao People's Democratic Republic" or Lesotho or Liberia or Madagascar or Malawi or Maldives or Seychelles or Mali or Marshall Islands or Mexico or Micronesia or Moldova or Mongolia or Morocco or Mozambique or Myanmar or Namibia or Nepal or Nicaragua or Niger or Nigeria or Pakistan or Palestine or Panama or Papua New Guinea or Paraguay or Peru or Philippines or Rwanda or Sao Tome or Principe or Senegal or Sierra Leone or Solomon Islands or Somalia or South Africa or South Sudan or Sri Lanka or St Lucia or Saint Lucia or St Vincent or Saint Vincent or Grenadines or Sudan or Suriname or Swaziland or Syrian Arab Republic or Syria or Tajikistan or Tanzania or Thailand or Timor Leste or Togo or Tonga or Trinidad or Tobago or Tunisia or Turkmenistan or Uganda or Uzbekistan or Vanuatu or Venezuela or Vietnam or West Bank or Gaza or Yemen or Zambia or Zimbabwe or Mauritania).ti,ab,kf,sh. [LMIC title, abstract, author kw, MeSH]<br>9. Developing Countries/ or (developing adj1 (nation? or countr*)).tw,kf.<br>10. (resource* adj1 (constrain* or limit* or low* or poor* or restrict*)).tw. or (resource constrain* or resource limit*).kf.<br>11. ((low* or middl*) adj1 income countr*).tw. or (low income* or middle income* or LMIC).kf.<br>12. ((developing or least* or less* or limit* or third world or underdevelop* or under develop*) adj3 (countr* or nation* or setting*)).mp.<br>13. emerging econom*.mp.<br>14. ((low or middle) adj3 (socio demographic index or SDI)).mp.<br>15. or/8-14<br>16. infant/ or child, preschool/ or child/ or (pediatric* or paediatric* or child* or baby or babies or infan* or toddler* or preschool* or preteen* or pre teen* or preadolescen* or pre adolescen* or youth* or youngster* or boy* or girl* or juvenile*).tw,kf,so,jw<br>17. (comment or editorial or letter or news).pt.<br>18. (7 and 15 and 16) not 17<br>19. (201911* or 2019 11* or 201912* or 2019 12*).dp,dt,ed,ep,ez.<br>20. limit 18 to yr="2020 -Current"<br>21. (18 and 19) or 20
**EMBASE**	#17 #16 AND [1-11-2019]/sd NOT [2-3-2021]/sd<br>#16 #6 AND #14 AND #15 NOT ('conference abstract'/it OR 'conference review'/it OR 'letter'/it OR 'editorial'/it OR 'note'/it)<br>#15 [infant]/lim OR [child]/lim OR [preschool]/lim OR [school]/lim OR pediatric*:ti,ab,kw OR paediatric*:ti,ab,kw OR child*:ti,ab,kw OR baby:ti,ab,kw OR babies:ti,ab,kw OR infan*:ti,ab,kw OR toddler*:ti,ab,kw OR preschool*:ti,ab,kw OR preteen*:ti,ab,kw OR preadolescen*:ti,ab,kw OR ((pre NEXT/1 (adolescen* OR teen*)):ti,ab,kw) OR youth*:ti,ab,kw OR youngster*:ti,ab,kw OR boy*:ti,ab,kw OR girl*:ti,ab,kw OR juvenile*:ti,ab,kw<br>#14 #7 OR #8 OR #9 OR #10 OR #11 OR #12 OR #13<br>#13 (('low middle' OR middle) NEAR/3 ('socio demographic index' OR sdi)):ti,ab,kw,de<br>#12 'emerging economy':ti,ab,kw,de OR 'emerging economies':ti,ab,kw,de<br>#11 ((developing OR underdevelop* OR underdeveloped OR least OR less OR limit* OR 'third world') NEAR/3 (countr* OR nation* OR setting*)):ti,ab,kw,de<br>#10 (((low* OR middl*) NEAR/2 countr*):ti,ab,kw) OR 'low income':ti,ab,kw OR 'middle income':ti,ab,kw OR lmic:ti,ab,kw<br>#9 (resource* NEAR/1 (constrain* OR limit* OR low* OR poor* OR restrict*)):ti,ab,kw<br>#8 'developing country'/exp OR ((developing NEAR/1 (nation* OR countr*)):ti,ab,kw)<br>#7 'afghanistan':ti,ab,de,kw OR 'albania':ti,ab,de,kw OR 'algeria':ti,ab,de,kw OR 'samoa':ti,ab,de,kw OR 'angola':ti,ab,de,kw OR 'bangladesh':ti,ab,de,kw OR 'belize':ti,ab,de,kw OR 'benin':ti,ab,de,kw OR 'bhutan':ti,ab,de,kw OR 'bolivia':ti,ab,de,kw OR 'botswana':ti,ab,de,kw OR 'brazil':ti,ab,de,kw OR 'burkina faso':ti,ab,de,kw OR 'burundi':ti,ab,de,kw OR 'cabo verde':ti,ab,de,kw OR 'cambodia':ti,ab,de,kw OR 'cameroon':ti,ab,de,kw OR 'central african republic':ti,ab,de,kw OR 'chad':ti,ab,de,kw OR 'timor':ti,ab,de,kw OR 'cape verde':ti,ab,de,kw OR 'colombia':ti,ab,de,kw OR 'comoros':ti,ab,de,kw OR 'congo':ti,ab,de,kw OR 'costa rica':ti,ab,de,kw OR 'cote d ivoire':ti,ab,de,kw OR 'cuba':ti,ab,de,kw OR 'djibouti':ti,ab,de,kw OR 'dominica':ti,ab,de,kw OR 'dominican republic':ti,ab,de,kw OR 'ecuador':ti,ab,de,kw OR 'egypt':ti,ab,de,kw

	OR 'el salvador':ti,ab,de,kw OR 'equatorial guinea':ti,ab,de,kw OR 'eritrea':ti,ab,de,kw OR 'ethiopia':ti,ab,de,kw OR 'fiji':ti,ab,de,kw OR 'gabon':ti,ab,de,kw OR 'gambia':ti,ab,de,kw OR 'ghana':ti,ab,de,kw OR 'grenada':ti,ab,de,kw OR 'guatemala':ti,ab,de,kw OR 'guinea':ti,ab,de,kw OR 'guinea bissau':ti,ab,de,kw OR 'guyana':ti,ab,de,kw OR 'haiti':ti,ab,de,kw OR 'honduras':ti,ab,de,kw OR 'india':ti,ab,de,kw OR 'indonesia':ti,ab,de,kw OR 'iraq':ti,ab,de,kw OR 'jamaica':ti,ab,de,kw OR 'jordan':ti,ab,de,kw OR 'kenya':ti,ab,de,kw OR 'kiribati':ti,ab,de,kw OR 'north korea':ti,ab,de,kw OR 'democratic people s republic of korea':ti,ab,de,kw OR 'kyrgyz republic':ti,ab,de,kw OR 'kyrgyzstan':ti,ab,de,kw OR 'laos':ti,ab,de,kw OR 'lao people s democratic republic':ti,ab,de,kw OR 'lesotho':ti,ab,de,kw OR 'liberia':ti,ab,de,kw OR 'madagascar':ti,ab,de,kw OR 'malawi':ti,ab,de,kw OR 'maldives':ti,ab,de,kw OR 'seychelles':ti,ab,de,kw OR 'mali':ti,ab,de,kw OR 'marshall islands':ti,ab,de,kw OR 'mexico':ti,ab,de,kw OR 'micronesia':ti,ab,de,kw OR 'moldova':ti,ab,de,kw OR 'mongolia':ti,ab,de,kw OR 'morocco':ti,ab,de,kw OR 'mozambique':ti,ab,de,kw OR 'myanmar':ti,ab,de,kw OR 'namibia':ti,ab,de,kw OR 'nepal':ti,ab,de,kw OR 'nicaragua':ti,ab,de,kw OR 'niger':ti,ab,de,kw OR 'nigeria':ti,ab,de,kw OR 'pakistan':ti,ab,de,kw OR 'palestine':ti,ab,de,kw OR 'panama':ti,ab,de,kw OR 'papua new guinea':ti,ab,de,kw OR 'paraguay':ti,ab,de,kw OR 'peru':ti,ab,de,kw OR 'philippines':ti,ab,de,kw OR 'rwanda':ti,ab,de,kw OR 'sao tome':ti,ab,de,kw OR 'principe':ti,ab,de,kw OR 'senegal':ti,ab,de,kw OR 'sierra leone':ti,ab,de,kw OR 'solomon islands':ti,ab,de,kw OR 'somalia':ti,ab,de,kw OR 'south africa':ti,ab,de,kw OR 'south sudan':ti,ab,de,kw OR 'sri lanka':ti,ab,de,kw OR 'st lucia':ti,ab,de,kw OR 'saint lucia':ti,ab,de,kw OR 'st vincent':ti,ab,de,kw OR 'saint vincent':ti,ab,de,kw OR 'grenadines':ti,ab,de,kw OR 'sudan':ti,ab,de,kw OR 'suriname':ti,ab,de,kw OR 'swaziland':ti,ab,de,kw OR 'syrian arab republic':ti,ab,de,kw OR 'syria':ti,ab,de,kw OR 'tajikistan':ti,ab,de,kw OR 'tanzania':ti,ab,de,kw OR 'thailand':ti,ab,de,kw OR 'timor leste':ti,ab,de,kw OR 'togo':ti,ab,de,kw OR 'tonga':ti,ab,de,kw OR 'trinidad':ti,ab,de,kw OR 'tobago':ti,ab,de,kw OR 'tunisia':ti,ab,de,kw OR 'turkmenistan':ti,ab,de,kw OR 'uganda':ti,ab,de,kw OR 'uzbekistan':ti,ab,de,kw OR 'vanuatu':ti,ab,de,kw OR 'venezuela':ti,ab,de,kw OR 'vietnam':ti,ab,de,kw OR 'west bank':ti,ab,de,kw OR 'gaza':ti,ab,de,kw OR 'yemen':ti,ab,de,kw OR 'zambia':ti,ab,de,kw OR 'zimbabwe':ti,ab,de,kw OR 'mauritania':ti,ab,de,kw<br>#6 #1 OR #2 OR #3 OR #4 OR #5<br>#5 ((((acute OR critical OR intensive) NEXT/1 care):ti,ab) OR (((hemodialysis OR respiratory) NEXT/2 (unit* OR ward*)):ti,ab) OR iicu*:ti,ab OR picu*:ti,ab) AND mortalit*<br>#4 ('intensive care unit'/mj OR 'medical intensive care unit'/mj OR 'pediatric intensive care unit'/mj OR 'hospital'/exp/mj OR 'tertiary care center'/mj OR 'intensive care'/exp/mj OR 'hospital patient'/mj OR 'hospitalized child'/mj) AND mortalit*<br>#3 'hospital mortality'/mj OR (((tertiary OR hospital*) NEAR/5 (mortalit* OR utilis* OR utiliz*)):ti,ab)<br>#2 admis*:ti,ab OR admit*:ti,ab OR ((patient NEXT/1 discharge*):ti,ab) OR readmis*:ti,ab OR readmit*:ti,ab OR rehospital*:ti,ab<br>#1 'hospitalization'/mj OR 'hospital admission'/mj OR 'hospital readmission'/mj
**CINAHL**	S21 S18 OR S20<br>S20 S7 AND S17 AND S19 AND Limiters - Published Date: 20191101-20211231<br>S19 TI (pediatric* OR paediatric* OR child* OR baby OR babies OR infan* OR toddler* OR preteen* OR (pre W1 teen*) OR preadolescen* OR (pre W1 adolescen*) OR youth* OR youngster* OR boy* OR girl* OR juvenile*) OR AB (pediatric* OR paediatric* OR child* OR baby OR babies OR infan* OR toddler* OR preschool* OR preteen* OR (pre W1 teen*) OR preadolescen* OR (pre W1 adolescen*) OR youth* OR youngster* OR boy* OR girl* OR juvenile*) OR JN (pediatric* OR paediatric* OR child* OR baby OR babies OR infan* OR toddler* OR preteen* OR (pre W1 teen*) OR preadolescen* OR (pre W1 adolescen*) OR youth* OR youngster* OR boy* OR girl* OR juvenile*)<br>S18 S7 AND S17 Limiters - Age Groups: Infant: 1-23 months, Child, Preschool: 2-5 years, Child: 6-12 years; Published Date: 20191101-20211231<br>S17 S8 OR S9 OR S10 OR S11 OR S12 OR S13 OR S14 OR S15 OR S16<br>S16 TI ((low OR middl*) N3 (socio demographic index OR sdi)) OR AB ((low OR middl*) N3 (socio demographic index OR sdi))<br>S15 TI (emerging N1 econom*) OR AB (emerging N1 econom*)<br>S14 TI ((developing OR least* OR less* OR limit* OR "third world" OR underdevelop* OR (under W1 develop*)) N3 (countr* OR nation* OR setting*)) OR AB ((developing OR least* OR less* OR limit* OR "third world" OR underdevelop* OR (under W1 develop*)) N3 (countr* OR nation* OR setting*))<br>S13 TI ((low* OR middl*) N1 income countr*) OR AB ((low* OR middl*) N1 income countr*) OR TI LMIC OR AB LMIC<br>S12 TI (resource* N1 (constrain* OR limit* OR low* OR poor* OR restrict*)) OR AB (resource* N1 (constrain* OR limit* OR low* OR poor* OR restrict*))<br>S11 (MH "Developing Countries") OR TI (developing N1 (nation* or countr*)) OR AB (developing N1 (nation* or countr*))<br>S10 MW Afghanistan or Albania or Algeria or Samoa or Angola or Bangladesh or Belize or Benin or Bhutan or Bolivia or Botswana or Brazil or “Burkina Faso” or Burundi or “Cabo Verde” or Cambodia or Cameroon or “Central African Republic” or Chad or Timor or “Cape Verde” or Colombia or Comoros or Congo or “Costa Rica” or “Cote d'Ivoire” or Cuba or Djibouti or Dominica or “Dominican Republic” or Ecuador or Egypt or “El Salvador” or “Equatorial Guinea” or Eritrea or Ethiopia or Fiji or Gabon or Gambia or Ghana or Grenada or Guatemala or Guinea or “Guinea-Bissau” or Guyana or Haiti or Honduras or India or Indonesia or Iraq or Jamaica or Jordan or Kenya or Kiribati or “North Korea” or Korea or “Kyrgyz Republic” or Kyrgyzstan or Laos or "Lao People's Democratic Republic" or Lesotho or Liberia or Madagascar or Malawi or Maldives or Seychelles or Mali or “Marshall Islands” or Mexico or Micronesia or Moldova or Mongolia or Morocco or Mozambique or Myanmar or Namibia or Nepal or Nicaragua or Niger or Nigeria or Pakistan or Palestine or Panama or “Papua New Guinea” or Paraguay or Peru or Philippines or Rwanda or “Sao Tome” or “Principe or Senegal” or “Sierra Leone” or “Solomon Islands” or Somalia or “South Africa” or “South Sudan” or “Sri Lanka” or “St Lucia” or “Saint Lucia” or “St Vincent” or “Saint

	Vincent" or Grenadines or Sudan or Suriname or Swaziland or "Syrian Arab Republic" or Syria or Tajikistan or Tanzania or Thailand or "Timor Leste" or Togo or Tonga or Trinidad or Tobago or Tunisia or Turkmenistan or Uganda or Uzbekistan or Vanuatu or Venezuela or Vietnam or "West Bank" or Gaza or Yemen or Zambia or Zimbabwe or Mauritania<br>S9 AB Afghanistan or Albania or Algeria or Samoa or Angola or Bangladesh or Belize or Benin or Bhutan or Bolivia or Botswana or Brazil or "Burkina Faso" or Burundi or "Cabo Verde" or Cambodia or Cameroon or "Central African Republic" or Chad or Timor or "Cape Verde" or Colombia or Comoros or Congo or "Costa Rica" or "Cote d'Ivoire" or Cuba or Djibouti or Dominica or "Dominican Republic" or Ecuador or Egypt or "El Salvador" or "Equatorial Guinea" or Eritrea or Ethiopia or Fiji or Gabon or Gambia or Ghana or Grenada or Guatemala or Guinea or "Guinea-Bissau" or Guyana or Haiti or Honduras or India or Indonesia or Iraq or Jamaica or Jordan or Kenya or Kiribati or "North Korea" or Korea or "Kyrgyz Republic" or Kyrgyzstan or Laos or "Lao People's Democratic Republic" or Lesotho or Liberia or Madagascar or Malawi or Maldives or Seychelles or Mali or "Marshall Islands" or Mexico or Micronesia or Moldova or Mongolia or Morocco or Mozambique or Myanmar or Namibia or Nepal or Nicaragua or Niger or Nigeria or Pakistan or Palestine or Panama or "Papua New Guinea" or Paraguay or Peru or Philippines or Rwanda or "Sao Tome" or "Principe or Senegal" or "Sierra Leone" or "Solomon Islands" or Somalia or "South Africa" or "South Sudan" or "Sri Lanka" or "St Lucia" or "Saint Lucia" or "St Vincent" or "Saint Vincent" or Grenadines or Sudan or Suriname or Swaziland or "Syrian Arab Republic" or Syria or Tajikistan or Tanzania or Thailand or "Timor Leste" or Togo or Tonga or Trinidad or Tobago or Tunisia or Turkmenistan or Uganda or Uzbekistan or Vanuatu or Venezuela or Vietnam or "West Bank" or Gaza or Yemen or Zambia or Zimbabwe or Mauritania<br>S8 TI Afghanistan or Albania or Algeria or Samoa or Angola or Bangladesh or Belize or Benin or Bhutan or Bolivia or Botswana or Brazil or "Burkina Faso" or Burundi or "Cabo Verde" or Cambodia or Cameroon or "Central African Republic" or Chad or Timor or "Cape Verde" or Colombia or Comoros or Congo or "Costa Rica" or "Cote d'Ivoire" or Cuba or Djibouti or Dominica or "Dominican Republic" or Ecuador or Egypt or "El Salvador" or "Equatorial Guinea" or Eritrea or Ethiopia or Fiji or Gabon or Gambia or Ghana or Grenada or Guatemala or Guinea or "Guinea-Bissau" or Guyana or Haiti or Honduras or India or Indonesia or Iraq or Jamaica or Jordan or Kenya or Kiribati or "North Korea" or Korea or "Kyrgyz Republic" or Kyrgyzstan or Laos or "Lao People's Democratic Republic" or Lesotho or Liberia or Madagascar or Malawi or Maldives or Seychelles or Mali or "Marshall Islands" or Mexico or Micronesia or Moldova or Mongolia or Morocco or Mozambique or Myanmar or Namibia or Nepal or Nicaragua or Niger or Nigeria or Pakistan or Palestine or Panama or "Papua New Guinea" or Paraguay or Peru or Philippines or Rwanda or "Sao Tome" or "Principe or Senegal" or "Sierra Leone" or "Solomon Islands" or Somalia or "South Africa" or "South Sudan" or "Sri Lanka" or "St Lucia" or "Saint Lucia" or "St Vincent" or "Saint Vincent" or Grenadines or Sudan or Suriname or Swaziland or "Syrian Arab Republic" or Syria or Tajikistan or Tanzania or Thailand or "Timor Leste" or Togo or Tonga or Trinidad or Tobago or Tunisia or Turkmenistan or Uganda or Uzbekistan or Vanuatu or Venezuela or Vietnam or "West Bank" or Gaza or Yemen or Zambia or Zimbabwe or Mauritania<br>S7 S1 OR S2 OR S3 OR S4 OR S5 OR S6<br>S6 ((MH "Critical Care") OR (MH "Hospitals, Pediatric") OR (MH "Child, Hospitalized")) AND TX mortalit*<br>S5 (TI (("acute care" OR "critical care" OR "intensive care" OR iicu* OR picu* OR "tertiary care") AND TX mortalit*) OR (AB (("acute care" OR "critical care" OR "intensive care" OR iicu* OR picu* OR "tertiary care") AND TX mortalit*)<br>S4 ((MH "Hospital Units") OR (MH "Pediatric Units") OR (MH "Intensive Care Units") OR (MH "Intensive Care Units, Pediatric") OR (MH "Respiratory Care Units") OR (MH "Tertiary Health Care")) AND TX mortalit<br>S3 (MH "Hospital Mortality") OR (TI ((tertiary OR hospital*) N5 (mortalit* OR utilis* OR utiliz*))) OR (AB ((tertiary OR hospital*) N5 (mortalit* OR utilis* OR utiliz*)))<br>S2 TI (admis* OR admit* OR (patient W1 discharge*) OR readmis* OR readmit* OR rehospital*) OR AB (admis* OR admit* OR (patient W1 discharge*) OR readmis* OR readmit* OR rehospital*)<br>S1 (MH "Hospitalization") OR (MH "Patient Admission") OR (MH "Readmission")
**LILACS**	VHL Advanced Search<br>#1<br>(mh:("Hospitalization" OR "Child, Hospitalized" OR "Patient Admission" OR "Patient Readmission" OR "Hospital Mortality")) AND (tw:(develop* OR desarrollo OR resource* OR "third world" OR underdeveloped OR under developed OR low income OR bajos ingresos OR middle income OR ingreso medio OR LMIC OR emerging econom* OR "socio demographic index" OR SDI OR Afghanistan OR Albania OR Algeria OR Samoa OR Angola OR Bangladesh OR Belize OR Benin OR Bhutan OR Bolivia OR Botswana OR Brazil OR "Burkina Faso" OR Burundi OR "Cabo Verde" OR Cambodia OR Cameroon OR "Central African Republic" OR Chad OR Timor OR "Cape Verde" OR Colombia OR Comoros OR Congo OR "Costa Rica" OR "Cote d'Ivoire" OR Cuba OR Djibouti OR Dominica OR "Dominican Republic" OR Ecuador OR Egypt OR "El Salvador" OR "Equatorial Guinea" OR Eritrea OR Ethiopia OR Fiji OR Gabon OR Gambia OR Ghana OR Grenada OR Guatemala OR Guinea OR "Guinea-Bissau" OR Guyana OR Haiti OR Honduras OR India OR Indonesia OR Iraq OR Jamaica OR Jordan OR Kenya OR Kiribati OR "North Korea" OR Korea OR "Kyrgyz Republic" OR Kyrgyzstan OR Laos OR "Lao People's Democratic Republic" OR Lesotho OR Liberia OR Madagascar OR Malawi OR Maldives OR Seychelles OR Mali OR "Marshall Islands" OR Mexico OR Micronesia OR Moldova OR Mongolia OR Morocco OR Mozambique OR Myanmar OR Namibia OR Nepal OR Nicaragua OR Niger OR Nigeria OR Pakistan OR Palestine OR Panama OR "Papua New Guinea" OR Paraguay OR Peru OR Philippines OR Rwanda OR "Sao Tome" OR "Principe or Senegal" OR "Sierra Leone" OR "Solomon Islands" OR Somalia OR "South Africa" OR "South Sudan" OR "Sri Lanka" OR "St Lucia" OR "Saint Lucia" OR "St Vincent" OR "Saint Vincent" OR Grenadines OR Sudan OR Suriname OR Swaziland OR "Syrian Arab Republic" OR Syria OR Tajikistan OR Tanzania OR Thailand OR "Timor Leste" OR Togo OR Tonga OR Trinidad OR Tobago OR Tunisia

	OR Turkmenistan OR Uganda OR Uzbekistan OR Vanuatu OR Venezuela OR Vietnam OR "West Bank" OR Gaza OR Yemen OR Zambia OR Zimbabwe OR Mauritania)) AND (tw:(pediatric* OR paediatric* OR child* OR baby OR babies OR infan* OR toddler* OR preschool* OR preteen* OR preadolescen* OR youth* OR youngster* OR boy* OR girl* OR juvenil* OR nina OR nino OR ninit* OR preescholar* OR chico* OR chica*))<br>Limited To: LILACS and 2019-2021<br><br>#2<br>(tw:(critical care OR cuidados críticos OR intensive care OR respiratory care OR cuidados respiratorios OR hospital* OR tertiary OR terciaria)) AND (tw:(mortali* OR admis* OR admit* OR readmis* OR readmit* OR rehospital*)) AND (tw:(develop* OR desarrollo OR resource* OR "third world" OR underdeveloped OR under developed OR low income OR bajos ingresos OR middle income OR ingreso medio OR LMIC OR emerging econom* OR "socio demographic index" OR SDI OR Afghanistan OR Albania OR Algeria OR Samoa OR Angola OR Bangladesh OR Belize OR Benin OR Bhutan OR Bolivia OR Botswana OR Brazil OR "Burkina Faso" OR Burundi OR "Cabo Verde" OR Cambodia OR Cameroon OR "Central African Republic" OR Chad OR Timor OR "Cape Verde" OR Colombia OR Comoros OR Congo OR "Costa Rica" OR "Cote d'Ivoire" OR Cuba OR Djibouti OR Dominica OR "Dominican Republic" OR Ecuador OR Egypt OR "El Salvador" OR "Equatorial Guinea" OR Eritrea OR Ethiopia OR Fiji OR Gabon OR Gambia OR Ghana OR Grenada OR Guatemala OR Guinea OR "Guinea-Bissau" OR Guyana OR Haiti OR Honduras OR India OR Indonesia OR Iraq OR Jamaica OR Jordan OR Kenya OR Kiribati OR "North Korea" OR Korea OR "Kyrgyz Republic" OR Kyrgyzstan OR Laos OR "Lao People's Democratic Republic" OR Lesotho OR Liberia OR Madagascar OR Malawi OR Maldives OR Seychelles OR Mali OR "Marshall Islands" OR Mexico OR Micronesia OR Moldova OR Mongolia OR Morocco OR Mozambique OR Myanmar OR Namibia OR Nepal OR Nicaragua OR Niger OR Nigeria OR Pakistan OR Palestine OR Panama OR "Papua New Guinea" OR Paraguay OR Peru OR Philippines OR Rwanda OR "Sao Tome" OR "Principe or Senegal" OR "Sierra Leone" OR "Solomon Islands" OR Somalia OR "South Africa" OR "South Sudan" OR "Sri Lanka" OR "St Lucia" OR "Saint Lucia" OR "St Vincent" OR "Saint Vincent" OR Grenadines OR Sudan OR Suriname OR Swaziland OR "Syrian Arab Republic" OR Syria OR Tajikistan OR Tanzania OR Thailand OR "Timor Leste" OR Togo OR Tonga OR Trinidad OR Tobago OR Tunisia OR Turkmenistan OR Uganda OR Uzbekistan OR Vanuatu OR Venezuela OR Vietnam OR "West Bank" OR Gaza OR Yemen OR Zambia OR Zimbabwe OR Mauritania)) AND (tw:(pediatric* OR paediatric* OR child* OR baby OR babies OR infan* OR toddler* OR preschool* OR preteen* OR preadolescen* OR youth* OR youngster* OR boy* OR girl* OR juvenil* OR nina OR nino OR ninit* OR preescholar* OR chico* OR chica*))

# MEDLINE strategy abbreviations:
ab = abstract
jw = journal word
kf = keyword heading word
mp = title, abstract, original title, name of substance word, subject heading word, floating sub-heading word, keyword heading word, organism supplementary concept word, protocol supplementary concept word, rare disease supplementary concept word, unique identifier, synonyms
pt = publication type
sh = MeSH subject heading
so = source
ti = title
tw = text word in abstract or title

Supplementary Table 4. Summary of searched databases and number of texts identified by the search strategy on November 6, 2019

Database	Date Searched	Results
Ovid MEDLINE(R) and Epub Ahead of Print, In-Process & Other Non Indexed Citations, Daily and Versions(R) (1946 to November 5, 2019)	11/6/2019	9564
EMBASE.com (1974 to Present, includes Medline 1966 to Present)	11/6/2019	9384
Cumulative Index to Nursing and Allied Health Literature (CINAHL, 1981 to Present)	11/6/2019	3316
Latin American and Caribbean Health Sciences Literature (LILACS, 1982 to Present)	11/6/2019	1282
	**TOTAL**	**23,546**

Supplementary Table 5. Summary of searched databases and number of texts identified by the gap search on March 1, 2021

Database	Dates Included	Date Searched	Number of Texts Identified
Ovid MEDLINE(R)<br>Epub Ahead of Print<br>In-Process and other Non-Indexed Citations<br>Daily and Versions(R)	1946 to February 26, 2021	3/1/2021	11240
EMBASE.com	1974 to Present (includes Medline 1966 to Present)	3/1/2021	11403
Cumulative Index to Nursing and Allied Health Literature (CINAHL)	1981 to Present	3/1/2021	3878
Latin American and Caribbean Health Sciences Literature (LILACS)	1982 to Present	3/1/2021	1453
**TOTAL**			**27974**

Supplementary Table 6. Organ-System Based Categorization of Causes of Admission and Death

System	High-level Diagnoses	Specific Diagnosis Terms	Explanation/Example
**Cardiovascular**			
	Congenital Heart Disease	Congenital heart disease	Includes variations on the term "congenital heart disease" and specific lesions (e.g., coarctation)
	Cardiac Failure	Heart failure	Includes acute heart failure, congestive heart failure, and anemic heart failure
	Shock (not septic)	Shock (not septic)	Includes unspecified, hypovolemic, cardiogenic and distributive shock
	Other Cardiovascular Disease		Includes less common specific cardiovascular diagnoses
		Rheumatic fever/heart disease	
		Other cardiovascular disease	Includes uncommon specific diagnoses not categorized under another high-level diagnosis, for example, endocarditis and cardiomyopathy
	Cardiovascular, NOS	Cardiovascular, NOS	Unspecified cardiovascular conditions, i.e., "heart diseases" and "cardiovascular conditions"
**Respiratory**			
	Pneumonia and Lower Respiratory Tract Infections		Pneumonia and other lower respiratory tract infections categorized together given overlapping clinical presentation and similar resource requirements for management
		Community-Acquired Pneumonia	Includes bacterial pneumonia, empyema, community-acquired pneumonia, etc.
		Pneumonia, not community-acquired	Includes aspiration pneumonia and hospital-acquired pneumonia
		Influenza	Includes unspecified influenza and H1N1

		Lower respiratory tract infection	Includes unspecified LRTI, acute LRTI, RSV LRTI, etc.
		Bronchiolitis/bronchitis	Includes bronchiolitis, RSV-related acute respiratory infection, bronchitis, etc.
	Asthma	Asthma	Includes asthmatic crisis, acute severe asthma, bronchial asthma, etc.
	Other Respiratory		
		Upper respiratory tract infection	
		Acute respiratory tract infection unspecified	Any unspecified acute respiratory infection
		Respiratory insufficiency/failure	Includes ARDS, respiratory insufficiency, and respiratory failure
		Other respiratory disease	Specific respiratory diagnoses not otherwise classified under a high-level diagnosis, for example, acute airway obstruction, COPD, pulmonary embolism, etc.
	Respiratory, NOS	Respiratory, NOS	Cardiovascular conditions that were not specified, for example, “respiratory diseases” and “other respiratory”
**Gastrointestinal**			
	Gastroenteritis and diarrhea		
		Gastroenteritis	Includes unspecified gastroenteritis, infectious gastroenteritis, food poisoning, etc.
		Diarrhea and dehydration	Includes rotavirus diarrhea, cholera, dysentery, etc.
	Liver disease, hepatitis, liver failure	Liver disease, hepatitis, liver failure	Includes jaundice, viral hepatitis, liver cirrhosis, etc.
	Malnutrition		
		Severe malnutrition	Includes severe acute malnutrition, stunting, wasting, etc.

		Protein-energy malnutrition, Kwashiorker and Marasmus	
		Rickets	
		Malnutrition NOS	Unspecified malnutrition, for example “malnutrition (mild, moderate, severe),” and “nutritional deficiencies
	Other GI	Gastritis and peptic ulcer disease	Includes gastrointestinal ulcers, gastritis, and PUD
	Gastrointestinal, NOS	Gastrointestinal, NOS	Gastrointestinal conditions that were unspecified or did not belong to another high-level diagnosis, i.e., fecal impaction, abdominal pain, “digestive diseases,” etc.
**Genitourinary/Renal**			
	Renal Failure		
		Acute renal failure or injury	Includes acute kidney injury, renal failure, community-acquired AKI, etc.
		Chronic kidney disease/failure	Includes chronic kidney disease, chronic renal failure, uremic encephalopathy/cirrhosis, etc.
	Urinary Tract Infection/Pyelonephritis	Urinary Tract Infection/Pyelonephritis	
	Glomerulonephritis, nephrotic and nephritic syndrome	Glomerulonephritis, nephrotic and nephritic syndrome	Includes unspecified glomerulonephritis, nephritis, post-strep glomerulonephritis, etc.
	Other genitourinary/renal		
		Hemolytic uremic syndrome	Includes HUS and congenital nephrotic syndrome
		Renal tubulo-interstitial diseases	Includes renal tubular acidosis, intrinsic renal disease, Bartter’s syndrome, etc.
		Obstructive/anatomical lesions	Includes urolithiasis, renal artery stenosis, obstructive uropathy, etc.
	Genitourinary/Renal, NOS	Genitourinary/Renal, NOS	Unspecified genitourinary/renal conditions, for example “diseases of the genitourinary system” and “nephropathies”
**Neurological/Psychiatric**			
	Epilepsy/Seizures		

		Epilepsy and seizure disorder	Includes epilepsy, seizures, cerebral palsy with seizure, etc.
		Febrile seizures	
	Meningitis/Encephalitis		
		Bacterial meningitis	Includes pneumococcal meningitis, haemophilus meningitis, unspecified meningitis, etc.
		Meningoencephalitis	Includes herpes encephalitis, viral meningitis, cryptococcal meningitis, etc.
		Unspecified or other CNS infection	Includes unspecified CNS infections and neurocysticercosis
	Other neurologic		
		Mental and behavioral disorders	Includes unspecified mental and behavioral conditions, schizophrenia, and drug abuse
		Cerebrovascular accident/disease	Includes stroke, multifocal vaso-occlusive crisis, and unspecified cerebrovascular conditions
		Encephalopathy and coma	Includes acute febrile encephalopathy, hepatic coma, hypoxic encephalopathy, etc.
		Acute Paralysis	Includes flaccid paralysis and Guillain Barré
	Neurologic/Psychiatric, NOS	Neurologic/Psychiatric, NOS	All neurological/psychiatric causes of admission and/or death that were not specified, for example “diseases of the nervous system” and “mental disorders”
**Infectious Diseases**			
	Malaria		
		Severe/complicated malaria	Includes cerebral malaria, severe malaria, complicated malaria
		Malaria	
	HIV/AIDS and related illnesses	HIV/AIDS and related illnesses	Includes HIV, AIDS, pneumocystis pneumonia, etc.
	Sepsis and Septic Shock		
		Sepsis	Includes typhoid septicemia, pneumococcal sepsis, bacteremia, etc.

		Severe sepsis	
		Septic shock	
	COVID-19	COVID-19	
	Tuberculosis		
		Pulmonary tuberculosis	
		Extra-pulmonary tuberculosis	Includes extrapulmonary TB, disseminated TB, and tuberculous meningitis
	Measles	Measles	
	Typhoid Fever	Typhoid Fever	
	Tetanus	Tetanus	Includes post-neonatal tetanus, wound infection/tetanus neonatorum, unspecified tetanus
	Dengue	Dengue	
	Other vaccine-preventable diseases	Other vaccine-preventable diseases	Includes rabies, pertussis, varicella, etc.
	Other infections		
		Ear, nose, and throat infections	Includes acute pharyngitis, adenoiditis, otitis media, etc.
		Fever without a source	Includes febrile illness, fever without focus, unspecified fever, etc.
		Skin and soft tissue infections	Includes abscesses, cellulitis, candida, etc.
		Hospital-acquired infections	Includes nosocomial and surgical infections
		Other uncommon infections	Includes rare infections such as leishmaniasis, scrub typhus, and leptospirosis
**Hematologic/Oncologic**			
	Malignancy		
		Hematologic malignancy	Includes acute lymphoblastic leukemia, acute myeloid leukemia, Burkitt lymphoma, etc.
		Solid tumor	Includes Wilm's tumor, hepatoma, neuroblastoma, etc.
	Anemia	Anemia	Includes severe anemia, hemolytic anemia, aplastic anemia, etc.
	Sickle cell and thalassemia	Sickle cell and thalassemia	Includes sickle cell disease, haemoglobinopathies and haemolytic anemia, thalassemia, etc.
	Non-malignant hematology	Non-malignant hematology	Includes pancytopenia, hemophilia, bleeding disorders, etc.

	Hematologic/Oncologic, NOS	Hematologic/Oncologic, NOS	Hematologic/oncologic conditions that were not further specified, for example, “malignancy” and “neoplasm”
**Trauma/Injury/Ingestion**			
	Trauma/Injury		
		Burns and electric shock	Includes burns and corrosions, electrocution, lightning victims, etc.
		Transport accidents	Includes road traffic accidents, pedestrian injuries, and motorcycle accidents
		Other trauma/injury	Trauma/injuries not belonging to an above category, for example, falls, assault, unspecified trauma
	Toxic Poisoning/Ingestion		
		Acute poisoning	Includes drug and chemical poisonings, caustic ingestion, etc.
		Kerosene ingestion	
		Alcohol poisoning	
	Other Poisoning/Ingestion		
		Bites and stings	Includes dog bites, scorpion stings, snake bites, etc.
		Foreign body ingestion/aspiration	
	Trauma/Injury/Ingestion, NOS	Trauma/Injury/Ingestion, NOS	Traumas, injuries, and ingestions that could not be further stratified, for example, “trauma, burns, poisoning” and “accidents and poisonings”
**Endocrine/Metabolic**			
	Diabetes/Diabetic Ketoacidosis (DKA) and Disorders of Blood Glucose	Diabetes/Diabetic Ketoacidosis (DKA) and Disorders of Blood Glucose	
	Endocrine/Metabolic, NOS	Endocrine/Metabolic, NOS	Endocrine/metabolic conditions that were not specified, for example, “metabolic disorders” and “other endocrine/metabolic”
**Congenital anomalies**			
	Congenital anomalies		
		Congenital Neurologic	Includes cerebral palsy, spina bifida, myelomeningocele, etc.

		Congenital Renal	Includes bladder exstrophy, undescended testis, and unspecified congenital genitourinary anomalies, etc.
		Congenital Gastrointestinal	Includes anal stenosis, congenital bowel atresia, and necrotizing enterocolitis
		Congenital Respiratory	
		Congenital anomalies, NOS	Congenital anomalies that were not further specified, for example, “congenital malformation” and “birth defects”
**Surgical Condition**			
	Surgical condition		
		Surgical condition	Includes appendicitis, intestinal obstruction, hernias, etc.
		Surgical unspecified	Includes general surgical “conditions”, “cases”, or “emergencies”
**Other**			
	Other systems		Other systems not listed above with relatively few diagnoses
		Rheumatologic and immunologic	Includes unspecified autoimmune disease, septic arthritis, rheumatoid arthritis, etc.
		Pregnancy and peripartum	Includes complications of labor and childbirth, puerperium-related edema, pregnancy, etc.
		HEENT	Includes eye disorders, dental conditions, diseases of the ear, etc.
		Skin, Musculoskeletal	Includes unspecified musculoskeletal diseases, skin pathologies, contracture, etc.
		Allergic conditions	Includes urticaria and allergic rhinitis
	Other, NOS	Other, NOS	Causes of admission/death presented as “other,” “unknown,” or otherwise uncategorizable

NOS: not otherwise specified, LRTI: lower respiratory tract infection, RSV: respiratory syncytial virus, ARDS: acute respiratory distress syndrome, COPD: chronic obstructive pulmonary disease, PUD: peptic ulcer disease, AKI: acute kidney injury, HUS: hemolytic uremic syndrome, HIV/AIDS: human immunodeficiency virus/acquired immunodeficiency syndrome, HEENT: head-ears-eyes-nose-throat

Supplementary Table 7. Simplified case report form

	Choices (if applicable)
**Study Tracking**	
Covidence ID	
Data extractor name	
Title	
First Author Last Name	
Published year	
Are there multiple sites (hospitals, countries, etc.) with site-level patient data in this study?	
Record the study site country for this entry here:	
Record the name of the hospital for this entry here:	
**Inclusion Criteria Screen**	
Enter the denominator here:	
Is the publication in English/French/Spanish?	Yes, No
Is the publication from a LMIC using SDI criteria?	Yes, No
Is the publication representative of the setting (e.g., publications from medical missions should be excluded)?	Yes, No
Is the publication original, peer-reviewed research (NOT an abstract, conference presentation, internal document, editorial, commentary, or review)?	Yes, No
Is it an observational study design (prospective or retrospective cohorts, surveillance studies, hospital database publications, cross-sectional studies, before data from before-and-after studies)?	Yes, No
Does the publication include research subjects that fit ALL of the following categories?<br>1. There must be inclusion of pediatric patients aged 28 days to 12 years (studies including larger pediatric or more narrow age ranges can be included if the age group of interest is included) and separated from adult patients. It is ok to include studies with children from < 28 days or adolescents.<br>2. Admitted to hospital in LMIC (any inpatient location EXCEPT NICU-specific studies)<br>3. Data derived from 2000-present (studies including cohort data before 2000 can be included if data presented by year in full text)	Yes, No
Does the publication include a measured (or can be calculated) denominator that fits ALL of the following:<br>1. Data are derived from a general pediatric admission dataset<br>2. Denominator is specific to the same population as the numerator (i.e. if the Numerator is pneumonia hospital admissions for children < 5 years of age, the denominator must be total hospital admissions for children < 5 years of age)	Yes, No
Does the publication include a measured (or can be calculated) numerator defined as AT LEAST ONE of the following?<br>1. Cause of hospital admission<br>2. Cause of inpatient mortality (EXCLUDE hospital-acquired infections/conditions)	Yes, No

Study Characteristics

Year and Month of enrollment start	
Year and Month of enrollment end	
Data source(s) (select all that apply)	Registry/Database, Electronic Medical Record (EMR) Chart Review, Non-electronic medical record Chart Review, Prospective Observation, Other, Not reported
If ‘other’ data source, list here:	
Study design	Retrospective cohort study, Prospective cohort study, Cross-sectional, Before and after intervention study (includes vaccine implementation studies), Surveillance study, Other
If ‘other’ study design, list here:	

Hospital Characteristics

Study site city/region (e.g., Nairobi)	
Study site country (e.g., Kenya)	
Name of hospital	
How is the hospital described? Select all that apply.	Public, Private, Faith-based, Referral, District, Specialty, Tertiary, Community, Academic/University, Not reported, Other
If you selected "other" for hospital description, please provide additional information here:	
Is the hospital a dedicated children's hospital?	Yes, No, Not reported
Study site urban vs. rural	Urban, Rural, Not applicable (Multi-site, aggregated data, mixed urban-rural, etc.), Not reported
Number of health facility beds (total)	
Number of health facility beds (pediatric)	
Are ICU resources available?	Yes, No, Not reported
Are there ICU-trained personnel available?	Yes, No, Not reported
Is a dedicated PICU available?	Yes, No, Not reported
If there is a dedicated PICU, please provide the number of PICU beds:	
What is the population (all ages) served by this hospital/catchment area (number) INCLUDING ADULTS?	
What is the PEDIATRIC population served by this hospital/catchment area (number)?	
Proportion of underlying population (all ages) living in poverty:	
Proportion of underlying population (pediatric only) living in poverty:	

Patient Characteristics

Mean/Median age: estimate, units, dispersion type, dispersion estimate, dispersion lower limit, dispersion upper limit
Minimum and Maximum ages included in the cohort (number and units)
Number of females at baseline

Weight: estimate, units, dispersion type, dispersion estimate, dispersion lower limit, dispersion upper limit	
Anthropometric measure of malnutrition reported (select all that are reported)	MUAC, Weight-for-age, Height-for-age, Weight-for-height, Not reported, Other
MUAC/Weight-for-Age/Height-for-Age/Weight-for-Height: estimate type	Mean, Median, Proportion
MUAC/Weight-for-Age/Height-for-Age/Weight-for-Height: estimate, units, dispersion type, dispersion estimate, dispersion lower limit, dispersion upper limit	
If ‘other’ anthropometric measures of malnutrition reported, please describe here:	
Number with HIV/preterm or premature/congenital heart disease/congenital abnormality/malignancy (any type)/anemia/acute malaria/malnutrition	
Other comorbidities (comorbidity, #, definition)	
If a definition for the comorbidity) is given, please provide it here:	
Is this a cohort study and this population is defined by this comorbidity?	
If this a cohort study, and this population is defined by this comorbidity, how many with this comorbidity died?	

Outcomes

Enter the sample size (denominator) for cause of admission data (entire cohort)	
Is the cause of admission listed by system or diagnosis?	System, Diagnosis
Causes of admission (Systems) (select all that apply)	Cardiac, Respiratory, Gastrointestinal, Renal, Neurological, Infection, Hematologic, Oncologic, Trauma/Injury, Ingestion, Other
Number of causes of admission	1-60
Cause of admission #1-60 (Diagnosis)	Animal Bite, Asthma, Bronchiolitis, Cardiac Disease, Dengue, Diabetes/DKA, Diarrhea, HIV, Ingestion, Malaria, Malnutrition, Meningitis, Pneumonia, Renal Disease, Rheumatic Fever, Sepsis, Surgical Condition, Trauma/Injury, Tuberculosis, Other infection, Other
Cause of admission definition	
Cause of admission observed cases (n)	
How is the cause of admission listed exactly in the text?	
Enter the total number of deaths for the entire cohort (all admission)	
Is the cause of death listed by system or diagnosis?	System, Diagnosis
Causes of death (Systems)	Cardiac, Respiratory, Gastrointestinal, Renal, Neurological, Infection, Hematologic, Oncologic, Trauma/Injury, Ingestion, Other
Number of causes of death	1-60
Cause of death #1-60 (Diagnosis)	Animal Bite, Asthma, Bronchiolitis, Cardiac Disease, Dengue, Diabetes/DKA, Diarrhea, HIV, Ingestion, Malaria, Malnutrition, Meningitis, Pneumonia, Renal Disease, Rheumatic Fever, Sepsis, Surgical Condition, Trauma/Injury, Tuberculosis, Other infection, Other

Cause of death definition	
Cause of death: Number of deaths	
If a case fatality rate cause of death, please provide it here (as a % rounding to the nearest whole number, e.g. 10):	
How is the cause of death listed exactly in the text?	
Length of stay: estimate type	Mean, Median
Length of stay: estimate, units, dispersion type, dispersion estimate, dispersion lower limit, dispersion upper limit	
**<u>Quality and Bias Assessment</u>**	
Study Participation<br>Do those subjects who are enrolled/analysed represent the general admission population of this age group at this facility (or these facilities if multisite)?<br><br>Items for consideration during assessment<br>(a) Adequate participation in the study by eligible persons (i.e. all those admitted in the target age group).<br>(b) Description of the source population or population of interest.<br>(c) Description of the baseline study sample.<br>(d) Adequate description of the sampling frame and recruitment (i.e., if not a census sample, effort was made to ensure a representative sample of the admission population).<br>(e) Adequate description of the period and place of recruitment (ex. representative in terms of seasonality, natural fluctuations in causes based on time of day, day of week, etc.).<br>(f) Adequate description of inclusion and exclusion criteria (i.e. any efforts in sample selection should be to make the sample more representative of the general admission population, not less).	1, High Bias: The relationship between the listed causes and outcome is very likely to be different for participants and eligible nonparticipants<br>2, Moderate Bias: The relationship between the listed causes and outcome may be different for participants and eligible nonparticipants<br>3, Low Bias: The relationship between the listed causes and outcome is unlikely to be different for participants and eligible nonparticipants
**<u>Study Attrition</u>**<br>Key issue in this systematic review: Do those subjects who are enrolled represent those in whom the outcome (cause of admission, cause of death) are measured? This is especially relevant to those studies assessing both causes of admission AND causes of death.<br><br>**<u>Items for consideration during assessment</u>**<br>(a) Those who are enrolled and those in whom a cause (of admission, death, etc.) was measured are the same<br>(b) Reasons for losses between enrollment (admission) and outcome ascertainment (cause of admission, cause of death) are provided.<br>(c) Adequate description of participant losses.<br>(d) There are no important differences between participants who completed the study and those who did not.	1, High Bias: The relationship between the listed causes and outcome is very likely to be different for participants and eligible nonparticipants<br>2, Moderate Bias: The relationship between the listed causes and outcome may be different for participants and eligible nonparticipants<br>3, Low Bias: The relationship between the listed causes and outcome is unlikely to be different for participants and eligible nonparticipants
**<u>Listed Causes Measurement</u>**<br>Key issue in this systematic review: Do those subjects in whom a cause of admission is reported have this cause (or these causes) measured reliably?	1, High Bias: The relationship between the listed causes and outcome is very likely to be different for participants and eligible nonparticipants

Items for consideration during assessment

(a) A clear definition or description of the listed causes is provided.
(b) Method of the determination of causes valid and reliable.
(c) The method and setting of measurement of listed causes is the same for all study participants.
(d) Appropriate methods of imputation are used for missing listed causes data.

2, Moderate Bias: The relationship between the listed causes and outcome may be different for participants and eligible nonparticipants
3, Low Bias: The relationship between the listed causes and outcome is unlikely to be different for participants and eligible nonparticipants

Supplementary Table 8. Risk of bias assessment domains and questions adapted from the Quality in Prognosis Studies (QUIPS) criteria

Domain	Key Issue in this Review	Items for Consideration During Assessment
**Study Participation**	Do those subjects who are enrolled/analyzed represent the general admission population of this age group at this facility (or these facilities if multisite)?	(a) Adequate participation in the study by eligible persons (i.e., all those admitted in the target age group).<br>(b) Description of the source population or population of interest.<br>(c) Description of the baseline study sample.<br>(d) Adequate description of the sampling frame and recruitment (i.e., if not a census sample, effort was made to ensure a representative sample of the admission population).<br>(e) Adequate description of the period and place of recruitment (e.g., representative in terms of seasonality, natural fluctuations in causes based on time of day, day of week, etc.).<br>(f) Adequate description of inclusion and exclusion criteria (i.e., any efforts in sample selection should be to make the sample more representative of the general admission population, not less)
**Study Attrition**	Do those subjects who are enrolled represent those in whom the outcome (cause of admission, cause of death) is measured? This is especially relevant to those studies assessing both causes of admission AND causes of death.	(a) Those who are enrolled and those in whom a cause (of admission, death, etc.) was measured are the same<br>(b) Reasons for losses between enrollment (admission) and outcome ascertainment (cause of admission, cause of death) are provided<br>(c) Adequate description of participant losses<br>(d) There are no important differences between participants who completed the study and those who did not
**Listed Causes of Measurement (i.e., measurement of admission/death)**	Do those subjects in whom a cause of admission/death is reported have this cause (or these causes) measured reliably?	(a) A clear definition or description of the listed causes is provided<br>(b) Method of the determination of causes valid and reliable<br>(c) The method and setting of measurement of listed causes is the same for all study participants<br>(d) Appropriate methods of imputation are used for missing listed causes data

Supplementary Table 9. Included study characteristics by GBD superregion. Studies that contributed data for more than one site are demarcated with an astericks (*).
NR: Not reported

Global Burden of Disease Super Region	Country	Socio-Demographic Index (SDI) Quintile	Study Identification	Study Period	Study Design	Age	Denominator	Risk of Bias for Participants/Attrition /Measurement	Reference
Central Europe, Eastern Europe, and Central Asia	Albania	Middle SDI	Thompson 2019*	2015–2017	Prospective cohort study	Range: 0–11 months	638	Low/Low/Low	(1)
Latin America and Caribbean	Brazil	Middle SDI	Araujo 2017	2014–2014	Cross-sectional	Max: 5 years	627	Low/Low/Low	(2)
Latin America and Caribbean	Brazil	Middle SDI	Berezin 2020	2011–2015	Retrospective cohort study	Range: 0–15 years	124000	High/Moderate/High	(3)
Latin America and Caribbean	Brazil	Middle SDI	Carvalho 2015	2009–2009	Other	Max: 5 years	24413	Low/Low/Low	(4)
Latin America and Caribbean	Guatemala	Low-middle SDI	Chua 2014	2013–2013	Retrospective cohort study	Range: 1 month–12 years	3885	Moderate/Low/High	(5)
Latin America and Caribbean	Brazil	Middle SDI	da Rocha 2012	2005–2008	Cross-sectional	Range: 0–6 years	6201	Low/Low/Low	(6)
Latin America and Caribbean	Brazil	Middle SDI	de Oliveira 2012*	2009–2009	Retrospective cohort study	Max: 5 years	1272	Moderate/Moderate/Low	(7)
Latin America and Caribbean	Brazil	Middle SDI	de Oliveira 2012*	2009–2009	Retrospective cohort study	Max: 5 years	1240	Moderate/Moderate/Low	(7)
Latin America and Caribbean	Brazil	Middle SDI	de Oliveira 2012*	2009–2009	Retrospective cohort study	Max: 5 years	288	Moderate/Moderate/Low	(7)
Latin America and Caribbean	Ecuador	Middle SDI	Diaz-Garrido 2018	2016–2016	Cross-sectional	Range: 0 days - 14 years	8917	Low/Moderate/Moderate	(8)
Latin America and Caribbean	Ecuador	Middle SDI	Gonzalez 2020	2016–2016	Retrospective cohort study	Range: 1 - 12months	645	Moderate/Low/Low	(9)
Latin America and Caribbean	Brazil	Middle SDI	Gouvea 2009	2002–2007	Retrospective cohort study	NR	1100	High/Moderate/High	(10)
Latin America and Caribbean	Brazil	Middle SDI	Jacomin 2020	2008–2012	Surveillance study	Range: 0 days–5 years	70342	Moderate/Moderate/Moderate	(11)
Latin America and Caribbean	Brazil	Middle SDI	Mangia 2011	2002–2006	Retrospective cohort study	Range: 28 days–19 years	16555446	Moderate/Moderate/High	(12)
Latin America and Caribbean	Peru	Middle SDI	Mansilla 2012	2002–2009	Retrospective cohort study	Range: 2–48 months	88718	Low/Moderate/Moderate	(13)
Latin America and Caribbean	Brazil	Middle SDI	Mariano 2018	2012–2012	Cross-sectional	Max: 5 years	32445	Moderate/Moderate/Moderate	(14)

Global Burden of Disease Super Region	Country	Socio-Demographic Index (SDI) Quintile	Study Identification	Study Period	Study Design	Age	Denominator	Risk of Bias for Participants/Attrition /Measurement	Reference
Latin America and Caribbean	Jamaica	Middle SDI	McCarthy 2009	2003–2005	Retrospective cohort study	Range: 0–9 years	3061	Low/Low/Low	(15)
Latin America and Caribbean	Mexico	Middle SDI	Noyola 2007	2003–2005	Cross-sectional	Range: 0–35 months	2036	Low/Low/Moderate	(16)
Latin America and Caribbean	Trinidad and Tobago	Middle SDI	Orrett 2010	2007–2007	Retrospective cohort study	Range: 1 month–12 years	5132	Low/Low/Low	(17)
Latin America and Caribbean	Brazil	Middle SDI	Paulo 2016	2007–2009	Retrospective cohort study	Range: 0 days–60 months	3852	Moderate/Low/Moderate	(18)
Latin America and Caribbean	Venezuela	Middle SDI	Risquez 2014	2011–2011	Cross-sectional	Range: 0 days–18 years	4222	Moderate/Moderate/Moderate	(19)
Latin America and Caribbean	Brazil	Middle SDI	Santos 2021	2014–2018	Prospective cohort study	Mean: 3.9years (95%CI,3.6–4.2)	641	Low/Low/Moderate	(20)
Latin America and Caribbean	Brazil	Middle SDI	Sarni 2009	NR	Prospective cohort study	Range: 1 month–5 years	907	High/High/High	(21)
Latin America and Caribbean	Nicaragua	Low-middle SDI	Thompson 2019*	2015-2017	Prospective cohort study	Range: 0–11 months	307	Low/Low/Low	(1)
Latin America and Caribbean	Haiti	Low SDI	Vinekar 2015	2011–2013	Retrospective cohort study	Range: 0–59 months	31565	Low/Low/Low	(22)
North Africa and Middle East	Yemen	Low SDI	Al Kubati 2018	2012–2015	Cross-sectional	Range: 0 months–14 years	8967	Moderate/Moderate/Moderate	(23)
North Africa and Middle East	Yemen	Low SDI	Al-Taiar 2006	2002–2004	Surveillance study	Range: 6 months–10 years	8068	Low/Moderate/Low	(24)
North Africa and Middle East	Iraq	Low-middle SDI	Ali 2015	2009–2012	Retrospective cohort study	Range: 1 months - 14 years	4785	Low/Moderate/Low	(25)
North Africa and Middle East	Iraq	Low-middle SDI	Al Janabi 2019	2011–2018	Retrospective cohort study	Range: < 1 year -14 years	84914	Low/Low/Low	(26)
North Africa and Middle East	Tunisia	Middle SDI	El Mhamdi 2015	2000–2010	Retrospective cohort study	Range: 0 month –18 years	52443	Low/Low/Low	(27)
North Africa and Middle East	Tunisia	Middle SDI	Maalej 2018	2005–2016	Retrospective cohort study	Range: 2–15 years	17115	Low/Moderate/Low	(28)
North Africa and Middle East	Yemen	Low SDI	Sallam 2005	2000–2003	Retrospective cohort study	Range: 1day - 14 years	4575	Low/Moderate/Low	(29)
North Africa and Middle East	Jordan	Middle SDI	Thompson 2019*	2015-2017	Prospective cohort study	Range: 0–11 months	664	Low/Low/Low	(1)

Global Burden of Disease Super Region	Country	Socio-Demographic Index (SDI) Quintile	Study Identification	Study Period	Study Design	Age	Denominator	Risk of Bias for Participants/Attrition /Measurement	Reference
South Asia	Nepal	Low SDI	Adhikari 2013	2007–2011	Retrospective cohort study	Range: 6 months–15 years	6975	Low/Moderate/Low	(30)
South Asia	India	Low-middle SDI	Ashraf 2016	2012–2013	Retrospective cohort study	Range 0 months - 18 years	28114	Low/Low/Moderate	(31)
South Asia	India	Low-middle SDI	Badhan 2018	2012–2013	Prospective cohort study	Range: 0 months–18 years	3605	Low/Low/Low	(32)
South Asia	India	Low-middle SDI	Basu 2009	2005–2008	Retrospective cohort study	Max: 12 years	17019	Moderate/Low/Moderate	(33)
South Asia	India	Low-middle SDI	Champatiray 2017	2013–2014	Prospective cohort study	Range: 2 months–5 years	10300	Low/Low/Low	(34)
South Asia	Nepal	Low SDI	Chapagain 2020	2019–2019	Prospective cohort study	Range: 1 month–14 years	9725	Low/Low/Low	(35)
South Asia	Nepal	Low SDI	Chaudhary 2017	2014–2016	Cross-sectional	Range: 2 months–16 years	4962	Low/Low/Low	(36)
South Asia	India	Low-middle SDI	Drolia 2010	NR	Prospective cohort study	Range: 2-24 months	9500	Low/Low/Low	(37)
South Asia	India	Low-middle SDI	Duwarah 2016	2005–2010	Retrospective cohort study	Range: 3-18 years	4445	Low/High/Moderate	(38)
South Asia	India	Low-middle SDI	Ganjoo 2014	2011–2012	Prospective cohort study	Range: 0 days–18 years	865	Moderate/High/High	(39)
South Asia	India	Low-middle SDI	Garg 2009*	2006–2007	Retrospective cohort study	Range: 1 month–18 years	245	Moderate/Low/Moderate	(40)
South Asia	India	Low-middle SDI	Garg 2009*	2006-2007	Retrospective cohort study	Range: 3 months–18 years	227	Moderate/High/Moderate	(40)
South Asia	Nepal	Low SDI	Giri 2018	2015–2015	Prospective cohort study	Range: 0–14 years	470	Moderate/Low/Moderate	(41)
South Asia	Nepal	Low SDI	Gupta 2015	2014–2014	Retrospective cohort study	Range: 0 - 5 years.	814	Moderate/Moderate/Moderate	(42)
South Asia	Nepal	Low SDI	Gupta 2018	2014–2016	Retrospective cohort study	Mean (SD): 9.8 years (3.9);	4962	Low/Low/Low	(43)
South Asia	India	Low-middle SDI	Gupta 2019	2014–2015	Cross-sectional	Median (IQR): 52 months (32–165);	9847	Moderate/Low/Low	(44)
South Asia	India	Low-middle SDI	Halder 2020	2011-2013	Cross-sectional	Range: 3 months–12 years	9216	Moderate/Low/Low	(45)

Global Burden of Disease Super Region	Country	Socio-Demographic Index (SDI) Quintile	Study Identification	Study Period	Study Design	Age	Denominator	Risk of Bias for Participants/Attrition/Measurement	Reference
South Asia	India	Low-middle SDI	Jose 2012	2007–2011	Prospective cohort study	Range: 0 days–18 years	25688	Low/Low/Low	(46)
South Asia	India	Low-middle SDI	Mahajan 2014	2009–2012	Retrospective cohort study	Range: 1 month–18 years	5815	Low/Low/Low	(47)
South Asia	Nepal	Low SDI	Malla 2011	2006–2010	Retrospective cohort study	Max: 14 years	5249	Low/Low/Low	(48)
South Asia	Pakistan	Low-middle SDI	Masood 2012	2010–2012	Prospective cohort study	Range: 1 month–12 years	11659	Moderate/Low/Low	(49)
South Asia	India	Low-middle SDI	Mathur 2018	2012–2014	Prospective cohort study	Range: 2–59 months	4354	Moderate/Moderate/Moderate	(50)
South Asia	India	Low-middle SDI	Mishra 2017	2015–2016	Prospective cohort study	Max: 14 years	13312	High/Moderate/Moderate	(51)
South Asia	Bangladesh	Low SDI	Naheed 2009	2004–2007	Surveillance study	Range: 0–59 months	156847	Low/Low/Low	(52)
South Asia	India	Low-middle SDI	Nath Roy 2012	2007–2007	Retrospective cohort study	Range: 0–11 years	3983	Moderate/Moderate/Moderate	(53)
South Asia	Nepal	Low SDI	Rajak 2019	2013–2018	Retrospective cohort study	Range: 0–5 years	11416	Low/Low/Low	(54)
South Asia	India	Low-middle SDI	Rao 2021	2020–2020	Retrospective cohort study	Median: 3 years	969	Low/Low/Low	(55)
South Asia	Pakistan	Low-middle SDI	Rasheed 2017	2016–2016	Cross-sectional	Range: 1 month–15 years	3107	Low/Low/Low	(56)
South Asia	Bangladesh	Low SDI	Rasul 2012	2007–2009	Prospective cohort study	Range: 1 month–12 years	5605	Low/Low/Low	(57)
South Asia	India	Low-middle SDI	Roy 2017	2015–2015	Retrospective cohort study	Range: 5–12 years	3817	Moderate/Moderate/Low	(58)
South Asia	Pakistan	Low-middle SDI	Sadiq 2021	2011–2019	Retrospective cohort study	Range: 1 month–18 years	80913	Low/Low/High	(59)
South Asia	India	Low-middle SDI	Sarangi 2017	2013–2014	Cross-sectional	Range: 1 month–5 years	3146	Low/Low/Low	(60)
South Asia	Nepal	Low SDI	Sharma P 2015	2011–2012	Prospective cohort study	Range: 0 days -14 years	1072	Low/Low/Low	(61)
South Asia	India	Low-middle SDI	Sharma M 2015*	2008–2011	Prospective cohort study	Range: 0 days–18 years	1977	Moderate/Moderate/High	(62)

Global Burden of Disease Super Region	Country	Socio-Demographic Index (SDI) Quintile	Study Identification	Study Period	Study Design	Age	Denominator	Risk of Bias for Participants/Attrition /Measurement	Reference
South Asia	India	Low-middle SDI	Sharma M 2015*	2008–2011	Prospective cohort study	Range: 0 days–18 years	4848	Moderate/Moderate/High	(62)
South Asia	Nepal	Low SDI	Shrestha 2011	2007–2011	Prospective cohort study	Range: 6 months–15 years	2696	Low/Moderate/Low	(63)
South Asia	Nepal	Low SDI	Shrestha 2018	2014–2015	Retrospective cohort study	Range: 1month - 10 year	1862	Low/Low/Low	(64)
South Asia	Bangladesh	Low SDI	Siddique 2011*	2005–2007	Surveillance study	Range: 0 days-59 months	256	Low/Low/Low	(65)
South Asia	Bangladesh	Low SDI	Siddique 2011*	2005–2007	Surveillance study	Range: 0 days -59 months	272	Low/Low/Low	(65)
South Asia	India	Low-middle SDI	Sil 2016	2015–2015	Retrospective cohort study	Range: 1 day–12 years	20883	Low/Low/Low	(66)
South Asia	India	Low-middle SDI	Singh 2006	2001–2002	Prospective cohort study	Range: 1 month - 15 years	2274	High/Moderate/Low	(67)
South Asia	India	Low-middle SDI	Sonowal 2019	2010–2011	Cross-sectional	Range: 0 days--13 years	3808	Low/Moderate/Moderate	(68)
South Asia	Pakistan	Low-middle SDI	van Deursen 2019*	2013–2016	Retrospective cohort study	Range: 0 days–7 months	2551	Low/Low/Low	(69)
South Asia	Pakistan	Low-middle SDI	van Deursen 2019*	2013–2016	Retrospective cohort study	Range: 0 days–6 months	2663	Low/Low/Low	(69)
South Asia	India	Low-middle SDI	Verma 2007*	2005–2005	Retrospective cohort study	Range: 0 days–18 years	912	High/High/High	(70)
South Asia	India	Low-middle SDI	Verma 2007*	2005–2006	Retrospective cohort study	Range: 0 days–18 years	2117	High/High/High	(70)
South Asia	Pakistan	Low-middle SDI	Zaheer 2009	2007–2007	Retrospective cohort study	Range: 1 month–15 years	6089	Low/Low/Low	(71)
Southeast Asia, East Asia, and Oceania	Laos	Low-middle SDI	Barennes 2016	2011–2011	Prospective cohort study	Range: 1 month–15 years	350	Moderate/Low/High	(72)
Southeast Asia, East Asia, and Oceania	Indonesia	Middle SDI	Bucens 2006	2002–2003	Prospective cohort study	Range: 2 months–12 years	880	Low/Moderate/High	(73)
Southeast Asia, East Asia, and Oceania	Timor-Leste	Low-middle SDI	Bucens 2013	2008–2010	Retrospective cohort study	Range: 1 month–15 years	5909	Low/Low/Low	(74)

Global Burden of Disease Super Region	Country	Socio-Demographic Index (SDI) Quintile	Study Identification	Study Period	Study Design	Age	Denominator	Risk of Bias for Participants/Attrition /Measurement	Reference
Southeast Asia, East Asia, and Oceania	Thailand	Middle SDI	Chusilp 2012	2010–2010	Retrospective cohort study	Range: 29 days–12 months	178982	Low/Low/Moderate	(75)
Southeast Asia, East Asia, and Oceania	Papua New Guinea	Low SDI	Duke 2016	2009–2014	Prospective cohort study	NR	96998	Low/Low/Low	(76)
Southeast Asia, East Asia, and Oceania	Vietnam	Middle SDI	Ho 2018	2005–2010	Retrospective cohort study	Max: 15 years	479244	Low/Low/Low	(77)
Southeast Asia, East Asia, and Oceania	Thailand	Middle SDI	Jetsrisuparb 2012	2010–2011	Retrospective cohort study	Range: 1–5 years	486845	Low/Low/Low	(78)
Southeast Asia, East Asia, and Oceania	Sri Lanka	Middle SDI	Kudagammana 2020	2016–2017	Retrospective cohort study	Range: 1 month–14 years	4447	Low/Low/Low	(79)
Southeast Asia, East Asia, and Oceania	Papua New Guinea	Low SDI	Laman 2019	2006–2009	Prospective cohort study	Range: .5–10 years	3019	Moderate/Moderate/Moderate	(80)
Southeast Asia, East Asia, and Oceania	Tonga	Middle SDI	Langridge 2017	2009–2013	Retrospective cohort study	Range: 5–11 years	1816	Low/Low/Low	(81)
Southeast Asia, East Asia, and Oceania	Myanmar	Low-middle SDI	Moe 2005	2002–2003	Prospective cohort study	Range: 1 day–5 years	30869	Moderate/Moderate/Moderate	(82)
Southeast Asia, East Asia, and Oceania	Indonesia	Middle SDI	Murni 2020	2016–2018	Prospective cohort study	Range: ≤ 12 months - > 120 months	1855	Low/Low/Low	(83)
Southeast Asia, East Asia, and Oceania	Vietnam	Middle SDI	Nguyen T 2017	2015–2016	Retrospective cohort study	Max: 14 years	134061	Low/Low/Low	(84)
Southeast Asia, East Asia, and Oceania	Vietnam	Middle SDI	Nguyen N 2017	2007–2014	Retrospective cohort study	Range: 0 days–17 years	199827	Low/Low/Low	(85)
Southeast Asia, East Asia, and Oceania	Vietnam	Middle SDI	Pham 2020	2015–2019	Retrospective cohort study	Range: 0 days–16 years	113999	Low/Low/Low	(86)
Southeast Asia, East Asia, and Oceania	Indonesia	Middle SDI	Poespoprodjo 2009	2004–2008	Retrospective cohort study	Range: 0 days–12 months	4976	Low/Low/Low	(87)
Southeast Asia, East Asia, and Oceania	Papua New Guinea	Low SDI	Rero 2016	2014–2015	Prospective cohort study	Max: 14 years	1061	Low/Low/Moderate	(88)

Global Burden of Disease Super Region	Country	Socio-Demographic Index (SDI) Quintile	Study Identification	Study Period	Study Design	Age	Denominator	Risk of Bias for Participants/Attrition /Measurement	Reference
Southeast Asia, East Asia, and Oceania	Thailand	Middle SDI	Suphakunpinyo 2012	2010–2011	Retrospective cohort study	Range: 6 years–12 years	332234	Low/Low/Moderate	(89)
Southeast Asia, East Asia, and Oceania	Vietnam	Middle SDI	Tan 2018*	2011–2011	Cross-sectional	Range: 1 day–5 years	140561	Moderate/Moderate/High	(90)
Southeast Asia, East Asia, and Oceania	Indonesia	Middle SDI	Tan 2018*	2010–2011	Cross-sectional	Max: 5 years	5263	Moderate/Moderate/Moderate	(90)
Southeast Asia, East Asia, and Oceania	Vietnam	Middle SDI	Thompson 2015	2005–2010	Surveillance study	Median (IQR): 1.2 years (.7–2.1);	479919	Low/Low/Low	(91)
Southeast Asia, East Asia, and Oceania	Philippines	Middle SDI	Thompson 2019*	2015-2017	Prospective cohort study	Range: 0–11 months	334	Low/Low/Low	(1)
Southeast Asia, East Asia, and Oceania	Papua New Guinea	Low SDI	Wandi 2006	2001–2004	Prospective cohort study	Median (IQR): 11 months (5–32);	1313	Moderate/Moderate/Low	(92)
Southeast Asia, East Asia, and Oceania	Indonesia	Middle SDI	Wilopo 2009*	2001–2004	Prospective cohort study	Range: 0 days–35 months	4283	Low/Low/Low	(93)
Southeast Asia, East Asia, and Oceania	Indonesia	Middle SDI	Wilopo 2009*	2001–2004	Prospective cohort study	Range: 0 days–35 months	1537	Low/Low/Low	(93)
Southeast Asia, East Asia, and Oceania	Indonesia	Middle SDI	Wilopo 2009*	2001–2004	Prospective cohort study	Range: 0 days–35 months	3109	Low/Low/Low	(93)
Sub-Saharan Africa	Ethiopia	Low SDI	Abebe 2005	2000–2002	Retrospective cohort study	Range: 1.5 months–16 years	388	Low/Low/Low	(94)
Sub-Saharan Africa	Ghana	Low-middle SDI	Adadey 2019*	2012–2014	Retrospective cohort study	Range: 0 days–5 years	36892	Moderate/Moderate/Moderate	(95)
Sub-Saharan Africa	Ghana	Low-middle SDI	Adadey 2019*	2012–2014	Retrospective cohort study	Range: 0 days–5 years	4798	Moderate/Moderate/Moderate	(95)
Sub-Saharan Africa	Ghana	Low-middle SDI	Adadey 2019*	2012–2014	Retrospective cohort study	Max: 5 years	12638	Moderate/Moderate/Moderate	(95)
Sub-Saharan Africa	Ghana	Low-middle SDI	Adadey 2019*	2012–2014	Retrospective cohort study	Max: 5 years	14402	Moderate/Moderate/Moderate	(95)
Sub-Saharan Africa	Nigeria	Low-middle SDI	Adeboye 2010	2001–2001	Prospective cohort study	Minimum: 29 days	606	Low/Low/Low	(96)

Global Burden of Disease Super Region	Country	Socio-Demographic Index (SDI) Quintile	Study Identification	Study Period	Study Design	Age	Denominator	Risk of Bias for Participants/Attrition /Measurement	Reference
Sub-Saharan Africa	Niger	Low SDI	Adeboye 2011	2007–2008	Prospective cohort study	Range: < 1 year - 12 years	1364	High/Moderate/Low	(97)
Sub-Saharan Africa	Nigeria	Low-middle SDI	Adeboye 2015	2004-2004	Cross-sectional	Range: 6 months–5 years	1675	Low/Low/Low	(98)
Sub-Saharan Africa	Nigeria	Low-middle SDI	Adegoke 2010	2007–2008	Cross-sectional	Range:6 weeks - 15 years	1193	Low/Low/Low	(99)
Sub-Saharan Africa	Nigeria	Low-middle SDI	Adegoke 2012	2010–2010	Cross-sectional	Range: 2 months–161 months	1735	Moderate/Moderate/Moderate	(100)
Sub-Saharan Africa	Nigeria	Low-middle SDI	Adekanmbi 2007	2002–2003	Prospective cohort study	Range: 0 days–14 years	1552	Low/Low/Low	(101)
Sub-Saharan Africa	Nigeria	Low-middle SDI	Adeleke 2010	1999–2006	Retrospective cohort study	Mean: 4.51 years	3585	High/Moderate/Moderate	(102)
Sub-Saharan Africa	Ethiopia	Low SDI	Adem 2020	2018–2018	Prospective cohort study	Range: 6 months–59 months	892	Moderate/Moderate/Moderate	(103)
Sub-Saharan Africa	Nigeria	Low-middle SDI	Ademola 2019	2016–2017	Prospective cohort study	Mean: 2.2 years	628	Moderate/Moderate/Moderate	(104)
Sub-Saharan Africa	Nigeria	Low-middle SDI	Adeyeye 2007	2000–2001	Cross-sectional	Range: 0 days–15 years	1226	Low/Low/Low	(105)
Sub-Saharan Africa	Benin	Low SDI	Agbeille 2019	2017–2017	Cross-sectional	Range: 8 months–98 months	804	Moderate/Low/Low	(106)
Sub-Saharan Africa	Nigeria	Low-middle SDI	Ahmed 2010	2002–2005	Retrospective cohort study	Range: 7 months-120 months.	2650	High/Moderate/Moderate	(107)
Sub-Saharan Africa	Mauritania	Low-middle SDI	Ahmed 2018	2011–2014	Retrospective cohort study	Range: 0 days–5 years	3695	Low/Low/Low	(108)
Sub-Saharan Africa	Mauritania	Low-middle SDI	Ahmed 2019	2015–2017	Retrospective cohort study	Range: 0 days –5 years	10480	Low/Low/Low	(109)
Sub-Saharan Africa	Kenya	Low-middle SDI	Akech 2019	2015–2018	Surveillance study	Range: 1 month - 15 years	14999	Low/Low/High	(110)
Sub-Saharan Africa	Nigeria	Low-middle SDI	Akinbami 2010	2007–2007	Prospective cohort study	Mean (SD): 26.5 months (12.3);	164	High/Moderate/High	(111)
Sub-Saharan Africa	Democratic Republic of the Congo	Low SDI	Aloni 2012	2000–2007	Retrospective cohort study	Range 1 years-13 years	5988	Low/Moderate/Low	(112)
Sub-Saharan Africa	Ethiopia	Low SDI	Amare 2018	2011–2013	Retrospective cohort study	Range: 2 months–14 years	4996	Moderate/Moderate/Moderate	(113)

Global Burden of Disease Super Region	Country	Socio-Demographic Index (SDI) Quintile	Study Identification	Study Period	Study Design	Age	Denominator	Risk of Bias for Participants/Attrition /Measurement	Reference
Sub-Saharan Africa	Guinea-Bissau	Low SDI	Andersen 2017	2001–2008	Retrospective cohort study	Range:6 weeks–8 months	4230	Low/Low/High	(114)
Sub-Saharan Africa	Nigeria	Low-middle SDI	Animasahun 2015	2011–2012	Prospective cohort study	Range: 1 day–12 years	5705	Low/Low/Low	(115)
Sub-Saharan Africa	Kenya	Low-middle SDI	Ayieko 2016*	2014–2015	Surveillance study	Range: 2 months - 15 years	26987	Moderate/Moderate/Moderate	(116)
Sub-Saharan Africa	Kenya	Low-middle SDI	Ayieko 2016*	2014–2015	Surveillance study	Range: 2 months–15 years	28689	Moderate/Moderate/Moderate	(116)
Sub-Saharan Africa	Mozambique	Low SDI	Bassat 2008	2003–2005	Surveillance study	Range: 0 days–15 years	8311	Low/Low/Low	(117)
Sub-Saharan Africa	Nigeria	Low-middle SDI	Belonwu 2008	1999–2005	Retrospective cohort study	Range: 4months - 8 years	4113	Low/Low/Low	(118)
Sub-Saharan Africa	Ethiopia	Low SDI	Berti 2008	2006–2006	Retrospective cohort study	Range: 1 day–5 years	1635	Moderate/Moderate/Moderate	(119)
Sub-Saharan Africa	Democratic Republic of the Congo	Low SDI	Birindwa 2020*	2010–2015	Retrospective cohort study	Range: 2 months–59 months	1114	Moderate/Moderate/Moderate	(120)
Sub-Saharan Africa	Democratic Republic of the Congo	Low SDI	Birindwa 2020*	2010–2015	Retrospective cohort study	Range: 2 months–59 months	556	Moderate/Moderate/Moderate	(120)
Sub-Saharan Africa	Democratic Republic of the Congo	Low SDI	Birindwa 2020*	2010–2015	Retrospective cohort study	Range: 2months–59 months	2033	Moderate/Moderate/Moderate	(120)
Sub-Saharan Africa	Democratic Republic of the Congo	Low SDI	Birindwa 2020*	2010–2015	Retrospective cohort study	Range: 2 months–59 months	1202	Moderate/Moderate/Moderate	(120)
Sub-Saharan Africa	Ethiopia	Low SDI	Bohn 2016	2011–2014	Retrospective cohort study	Range: 29 days–14 years	6866	Low/Low/Low	(121)
Sub-Saharan Africa	Gabon	Middle SDI	Bouyou-Akotet 2012	2008–2008	Cross-sectional	Range: 1 month - 192 month	804	Moderate/Moderate/Moderate	(122)
Sub-Saharan Africa	Uganda	Low SDI	Boyle 2017	2009–2014	Retrospective cohort study	Range: 1 day–5 years	3428	Low/Low/Low	(123)
Sub-Saharan Africa	Uganda	Low SDI	Boyle 2020	2009–2014	Retrospective cohort study	Mean (SD): 2.3 years (0.97)	3428	Low/Low/Low	(124)

Global Burden of Disease Super Region	Country	Socio-Demographic Index (SDI) Quintile	Study Identification	Study Period	Study Design	Age	Denominator	Risk of Bias for Participants/Attrition /Measurement	Reference
Sub-Saharan Africa	South Africa	Middle SDI	Brits 2020	2016–2017	Retrospective cohort study	Range: 2 months–71 months	1352	Low/Low/Low	(125)
Sub-Saharan Africa	Mozambique	Low SDI	Brugnolaro 2020	2017–2018	Retrospective cohort study	Range: 0 days–15 years	4997	Moderate/High/High	(126)
Sub-Saharan Africa	Tanzania	Low SDI	Chami 2019	2014–2014	Prospective cohort study	Range: 2 years–12 years	505	Low/Moderate/Moderate	(127)
Sub-Saharan Africa	Nigeria	Low-middle SDI	Charles 2014	2004–2008	Retrospective cohort study	Range: 1 month–15 years	3814	Low/Low/Low	(128)
Sub-Saharan Africa	Cameroon	Low-middle SDI	Chelo 2020	2020–2020	Retrospective cohort study	NR	1701	Low/Low/Low	(129)
Sub-Saharan Africa	Cameroon	Low-middle SDI	Chiabi 2009	2007–2007	Cross-sectional	Range: 1 month–15 years	1060	Low/Moderate/Low	(130)
Sub-Saharan Africa	Cameroon	Low-middle SDI	Chiabi 2017	2006–2015	Retrospective cohort study	Range: 23 days - 112 months	17981	High/High/High	(131)
Sub-Saharan Africa	Cameroon	Low-middle SDI	Chiabi 2020	2015–2016	Cross-sectional	Range: 3 months–15 years	1782	Low/Low/Low	(132)
Sub-Saharan Africa	Liberia	Low SDI	Couto 2013	2009–2009	Retrospective cohort study	Range: 0 days–15 years	8254	Moderate/Moderate/Low	(133)
Sub-Saharan Africa	Nigeria	Low-middle SDI	Edelu 2018	2016–2016	Cross-sectional	Range: 1 month–15 years	1848	Moderate/Moderate/Moderate	(134)
Sub-Saharan Africa	Kenya	Low-middle SDI	Ekaru 2012	NR	Prospective cohort study	Range: 1 year–5 years	1882	Moderate/Moderate/Low	(135)
Sub-Saharan Africa	Nigeria	Low-middle SDI	Ekenze 2009	2002–2007	Retrospective cohort study	Range: 1 month–15 years	6156	Moderate/Moderate/Moderate	(136)
Sub-Saharan Africa	Nigeria	Low-middle SDI	Enyuma 2019	2014–2014	Cross-sectional	Range: 1 day–12 years	633	Low/Low/Low	(137)
Sub-Saharan Africa	Nigeria	Low-middle SDI	Eseigbe 2012	2008–2010	Retrospective cohort study	Range: 9 months–5 years	635	Low/Low/Low	(138)
Sub-Saharan Africa	Nigeria	Low-middle SDI	Esezobor 2012	2010–2012	Retrospective cohort study	Range: 1 month–16 years	4015	Low/Low/Low	(139)
Sub-Saharan Africa	Ethiopia	Low SDI	Eshetie 2015	2011–2011	Prospective cohort study	Range: 7 days–14 years	634	Moderate/Low/Moderate	(140)
Sub-Saharan Africa	Malawi	Low SDI	Evans 2018	2016–2016	Prospective cohort study	Median (IQR): 4 years (1–8);	412	Moderate/Low/Moderate	(141)

Global Burden of Disease Super Region	Country	Socio-Demographic Index (SDI) Quintile	Study Identification	Study Period	Study Design	Age	Denominator	Risk of Bias for Participants/Attrition /Measurement	Reference
Sub-Saharan Africa	Nigeria	Low-middle SDI	Fadero 2012	2009–2009	Retrospective cohort study	Range: 1month - 5 years	1132	Moderate/High/Moderate	(142)
Sub-Saharan Africa	Nigeria	Low-middle SDI	Forae 2014	2007–2011	Retrospective cohort study	Range: 0 days–17 years	12442	Low/Low/Low	(143)
Sub-Saharan Africa	Zimbabwe	Low-middle SDI	Gapu 2015	2012–2013	Cross-sectional	Range: 1–12 years	2601	Low/Low/Low	(144)
Sub-Saharan Africa	Nigeria	Low-middle SDI	Garba 2017	2013–2016	Retrospective cohort study	Range: 1 month - 14 years	2658	Low/Moderate/Moderate	(145)
Sub-Saharan Africa	Kenya	Low-middle SDI	Gardner 2020	2014–2018	Retrospective cohort study	Range: 30 days - 5 years	2203	Moderate/Moderate/High	(146)
Sub-Saharan Africa	Kenya	Low-middle SDI	Gathara 2017*	2013–2015	Retrospective cohort study	Range: 2months–59 months	4757	Low/Moderate/Low	(147)
Sub-Saharan Africa	Kenya	Low-middle SDI	Gathara 2017*	2013–2015	Retrospective cohort study	Range: 2months–59 months	2445	Low/Moderate/Low	(147)
Sub-Saharan Africa	Kenya	Low-middle SDI	Gathara 2017*	2013–2015	Retrospective cohort study	Range: 2months–59 months	4175	Low/Moderate/Low	(147)
Sub-Saharan Africa	Kenya	Low-middle SDI	Gathara 2017*	2013–2015	Retrospective cohort study	Range: 2months–59 months	2440	Low/Moderate/Low	(147)
Sub-Saharan Africa	Kenya	Low-middle SDI	Gathara 2017*	2013–2015	Retrospective cohort study	Range: 2months–59 months	2209	Low/Moderate/Low	(147)
Sub-Saharan Africa	Kenya	Low-middle SDI	Gathara 2017*	2013–2015	Retrospective cohort study	Range: 2months–59 months	2146	Low/Moderate/Low	(147)
Sub-Saharan Africa	Kenya	Low-middle SDI	Gathara 2017*	2013–2015	Retrospective cohort study	Range: 2months–59 months	3066	Low/Moderate/Low	(147)
Sub-Saharan Africa	Kenya	Low-middle SDI	Gathara 2017*	2013–2015	Retrospective cohort study	Range: 2months–59 months	3270	Low/Moderate/Low	(147)
Sub-Saharan Africa	Kenya	Low-middle SDI	Gathara 2017*	2013–2015	Retrospective cohort study	Range: 2months–59 months	1853	Low/Moderate/Low	(147)
Sub-Saharan Africa	Kenya	Low-middle SDI	Gathara 2017*	2013–2015	Retrospective cohort study	Range: 2months–59 months	1881	Low/Moderate/Low	(147)
Sub-Saharan Africa	Kenya	Low-middle SDI	Gathara 2017*	2013–2015	Retrospective cohort study	Range: 2months–59 months	1982	Low/Moderate/Low	(147)

Global Burden of Disease Super Region	Country	Socio-Demographic Index (SDI) Quintile	Study Identification	Study Period	Study Design	Age	Denominator	Risk of Bias for Participants/Attrition /Measurement	Reference
Sub-Saharan Africa	Kenya	Low-middle SDI	Gathara 2017*	2013–2015	Retrospective cohort study	Range: 2months–59 months	3517	Low/Moderate/Low	(147)
Sub-Saharan Africa	Ethiopia	Low SDI	Gebremariam 2016	2012–2015	Retrospective cohort study	Range: 2 months–14 years	3672	Low/Moderate/Low	(148)
Sub-Saharan Africa	Nigeria	Low-middle SDI	George 2009	2007–2008	Cross-sectional	Range: 2 months-10years	2174	Low/Low/Low	(149)
Sub-Saharan Africa	Nigeria	Low-middle SDI	George 2010	2008–2008	Cross-sectional	Range: 1 month–16 years	2009	Low/Low/Moderate	(150)
Sub-Saharan Africa	Ethiopia	Low SDI	Gordon 2013	2009–2010	Cross-sectional	Median (IQR): 2.2 years (1–7)	1927	Low/Low/Low	(151)
Sub-Saharan Africa	Nigeria	Low-middle SDI	Graham 2019	2015–2017	Prospective cohort study	Range: 28 days - 14 years	16453	Low/Low/Low	(152)
Sub-Saharan Africa	Nigeria	Low-middle SDI	Graham 2020	2015–2017	Prospective cohort study	Range: 28 days–15 years	16184	Low/Low/Low	(153)
Sub-Saharan Africa	Kenya	Low-middle SDI	Gwer 2012	2004–2009	Retrospective cohort study	Median (IQR): 32 months (20-46)	28517	Low/Low/Low	(154)
Sub-Saharan Africa	Kenya	Low-middle SDI	Hammitt 2012	2010–2010	Prospective cohort study	Range: 1month–59 months	2606	Moderate/Moderate/Low	(155)
Sub-Saharan Africa	Malawi	Low SDI	Harris 2019	2015–2016	Prospective cohort study	Range : 0 days - 12years	13827	Low/Low/Low	(156)
Sub-Saharan Africa	Tanzania	Low SDI	Hau 2018	2014–2014	Prospective cohort study	Range: 2 years–12 years	506	Moderate/Moderate/High	(157)
Sub-Saharan Africa	Liberia	Low SDI	Huerga 2009	2005–2005	Retrospective cohort study	Range: 1 month–14 years	1509	Low/Low/Low	(158)
Sub-Saharan Africa	Nigeria	Low-middle SDI	Ibekwe 2011	2007–2007	Retrospective cohort study	Minimum: 2 months	673	Low/Low/Low	(159)
Sub-Saharan Africa	Kenya	Low-middle SDI	Idro 2008	2004–2006	Prospective cohort study	Range: 0 days–13 years	4921	High/Low/Low	(160)
Sub-Saharan Africa	South Africa	Middle SDI	Isaacs-Long 2017	2013–2013	Retrospective cohort study	Median (IQR): 3 years (1–6.4);	21751	Low/Low/Low	(161)
Sub-Saharan Africa	Nigeria	Low-middle SDI	John 2013	2011–2012	Retrospective cohort study	Range: 5 years-17 years	857	Low/Low/Low	(162)
Sub-Saharan Africa	Kenya	Low-middle SDI	Karuri 2017	2014–2015	Retrospective cohort study	Range: 1 month–5 years	20528	Low/Low/High	(163)

Global Burden of Disease Super Region	Country	Socio-Demographic Index (SDI) Quintile	Study Identification	Study Period	Study Design	Age	Denominator	Risk of Bias for Participants/Attrition /Measurement	Reference
Sub-Saharan Africa	Uganda	Low SDI	Kiggundu 2013*	2011–2012	Cross-sectional	Range: 0 days–5 years	800	Moderate/Low	(164)
Sub-Saharan Africa	Uganda	Low SDI	Kiggundu 2013*	2011–2012	Cross-sectional	Range: 0–4 years	1671	Low/Low/Low	(164)
Sub-Saharan Africa	Uganda	Low SDI	Ku 2020	2009–2014	Retrospective cohort study	Mean (SD): 19.8 months (13.9);	3428	Moderate/Moderate/Moderate	(165)
Sub-Saharan Africa	The Gambia	Low SDI	Kuti 2013	2010–2011	Prospective cohort study	Range: 2months–59 months	1517	Low/Low/Low	(166)
Sub-Saharan Africa	The Gambia	Low SDI	Kuti 2014	2010–2011	Prospective cohort study	Range: 2months–59 months	1517	Low/Low/Low	(167)
Sub-Saharan Africa	Nigeria	Low-middle SDI	Kuti 2015	2011–2013	Retrospective cohort study	Range: 1 month–15 years	1470	Low/Low/Low	(168)
Sub-Saharan Africa	Uganda	Low SDI	Lamorde 2018*	2017–2017	Surveillance study	Max: 14 years	1153	Low/Moderate/Low	(169)
Sub-Saharan Africa	Uganda	Low SDI	Lamorde 2018*	2017–2017	Surveillance study	Max: 14 years	1019	Low/Moderate/Low	(169)
Sub-Saharan Africa	Uganda	Low SDI	Lamorde 2018*	2016–2017	Surveillance study	Range: 1 day–14 years	4731	Low/Moderate/Low	(169)
Sub-Saharan Africa	Uganda	Low SDI	Lamorde 2018*	2016–2017	Surveillance study	Range: 1 day–14 years	9667	Low/Low/Low	(169)
Sub-Saharan Africa	Uganda	Low SDI	Lamorde 2018*	2017–2017	Surveillance study	Max: 14 years	690	Low/Moderate/Low	(169)
Sub-Saharan Africa	Uganda	Low SDI	Lamorde 2018*	2016–2017	Surveillance study	Range: 1 day–14 years	3939	Low/Moderate/Low	(169)
Sub-Saharan Africa	Cameroon	Low-middle SDI	Libwea 2019	2006–2012	Retrospective cohort study	Range: 1–59 months	85000	Low/Low/Low	(170)
Sub-Saharan Africa	Democratic Republic of the Congo	Low SDI	Likwela 2012	2010–2011	Prospective cohort study	NR	1154	Low/Moderate/Moderate	(171)
Sub-Saharan Africa	Uganda	Low SDI	Lowlaavar 2016	2012–2013	Prospective cohort study	Range: 6 months–5 years	1824	Moderate/Low/High	(172)
Sub-Saharan Africa	Tanzania	Low SDI	Lugangira 2017	2015–2015	Retrospective cohort study	Range: 2–59 months	1130	Low/Low/Low	(173)
Sub-Saharan Africa	Tanzania	Low SDI	Lundgren 2015	2006–2009	Prospective cohort study	Max: 60 months	909	Moderate/Moderate/Moderate	(174)
Sub-Saharan Africa	Senegal	Low SDI	Ly 2019	2013–2015	Retrospective cohort study	Range: 7–180 months	2537	Moderate/Low/Low	(175)

Global Burden of Disease Super Region	Country	Socio-Demographic Index (SDI) Quintile	Study Identification	Study Period	Study Design	Age	Denominator	Risk of Bias for Participants/Attrition /Measurement	Reference
Sub-Saharan Africa	Kenya	Low-middle SDI	Macharia 2017	2000–2004	Surveillance study	Range: 0 days- 13 years	18873	Low/Low/Low	(176)
Sub-Saharan Africa	Mozambique	Low SDI	Madrid 2016	2001–2013	Retrospective cohort study	Mean: 18 months;	45573	Moderate/Moderate/Moderate	(177)
Sub-Saharan Africa	Sudan	Low-middle SDI	Mahgoub 2012	2007–2009	Retrospective cohort study	Range: 6–60 months	4020	Low/Low/Low	(178)
Sub-Saharan Africa	Kenya	Low-middle SDI	Maitland 2006	2000–2002	Prospective cohort study	Minimum: 3 months	7869	High/High/Moderate	(179)
Sub-Saharan Africa	Kenya	Low-middle SDI	Maitland 2019	2002–2009	Retrospective cohort study	Range: 60 days–15 years	29226	Low/Low/Low	(180)
Sub-Saharan Africa	Malawi	Low SDI	McCollum 2013	2011–2011	Prospective cohort study	Range: 0 years–15 years	761	Low/Moderate/Low	(181)
Sub-Saharan Africa	Namibia	Middle SDI	Mdala 2015	2013–2013	Prospective cohort study	Range: 8 days–5 years	4898	Low/Moderate/Moderate	(182)
Sub-Saharan Africa	Tanzania	Low SDI	Mhando 2008	2004–2005	Retrospective cohort study	Range: 1 day–14 years	2824	Low/Low/Low	(183)
Sub-Saharan Africa	Kenya	Low-middle SDI	Migowa 2017	2011–2011	Retrospective cohort study	Range: 3 months - 15 years	8011	Moderate/Moderate/Low	(184)
Sub-Saharan Africa	Madagascar	Low SDI	Mioramalala 2018	2012–2015	Retrospective cohort study	Range: 3–59 months	13073	Low/Low/Low	(185)
Sub-Saharan Africa	Tanzania	Low SDI	Mitchell 2013	2010–2011	Retrospective cohort study	NR	5244	Low/Low/Low	(186)
Sub-Saharan Africa	Burundi	Low SDI	Moise 2018	2010–2010	Retrospective cohort study	Range: 1–59 months	11632	Low/Low/Low	(187)
Sub-Saharan Africa	Ethiopia	Low SDI	Mola 2016	2012–2015	Retrospective cohort study	Range: 15 days - 17 years	14521	Low/Low/Moderate	(188)
Sub-Saharan Africa	Zimbabwe	Low-middle SDI	Mujuru 2012	2004–2005	Prospective cohort study	Median (IQR): 16 months (4–36);	737	High/High/High	(189)
Sub-Saharan Africa	Nigeria	Low-middle SDI	Muoneke 2016	2011–2013	Retrospective cohort study	Max: 17 years	1780	Low/Low/Low	(190)
Sub-Saharan Africa	Tanzania	Low SDI	Muro 2020	2013–2014	Prospective cohort study	Range: 2–59 months	978	Low/Low/Low	(191)

Global Burden of Disease Super Region	Country	Socio-Demographic Index (SDI) Quintile	Study Identification	Study Period	Study Design	Age	Denominator	Risk of Bias for Participants/Attrition /Measurement	Reference
Sub-Saharan Africa	Democratic Republic of the Congo	Low SDI	Mutombo 2018	2014–2016	Cross-sectional	Max: 5 years	3092	Low/Low/Low	(192)
Sub-Saharan Africa	Kenya	Low-middle SDI	Mwangome 2017	2007–2013	Retrospective cohort study	Range: 1–6 months	2882	Low/Moderate/Low	(193)
Sub-Saharan Africa	Kenya	Low-middle SDI	Mwaniki 2009	2002–2004	Retrospective cohort study	Median (IQR): 32 months (11–42);	13183	Low/Low/Low	(194)
Sub-Saharan Africa	South Africa	Middle SDI	Nabukeera-Barungi 2014	2009–2009	Retrospective cohort study	Range: 42 days-12years	22943	Low/Low/Low	(195)
Sub-Saharan Africa	Uganda	Low SDI	Nakawesi 2010	2006–2007	Cross-sectional	Range: 3–59 months	5230	Low/Low/Low	(196)
Sub-Saharan Africa	Nigeria	Low-middle SDI	Ndukwu 2015	2012–2014	Retrospective cohort study	Mean (SD): 50 months (113)	1964	Low/Moderate/Moderate	(197)
Sub-Saharan Africa	Kenya	Low-middle SDI	Ngari 2017	2007–2012	Surveillance study	Range: 1–59 months	13256	Low/Low/Low	(198)
Sub-Saharan Africa	Kenya	Low-middle SDI	Ngari 2021	2007–2016	Retrospective cohort study	Range: 60–155 months	3907	Low/Low/Moderate	(199)
Sub-Saharan Africa	Rwanda	Low SDI	Ngirabega 2011	2008–2009	Prospective cohort study	Range: 6–59 months	810	Low/Low/Low	(200)
Sub-Saharan Africa	Somalia	Low SDI	Ngoy 2013	2010–2011	Cross-sectional	Range: 0–15 years	6211	Low/Low/Low	(201)
Sub-Saharan Africa	Mozambique	Low SDI	Nhampossa 2013	2001–2010	Retrospective cohort study	Max: 5 years	16843	Moderate/Moderate/High	(202)
Sub-Saharan Africa	Ethiopia	Low SDI	Nigussie 2019	2014–2016	Retrospective cohort study	Range: 2 months–14 years	2000	Moderate/Moderate/Low	(203)
Sub-Saharan Africa	Kenya	Low-middle SDI	Njuguna 2019	1989–2016	Retrospective cohort study	Range: 14 days–14 years	99126	Moderate/Low/Low	(204)
Sub-Saharan Africa	Kenya	Low-middle SDI	Nokes 2008	2002–2004	Retrospective cohort study	Range: 0 days–13 years	15347	Low/Low/Moderate	(205)
Sub-Saharan Africa	Kenya	Low-middle SDI	Nokes 2009	2002–2007	Surveillance study	Max: 59 months	25149	Low/Low/Moderate	(206)
Sub-Saharan Africa	Kenya	Low-middle SDI	Nyaga 2010	2005–2006	Prospective cohort study	Range: 1–13 years	12000	High/High/High	(207)
Sub-Saharan Africa	Kenya	Low-middle SDI	Obonyo 2007	2002–2002	Retrospective cohort study	Range: 1 month - 60 month	1116	Moderate/Moderate/Moderate	(208)

Global Burden of Disease Super Region	Country	Socio-Demographic Index (SDI) Quintile	Study Identification	Study Period	Study Design	Age	Denominator	Risk of Bias for Participants/Attrition /Measurement	Reference
Sub-Saharan Africa	Uganda	Low SDI	Obura 2020	2019–2019	Prospective cohort study	Range: 0 days–59 months	427	High/Moderate/High	(209)
Sub-Saharan Africa	Nigeria	Low-middle SDI	Odetunde 2014	2007–2012	Retrospective cohort study	Range: < 5years - 16 years	3002	Low/Low/Moderate	(210)
Sub-Saharan Africa	Nigeria	Low-middle SDI	Ofovwe 2005	1996–2001	Retrospective cohort study	Range: 1month - 16 years	1027	Low/Low/Low	(211)
Sub-Saharan Africa	Nigeria	Low-middle SDI	Ogunfowora 2019	2010–2017	Retrospective cohort study	Range: 28 days–15 years	3986	Low/Low/Low	(212)
Sub-Saharan Africa	Nigeria	Low-middle SDI	Okike 2020	2008–2016	Retrospective cohort study	Mean (SD): 8.9 years (3.1)	3693	Low/Low/Low	(213)
Sub-Saharan Africa	Malawi	Low SDI	Okiro 2013*	2000–2010	Retrospective cohort study	Range: 0 days–5 years	2559	Moderate/Moderate/Low	(214)
Sub-Saharan Africa	Malawi	Low SDI	Okiro 2013*	2000–2010	Retrospective cohort study	Range: 0 days–5 years	16712	Moderate/Moderate/Low	(214)
Sub-Saharan Africa	Malawi	Low SDI	Okiro 2013*	2000–2010	Retrospective cohort study	Range: 0 days–5 years	6408	Moderate/Moderate/Low	(214)
Sub-Saharan Africa	Malawi	Low SDI	Okiro 2013*	2000–2010	Retrospective cohort study	Range: 0 days–5 years	4822	Moderate/Moderate/Low	(214)
Sub-Saharan Africa	Nigeria	Low-middle SDI	Okoroiwu 2020	2012–2017	Retrospective cohort study	Range: 0–14 years	14370	Low/Low/Low	(215)
Sub-Saharan Africa	Nigeria	Low-middle SDI	Okoronkwo 2018	2012–2014	Retrospective cohort study	Max: 5 years	2278	Moderate/Moderate/Moderate	(216)
Sub-Saharan Africa	Nigeria	Low-middle SDI	Olatunya 2015	2011–2014	Retrospective cohort study	Range: 1.5 months–15 years	5256	Moderate/Moderate/Moderate	(217)
Sub-Saharan Africa	Kenya	Low-middle SDI	Oliwa 2019	2015–2018	Prospective cohort study	Median (IQR): 19 months (9–47);	42107	Low/Low/Low	(218)
Sub-Saharan Africa	Nigeria	Low-middle SDI	Olorunmoteni 2020	2016–2016	Prospective cohort study	Range: 3-13 years	641	Moderate/Moderate/Moderate	(219)
Sub-Saharan Africa	Nigeria	Low-middle SDI	Olowu 2012	2004–2008	Retrospective cohort study	Max: 17 years	3286	Moderate/Low/Moderate	(220)
Sub-Saharan Africa	Uganda	Low SDI	Olupot-Olupot 2020	2011–2012	Cross-sectional	Range: 2 months–12 years	10208	Moderate/Moderate/Moderate	(221)

Global Burden of Disease Super Region	Country	Socio-Demographic Index (SDI) Quintile	Study Identification	Study Period	Study Design	Age	Denominator	Risk of Bias for Participants/Attrition /Measurement	Reference
Sub-Saharan Africa	Nigeria	Low-middle SDI	Oluwayemi 2013	2009–2010	Prospective cohort study	Range: 2–128 months	1202	Low/Low/Low	(222)
Sub-Saharan Africa	Kenya	Low-middle SDI	Omore 2019	2010–2013	Prospective cohort study	Range: 0–59 months	3793	Low/Low/Low	(223)
Sub-Saharan Africa	Uganda	Low SDI	Opoka 2018	2016–2017	Retrospective cohort study	Range: 0 days–5 years	2275	Moderate/Moderate/Moderate	(224)
Sub-Saharan Africa	Nigeria	Low-middle SDI	Orimadegun 2007	2000–2005	Retrospective cohort study	Range: 6 months–15 years	16031	Low/Moderate/Low	(225)
Sub-Saharan Africa	Kenya	Low-middle SDI	Osano 2017	2013–2013	Retrospective cohort study	Range: 5–17 years	4520	Low/Low/Low	(226)
Sub-Saharan Africa	Nigeria	Low-middle SDI	Oshikoya 2020	2017–2018	Retrospective cohort study	Range: 1–15 years	4812	High/High/High	(227)
Sub-Saharan Africa	Burkina Faso	Low SDI	Ouedraogo 2010	2005–2006	Retrospective cohort study	Mean: 2.1 years;	5803	Low/Low/Low	(228)
Sub-Saharan Africa	Nigeria	Low-middle SDI	Oyedeji 2012	2006–2008	Prospective cohort study	Range: 0–15 years	1681	Low/Low/Low	(229)
Sub-Saharan Africa	Angola	Low-middle SDI	Pinto 2008	2004–2005	Cross-sectional	Range: 0 days–11 years	1322	High/High/High	(230)
Sub-Saharan Africa	Madagascar	Low SDI	Rahajamanana 2018	2014–2016	Surveillance study	Range: 0 days–5 years	5821	Low/Low/Moderate	(231)
Sub-Saharan Africa	South Africa	Middle SDI	Richards 2020	2013–2018	Before and after intervention study (includes vaccine implementation studies)	Range: 2 months - 13 years	8733	Low/Low/Low	(232)
Sub-Saharan Africa	Mozambique	Low SDI	Roca 2008	2001–2005	Surveillance study	Range: 0 days–5 years	18373	High/High/High	(233)
Sub-Saharan Africa	Uganda	Low SDI	Rudd 2017	2013–2013	Prospective cohort study	Median (IQR): 3 (2-7years)	115	Moderate/Low/High	(234)
Sub-Saharan Africa	Cameroon	Low-middle SDI	Sap 2020	2013–2017	Retrospective cohort study	Median (IQR): 8 years (6–11)	164	Low/Low/Low	(235)
Sub-Saharan Africa	Burkina Faso	Low SDI	Sawadogo 2020	2016–2016	Cross-sectional	Range: 0–14 years	882	Moderate/Moderate/Moderate	(236)
Sub-Saharan Africa	Senegal	Low SDI	Seck 2018	2017–2017	Cross-sectional	Range: 1–24 months	1328	Low/Low/Low	(237)

Global Burden of Disease Super Region	Country	Socio-Demographic Index (SDI) Quintile	Study Identification	Study Period	Study Design	Age	Denominator	Risk of Bias for Participants/Attrition /Measurement	Reference
Sub-Saharan Africa	Mali	Low SDI	Sidibe 2008	2001–2002	Retrospective cohort study	NR	2000	Low/Low/Moderate	(238)
Sub-Saharan Africa	Rwanda	Low SDI	Sievers 2008	2005–2006	Before and after intervention study (includes vaccine implementation studies)	Range: < 1years - 5years	322	Low/Low/Moderate	(239)
Sub-Saharan Africa	Mozambique	Low SDI	Sigauque 2009 (Pediatric Infect Dis J)	2001–2006	Surveillance study	Median (IQR): 13 months (6–25)	19896	Moderate/Low/Moderate	(240)
Sub-Saharan Africa	Mozambique	Low SDI	Sigauque 2009 (J Trop Pediatric)	2004–2006	Prospective cohort study	Range: 0–23 months	4838	Low/Low/Low	(241)
Sub-Saharan Africa	Mozambique	Low SDI	Sigauque 2018	2001–2012	Surveillance study	Range: 0 days–5 years	41106	Moderate/Moderate/Low	(242)
Sub-Saharan Africa	Kenya	Low-middle SDI	Silaba 2019	2014–2014	Surveillance study	Range: 2–143 months	1497	Low/Low/Moderate	(243)
Sub-Saharan Africa	Tanzania	Low SDI	Smart 2016	2011–2012	Prospective cohort study	Range: 3 months–12 years	1492	High/Moderate/Low	(244)
Sub-Saharan Africa	Senegal	Low SDI	Sylla 2015	2012–2012	Retrospective cohort study	Range: 0–59 months	393	Low/Low/Low	(245)
Sub-Saharan Africa	Ghana	Low-middle SDI	Tette 2016	2013–2013	Retrospective cohort study	Range: 1 day–9 years	4727	High/Moderate/High	(246)
Sub-Saharan Africa	Zambia	Low-middle SDI	Theo 2018	2011–2014	Surveillance study	Range: 2 days–5 years	49435	Low/Low/Low	(247)
Sub-Saharan Africa	Kenya	Low-middle SDI	Tornheim 2010	2001–2003	Retrospective cohort study	Range: 0–5 years	4814	Low/Low/Low	(248)
Sub-Saharan Africa	Liberia	Low SDI	Tsai 2017	2013–2013	Retrospective cohort study	Range: < 1month -5 years	920	Low/Low/High	(249)
Sub-Saharan Africa	Nigeria	Low-middle SDI	Ugege 2021	2018–2018	Prospective cohort study	Range: 1–156 months	376	Low/Low/High	(250)
Sub-Saharan Africa	Nigeria	Low-middle SDI	Ugwu 2014	2010–2012	Retrospective cohort study	Range: >28days-10 years	6875	Moderate/Low/Moderate	(251)
Sub-Saharan Africa	Malawi	Low SDI	Vonasek 2020	2018–2019	Prospective cohort study	Range: 6–36 months	6752	Low/Low/Low	(252)

Global Burden of Disease Super Region	Country	Socio-Demographic Index (SDI) Quintile	Study Identification	Study Period	Study Design	Age	Denominator	Risk of Bias for Participants/Attrition /Measurement	Reference
Sub-Saharan Africa	Sudan	Low-middle SDI	Zeidan 2006	2000–2000	Cross-sectional	Range: 0 days- 15 years	20944	Moderate/Moderate/Moderate	(253)

References

1. Thompson MG, Levine MZ, Bino S, Hunt DR, Al-Sanouri TM, Simões EAF, et al. Underdetection of laboratory-confirmed influenza-associated hospital admissions among infants: a multicentre, prospective study. Lancet Child Adolesc Health. 2019;3(11):781-94.
2. Araujo EMN, Costa GMC, Pedraza DF. Hospitalizations due to primary care-sensitive conditions among children under five years of age: cross-sectional study. Sao Paulo Med J. 2017;135(3):270-6.
3. Berezin EN, Jarovsky D, Cardoso MRA, Mantese OC. Invasive pneumococcal disease among hospitalized children in Brazil before and after the introduction of a pneumococcal conjugate vaccine. Vaccine. 2020;38(7):1740-5.
4. Carvalho SC, Mota E, Dourado I, Aquino R, Teles C, Medina MG. Hospitalizations of children due to primary health care sensitive conditions in Pernambuco State, Northeast Brazil. 2015;1(4):744-54.
5. Chúa C. ¿De qué se hospitalizan a los niños en Guatemala? Rev Col Méd Cir Guatem. 2014;151:44-5.
6. Rocha MC, Carminate DL, Tibiriçá SH, Carvalho IP, Silva ML, Chebli JM. Acute diarrhea in hospitalized children of the municipality of Juiz de Fora, MG, Brazil: prevalence and risk factors associated with disease severity. Arq Gastroenterol. 2012;49(4):259-65.
7. de Oliveira RR, da Costa JR, Mathias TAF. Hospitalization of children under five years of age due to avoidable causes. Revista Latino-Americana de Enfermagem. 2012;20(1):135-42.
8. Díaz-Garrido D, Pinto-Zaldumbide SC, Lazo-Álvarez MÁ, Cárdenas-Vivero AS, Valencia-Calderón CJ, Vásquez-Hahn C, Arbeláez-Rodríguez GDR. Causes of death in a pediatric tertiary care hospital in Ecuador. Revista Mexicana de Pediatria. 2018;85(6):207-11.
9. González RG, Granja AP, Caisaguano AT, Tibanta DL, Jiménez EB, Ortiz AG, et al. Incidence and clinical characteristics of children with community acquired pneumonia attending the hospital pediátrico “Baca Ortiz”, Ecuador. Archivos Venezolanos de Farmacologia y Terapeutica. 2020;39(4):260-3.
10. Gouvea VS, Dias GS, Aguiar EA, Pedro AR, Fichman ER, Chinem ES, et al. Acute gastroenteritis in a pediatric hospital in rio de janeiro in pre- and post-rotavirus vaccination settings. Open Virol J. 2009;3:26-30.
11. Jacomin V, Cruz Shibukawa BM, Ieda Harumi H. INFANT HOSPITALIZATION BY PRIMARY CARE'S SENSITIVE CONDITIONS IN A SOUTHERN BRAZILIAN STATE. Revista de Pesquisa: Cuidado e Fundamental. 2020;12(1):958-64.
12. Mangia CM, Kissoon N, Branchini OA, Andrade MC, Kopelman BI, Carcillo J. Bacterial sepsis in Brazilian children: a trend analysis from 1992 to 2006. PloS one. 2011;6(6):e14817.
13. Mansilla Flower P. Características clínico-epidemiológicas de pacientes con diagnóstico de Síndrome Urémico Hemolítico. Rev peru pediatr. 2012;65(3):29-.

14. Mariano TDSO, Nedel FB. Hospitalization for Ambulatory Care Sensitive Conditions in children under five years old in Santa Catarina State, Brazil, 2012: a descriptive study. Epidemiol Serv Saude. 2018;27(3):e2017322.
15. McCarthy JE, Evans-Gilbert T. Descriptive epidemiology of mortality and morbidity of health-indicator diseases in hospitalized children from western Jamaica. The American journal of tropical medicine and hygiene. 2009;80(4):596-600.
16. Noyola DE, Zuviri-González A, Castro-García JA, Ochoa-Zavala JR. Impact of respiratory syncytial virus on hospital admissions in children younger than 3 years of age. The Journal of infection. 2007;54(2):180-4.
17. Orrett FA, Changoor E, Maharaj N. Pediatric drug prescribing in a regional hospital in Trinidad. Journal of Chinese Clinical Medicine. 2010;5(3):157-63.
18. Paulo RL, Rodrigues AB, Machado BM, Gilio AE. The impact of rotavirus vaccination on emergency department visits and hospital admissions for acute diarrhea in children under 5 years. Rev Assoc Med Bras (1992). 2016;62(6):506-12.
19. Risquez A, Urbina-Medina H, Ponce A. Indicadores Hospitalarios 2011-2012: Hospital de niños JM de los Ríos de Caracas, Venezuela. Arch venez pueric pediatr. 2014;77(4):162-9.
20. Santos CAD, Rosa CDOB, Franceschini SDCC, Firmino HH, Ribeiro AQ. Usefulness of the StrongKids Screening Tool in Detecting Anemia and Inflammation in Hospitalized Pediatric Patients. Journal of the American College of Nutrition. 2021;40(2):155-63.
21. Sarni RO, Carvalho Mde F, Monte CM, Albuquerque ZP, Souza FI. Anthropometric evaluation, risk factors for malnutrition, and nutritional therapy for children in teaching hospitals in Brazil. Jornal de pediatria. 2009;85(3):223-8.
22. Vinekar K, Schaad N, Ber Lucien MA, Leshem E, Oboho IK, Joseph G, et al. Hospitalizations and Deaths Because of Respiratory and Diarrheal Diseases Among Haitian Children Under Five Years of Age, 2011-2013. The Pediatric infectious disease journal. 2015;34(10):e238-43.
23. Al Kubati AKAS. Non-induced traumatic coma in children in northern region of Yemen causes and mortality. Journal Medical Libanais. 2018;66(3):154-60.
24. Al-Taiar A, Jaffar S, Assabri A, Al-Habori M, Azazy A, Al-Mahdi N, et al. Severe malaria in children in Yemen: two site observational study. BMJ (Clinical research ed). 2006;333(7573):827.
25. Ali SH, Hussien FS, Abd Al-Amer H. Profile of renal diseases in Iraqi children: A single-center report. Saudi J Kidney Dis Transpl. 2015;26(3):613-8.
26. Al-Janabi HA, Raffas HA, Jassim SA. Incidence rate of mortality form massive pulmonary embolism in al-diwaniyah province, iraq, during the period from january 2011 through december 2018. Indian Journal of Forensic Medicine and Toxicology. 2019;13(3):330-4.
27. El Mhamdi S, Herizi C, Sriha A, Bouanene I, Ben Salah A, Gaaliche S, Soltani MS. [Profile and trends of pediatric hospital morbidity in the region of Monastir (Tunisia) for a decade]. Rev Med Brux. 2015;36(5):410-4.
28. Maalej B, Ben Amor M, Jallouli M, Weli M, Charfi M, Abdelkefi Y, et al. [Post-streptococcal glomerulonephritis in the south of Tunisia: A 12-year retrospective review]. Nephrol Ther. 2018;14(7):518-22.
29. Sallam AK. Common causes of child mortality in Sana'a, Yemen. Saudi Med J. 2005;26(7):1112-5.
30. Adhikari S, Sathian B, Koirala DP, Rao KS. Profile of children admitted with seizures in a tertiary care hospital of Western Nepal. BMC pediatrics. 2013;13:43.
31. Ashraf M, Kumar V, Bano RA, Wani KA, Ahmed J, Ahmed K. Spectrum of Renal and Urinary Tract Diseases in Kashmiri Children. Journal of clinical and diagnostic research : JCDR. 2016;10(6):SM01-2.

32. Badhan A, Bhardwaj P, Yadav V, Grover N, Seem RK. Demographic profile of pediatric malignancies in Himachal Pradesh. Indian Journal of Medical and Paediatric Oncology. 2018;39(3):287-91.
33. Basu M, Kundu TK, Dasgupta MK, Das DK, Saha I. Poisoning, stings and bites in children-- what is new? An experience from a tertiary care hospital in Kolkata. Indian J Public Health. 2009;53(4):229-31.
34. Champatiray J, Satapathy J, Kashyap B, Mondal D. Clinico-Aetiological Study of Severe and Very Severe Pneumonia in Two Months to Five Years Children in a Tertiary Health Care Centre in Odisha, India. Journal of clinical and diagnostic research : JCDR. 2017;11(9):SC06-SC10.
35. Chapagain RH, Agrawal S, Pokharel S, Kayastha M, Bhattrai S, Jha A, et al. Clinico-Laboratory Profile, Complications and Therapeutic Outcome of Scrub Typhus in Children. J Nepal Health Res Counc. 2020;18(2):282-7.
36. Chaudhary N, Gupta MM, Shrestha S, Pathak S, Kurmi OP, Bhatia BD, Agarwal KN. Clinicodemographic Profile of Children with Seizures in a Tertiary Care Hospital: A Cross-Sectional Observational Study. Neurol Res Int. 2017;2017:1524548.
37. Drolia A, Dewan P, Gupta P. Predicting the severity of bronchiolitis in a resource-poor setting. Internet Journal of Pediatrics and Neonatology. 2010;11(1).
38. Duwarah SG, Hazarika RD, Barman H, Deka P. Profile of hypertension in children: Experience from a tertiary care institute in North East India. Indian Journal of Medical Specialities. 2016;7(3):100-2.
39. Ganjoo S, Ahmad K, Qureshi UA, Mir ZH. Clinical Epidemiology of SIRS and Sepsis in Newly Admitted Children. Indian journal of pediatrics. 2015;82(8):698-702.
40. Garg P. Pediatric hospitalizations at two different setting community hospitals in north India: implications for regionalization of care. Indian journal of pediatrics. 2009;76(7):711-6.
41. Giri BR, Chapagain RH, Sharma S, Shrestha S, Ghimire S, Shankar PR. Effect of the 2015 earthquake on pediatric inpatient pattern at a tertiary care hospital in Nepal. BMC pediatrics. 2018;18(1):28.
42. Gupta SK, Sarmah BK, Tiwari D, Thapa S. Pattern of Pediatric Admissions in a Tertiary Care Hospital of Central Nepal. JNMA J Nepal Med Assoc. 2015;53(198):118-22.
43. Gupta MM, Chaudhary N, Pathak S, Agrawal N, Yadav J, Shrestha S, et al. Neurocysticercosis in Children with Seizures: A Cross-Sectional Study. Int J Pediatr. 2018;2018:1030878.
44. Gupta PK, Singhi P, Singhi S, Kasinathan A, Sankhyan N. How Different is AMAN from AIDP in Childhood GBS? A Prospective Study from North India. Indian journal of pediatrics. 2019;86(4):329-34.
45. Halder R, Malik R, Aggarwal KC, Nair D, Sharma S. Clinical Profile of Bacterial Meningitis in Children and Comparative Inter-Alia Analysis of Various Microbiological Tests. Journal of Child Science. 2020;10(1):E38-e44.
46. Jose A, Sivanandam S, Matthai J. Poisoning in children from an educationally and economically advanced urban area of South India. Asian Journal of Epidemiology. 2012;5(4):123-9.
47. Mahajan V, Kaur A, Sharma A, Azad C, Guglani V. Modifiable factors for prevention of childhood mortality. 2014;1(1):45-7.
48. Malla T, Malla KK, Rao KS, Gauchan E, Basnet S, Koirala DP. A scenario of poisoning in children in Manipal teaching hospital. Journal of Nepal Paediatric Society. 2011;31:83-8.
49. Masood-Us-Syed SS, Basit AK, Mohammad S, Iqbal Ghumman MZ, Sattar A, Ammad Ul I, et al. Screening of childhood tuberculosis with Pakistan pediatric association scoring chart system. Pakistan Paediatric Journal. 2012;36(4):220-4.

50. Mathur A, Tahilramani G, Makhija S, Devgan V. Burden of Severe Acute Malnutrition in under-five Children (2-59 Months) Admitted in a Tertiary Care Hospital of Delhi. Journal of tropical pediatrics. 2018;64(1):45-50.
51. Mishra S, Ramkumar TV, Biswas AK, Panigrahi S. Childhood poisoning, a rising epidemic in developing nations: Large single centre study. Journal of Nepal Paediatric Society. 2017;37(2):117-21.
52. Naheed A, Saha SK, Breiman RF, Khatun F, Brooks WA, El Arifeen S, et al. Multihospital surveillance of pneumonia burden among children aged <5 years hospitalized for pneumonia in Bangladesh. Clinical infectious diseases : an official publication of the Infectious Diseases Society of America. 2009;48 Suppl 2:S82-9.
53. Roy RN, Shrivastava P, Das DK, Saha I, Sarkar AP. Burden of hospitalized pediatric morbidity and utilization of beds in a tertiary care hospital of kolkata, India. Indian J Community Med. 2012;37(4):252-5.
54. Rajak K, Twayana AR, Shrestha R, Amatya P, Ghimire C. Prevalence of kawasaki disease in a tertiary care hospital: A descriptive cross-sectional study. Journal of the Nepal Medical Association. 2019;57(220):416-9.
55. Rao S, Gavali V, Prabhu SS, Mathur R, Dabre LR, Prabhu SB, Bodhanwala M. Outcome of Children Admitted With SARS-CoV-2 Infection: Experiences From a Pediatric Public Hospital. Indian pediatrics. 2021;58(4):358-62.
56. Rasheed J, Wakeel N, Aleem T, Khalid M, Zafar F. Pattern and outcome of pediatric admissions in a tertiary care hospital Multan. Pakistan Paediatric Journal. 2017;41(3):168-73.
57. Rasul CH, Muhammad F, Hossain MJ, Ahmed KU, Rahman M. Acute meningoencephalitis in hospitalised children in southern Bangladesh. Malays J Med Sci. 2012;19(2):67-73.
58. Roy MP, Gupta R, Bhatt M, Aggarwal KC. Causes of Death among Children Aged >5 Years in a Public Hospital in New Delhi. Indian pediatrics. 2017;54(1):60-1.
59. Sadiq MW, Ukrani RD, Arif A, Akbar I, Altaf S, Moiz B. Risk Assessment and Outcome of Venous Thromboembolism in Pediatric Population in an Academic Care Center of a Low-Middle Income Country. Clinical and applied thrombosis/hemostasis : official journal of the International Academy of Clinical and Applied Thrombosis/Hemostasis. 2021;27:1076029621995895.
60. Sarangi R, Pattnaik L, Satpathy SK, Sahu MC. Mortality pattern of under-five children – A hospital-based cross-sectional study in a tertiary care hospital of India. Asian Journal of Pharmaceutical and Clinical Research. 2017;10(9):82-4.
61. Sharma P, Sarmah BK, Kayastha P, Shrestha A, Tiwari D. Clinical profile of children with acute febrile encephalopathy in a tertiary health care center of Nepal. Journal of Nepal Paediatric Society. 2015;35(3):224-30.
62. Sharma M, Damlin A, Pathak A, Lundborg CS. Antibiotic prescribing among pediatric inpatients with potential infections in two private sector hospitals in Central India. PloS one. 2015;10(11).
63. Shrestha S, Bichha RP, Sharma A, Upadhyay S, Rijal P. Clinical profile of tuberculosis in children. Nepal Med Coll J. 2011;13(2):119-22.
64. Shrestha D, Regmi D, Raya G, Prajapati A, Dhaubhadel S, Puri S. Radiographically Confirmed Community-Acquired Pneumonia in Pediatric Patients Prior to Pneumococcal Vaccination in Nepal. Journal of Pediatric Infectious Diseases. 2018;13(1):57-62.
65. Siddique AK, Ahmed S, Iqbal A, Sobhan A, Poddar G, Azim T, et al. Epidemiology of rotavirus and cholera in children aged less than five years in rural Bangladesh. J Health Popul Nutr. 2011;29(1):1-8.
66. Sil A, Ghosh TN, Bhattacharya S, Konar MC, Soren B, Nayek K. A study on clinico-epidemiological profile of poisoning in children in a rural tertiary care hospital. Journal of Nepal Paediatric Society. 2016;36(2):105-9.
67. Singh D, Chopra A, Pooni PA, Bhatia RC. A clinical profile of shock in children in Punjab, India. Indian pediatrics. 2006;43(7):619-23.

68. Sonowal R. Profile of renal diseases in North-East Indian children. Saudi J Kidney Dis Transpl. 2019;30(5):1151-5.
69. van Deursen B, Lenglet A, Ariti C, Hussain B, Karsten J, Roggeveen H, et al. Risks and seasonal pattern for mortality among hospitalized infants in a conflict-affected area of Pakistan, 2013-2016. A retrospective chart review. F1000Res. 2019;8:954.
70. Verma S, Rai SK, Kant S, Choudhury K. Morbidity profile of paediatric inpatents at a community health centre and a nearby [correction of near by] district hospital in northern India. Indian J Public Health. 2007;51(2):125-6.
71. Zaheer A, Rashid A, Chishty AL. Neurological disease spectrum and associated factors for morbidity and mortality among admitted children. Pakistan Paediatric Journal. 2009;33(1):24-9.
72. Barennes H, Sayavong E, Pussard E. High Mortality Risk in Hypoglycemic and Dysglycemic Children Admitted at a Referral Hospital in a Non Malaria Tropical Setting of a Low Income Country. PloS one. 2016;11(2):e0150076.
73. Bucens IK, Maclennan C. Survey of childhood malnutrition at Dili National Hospital, East Timor. Journal of paediatrics and child health. 2006;42(1-2):28-32.
74. Bucens IK, Reid A, Barreto AC, Dwivedi V, Counahan M. Three years of paediatric morbidity and mortality at the National Hospital in Dili, East Timor. Journal of paediatrics and child health. 2013;49(12):1004-9.
75. Chusilp K, Kosuwon P, Panthongwiriyakul C, Thepsuthammarat K, Wangsai S, Sutra S. Thai infant health situation: essential medical information for family centered care. J Med Assoc Thai. 2012;95 Suppl 7:S24-9.
76. Duke T, Yano E, Hutchinson A, Hwaihwanje I, Aipit J, Tovilu M, et al. Large-scale data reporting of paediatric morbidity and mortality in developing countries: It can be done. Archives of Disease in Childhood. 2016;101:392-7.
77. Ho NT, Thompson C, Nhan LNT, Van HMT, Dung NT, Tran My P, et al. Retrospective analysis assessing the spatial and temporal distribution of paediatric acute respiratory tract infections in Ho Chi Minh City, Vietnam. BMJ open. 2018;8(1):e016349.
78. Jetsrisuparb A, Teeratakulpisarn J, Weraarchakul W, Thepsuthammarat K, Sutra S. Health situation analysis of Thai children aged 1-5 years in 2010: implications for health education and health service reform. J Med Assoc Thai. 2012;95 Suppl 7:S30-42.
79. Kudagammana ST, Karunaratne RR, Munasinghe TS, Kudagammana HDWS. Community acquired paediatric pneumonia; experience from a pneumococcal vaccine- naive population. Pneumonia (Nathan Qld). 2020;12:8.
80. Laman M, Aipit S, Bona C, Aipit J, Davis TME, Manning L. Contribution of Malaria to Inhospital Mortality in Papua New Guinean Children from a Malaria-Endemic Area: A Prospective Observational Study. The American journal of tropical medicine and hygiene. 2019;100(4):835-41.
81. Langridge FC, Hufanga SV, ‘Ofanoa MM, Fakakovikaetau T, Percival TM, Grant CC, Ofanoa MM. Child morbidity as described by hospital admissions for primary school aged children in Tonga 2009-2013. New Zealand Medical Journal. 2017;130(1465):29-43.
82. Moe K, Hummelman EG, Oo WM, Lwin T, Htwe TT. Hospital-based surveillance for rotavirus diarrhea in children in Yangon, Myanmar. Journal of Infectious Diseases. 2005;192(SUPPL. 1):S111-S3.
83. Murni IK, Duke T, Kinney S, Daley AJ, Laksanawati IS, Nurnaningsih, et al. Multifaceted interventions for healthcare-associated infections and rational use of antibiotics in a low-to-middle-income country: Can they be sustained? PloS one. 2020;15(6 June).
84. Nguyen TKP, Nguyen DV, Truong TNH, Tran MD, Graham SM, Marais BJ. Disease spectrum and management of children admitted with acute respiratory infection in Viet Nam. Tropical medicine & international health : TM & IH. 2017;22(6):688-95.
85. Nguyen NTT, Dien TM, Schindler C, Lien NTB, Probst-Hensch N, Lan VTH, et al. Childhood hospitalisation and related deaths in Hanoi, Vietnam: a tertiary hospital database analysis from 2007 to 2014. BMJ open. 2017;7(7):e015260.

86. Pham TD, Hoang VT, Dao TL, Tran XD, Phi DL, To MM, et al. Morbidity and Mortality Patterns in Children Admitted to Hospital in Thai Binh, Vietnam: A Five-year Descriptive Study with a Focus on Infectious Diseases. J Epidemiol Glob Health. 2020;29:29.
87. Poespoprodjo JR, Fobia W, Kenangalem E, Lampah DA, Hasanuddin A, Warikar N, et al. Vivax malaria: a major cause of morbidity in early infancy. Clinical infectious diseases : an official publication of the Infectious Diseases Society of America. 2009;48(12):1704-12.
88. Rero A, Aipit J, Yarong-Kote T, Watch V, Bolnga JW, Vei R, et al. The Burden of Child Maltreatment Leading to Hospitalization in a Provincial Setting in Papua New Guinea. Journal of tropical pediatrics. 2016;62(4):282-7.
89. Suphakunpinyo C, Areemit R, Thepsuthammarat K, Sutra S. The health situation among Thai elementary school-age children: 2010. J Med Assoc Thai. 2012;95 Suppl 7:S43-50.
90. Tan KK, Dang DA, Kim KH, Kartasasmita C, Kim HM, Zhang XH, et al. Burden of hospitalized childhood community-acquired pneumonia: A retrospective cross-sectional study in Vietnam, Malaysia, Indonesia and the Republic of Korea. Hum Vaccin Immunother. 2018;14(1):95-105.
91. Thompson CN, Zelner JL, Nhu TDH, Phan MVT, Hoang Le P, Nguyen Thanh H, et al. The impact of environmental and climatic variation on the spatiotemporal trends of hospitalized pediatric diarrhea in Ho Chi Minh City, Vietnam. Health and Place. 2015;35:147-54.
92. Wandi F, Peel D, Duke T. Hypoxaemia among children in rural hospitals in Papua New Guinea: epidemiology and resource availability--a study to support a national oxygen programme. Annals of tropical paediatrics. 2006;26(4):277-84.
93. Wilopo SA, Soenarto Y, Bresee JS, Tholib A, Aminah S, Cahyono A, et al. Rotavirus surveillance to determine disease burden and epidemiology in Java, Indonesia, August 2001 through April 2004. Vaccine. 2009;27(SUPPL. 5):F61-F6.
94. Abebe Gm. A two year retrospective review of reasons for pediatric admission to Chiro Hospital, Eastern Ethiopia. Ethiop Med J. 2005;43(4):241-9.
95. Adadey SM, Ayee R, Languon S, Quansah D, Quaye O. Patterns of Frequently Diagnosed Pediatric Morbidities in Hospitalized Children in the Volta Region of Ghana. Glob. 2019;6:2333794X19889230.
96. Adeboye MA, Ojuawo A, Ernest SK, Fadeyi A, Salisu OT. Mortality pattern within twenty-four hours of emergency paediatric admission in a resource-poor nation health facility. West Afr J Med. 2010;29(4):249-52.
97. Adeboye M, Adesiyun O, Adegboye A, Eze E, Abubakar U, Ahmed G, et al. Measles in a tertiary institution in bida, niger state, Nigeria: prevalence, immunization status and mortality pattern. Oman med. 2011;26(2):114-7.
98. Adeboye M, Ojuawo A, Adeniyi A, Ibraheem RM, Amiwero C. Febrile Convulsion among Hospitalized Children Aged Six Months to Five Years and Its Association With Haemoglobin Electrophoretic Pattern. Ethiopian journal of health sciences. 2015;25(3):251-6.
99. Adegoke SA, Dedeke IO, Oyelami OA. Childhood injuries in Ilesa, South-Western Nigeria: causes, pattern, and outcome. West Afr J Med. 2010;29(4):253-8.
100. Adegoke S, Ayansanwo A, Oluwayemi I, Okeniyi J. Determinants of mortality in Nigerian children with severe anaemia. Samj, S. 2012;102(10):807-10.
101. Adekanmbi AF, Ogunlesi TA, Olowu AO, Fetuga MB. Current trends in the prevalence and aetiology of childhood congestive cardiac failure in Sagamu. Journal of tropical pediatrics. 2007;53(2):103-6.
102. Adeleke SI, Asani MO, Belonwu RO, Gwarzo GD, Farouk ZL. Childhood diabetes mellitus in Kano, North West, Nigeria. Niger J Med. 2010;19(2):145-7.

103. Adem F, Edessa D, Bayissa B, Hassen MM, Mohammed MA. Treatment outcomes and associated factors in hospitalised children with severe acute malnutrition: A prospective cohort study. Pediatric Health, Medicine and Therapeutics. 2020;11:235-43.
104. Ademola AD, Asinobi AO, Ekpe-Adewuyi E, Ayede AI, Ajayi SO, Raji YR, et al. Acute kidney injury among paediatric emergency room admissions in a tertiary hospital in South West Nigeria: a cohort study. Clin Kidney J. 2019;12(4):521-6.
105. Adeyeye EI, Adanlawo IG. Analysis of medical admissions into children's ward of the Ekiti state specialist hospital, Ado-Ekiti, Nigeria, 2000-2001. Biosciences Biotechnology Research Asia. 2007;4(1):123-34.
106. Agbeille MF, Adedemy JD, Noudamadjo A, Kpanidja, Mbanga-Ngoume JJ, Agossou J. Cerebral malaria in child in Departmental University Teaching Hospital of Borgou, Benin. Medecine d'Afrique Noire. 2019;66(3):131-8.
107. Ahmed PA, Babaniyi IB, Otuneye AT. Review of childhood measles admissions at the National Hospital, Abuja. Nigerian journal of clinical practice. 2010;13(4):413-6.
108. Ahmed M, Weddih A, Benhafid M, Bollahi MA, Sidatt M, Makhalla K, et al. Hospitalizations and Deaths Associated with Diarrhea and Respiratory Diseases among Children Aged 0-5 Years in a Referral Hospital of Mauritania. Trop. 2018;3(3):17.
109. Ahmed MC, Heukelbach J, Weddih A, Filali-Maltouf A, Sidatt M, Makhalla K, et al. Reduction of hospitalizations with diarrhea among children aged 0-5years in Nouakchott, Mauritania, following the introduction of rotavirus vaccine. Vaccine. 2019;37(11):1407-11.
110. Akech S, Chepkirui M, Ogero M, Agweyu A, Irimu G, English M, Snow RW. The Clinical Profile of Severe Pediatric Malaria in an Area Targeted for Routine RTS,S/AS01 Malaria Vaccination in Western Kenya. Clinical infectious diseases : an official publication of the Infectious Diseases Society of America. 2020;71(2):372-80.
111. Akinbami FO, Hamzat TH, Orimadegun AE, Tongo O, Oyeyemi L, Okafor O, Akinyinka OO. Body mass composition: a predictor of admission outcomes among hospitalized Nigerian under 5 children. Asia Pac J Clin Nutr. 2010;19(3):295-300.
112. Aloni MN, Nsibu CN, Meeko-Mimaniye M, Ekulu PM, Bodi JM. Acute renal failure in Congolese children: a tertiary institution experience. Acta Paediatrica. 2012;101(11):e514-8.
113. Amare AT, Kebede ZT, Welch HD. Epidemiology of bacterial meningitis in children admitted to Gondar University Hospital in the post pneumococcal vaccine era. Pan Afr Med J. 2018;31:193.
114. Andersen A, Bjerregaard-Andersen M, Rodrigues A, Umbasse P, Fisker AB. Sex-differential effects of diphtheria-tetanus-pertussis vaccine for the outcome of paediatric admissions? A hospital based observational study from Guinea-Bissau. Vaccine. 2017;35(50):7018-25.
115. Animasahun A, Itiola J, Falase B, Gbelee O, Kehinde O, Odusanya O, et al. Congestive cardiac failure among Nigerian children; pattern and outcome. International Cardiovascular Research Journal. 2015;9(3):164-8.
116. Ayieko P, Ogero M, Makone B, Julius T, Mbevi G, Nyachiro W, et al. Characteristics of admissions and variations in the use of basic investigations, treatments and outcomes in Kenyan hospitals within a new Clinical Information Network. Archives of Disease in Childhood. 2016;101(3):223-9.
117. Bassat Q, Guinovart C, Sigauque B, Aide P, Sacarlal J, Nhampossa T, et al. Malaria in rural Mozambique. Part II: children admitted to hospital. Malar J. 2008;7:37.
118. Belonwu RO, Adeleke SI. A seven-year review of accidental kerosene poisoning in children at Aminu Kano Teaching Hospital, Kano. Niger J Med. 2008;17(4):380-2.

119. Berti A, Bregani ER, Manenti F, Pizzi C. Outcome of severely malnourished children treated according to UNICEF 2004 guidelines: a one-year experience in a zone hospital in rural Ethiopia. Transactions of the Royal Society of Tropical Medicine and Hygiene. 2008;102(9):939-44.
120. Birindwa AM, Manegabe JT, Mindja A, Norden R, Andersson R, Skovbjerg S. Decreased number of hospitalized children with severe acute lower respiratory infection after introduction of the pneumococcal conjugate vaccine in the Eastern Democratic Republic of the Congo. Pan Afr Med J. 2020;37:211.
121. Bohn JA, Kassaye BM, Record D, Chou BC, Kraft IL, Purdy JC, et al. Demographic and mortality analysis of hospitalized children at a referral hospital in Addis Ababa, Ethiopia. BMC pediatrics. 2016;16(1):168.
122. Bouyou-Akotet MK, Mawili-Mboumba DP, Kendjo E, Eyang Ekouma A, Abdou Raouf O, Engohang Allogho E, Kombila M. Complicated malaria and other severe febrile illness in a pediatric ward in Libreville, Gabon. BMC Infect Dis. 2012;12:216.
123. Boyle KL, Periyanayagam U, Babu KM, Rice BT, Bisanzo M. Pediatric Poisonings in a Rural Ugandan Emergency Department. Pediatric emergency care. 2017;09:09.
124. Boyle KL, Periyanayagam U, Babu KM, Rice BT, Bisanzo M. Pediatric Poisonings in a Rural Ugandan Emergency Department. Pediatric emergency care. 2020;36(3):e160-e2.
125. Brits H, Botha L, Maakomane W, Malefane T, Luthfiya T, Tsoeueamakwa T, Joubert G. The profile and clinical picture of children with undernutrition admitted to National District Hospital. Pan Afr Med J. 2020;37:237.
126. Brugnolaro V, Fovino LN, Calgaro S, Putoto G, Muhelo AR, Gregori D, et al. Pediatric emergency care in a low-income country: Characteristics and outcomes of presentations to a tertiary-care emergency department in Mozambique. PloS one. 2020;15(11):e0241209.
127. Chami N, Hau DK, Masoza TS, Smart LR, Kayange NM, Hokororo A, et al. Very severe anemia and one year mortality outcome after hospitalization in Tanzanian children: A prospective cohort study. PloS one. 2019;14(6):e0214563.
128. Charles NC, Chuku A, Anazodo NM. Childhood mortality in federal medical centre umuahia, South eastern Nigeria. Oman med. 2014;29(5):320-4.
129. Chelo D, Mekone Nkwelle I, Nguefack F, Mbassi Awa HD, Enyama D, Nguefack S, et al. Decrease in Hospitalizations and Increase in Deaths during the Covid-19 Epidemic in a Pediatric Hospital, Yaounde-Cameroon and Prediction for the Coming Months. Fetal Pediat Pathol. 2020:1-14.
130. Chiabi A, Takou V, Tchokoteu PF, Um SN, Essoh L, Immumboeh P. Initial treatment of severe malaria in children is inadequate - A study from a referral hospital in Cameroon. SAJCH South African Journal of Child Health. 2009;3(1):9-11.
131. Chiabi A, Malangue B, Nguefack S, Dongmo FN, Fru F, Takou V, Angwafo F, 3rd. The clinical spectrum of severe acute malnutrition in children in Cameroon: a hospital-based study in Yaounde, Cameroon. Transl. 2017;6(1):32-9.
132. Chiabi A, Djimafo ANM, Nguefack S, Mah E, Nguefack Dongmo F, Angwafo F, 3rd. Severe malaria in Cameroon: Pattern of disease in children at the Yaounde Gynaeco-Obstetric and Pediatric hospital. J Infect Public Health. 2020;13(10):1469-72.
133. Couto TB, Farhat SCL, Reid T, Schvartsman C. Mortalidade em hospital secundário pediátrico na Libéria pós-conflito em 2009. Einstein (Säo Paulo). 2013;11(4):413-20.
134. Edelu BO, Ndu IK, Igbokwe OO, Iloh ON. Severe falciparum malaria in children in Enugu, South East Nigeria. Nigerian journal of clinical practice. 2018;21(10):1349-55.

135. Ekaru H, Mbarak N, Shurie S, Kosgei E, Oyungu E, Kwena A. Community Acquired Pneumonia among Children Admitted in a Tertiary Hospital: The Burden and Related Factors. East African medical journal. 2012;89(9):301-5.
136. Ekenze SO, Ekwunife H, Eze BI, Ikefuna A, Amah CC, Emodi IJ. The burden of pediatric malignant solid tumors in a developing country. Journal of tropical pediatrics. 2010;56(2):111-4.
137. Enyuma CO, Anah MU, Pousson A, Olorunfemi G, Ibisomi L, Abang BE, Imoke EJ. Patterns of paediatric emergency admissions and predictors of prolonged hospital stay at the children emergency room, University of Calabar Teaching Hospital, Calabar, Nigeria. African health sciences. 2019;19(2):1910-23.
138. Eseigbe EE, Adama SJ, Eseigbe P. Febrile seizures in Kaduna, north western Nigeria. Nigerian medical journal : journal of the Nigeria Medical Association. 2012;53(3):140-4.
139. Esezobor CI, Ladapo TA, Osinaike B, Lesi FE. Paediatric acute kidney injury in a tertiary hospital in Nigeria: prevalence, causes and mortality rate. PloS one. 2012;7(12):e51229.
140. Eshetie TC, Hailemeskel B, Mekonnen N, Paulos G, Mekonnen AB, Girma T. Adverse drug events in hospitalized children at Ethiopian University Hospital: a prospective observational study. BMC pediatrics. 2015;15:83.
141. Evans RDR, Docherty M, Seeley A, Craik A, Mpugna M, Mann S, et al. Incidence, Etiology, and Outcomes of Community-Acquired Acute Kidney Injury in Pediatric Admissions in Malawi. Perit Dial Int. 2018;38(6):405-12.
142. Fadero FF, Onigbinde MA, Oyedeji OA. Trends in childhood deaths in Eleta Hospital Ibadan, Oyo State, Southwestern Nigeria. International Journal of Tropical Medicine. 2012;7(5):173-6.
143. Forae GD, Uchendu OJ, Igbe AP. An audit of paediatric mortality patterns in a Nigerian teaching hospital. Nigerian medical journal : journal of the Nigeria Medical Association. 2014;55(2):130-3.
144. Gapu P, Bwakura-Dangarembizi M, Kandawasvika G, Kao D, Bannerman C, Hakim J, Matenga JA. Rheumatic fever and rheumatic heart disease among children presenting to two referral hospitals in Harare, Zimbabwe. Samj, S. 2015;105(5):384-8.
145. Garba BI, Muhammad AS, Obasi AB, Adeniji AO. Presentation and pattern of childhood renal diseases in Gusau, North-Western Nigeria. SAJCH South African Journal of Child Health. 2017;11(2):96-8.
146. Gardner A, Fraile K, Shirk A. Comparative mortality for children at one hospital in Kenya staffed with pediatric emergency medicine specialists. Afr J Emerg Med. 2020;10(4):224-8.
147. Gathara D, Malla L, Ayieko P, Karuri S, Nyamai R, Irimu G, et al. Variation in and risk factors for paediatric inpatient all-cause mortality in a low income setting: Data from an emerging clinical information network. BMC pediatrics. 2017;17(1).
148. Gebremariam S, Moges T. Pediatric Heart Failure, Lagging, and Sagging of Care in Low Income Settings: A Hospital Based Review of Cases in Ethiopia. Cardiol Res Pract. 2016;2016:7147234.
149. George IO, Alex-Hart BA, Frank-Briggs AI. Mortality pattern in children: a hospital based study in Nigeria. International journal of biomedical science : IJBS. 2009;5(4):369-72.
150. George IO, Tabansi PN. An audit of cases admitted in the children emergency ward in a nigerian tertiary hospital. Pakistan Journal of Medical Sciences. 2010;26(3):740-3.
151. Gordon DM, Frenning S, Draper HR, Kokeb M. Prevalence and burden of diseases presenting to a general pediatrics ward in Gondar, Ethiopia. Journal of tropical pediatrics. 2013;59(5):350-7.

152. Graham H, Bakare AA, Ayede AI, Oyewole OB, Gray A, Peel D, et al. Hypoxaemia in hospitalised children and neonates: A prospective cohort study in Nigerian secondary-level hospitals. EClinicalMedicine. 2019;16:51-63.
153. Graham H, Bakare AA, Ayede AI, Oyewole OB, Gray A, Neal E, et al. Diagnosis of pneumonia and malaria in Nigerian hospitals: A prospective cohort study. Pediatr Pulmonol. 2020;55 Suppl 1:S37-S50.
154. Gwer S, Thuo N, Idro R, Ndiritu M, Boga M, Newton C, Kirkham F. Changing trends in incidence and aetiology of childhood acute non-traumatic coma over a period of changing malaria transmission in rural coastal Kenya: a retrospective analysis. BMJ open. 2012;2(2):e000475.
155. Hammitt LL, Kazungu S, Morpeth SC, Gibson DG, Mvera B, Brent AJ, et al. A preliminary study of pneumonia etiology among hospitalized children in Kenya. Clinical infectious diseases : an official publication of the Infectious Diseases Society of America. 2012;54 Suppl 2:S190-9.
156. Harris C, Mills R, Seager E, Blackstock S, Hiwa T, Pumphrey J, et al. Paediatric deaths in a tertiary government hospital setting, Malawi. Paediatrics and international child health. 2019;39(4):240-8.
157. Hau DK, Chami N, Duncan A, Smart LR, Hokororo A, Kayange NM, Peck RN. Post-hospital mortality in children aged 2-12 years in Tanzania: A prospective cohort study. PloS one. 2018;13(8):e0202334.
158. Huerga H, Vasset B, Prados E. Adult and paediatric mortality patterns in a referral hospital in Liberia 1 year after the end of the war. Transactions of the Royal Society of Tropical Medicine and Hygiene. 2009;103(5):476-84.
159. Ibekwe RC, Ibekwe MU, Onwe OE, Nnebe-Agumadu UH, Ibe BC. Non-traumatic childhood coma in Ebonyi State University Teaching Hospital, Abakaliki, South Eastern Nigeria. Nigerian journal of clinical practice. 2011;14(1):43-6.
160. Idro R, Gwer S, Kahindi M, Gatakaa H, Kazungu T, Ndiritu M, et al. The incidence, aetiology and outcome of acute seizures in children admitted to a rural Kenyan district hospital. BMC pediatrics. 2008;8:5.
161. Isaacs-Long Y, Myer L, Zar HJ. Trends in admissions, morbidity and outcomes at Red Cross war memorial children's hospital, Cape Town, 2004 - 2013. South African Medical Journal. 2017;107(3):219-26.
162. John C, Abok II, Yilgwan C. Clinical profile of childhood type 1 diabetes in Jos, Nigeria. African Journal of Diabetes Medicine. 2013;21(1):11-3.
163. Karuri SW, Murithi MK, Irimu G, English M, Clinical Information Network a. Using data from a multi-hospital clinical network to explore prevalence of pediatric rickets in Kenya. Wellcome Open Res. 2017;2:64.
164. Kiggundu VL, O'Meara WP, Musoke R, Nalugoda FK, Kigozi G, Baghendaghe E, et al. High prevalence of malaria parasitemia and anemia among hospitalized children in Rakai, Uganda. PloS one. 2013;8(12):e82455.
165. Ku BC, Zonfrillo MR, Periyanayagam U, Bisanzo M, Rice B, Maling S, Cohn KA. The Association of Malnutrition and Disease Conditions in Mortality of Pediatric Patients Presenting to a Rural Emergency Department in Uganda. Pediatric emergency care. 2020;08:08.
166. Kuti BP, Adegoke SA, Ebruke BE, Howie S, Oyelami OA, Ota MOC. Risk factors for mortality in childhood pneumonia in a rural West African region. Journal of Pediatric Infectious Diseases. 2013;8(3):131-8.
167. Kuti BP, Adegoke SA, Oyelami OA, Ota MO. Predictors of prolonged hospitalisation in childhood pneumonia in a rural health centre. SAJCH South African Journal of Child Health. 2014;8(1):11-5.
168. Kuti BP, Bello EO, Jegede TO, Olubosede O. Epidemiological, clinical and prognostic profile of childhood acute bacterial meningitis in a resource poor setting. J Neurosci Rural Pract. 2015;6(4):549-57.

169. Lamorde M, Mpimbaza A, Walwema R, Kamya M, Kapisi J, Kajumbula H, et al. A Cross-Cutting Approach to Surveillance and Laboratory Capacity as a Platform to Improve Health Security in Uganda. Health Secur. 2018;16(S1):S76-S86.
170. Libwea JN, Kingue SRB, Ashukem NT, Kobela M, Boula A, Sinata KS, Ndombo PK. Assessing the causes of under-five mortality and proportion associated with pneumococcal diseases in Cameroon. A case-finding retrospective observational study: 2006–2012. PloS one. 2019;14(4).
171. Losimba Likwela J, D'Alessandro U, Donnen P, Wilmet Dramaix M. Clinical aspects and outcome of suspected severe pediatric malaria. Medecine et Maladies Infectieuses. 2012;42(7):315-20.
172. Lowlaavar N, Larson CP, Kumbakumba E, Zhou G, Ansermino JM, Singer J, et al. Pediatric in-Hospital Death from Infectious Disease in Uganda: Derivation of Clinical Prediction Models. PloS one. 2016;11(3):e0150683.
173. Lugangira K, Kazaura M, Kalokola F. Morbidity and mortality of children aged 2-59 months admitted in the Tanzania Lake Zone's public hospitals: a cross-sectional study. BMC research notes. 2017;10(1):502.
174. Lundgren IS, Heltshe SL, Smith AL, Chibwana J, Fried MW, Duffy PE. Bacteremia and malaria in Tanzanian children hospitalized for acute febrile illness. Journal of tropical pediatrics. 2015;61(2):81-5.
175. Ly F, Camara B, Ngomna F, Sakho Kane A, Sall Diouf A, Sow A, et al. Acute intoxications in the pediatric ward of the Pikine National Hospital Center (CHN): On 34 cases collected. Medecine d'Afrique Noire. 2019;66(4):200-8.
176. Macharia AW, Mochamah G, Uyoga S, Ndila CM, Nyutu G, Makale J, et al. The clinical epidemiology of sickle cell anemia In Africa. Am J Hematol. 2018;93(3):363-70.
177. Madrid L, Acacio S, Nhampossa T, Lanaspa M, Sitoe A, Maculuve SA, et al. Hypoglycemia and Risk Factors for Death in 13 Years of Pediatric Admissions in Mozambique. The American journal of tropical medicine and hygiene. 2016;94(1):218-26.
178. Mahgoub HM, Adam I. Morbidity and mortality of severe malnutrition among Sudanese children in New Halfa Hospital, Eastern Sudan. Transactions of the Royal Society of Tropical Medicine and Hygiene. 2012;106(1):66-8.
179. Maitland K, Berkley JA, Shebbe M, Peshu N, English M, Newton CR. Children with severe malnutrition: can those at highest risk of death be identified with the WHO protocol? PLoS Med. 2006;3(12):e500.
180. Maitland K, Ohuma EO, Mpoya A, Uyoga S, Hassall O, Williams TN. Informing thresholds for paediatric transfusion in Africa: the need for a trial. Wellcome Open Res. 2019;4:27.
181. McCollum ED, Bjornstad E, Preidis GA, Hosseinipour MC, Lufesi N. Multicenter study of hypoxemia prevalence and quality of oxygen treatment for hospitalized Malawian children. Transactions of the Royal Society of Tropical Medicine and Hygiene. 2013;107(5):285-92.
182. Mdala JF, Mash R. Causes of mortality and associated modifiable health care factors for children (< 5-years) admitted at Onandjokwe Hospital, Namibia. Afr. 2015;7(1):03.
183. Mhando S, Young B, Lakhoo K. The scope of emergency paediatric surgery in Tanzania. Pediatr Surg Int. 2008;24(2):219-22.
184. Migowa A, Colmegna I, Hitchon C, Were E, Ng'ang'a E, Ngwiri T, et al. The spectrum of rheumatic in-patient diagnoses at a pediatric hospital in Kenya. Pediatr. 2017;15(1):4.
185. Mioramalala SA, Ramasy Razafindratovo RM, Rakotozanany A, Miarimbola R, Weldegebriel G, Mwenda JM, Robinson AL. Analysis of Death and Survival Factors Associated with Childhood Bacterial Meningitis at a Reference Pediatric Hospital in Antananarivo, Madagascar. J Immunol Sci. 2018;Suppl(2):8-14.

186. Mitchell KB, Giiti G, Gallagher JJ. Survey of care and evaluation of East African burn unit feasibility: an academic burn center exchange. J Burn Care Res. 2013;34(1):78-81.
187. Moise IK. Causes of Morbidity and Mortality among Neonates and Children in Post-Conflict Burundi: A Cross-Sectional Retrospective Study. Children (Basel). 2018;5(9):08.
188. Mola K, Shimelis D. Pattern and Outcome of Renal Diseases in Hospitalized Children in Tikur Anbessa Specialized Teaching Hospital, Addis Ababa, Ethiopia. Ethiop Med J. 2016;54(3):117-23.
189. Mujuru HA, Kambarami RA. Mortality within 24 hours of admission to the Paediatric Unit, Harare Central Hospital, Zimbabwe. Cent Afr J Med. 2012;58(5-6):17-22.
190. Muoneke VU, Una AF, Eke CB, Anyanwu OU. The Burden and Outcome of Pediatric Renal Admissions at the Federal Teaching Hospital Abakaliki: A 3-year Review (2011-2013). ann. 2016;6(4):243-50.
191. Muro RP, Masoza TS, Kasanga G, Kayange N, Kidenya BR. Predictors and outcome of first line treatment failure among under-five children with community acquired severe pneumonia at Bugando Medical Centre, Mwanza, Tanzania: A prospective cohort study. PloS one. 2020;15(12):e0243636.
192. Mutombo AM, Mukuku O, Tshibanda KN, Swana EK, Mukomena E, Ngwej DT, et al. Severe malaria and death risk factors among children under 5 years at Jason Sendwe hospital in democratic republic of Congo. Pan African Medical Journal. 2018;29.
193. Mwangome M, Ngari M, Fegan G, Mturi N, Shebe M, Bauni E, Berkley JA. Diagnostic criteria for severe acute malnutrition among infants aged under 6 mo. The American journal of clinical nutrition. 2017;105(6):1415-23.
194. Mwaniki MK, Nokes DJ, Ignas J, Munywoki P, Ngama M, Newton CRJ, et al. Emergency triage assessment for hypoxaemia in neonates and young children in a Kenyan hospital: an observational study. Bulletin of the World Health Organization. 2009;87(4):263-70.
195. Nabukeera-Barungi N, Wilmshurst J, Rudzani M, Nuttall J. Presentation and outcome of tuberculous meningitis among children: experiences from a tertiary children's hospital. African health sciences. 2014;14(1):143-9.
196. Nakawesi JS, Wobudeya E, Ndeezi G, Mworozi EA, Tumwine JK. Prevalence and factors associated with rotavirus infection among children admitted with acute diarrhea in Uganda. BMC pediatrics. 2010;10:69.
197. Ndukwu CI, Onah SK. Pattern and outcome of postneonatal pediatric emergencies in Nnamdi Azikiwe University Teaching Hospital, Nnewi, South East Nigeria. Nigerian journal of clinical practice. 2015;18(3):348-53.
198. Ngari MM, Fegan G, Mwangome MK, Ngama MJ, Mturi N, Scott JAG, et al. Mortality after Inpatient Treatment for Severe Pneumonia in Children: a Cohort Study. Paediatr Perinat Epidemiol. 2017;31(3):233-42.
199. Ngari MM, Obiero C, Mwangome MK, Nyaguara A, Mturi N, Murunga S, et al. Mortality during and following hospital admission among school-aged children: a cohort study. Wellcome Open Res. 2020;5:234.
200. Ngirabega JD, Munyanshongore C, Donnen P, Dramaix M. [Influence of malnutrition on childhood mortality in a rural hospital in Rwanda]. Rev Epidemiol Sante Publique. 2011;59(5):313-8.
201. Ngoy BB, Zachariah R, Hinderaker SG, Khogali M, Manzi M, van Griensven J, et al. Paediatric in-patient care in a conflict-torn region of Somalia: are hospital outcomes of acceptable quality? Public health action. 2013;3(2):125-7.
202. Nhampossa T, Sigaúque B, Machevo S, Macete E, Alonso P, Bassat Q, et al. Severe malnutrition among children under the age of 5 years admitted to a rural district hospital in southern Mozambique. Public health nutrition. 2013;16(9):1565-74.
203. Nigussie B, Tadele H. Heart Failure in Ethiopian Children: Mirroring the Unmet Cardiac Services. Ethiop. 2019;29(1):811-8.

204. Njuguna P, Maitland K, Nyaguara A, Mwanga D, Mogeni P, Mturi N, et al. Observational study: 27 years of severe malaria surveillance in Kilifi, Kenya. BMC medicine. 2019;17(1):N.PAG-N.PAG.
205. Nokes DJ, Abwao J, Pamba A, Peenze I, Dewar J, Maghenda JK, et al. Incidence and clinical characteristics of group A rotavirus infections among children admitted to hospital in Kilifi, Kenya. PLoS Med. 2008;5(7):e153.
206. Nokes DJ, Ngama M, Bett A, Abwao J, Munywoki P, English M, et al. Incidence and severity of respiratory syncytial virus pneumonia in rural Kenyan children identified through hospital surveillance. Clinical infectious diseases : an official publication of the Infectious Diseases Society of America. 2009;49(9):1341-9.
207. Nyaga EM, Ndungu Jm JM, Anangwe GCN. Acute non-traumatic abdominal pain in childhood at Kenyatta National Hospital, Kenya. Annals of African Surgery. 2010;6:14-7.
208. Obonyo CO, Vulule J, Akhwale WS, Grobbee DE. In-hospital morbidity and mortality due to severe malarial anemia in western Kenya. The American journal of tropical medicine and hygiene. 2007;77(6 Suppl):23-8.
209. Obura B, Alele PE, Obua C. Off-label antibiotic use among paediatric in-patients: a mixed-method prospective study at a tertiary hospital in southwestern Uganda. Int J Clin Pharm. 2020;18:18.
210. Odetunde OI, Okafor HU, Uwaezuoke SN, Ezeonwu BU, Adiele KD, Ukoha OM. Chronic kidney disease in children as seen in a tertiary hospital in Enugu, South-East, Nigeria. Nigerian journal of clinical practice. 2014;17(2):196-200.
211. Ofovwe GE, Ibadin MO, Okunola PO, Ofoegbu B. Pattern of emergency neurologic morbidities in children. J Natl Med Assoc. 2005;97(4):488-92.
212. Ogunfowora OB, Ogunlesi TA, Oba-Daini OO. Epidemiological trend of post-neonatal tetanus in a Nigerian teaching hospital. SAJCH South African Journal of Child Health. 2019;13(4):158-63.
213. Okike CO, Muoneke UV, Uwaezuoke SN, Mbagwu EN, Onyeka-Okite E. The Prevalence and Case-Fatality Rates of Post-Neonatal Tetanus in a Population of Hospitalized Nigerian Children: An 8-Year Retrospective Review. Journal of tropical pediatrics. 2020;66(2):201-9.
214. Okiro EA, Kazembe LN, Kabaria CW, Ligomeka J, Noor AM, Ali D, Snow RW. Childhood malaria admission rates to four hospitals in Malawi between 2000 and 2010. PloS one. 2013;8(4):e62214.
215. Okoroiwu HU, Uchendu KI, Essien RA. Causes of morbidity and mortality among patients admitted in a tertiary hospital in southern Nigeria: A 6 year evaluation. PloS one. 2020;15(8):e0237313.
216. Okoronkwo NC, Onyearugha CN, Ohanenye CA. Pattern and outcomes of paediatric medical admissions at the Living Word Mission Hospital, Aba, South East Nigeria. Pan Afr Med J. 2018;30:202.
217. Olatunya OS, Isinkaye AO, Ogundare EO, Oluwayemi IO, Akinola FJ. Childhood poisoning at a tertiary hospital in South West Nigeria. Journal of Nepal Paediatric Society. 2015;35(2):103-10.
218. Oliwa JN, Gathara D, Ogero M, Van Hensbroek MB, English M, Van't Hoog A. Diagnostic practices and estimated burden of tuberculosis among children admitted to 13 government hospitals in Kenya: An analysis of two years' routine clinical data. PloS one. 2019;14(9).
219. Olorunmoteni OE, Onyia CU, Elusiyan JBE, Ugowe OJ, Babalola TE, Samuel I. Intracranial abscesses in children at Ile-Ife, Nigeria: a case series and review of literature. Childs Nerv Syst. 2020;36(8):1767-71.
220. Olowu WA, Adefehinti O, Bisiriyu AL. Hospital-acquired acute kidney injury in critically ill children and adolescents. Saudi J Kidney Dis Transpl. 2012;23(1):68-77.

221. Olupot-Olupot P, Engoru C, Nteziyaremye J, Chebet M, Ssenyondo T, Muhindo R, et al. The clinical spectrum of severe childhood malaria in Eastern Uganda. Malar J. 2020;19(1):322.
222. Oluwayemi OI, Brown BJ, Oyedeji OA, Adegoke SA, Adebami OJ, Oyedeji GA. Clinical and laboratory predictors of outcome in cerebral malaria in suburban Nigeria. J. 2013;7(8):600-7.
223. Omore R, Khagayi S, Ogwel B, Onkoba R, Ochieng JB, Juma J, et al. Rates of hospitalization and death for all-cause and rotavirus acute gastroenteritis before rotavirus vaccine introduction in Kenya, 2010-2013. BMC Infectious Diseases. 2019;19(1).
224. Opoka RO, Ssemata AS, Oyang W, Nambuya H, John CC, Tumwine JK, Karamagi C. High rate of inappropriate blood transfusions in the management of children with severe anemia in Ugandan hospitals. BMC health services research. 2018;18(1):566.
225. Orimadegun AE, Fawole O, Okereke JO, Akinbami FO, Sodeinde O. Increasing burden of childhood severe malaria in a Nigerian tertiary hospital: implication for control. Journal of tropical pediatrics. 2007;53(3):185-9.
226. Osano BO, Were F, Mathews S. Mortality among 5-17 year old children in Kenya. Pan Afr Med J. 2017;27:121.
227. Oshikoya KA, Ogunyinka IA, Imuzei SE, Garba BI, Jiya NM. A Retrospective Audit of Pharmacologic and Non-Pharmacologic Management of Childhood Acute Asthma Exacerbation at Usmanu Danfodiyo University Teaching Hospital, Sokoto: Adherence to Global Treatment Guidelines. Front Pharmacol. 2020;11:531894.
228. Ouedraogo SM, Toloba Y, Ouedraogo G, Badoum G, Boncoungou K, Bambara M, et al. [Epidemio-clinical aspects of bacterial acute infant Pneumopathies at Yalgado Ouedraogo University Health Center]. Mali med. 2010;25(3):19-22.
229. Oyedeji OA, Fadero F, Joel-Medewase V, Elemile P, Oyedeji GA. Trends in neonatal and post-neonatal tetanus admissions at a Nigerian teaching hospital. J. 2012;6(12):847-53.
230. e Pinto EA, Alves JG. The causes of death of hospitalized children in Angola. Tropical doctor. 2008;38(1):66-7.
231. Rahajamanana VL, Raboba JL, Rakotozanany A, Razafindraibe NJ, Andriatahirintsoa E, Razafindrakoto AC, et al. Impact of rotavirus vaccine on all-cause diarrhea and rotavirus hospitalizations in Madagascar. Vaccine. 2018;36(47):7198-204.
232. Richards M, Le Roux D, Cooke L, Argent A. The Influence of High Flow Nasal Cannulae on the Outcomes of Severe Respiratory Disease in Children Admitted to a Regional Hospital in South Africa. Journal of tropical pediatrics. 2020;66(6):612-20.
233. Roca A, Quinto L, Abacassamo F, Morais L, Valles X, Espasa M, et al. Invasive Haemophilus influenzae disease in children less than 5 years of age in Manhica, a rural area of southern Mozambique. Tropical medicine & international health : TM & IH. 2008;13(6):818-26.
234. Rudd KE, Tutaryebwa LK, West TE. Presentation, management, and outcomes of sepsis in adults and children admitted to a rural Ugandan hospital: A prospective observational cohort study. PloS one. 2017;12(2):e0171422.
235. Ngo Um Sap S, Tchaptchet K, Mekone I, Minka B, Mbono RC, Tony J, et al. Mortality of children aged 5-15 years in a tertiary care center in Yaounde, Cameroon. Arch Pediatr. 2020;27(5):257-60.
236. Sawadogo S, Nebie K, Millogo T, Kafando E. Blood transfusion requirements among children with severe malarial anemia: a cross-sectional study in a second level reference hospital in Burkina Faso. Pan Afr Med J. 2020;37:108.
237. Seck N, Basse I, Keïta Y, Boiro D, Thiam L, Ndongo AA, Diagne I. Bronchiolitis in tropical environment. Journal de Pediatrie et de Puericulture. 2018;31(5):241-6.
238. Sidibe T, Sangho H, Traore MS, Cisse MB, Togo B, Sy O, Keita MM. [Morbidity and mortality in the pediatric service at Gabriel Toure's University Hospital in Mali]. Mali med. 2008;23(4):34-7.

239. Sievers AC, Lewey J, Musafiri P, Franke MF, Bucyibaruta BJ, Stulac SN, et al. Reduced paediatric hospitalizations for malaria and febrile illness patterns following implementation of community-based malaria control programme in rural Rwanda. Malar J. 2008;7:167.
240. Sigauque B, Roca A, Mandomando I, Morais L, Quinto L, Sacarlal J, et al. Community-acquired bacteremia among children admitted to a rural hospital in Mozambique. The Pediatric infectious disease journal. 2009;28(2):108-13.
241. Sigauque B, Roca A, Bassat Q, Morais L, Quinto L, Berenguera A, et al. Severe pneumonia in Mozambican young children: clinical and radiological characteristics and risk factors. Journal of tropical pediatrics. 2009;55(6):379-87.
242. Sigauque B, Verani JR, Massora S, Vubil D, Quinto L, Acacio S, et al. Burden of invasive pneumococcal disease among children in rural Mozambique: 2001-2012. PloS one. 2018;13(1):e0190687.
243. Silaba M, Ooko M, Bottomley C, Sande J, Benamore R, Park K, et al. Effect of 10-valent pneumococcal conjugate vaccine on the incidence of radiologically-confirmed pneumonia and clinically-defined pneumonia in Kenyan children: an interrupted time-series analysis. The Lancet Global health. 2019;7(3):e337-e46.
244. Smart LR, Orgenes N, Mazigo HD, Minde M, Hokororo A, Shakir M, et al. Malaria and HIV among pediatric inpatients in two Tanzanian referral hospitals: A prospective study. Acta tropica. 2016;159:36-43.
245. Sylla A, Gueye M, Keita Y, Seck N, Seck A, Mbow F, et al. [Dehydration and malnutrition as two independent risk factors of death in a Senegalese pediatric hospital]. Arch Pediatr. 2015;22(3):235-40.
246. Tette EM, Neizer M, Nyarko MY, Sifah EK, Nartey ET, Donkor ES. Changing Patterns of Disease and Mortality at the Children's Hospital, Accra: Are Infections Rising? PloS one. 2016;11(4):e0150387.
247. Theo A, Tempia S, Cohen AL, Simusika P, Chentulo E, Chikamukwa CM, Monze M. The national burden of influenza-associated severe acute respiratory illness hospitalization in Zambia, 2011-2014. Influenza other respi. 2018;12(1):46-53.
248. Tornheim JA, Manya AS, Oyando N, Kabaka S, O'Reilly CE, Breiman RF, Feikin DR. The epidemiology of hospitalization with diarrhea in rural Kenya: the utility of existing health facility data in developing countries. Int J Infect Dis. 2010;14(6):e499-505.
249. Tsai C, Walters CB, Sampson J, Kateh F, Chang MP. Pediatric Mortality in a Rural Tertiary Care Center in Liberia. Children (Basel). 2017;4(2):30.
250. Ugege MO, Chikani UN, Yusuf T, Amodu-Sanni M, Ibitoye PK, Abdul Rahman MB. Abnormal blood glucose and the relationship with clinical outcome in acutely ill children admitted to the emergency unit of a Nigerian Tertiary Hospital. Nigerian journal of clinical practice. 2021;24(2):205-12.
251. Ugwu GI, Nwajei G, Chinemelu U. Pattern of Renal Diseases among Children in The Niger Delta Region, Nigeria. Arab J Nephrol Transplant. 2014;7(1):49-50.
252. Vonasek BJ, Chiume M, Crouse HL, Mhango S, Kondwani A, Ciccone EJ, et al. Risk factors for mortality and management of children with complicated severe acute malnutrition at a tertiary referral hospital in Malawi. Paediatrics and international child health. 2020;40(3):148-57.
253. Zeidan Z, Kojal H, Habour A, Nowary K, Hashim F, Awadelkarim M. Clinical and epidemiological features of severe malaria in children in four hospitals in Sudan. Eastern Mediterranean health journal = La revue de sante de la Mediterranee orientale = al-Majallah al-sihhiyah li-sharq al-mutawassit. 2006;12(6):783-91.

Supplementary Table 10. Recommended basic pediatric emergency and critical care resources, including medications, supplies, and workforce by organ system.

Organ System	Medications	Supplies	Paediatric Workforce
All systems	Antipyretics<br>Dextrose containing, isotonic IV fluids	Vital sign monitoring and appropriately sized equipment<br>• Blood pressure cuff<br>• Pulse oximeter<br>• Thermometer<br>• Cardiac monitor<br>• Respiratory monitor<br>Hand hygiene and infection prevention supplies<br>IV placement supplies<br>Central venous catheters	Paediatric sub-specialists<br>• Emergency medicine<br>• Intensive care<br>• Hospitalist medicine<br>• Surgery<br>• Anaesthesia<br>• Radiology<br>Paediatric nursing<br>• Emergency<br>• Critical care<br>• General medical and surgical
Respiratory	Sedatives<br>Analgesics<br>Bronchodilators<br>Corticosteroids<br>Diuretics	Pulse oximeter<br>Blood gas analyser<br>Oxygen<br>Low-and high-flow nasal cannula<br>Continuous positive airway pressure<br>Bilevel-positive airway pressure<br>Nebulizers<br>Chest tubes<br>Portable ultrasound<br>Portable X-ray	Respiratory therapist<br>Radiology technicians<br>Paediatric pulmonologist<br>Paediatric sub-specialists<br>Paediatric nursing
Infectious Diseases	Antibiotics<br>Antivirals<br>Antimalarials<br>Antifungals<br>Antiparasitic<br>Antipyretics<br>Analgesics<br>Vasopressors<br>Inotropes	Supplies to collect blood, urine, and CSF for culture<br>Personal protective equipment<br>Pressure infusion bag for IV fluids<br>Diagnostic testing kits	Laboratory technologists<br>Microbiologist<br>Paediatric infectious disease physician<br>Paediatric sub-specialists<br>Paediatric nursing
Gastrointestinal	Oral rehydration solution<br>Proton pump inhibitors<br>Antiemetics<br>Vitamin A<br>Zinc	Nasogastric tubes<br>Enteral nutrition<br>Therapeutic milk formulas and ready-to-use therapeutic food<br>Portable ultrasound<br>Portable X-ray<br>Computed tomography	Nutritionists<br>Paediatric gastroenterologist<br>Paediatric sub-specialists<br>Paediatric nursing
Neurological	Antipyretics<br>Analgesics<br>Sedatives<br>Antiepileptics<br>Antibiotics<br>Antivirals<br>Hyperosmolar therapy<br>Muscle relaxants	Electroencephalogram<br>Temperature probe (rectal/oesophageal)<br>External ventricular catheters<br>Computed tomography	Paediatric neurologist<br>Neurosurgeons<br>Operating theatre nurses and technicians<br>Paediatric anaesthesiologists<br>Paediatric sub-specialists<br>Paediatric nursing
Haematologic/Oncologic	Antiemetics	Transfusion supplies	Paediatric haematologic/oncologic specialists

	Chemotherapy<br>Anticoagulants and antiplatelet agents<br>Immunosuppressants<br>Hematopoietic growth factors<br>Antipyretics<br>Analgesics<br>Antiepileptics<br>Hydroxyurea<br>Blood products	Personal protective equipment	Pathologists<br>Blood bank/transfusion services<br>Paediatric sub-specialists<br>Paediatric nursing
Trauma	Blood products<br>Analgesics<br>Anticoagulants<br>Antibiotics<br>Vasopressors	Chest tubes<br>Haemostatic agents and dressings<br>Computed tomography<br>Portable ultrasound<br>Portable X-ray<br>Transfusion supplies	Paediatric emergency physicians<br>Surgeons<br>Paediatric anaesthesiologists<br>Blood bank/transfusion services<br>Operating theatre nurses and technicians<br>Paediatric sub-specialists<br>Paediatric nursing<br>Physical therapists<br>Occupational therapists
Renal	Albumin<br>Diuretics<br>Antihypertensives	Peritoneal dialysis catheters<br>Foley catheters<br>Electrocardiogram machine	Nephrologists<br>Paediatric sub-specialists<br>Paediatric nursing
Cardiovascular	Vasopressors<br>Inotropes<br>Antiarrhythmics<br>Anticoagulants and antiplatelet agents<br>Prostaglandins	Chest tubes<br>Defibrillators<br>Electrocardiogram machine<br>Echocardiography	Paediatric cardiologists<br>Paediatric cardiac surgeons<br>Operating theatre nurses and technicians<br>Paediatric anaesthesiologists<br>Paediatric sub-specialists<br>Paediatric nursing
Surgical Conditions	Antipyretics<br>Analgesics<br>Vasopressors<br>Antiemetics<br>Anaesthetics<br>Proton pump inhibitors and Hydrogen blockers<br>Sedatives<br>Blood products	Portable ultrasound<br>Sterile surgical instruments<br>Suture materials<br>Drainage devices<br>Wound care supplies<br>Sterile personal protective equipment<br>Portable X-ray<br>Computed tomography<br>Transfusion supplies<br>Mechanical ventilators and advanced airway supplies	Paediatric surgeons<br>Paediatric Anaesthesiologists<br>Operating theatre nurses and technicians<br>Blood product/transfusion services<br>Paediatric sub-specialists<br>Paediatric nursing
Congenital Anomalies*	Analgesics<br>Sedatives<br>Diuretics<br>Prostaglandins<br>Hormone replacement therapy	Nasogastric tubes<br>Enteral nutrition<br>Oxygen<br>Portable X-ray	Geneticists<br>Paediatric sub-specialists<br>Paediatric nursing
Endocrine	Insulin<br>Glucocorticoids<br>Vasopressin<br>Thyroid hormones and antithyroid hormones	Glucose monitoring supplies<br>Insulin administration supplies<br>Hormonal and metabolic lab tests	Paediatric endocrinologists<br>Paediatric sub-specialists<br>Paediatric nursing
*Congenital Anomalies can include any system<br>IV: intravenous; CSF: cerebrospinal fluid			

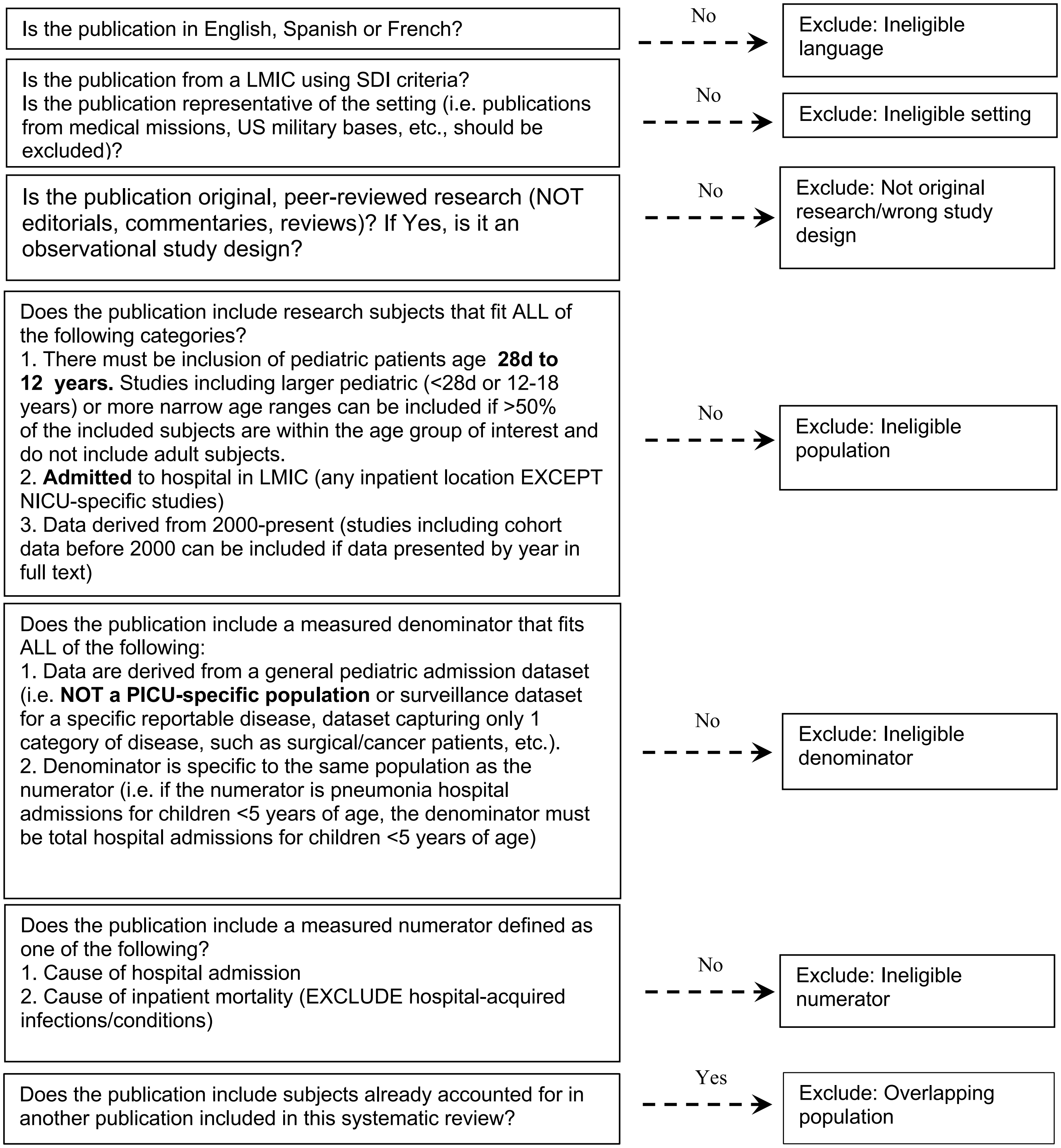

Supplementary Figure 1. Systematic review inclusion and exclusion criteria
General guidance/approach: The full text must include both the numerator and denominator as described below to be included. Please only select 'Yes' or 'No'; avoid selecting 'Maybe'.

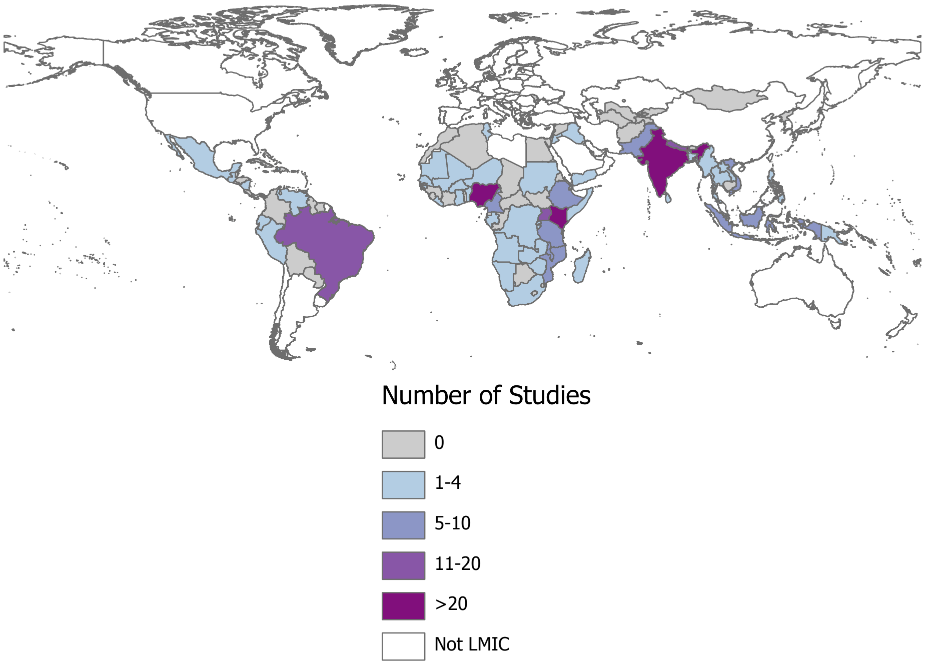

Supplementary Figure 2. Map of distribution of included studies

The map depicts locations of the 253 included studies. Darker colours represent countries with more studies included in the review. Countries that are not low-, low-middle- or middle-income countries (LMICs) were not included in the review and are depicted in white.

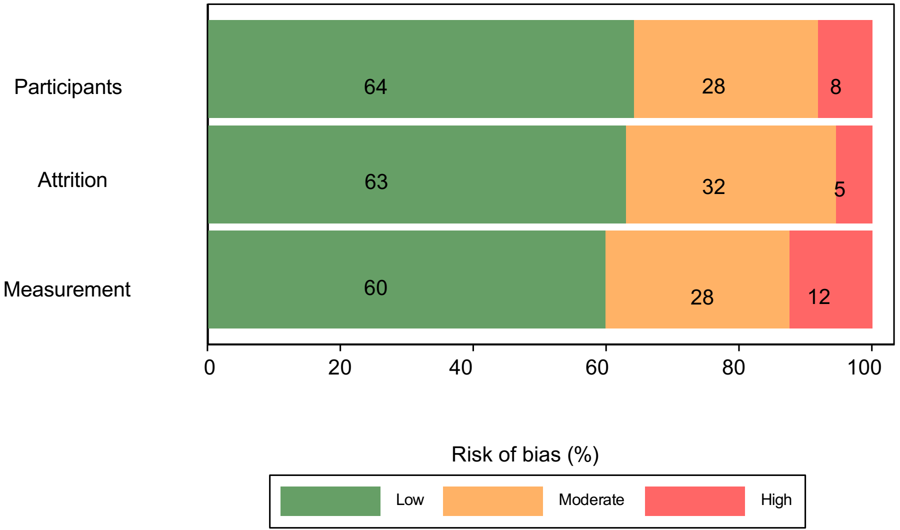

Supplementary Figure 3. Risk of bias assessment summary
Risk of bias was classified as low, moderate, or high for three domains (study participation and generalizability to underlying population; study attrition; and factor/cause of admission measurement) adapted from the Quality in Prognosis Studies (QUIPS) tool.

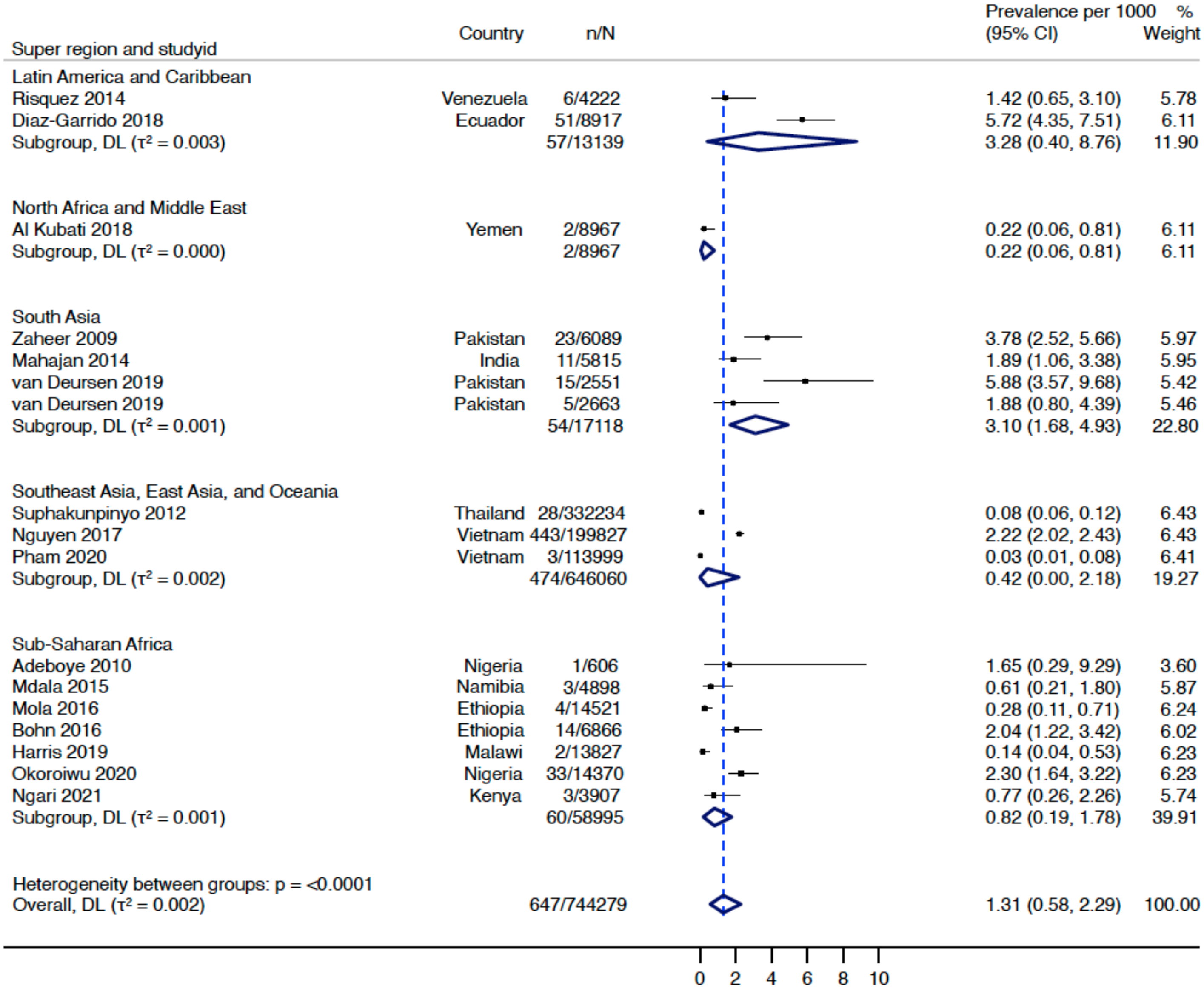

Supplementary Figure 4. Forest plot for causes of hospital mortality: System Congenital anomalies – death

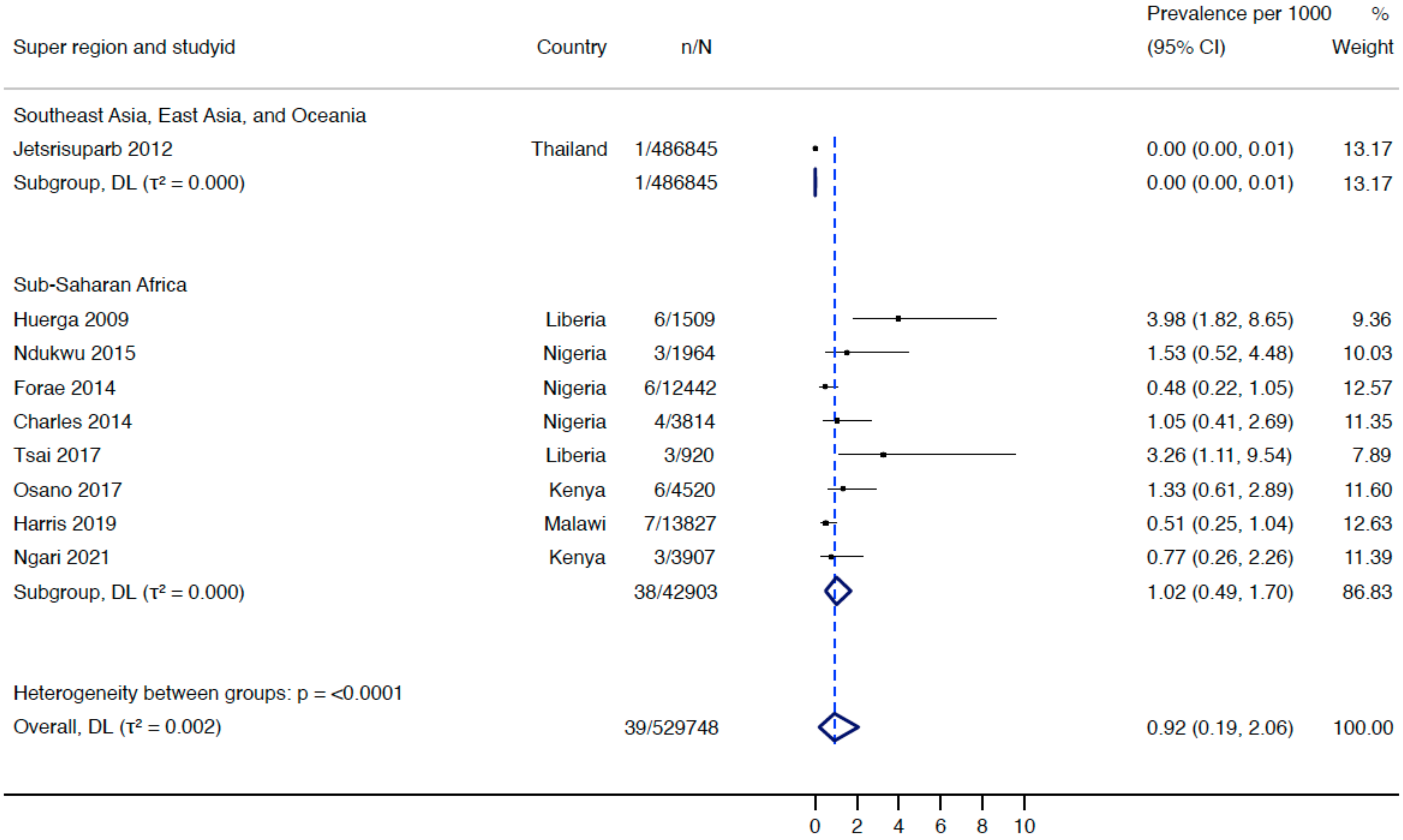

Supplementary Figure 4.1. Forest plot for causes of hospital mortality: System Surgical condition – death

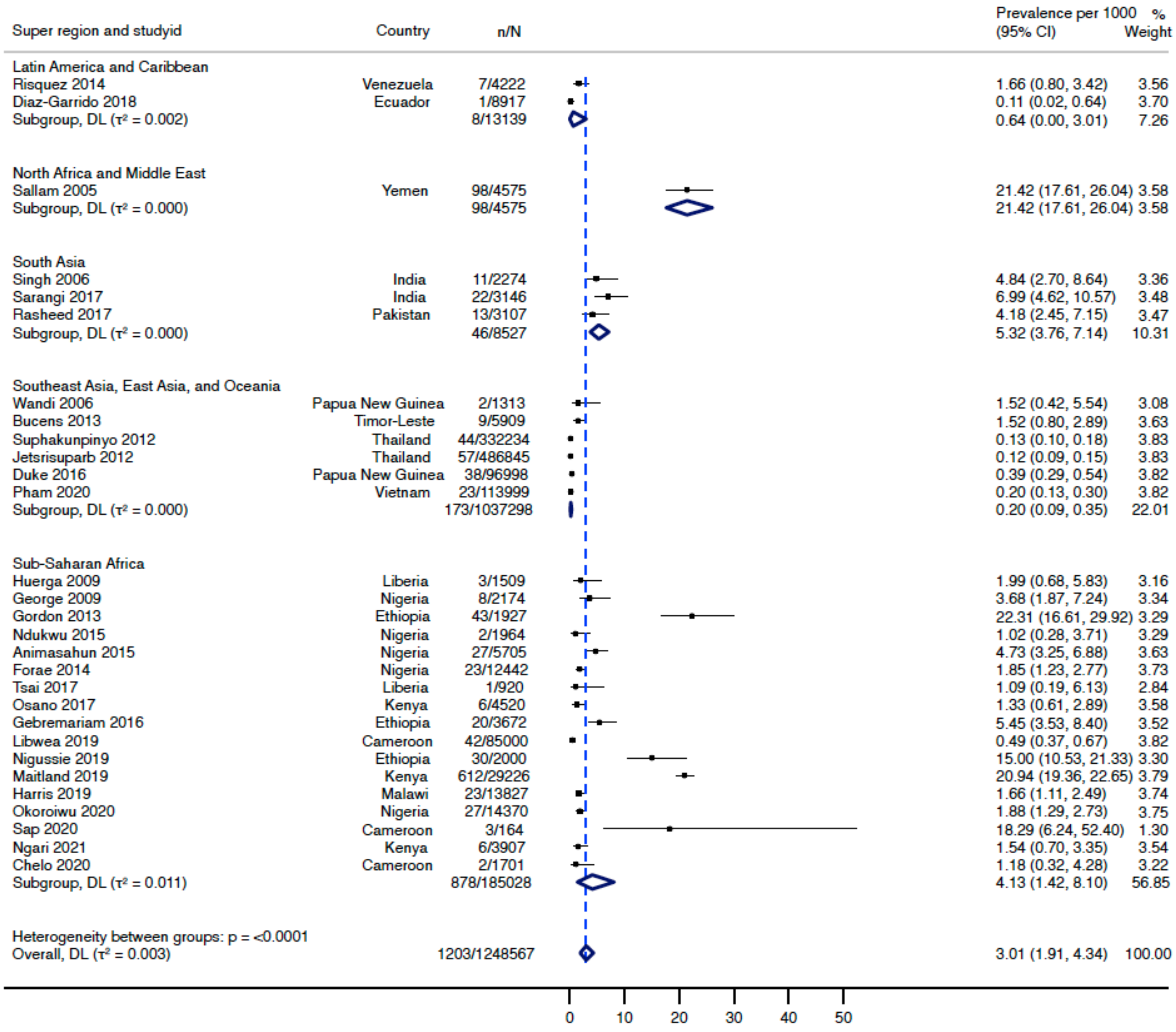

Supplementary Figure 4.2. Forest plot for causes of hospital mortality: System Cardiovascular – death

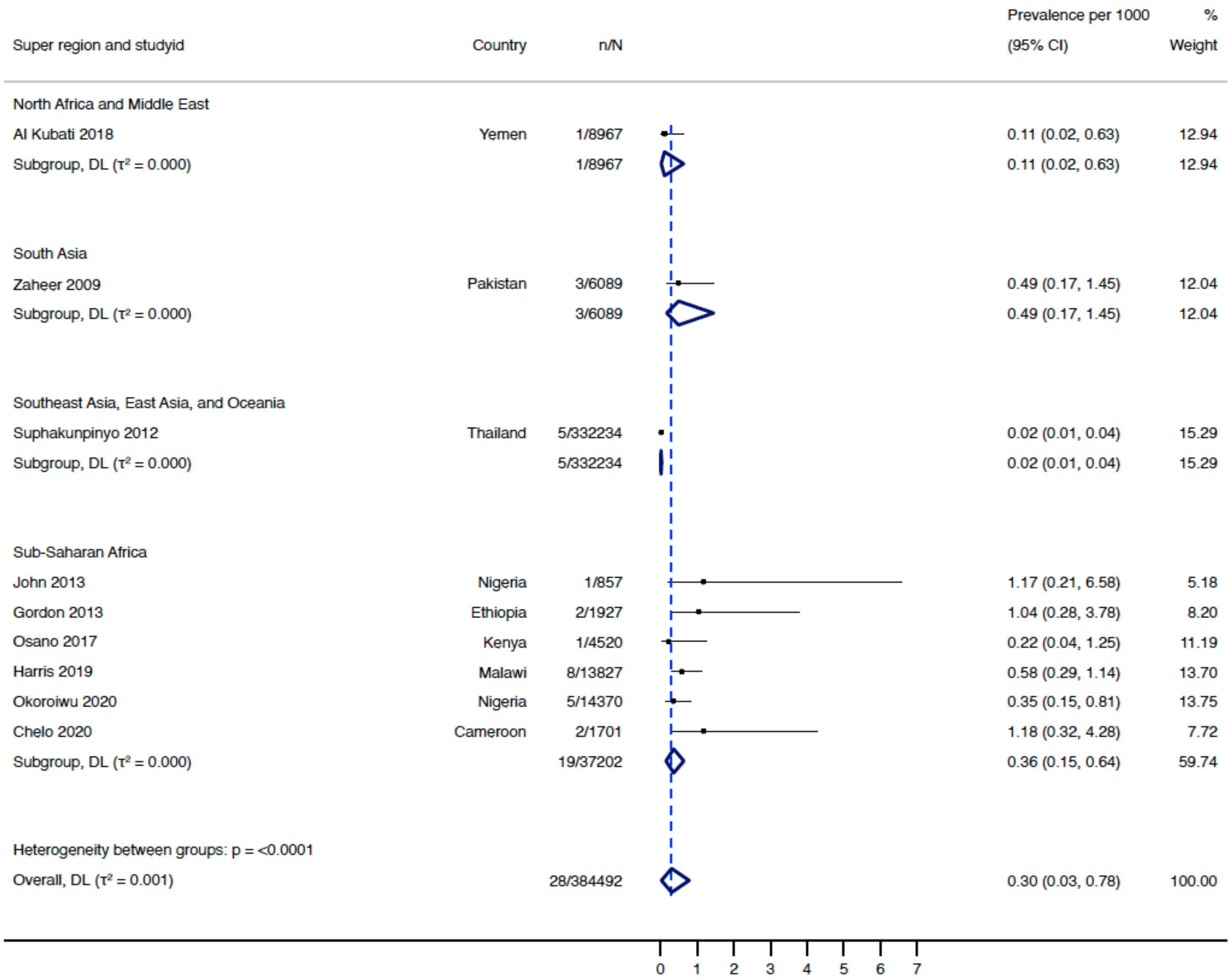

Supplementary Figure 4.3. Forest plot for causes of hospital mortality: System Endocrine– death

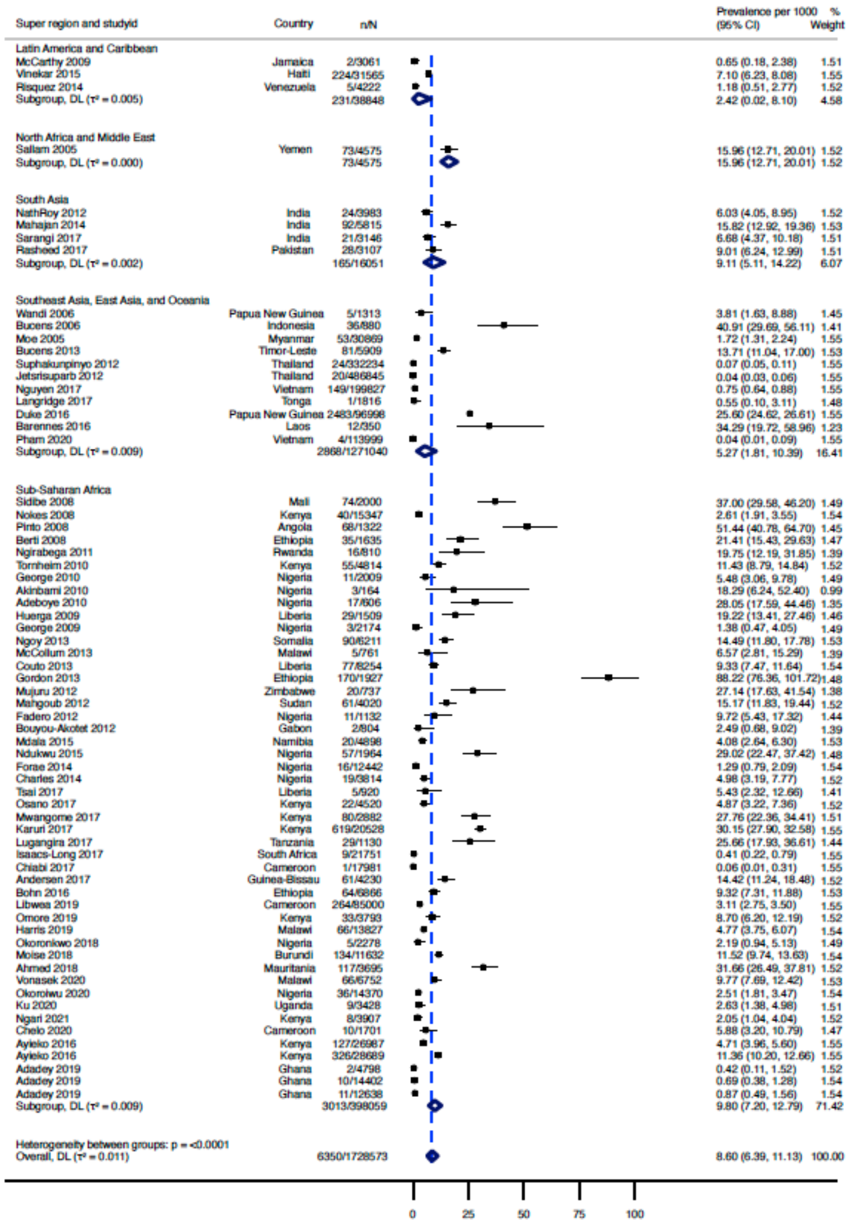

Supplementary Figure 4.4. Forest plot for causes of hospital mortality: System Gastrointestinal– death

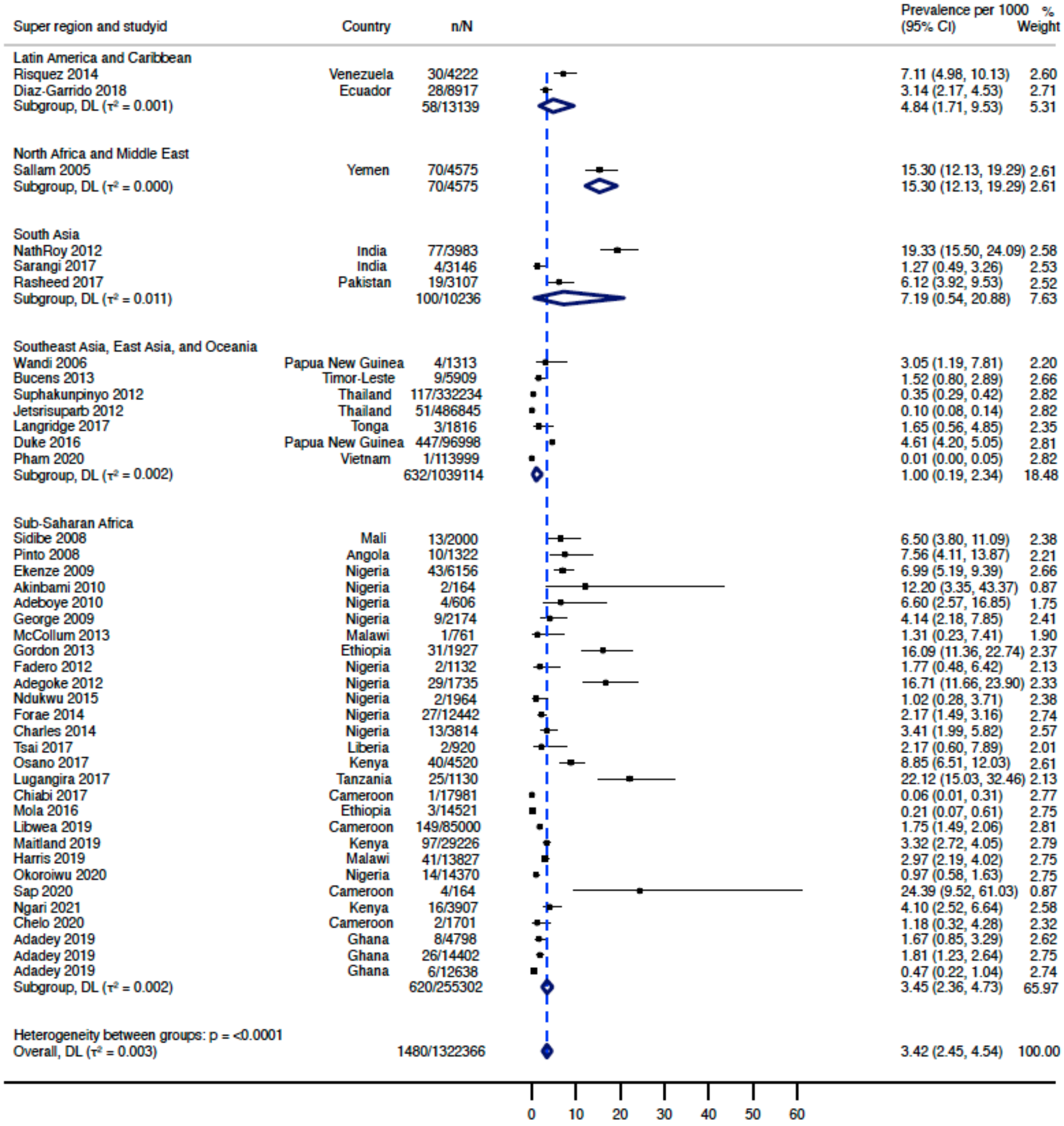

Supplementary Figure 4.5 Forest plot for causes of hospital mortality: System Hematologic– death

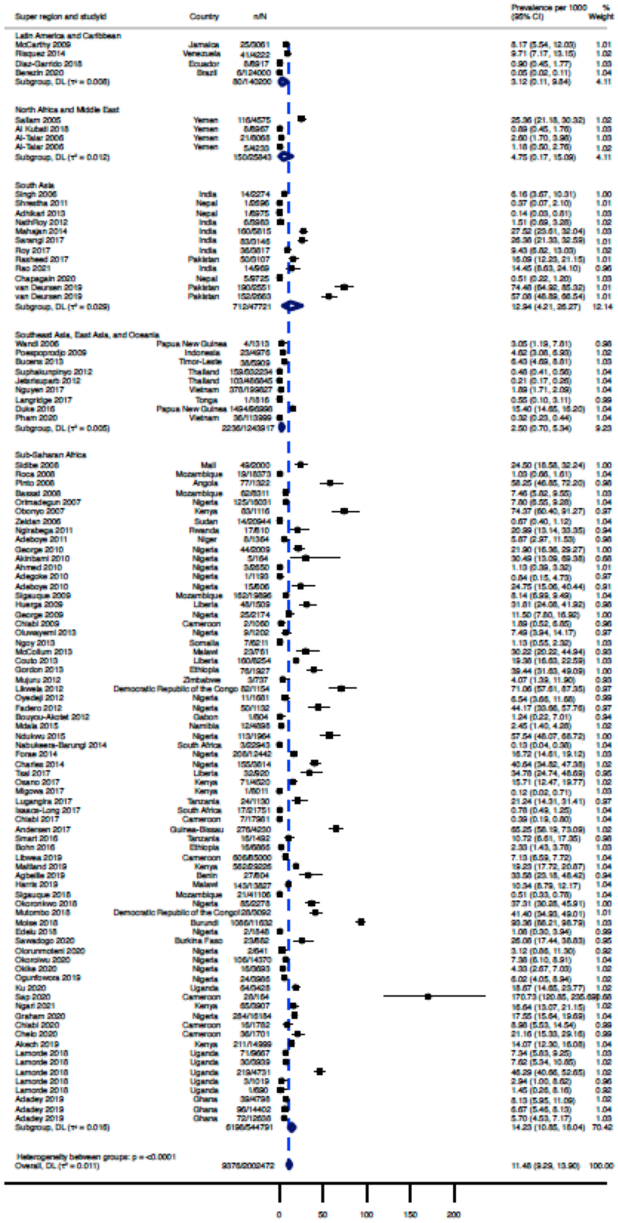

Supplementary Figure 4.6. Forest plot for causes of hospital mortality: System Infection– death

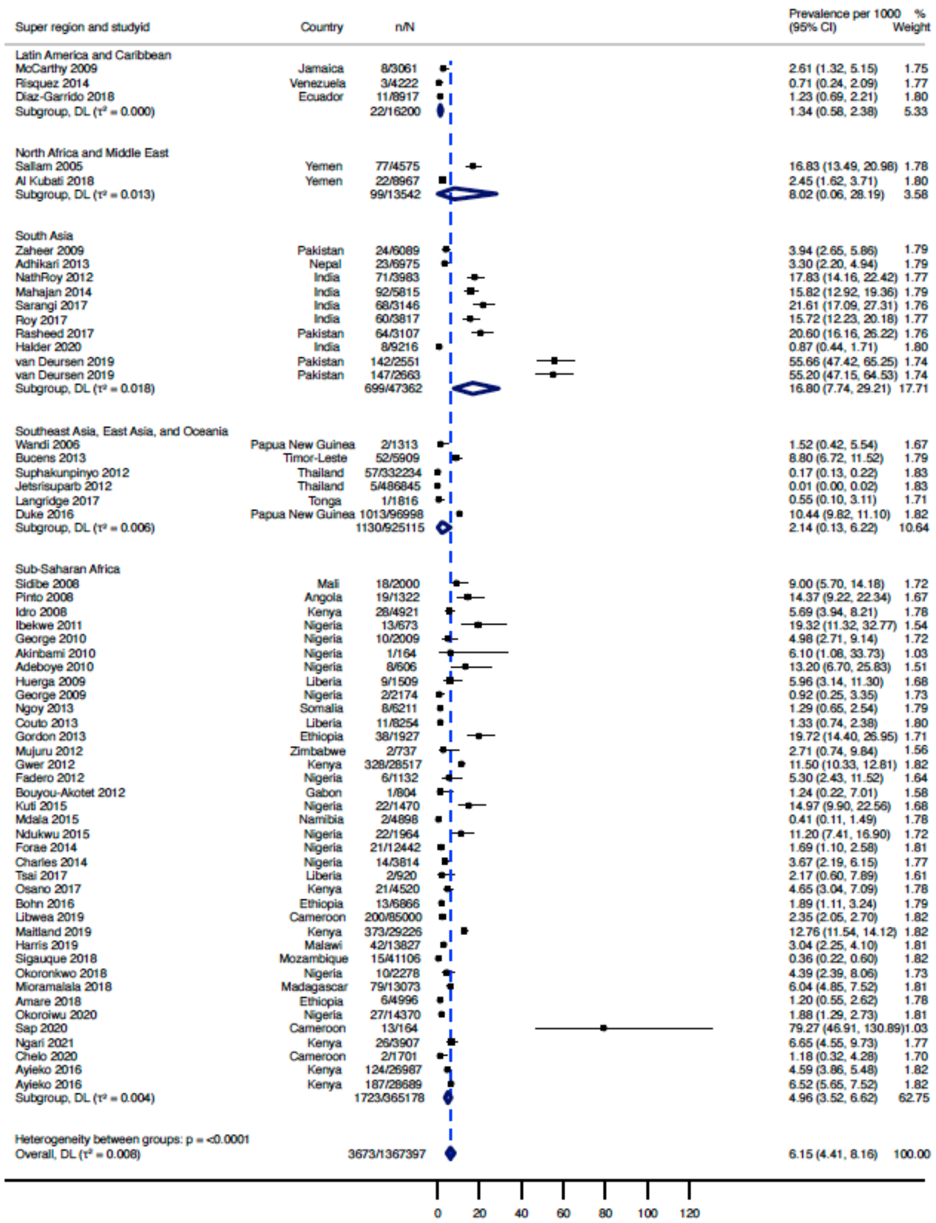

Supplementary Figure 4.7. Forest plot for causes of hospital mortality: System Neurological– death

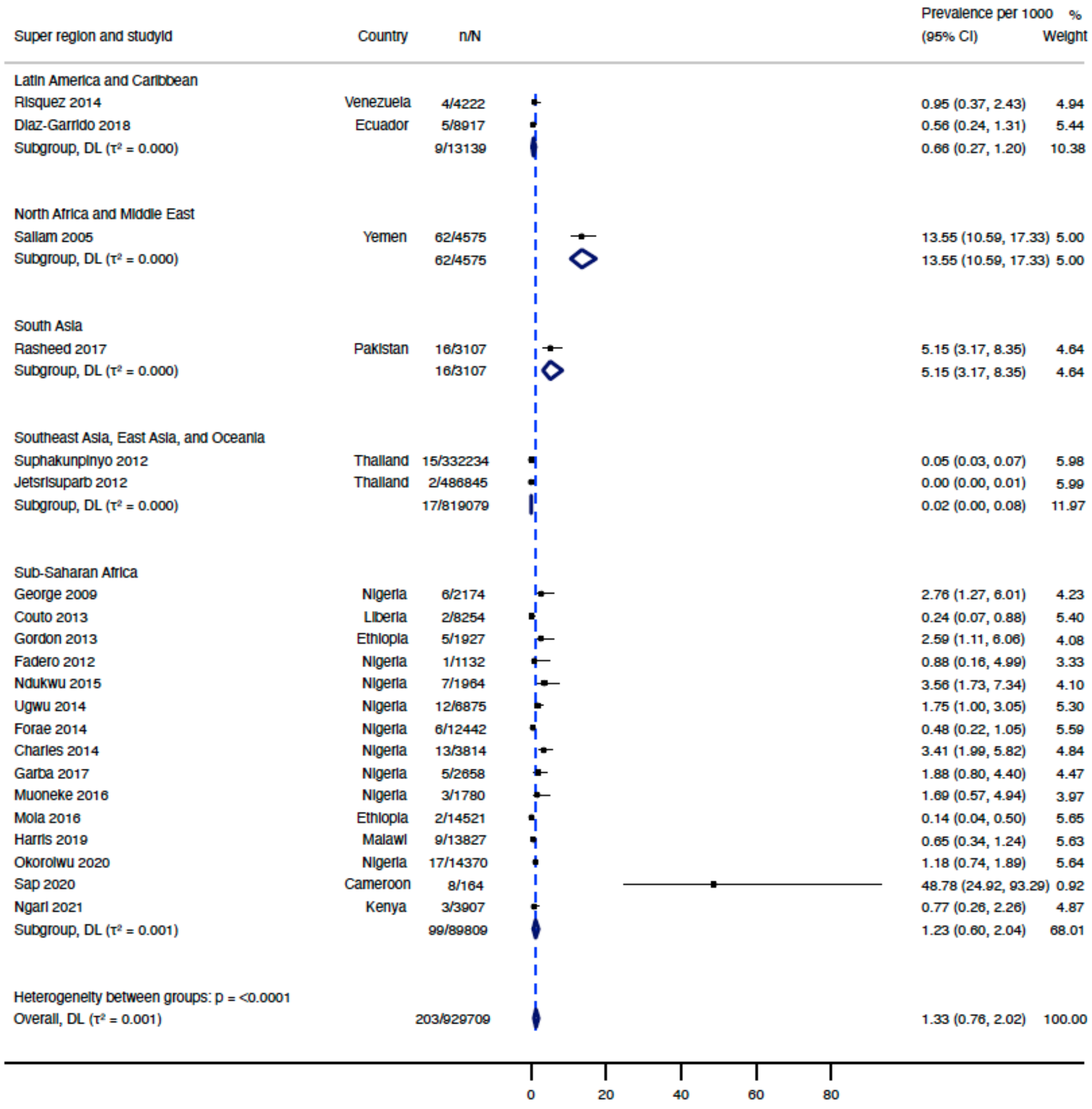

Supplementary Figure 4.8. Forest plot for causes of hospital mortality: System Renal– death

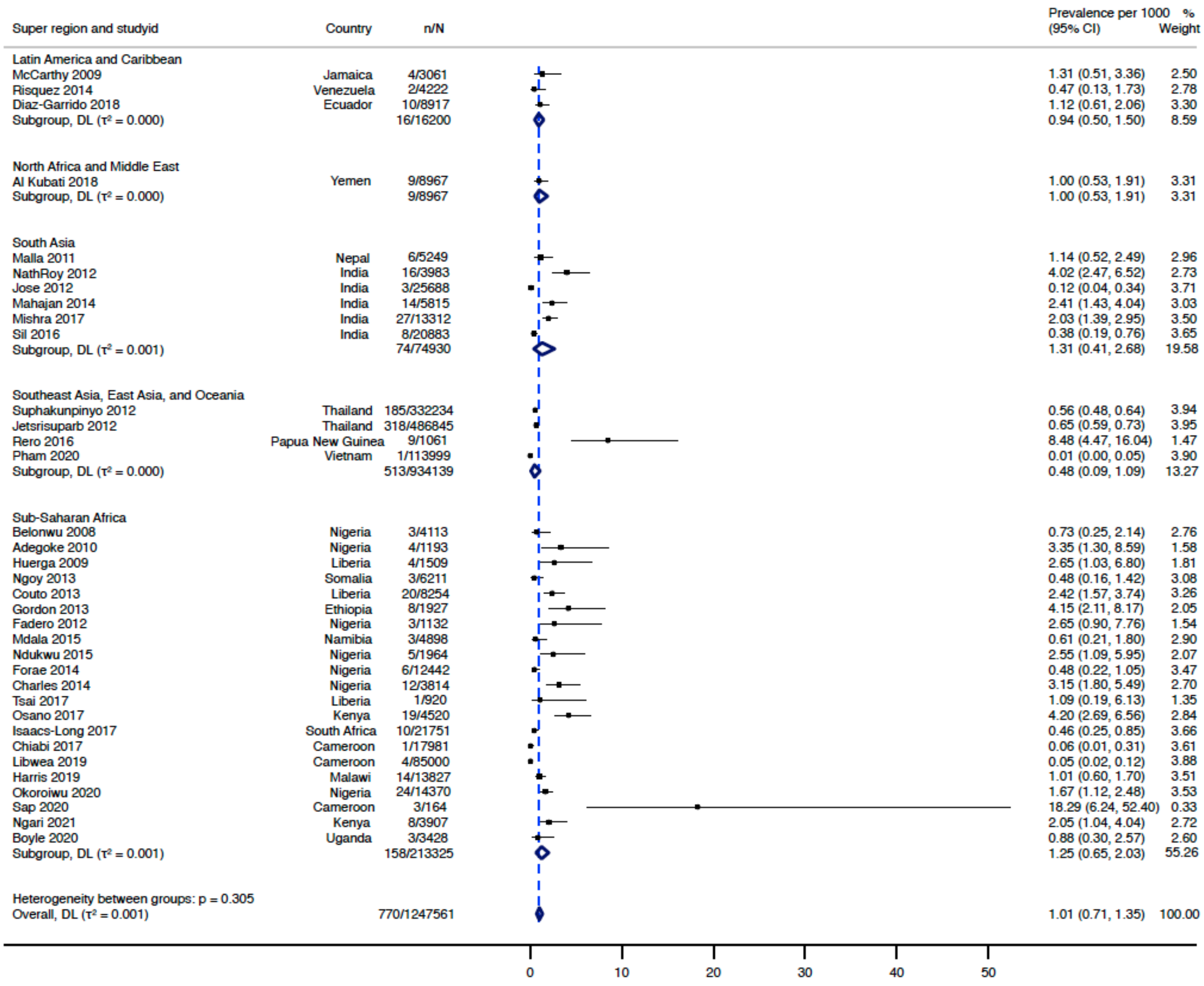

Supplementary Figure 4.9. Forest plot for causes of hospital mortality: System Trauma– death

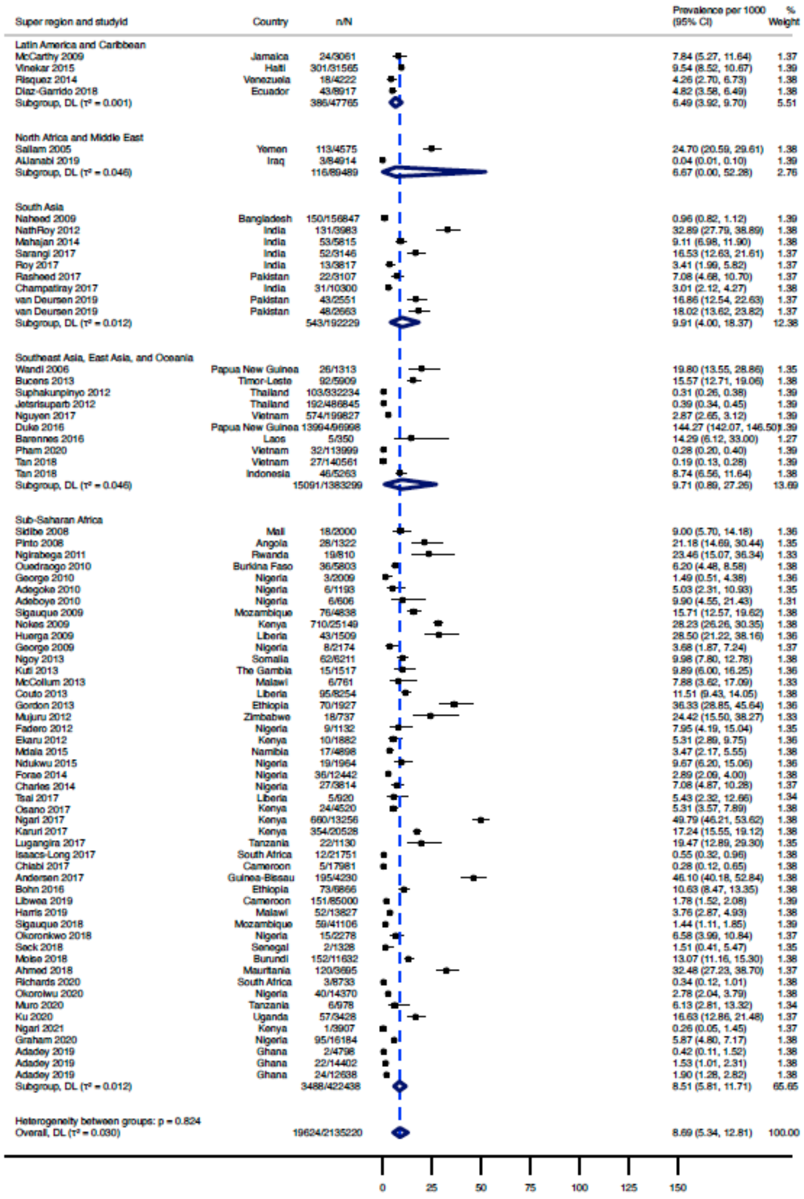

Supplementary Figure 4.10. Forest plot for causes of hospital mortality: System Respiratory– death

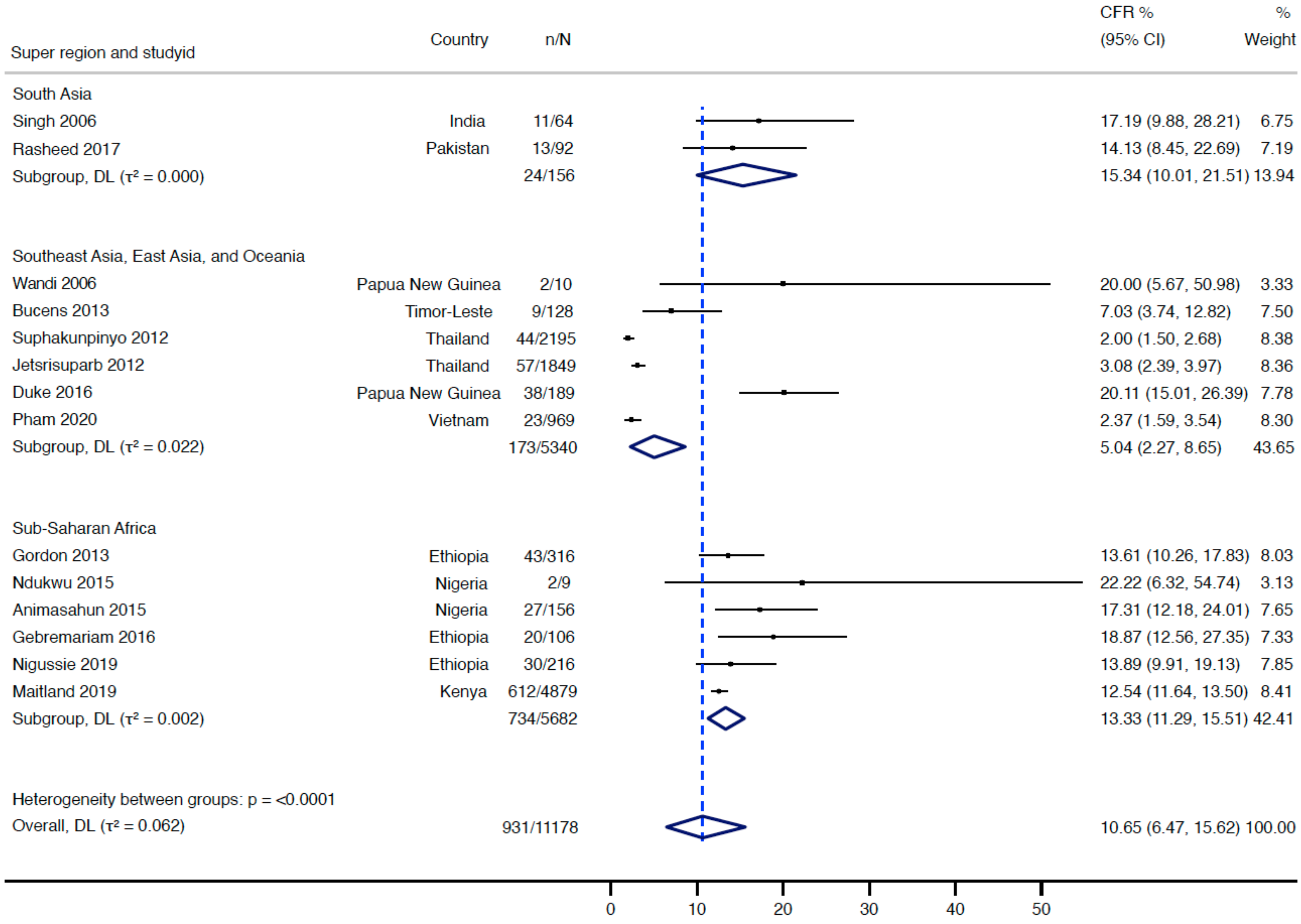

Supplementary Figure 5. Forest plot of case fatality rates (CFR): System Cardiovascular - CFR

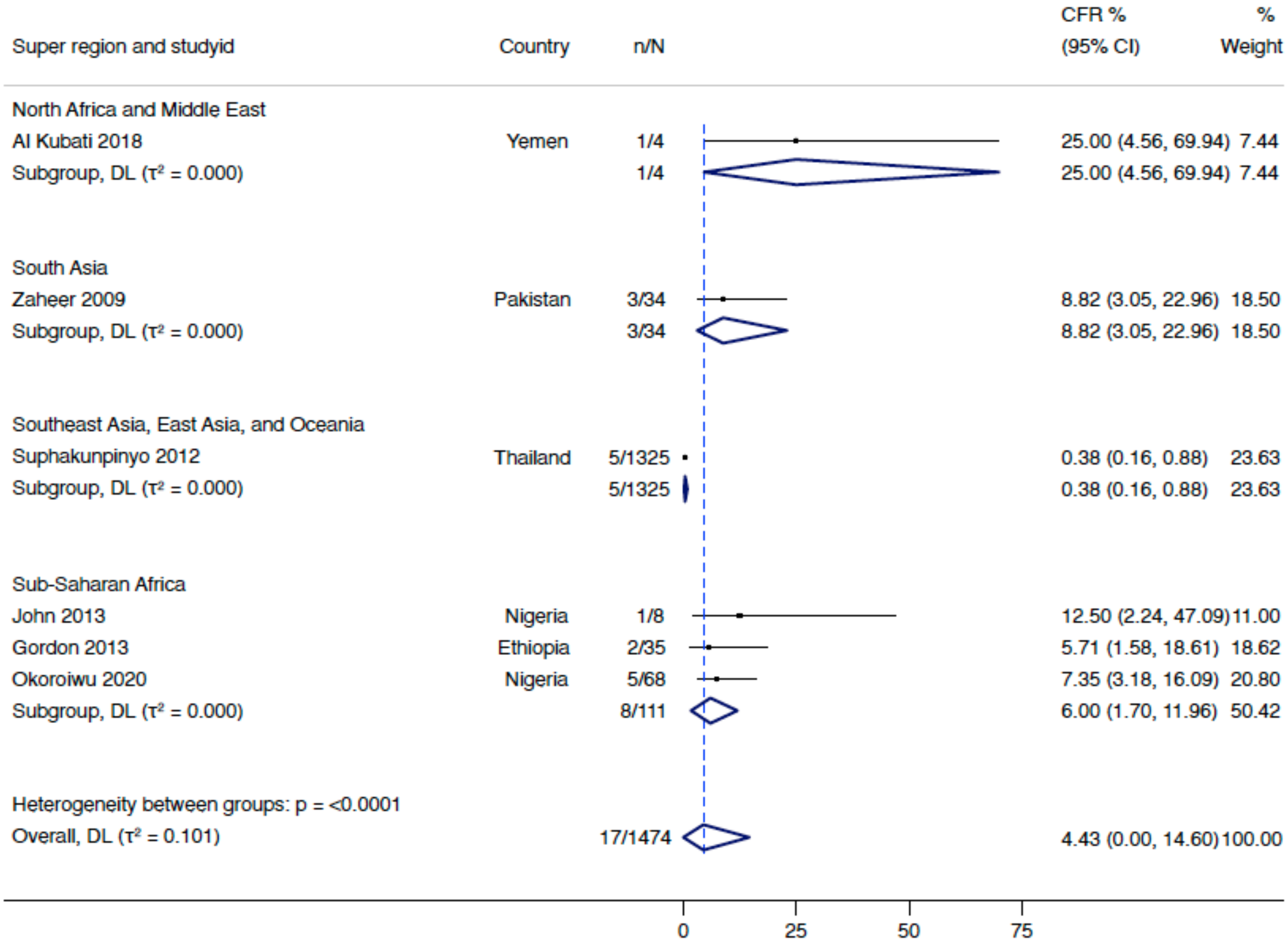

Supplementary Figure 5.1. Forest plot of case fatality rates (CFR): System Endocrine - CFR

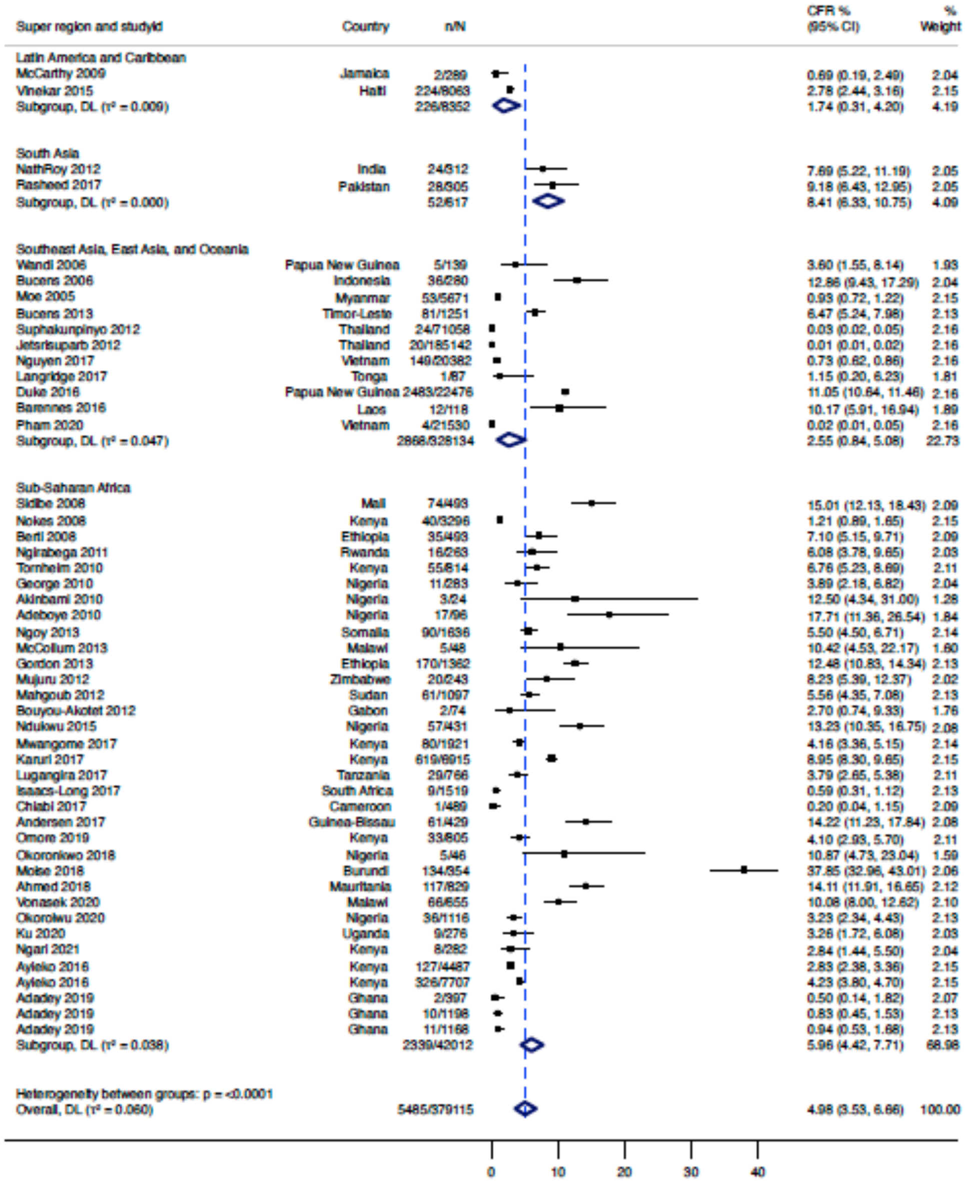

Supplementary Figure 5.2. Forest plot of case fatality rates (CFR): System Gastrointestinal - CFR

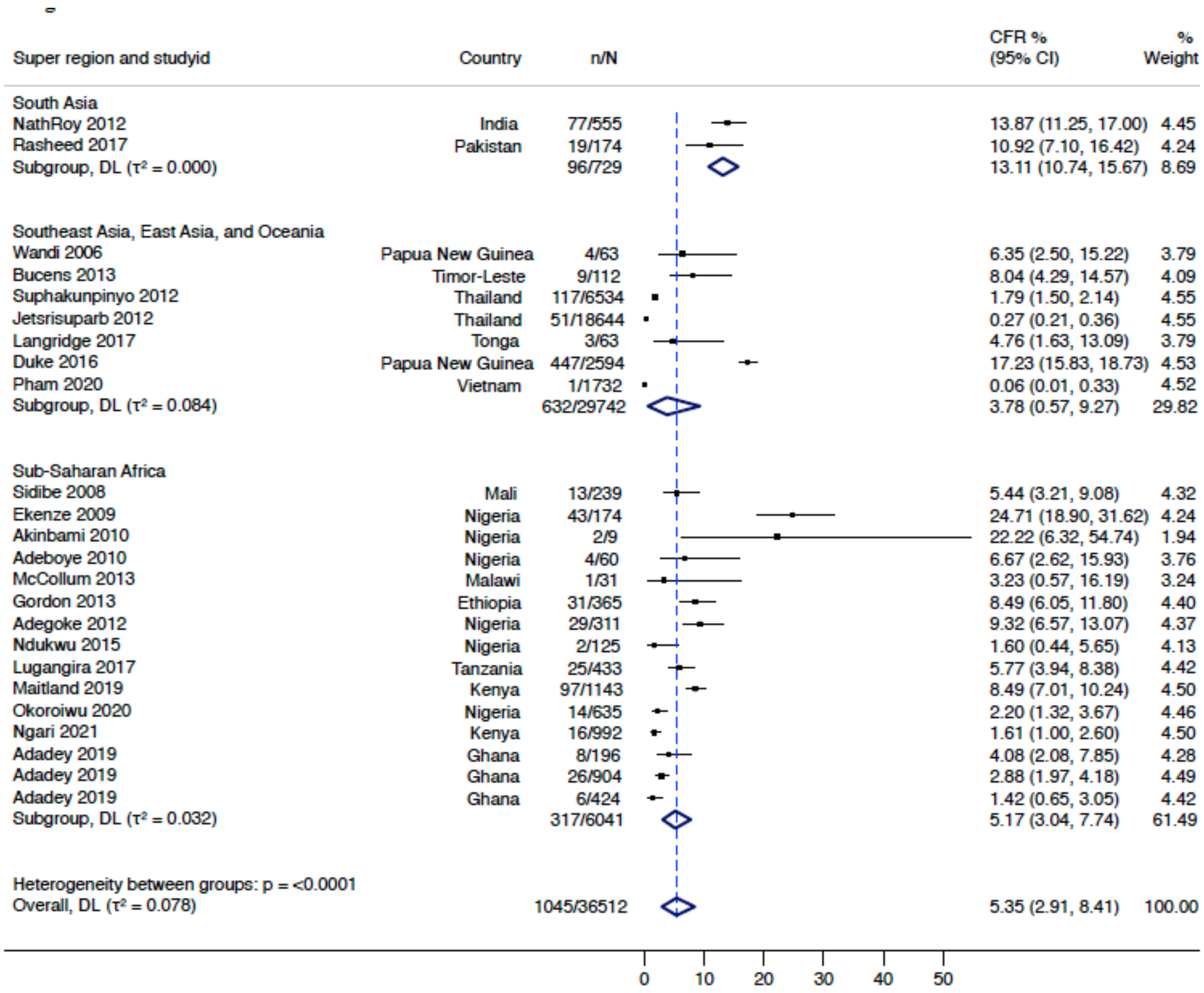

Supplementary Figure 5.3. Forest plot of case fatality rates (CFR): System Hematologic- CFR

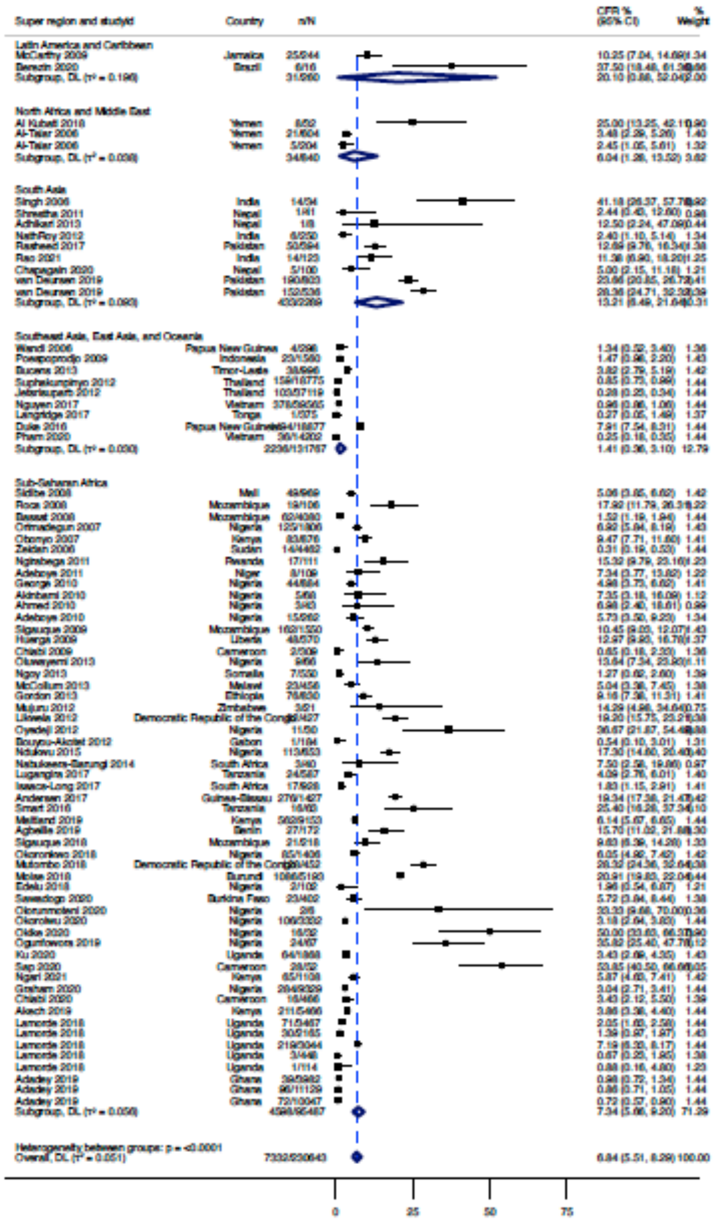

Supplementary Figure 5.4. Forest plot of case fatality rates (CFR): System Infection- CFR

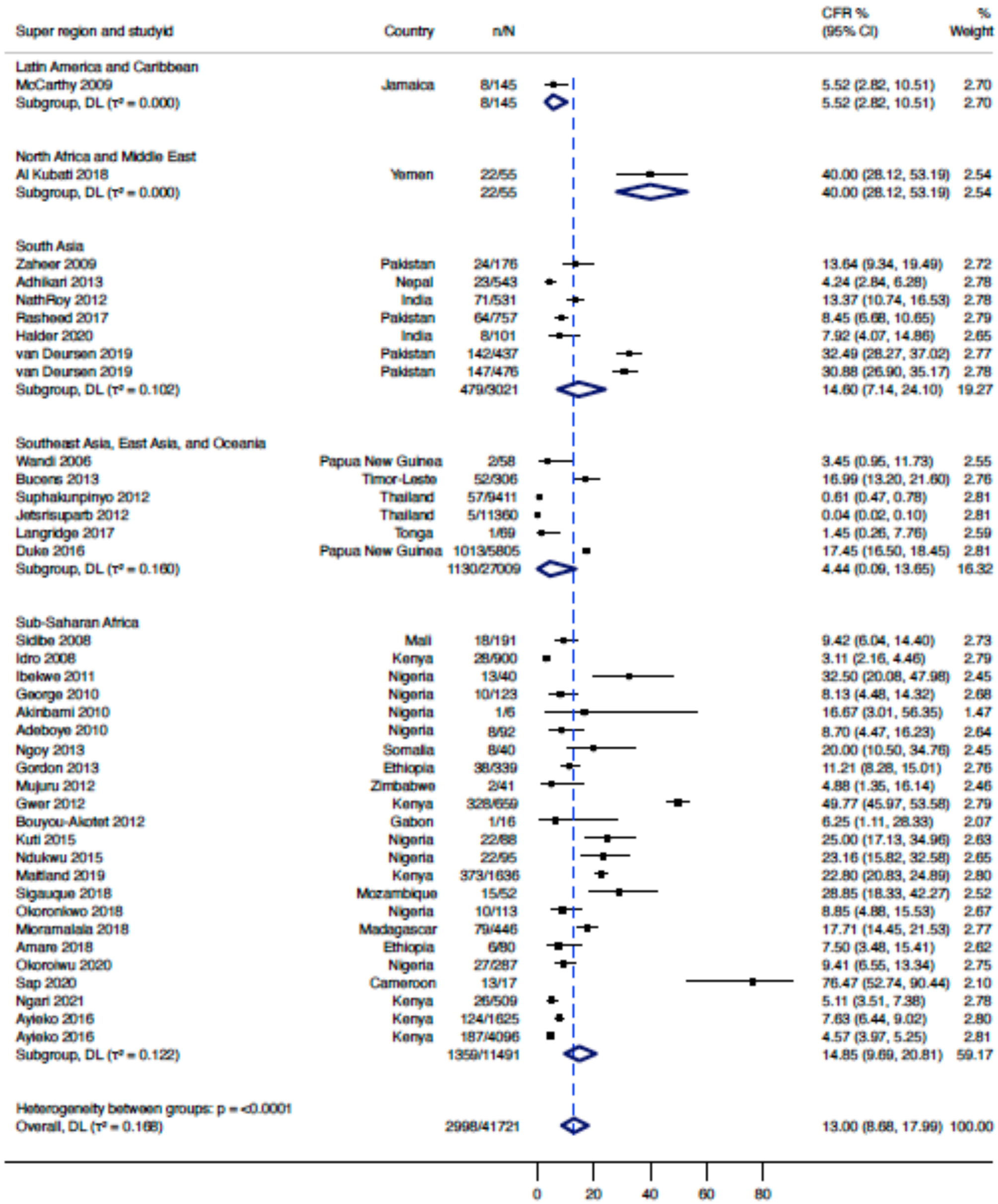

Supplementary Figure 5.5. Forest plot of case fatality rates (CFR): System Neurological- CFR

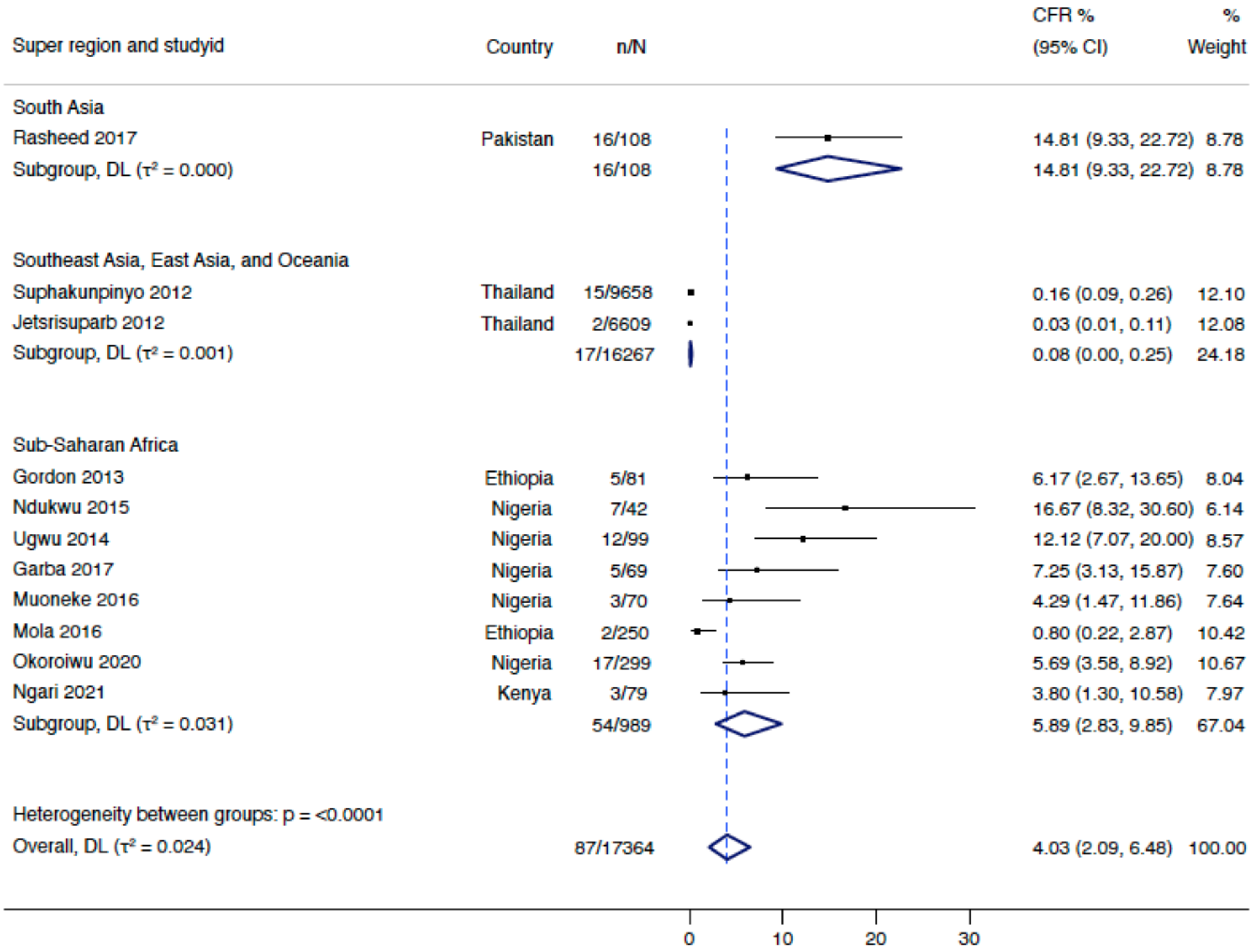

Supplementary Figure 5.6. Forest plot of case fatality rates (CFR): System Renal- CFR

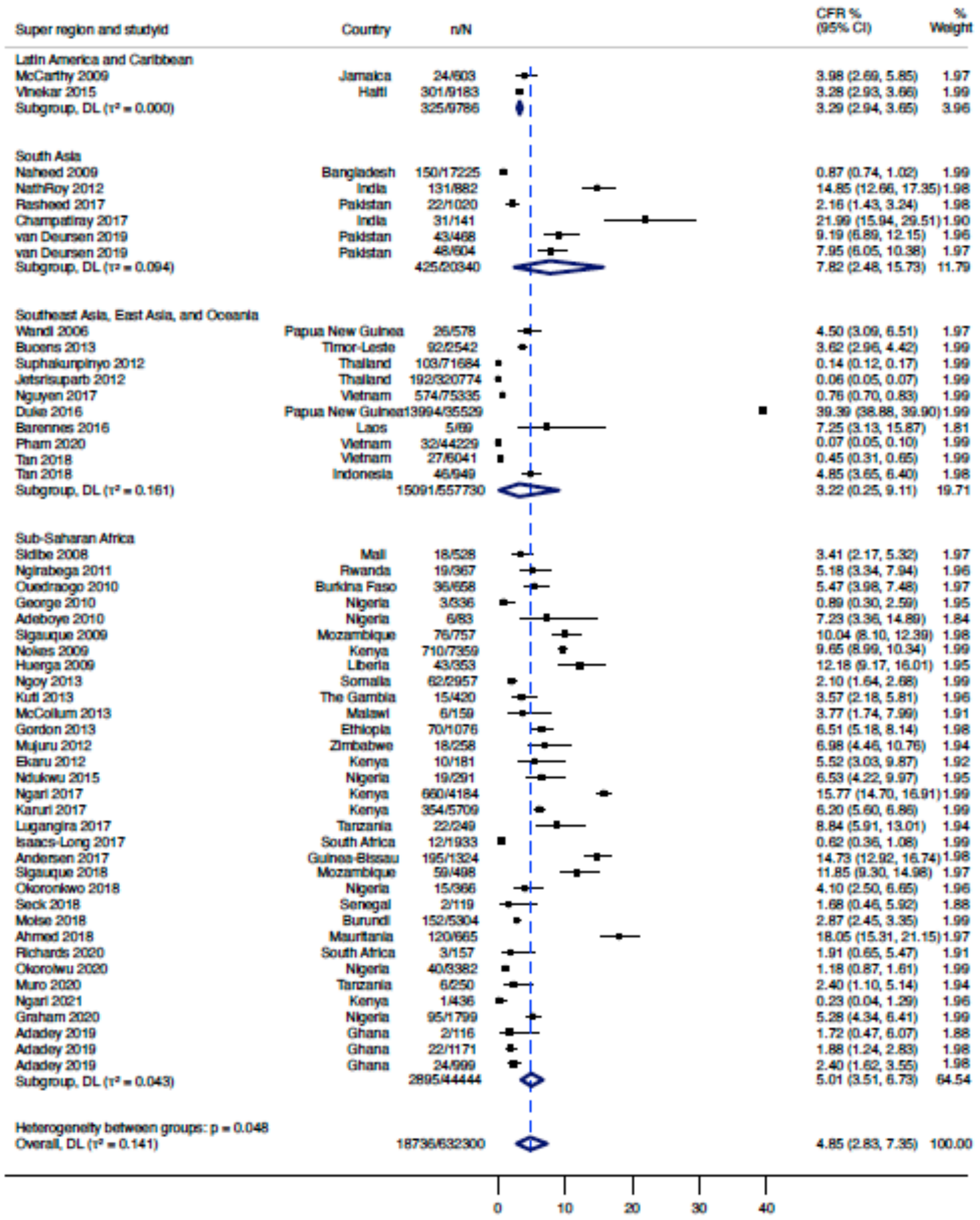

Supplementary Figure 5.7. Forest plot of case fatality rates (CFR): System Respiratory- CFR

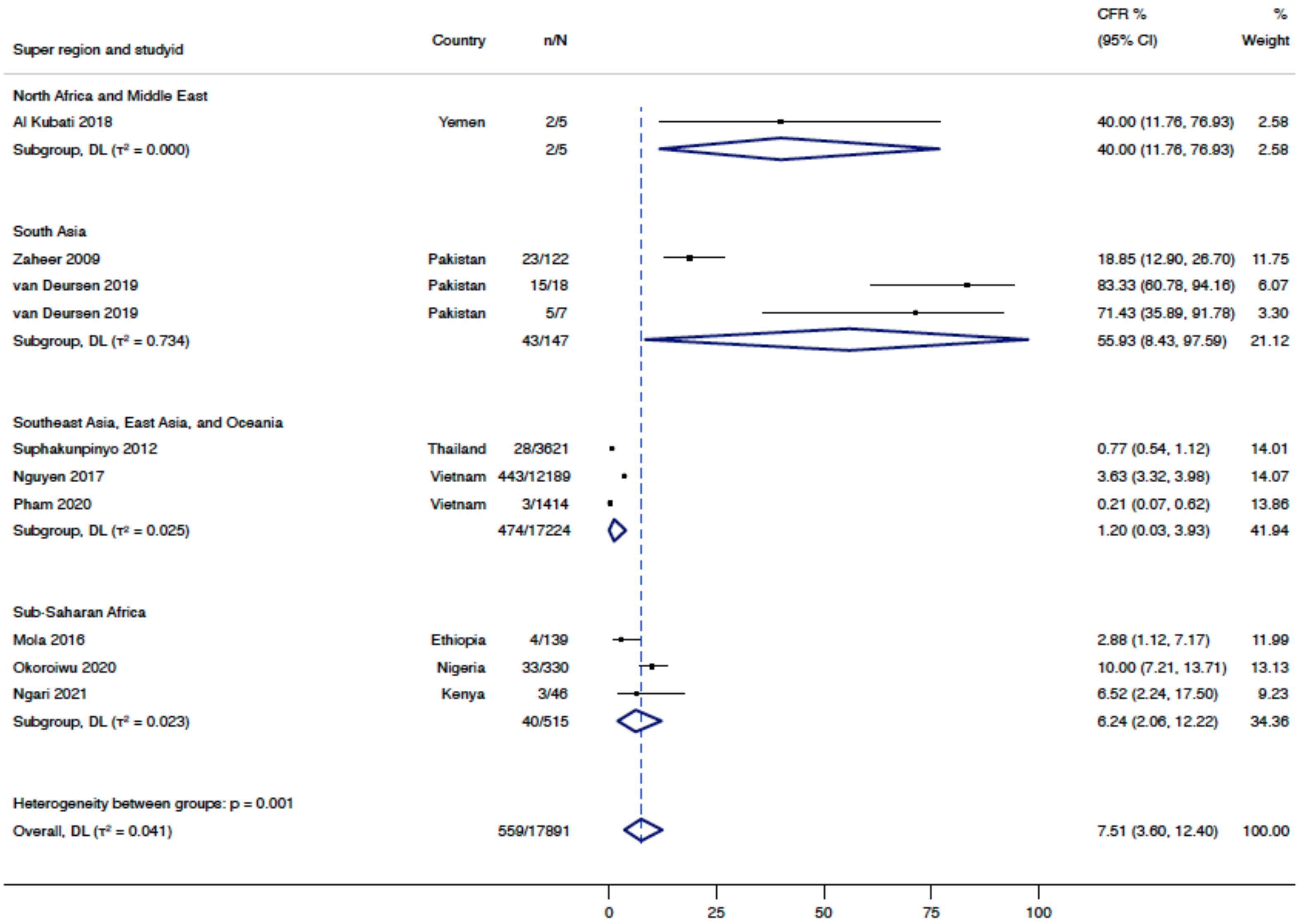

Supplementary Figure 5.8. Forest plot of case fatality rates (CFR): System Surgical Conditions-CFR

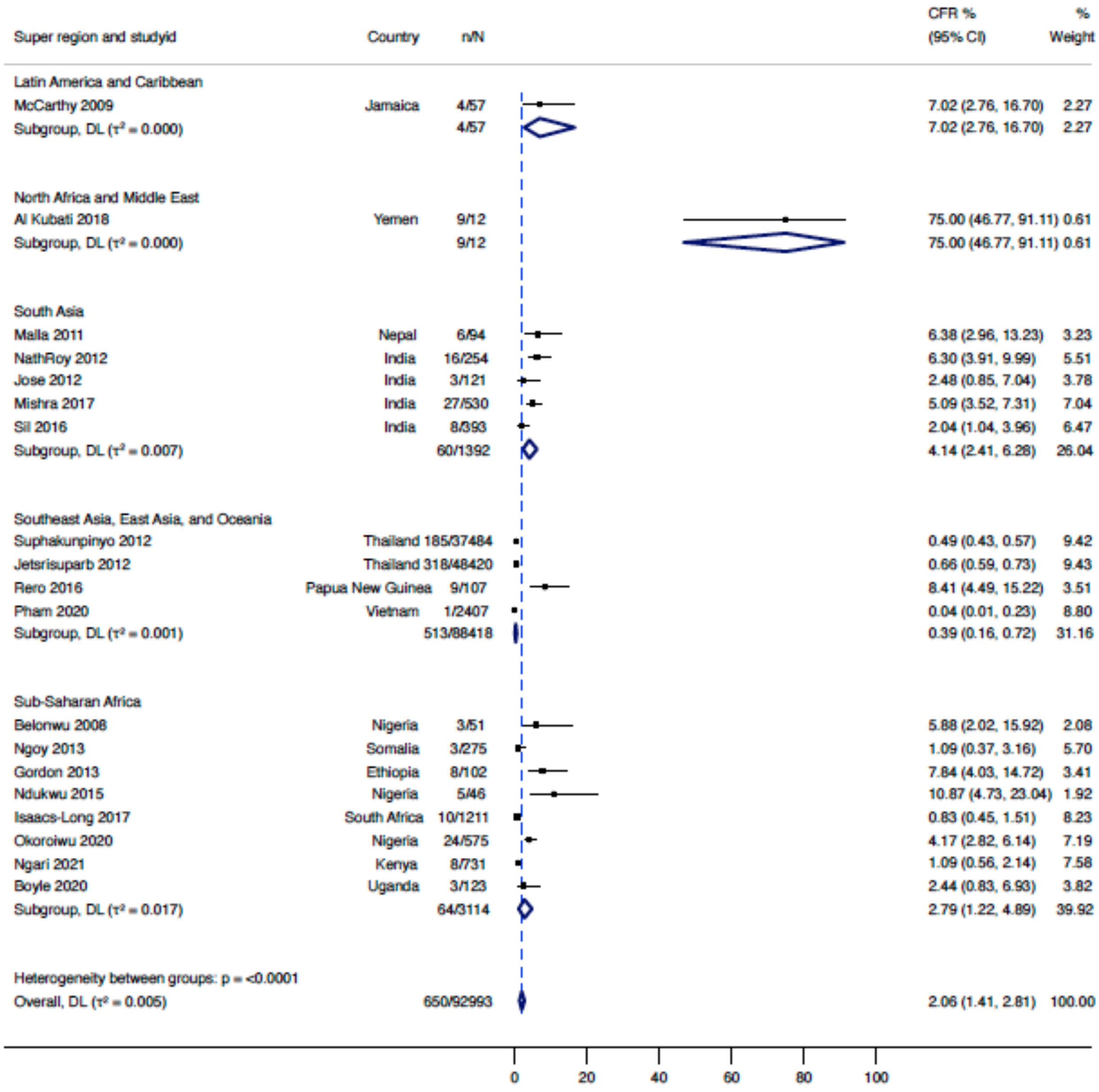

Supplementary Figure 5.9. Forest plot of case fatality rates (CFR): System Trauma-CFR

Super and Study ID	region Country	n/N	Prevalence per 1000 (95% CI)	% Weight
Latin America and Caribbean				
Chua 2014	Guatemala	2/3885	0.51 (0.14, 1.88)	4.76
Subgroup, DL (τ^2 = 0.000)		2/3885	0.51 (0.14, 1.88)	4.76
North Africa and Middle East				
ElMhamdi 2015	Tunisia	4556/52443	86.88 (84.49, 89.32)	4.80
Al Kubati 2018	Yemen	5/8967	0.56 (0.24, 1.30)	4.78
Subgroup, DL (τ^2 = 0.151)		4561/61410	25.99 (0.00, 174.51)	9.58
South Asia				
Ashraf 2016	India	18/28114	0.64 (0.41, 1.01)	4.79
Sonowal 2019	India	3/3808	0.79 (0.27, 2.31)	4.76
Zaheer 2009	Pakistan	122/6089	20.04 (16.81, 23.87)	4.77
van Deursen 2019	Pakistan	18/2551	7.06 (4.47, 11.13)	4.73
van Deursen 2019	Pakistan	7/2663	2.63 (1.27, 5.42)	4.74
Subgroup, DL (τ^2 = 0.012)		168/43225	4.39 (0.22, 13.35)	23.80
Southeast Asia, East Asia, and Oceania				
Jetsrisuparb 2012	Thailand	1849/486845	3.80 (3.63, 3.97)	4.80
Suphakunpinyo 2012	Thailand	3621/332234	10.90 (10.55, 11.26)	4.80
Nguyen 2017	Vietnam	12189/199827	61.00 (59.96, 62.06)	4.80
Pham 2020	Vietnam	1414/113999	12.40 (11.78, 13.06)	4.80
Subgroup, DL (τ^2 = 0.026)		19073/1132905	17.28 (2.82, 43.70)	19.20
Sub-Saharan Africa				
Mola 2016	Ethiopia	139/14521	9.57 (8.11, 11.29)	4.79
Adadey 2019	Ghana	198/36892	5.37 (4.67, 6.17)	4.80
Ngari 2021	Kenya	46/3907	11.77 (8.84, 15.67)	4.76
Nyaga 2010	Kenya	1/12000	0.08 (0.01, 0.47)	4.79
Adeboye 2010	Nigeria	1/606	1.65 (0.29, 9.29)	4.54
George 2010	Nigeria	5/2009	2.49 (1.06, 5.81)	4.72
Muoneke 2016	Nigeria	3/1780	1.69 (0.57, 4.94)	4.71
Okoroiwu 2020	Nigeria	330/14370	22.96 (20.64, 25.54)	4.79
Isaacs-Long 2017	South Africa	331/21751	15.22 (13.67, 16.93)	4.79
Subgroup, DL (τ^2 = 0.008)		1054/107836	6.23 (2.33, 11.92)	42.67
Heterogeneity between groups: p = 0.002				
Overall, DL (τ^2 = 0.028)		24858/1349261	8.33 (3.00, 16.22)	100.00

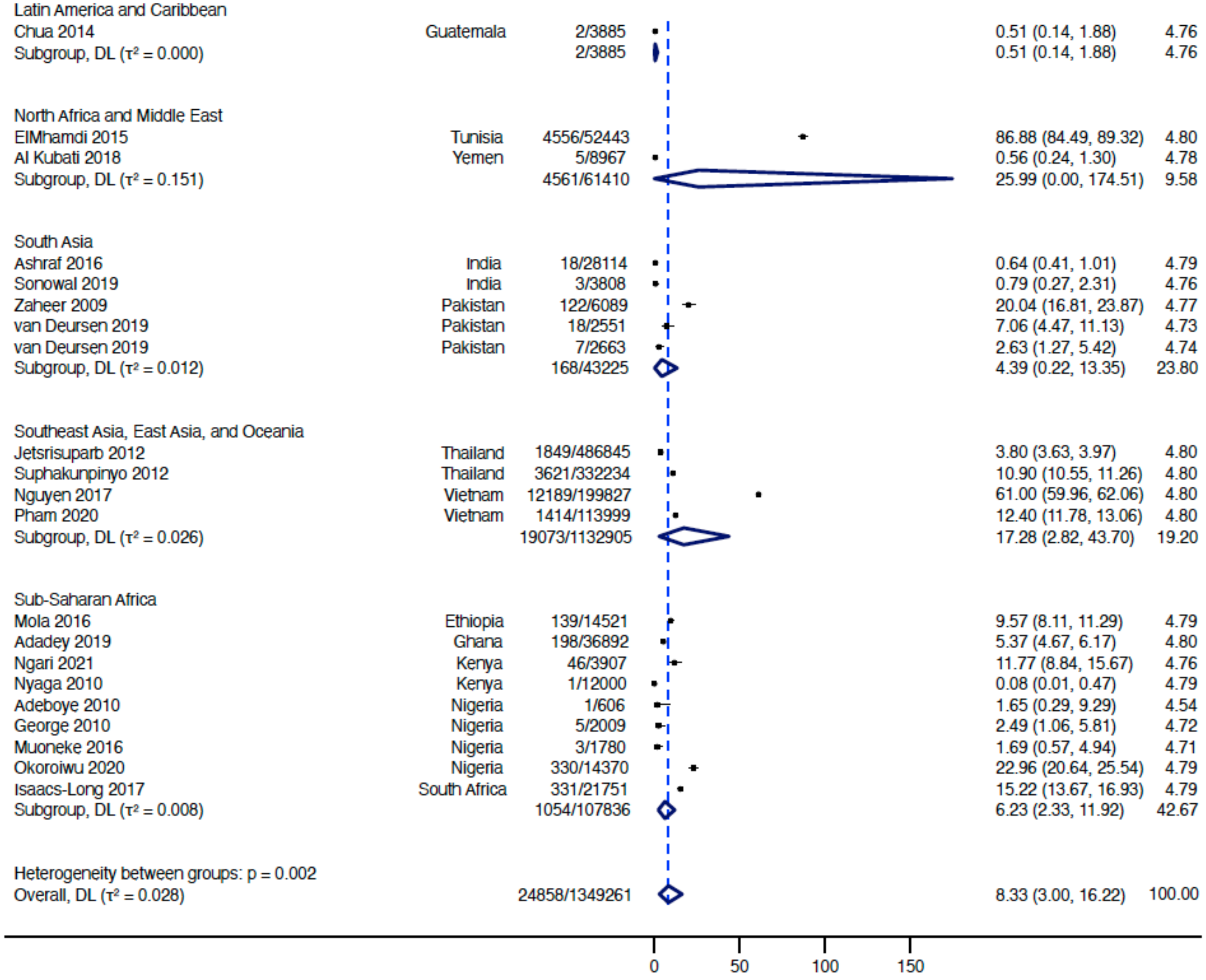

Supplementary Figure 6. Forest Plot for Causes of Hospital Admission: System Congenital anomalies

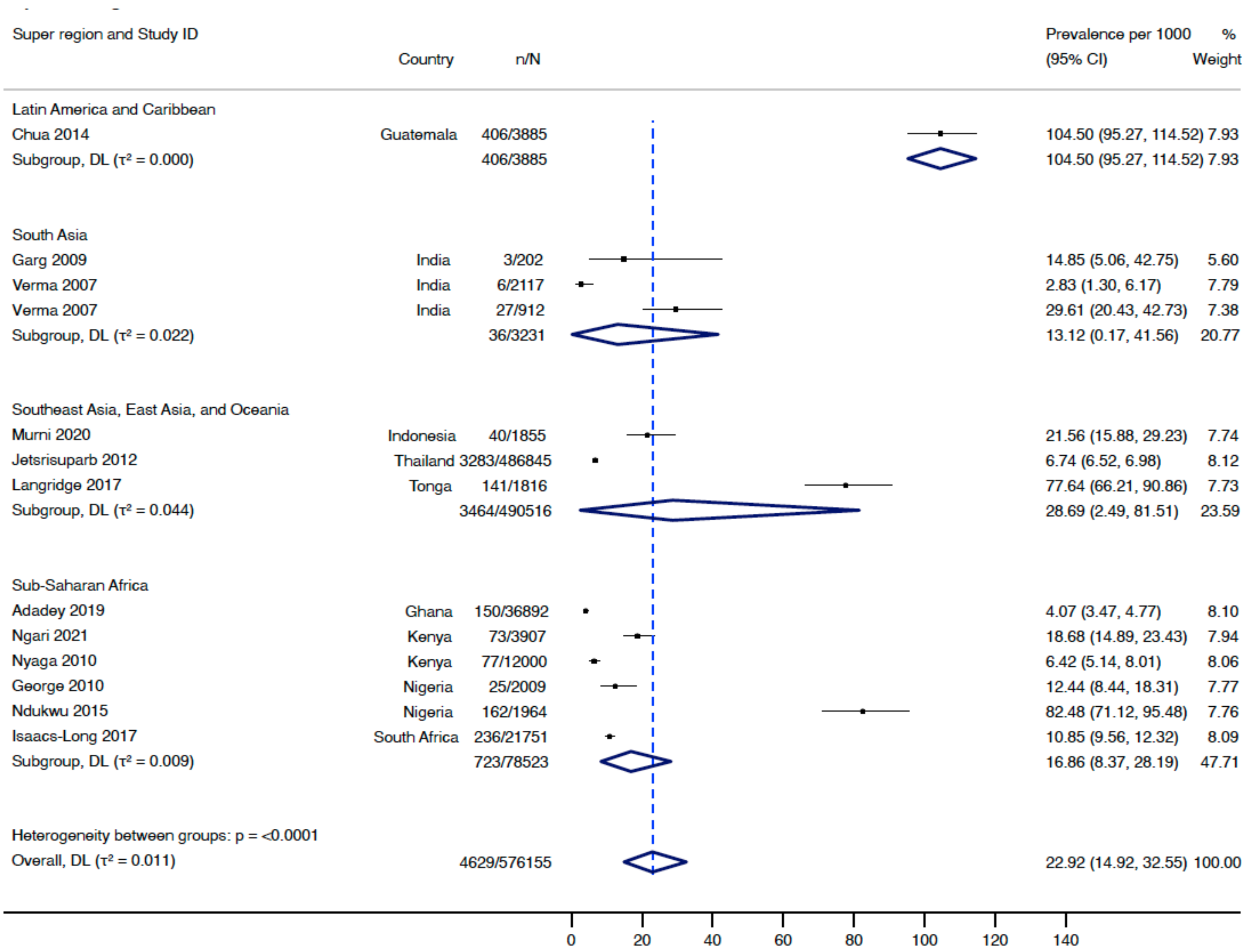

Supplementary Figure 6.1. Forest Plot for Causes of Hospital Admission: System Surgical conditions

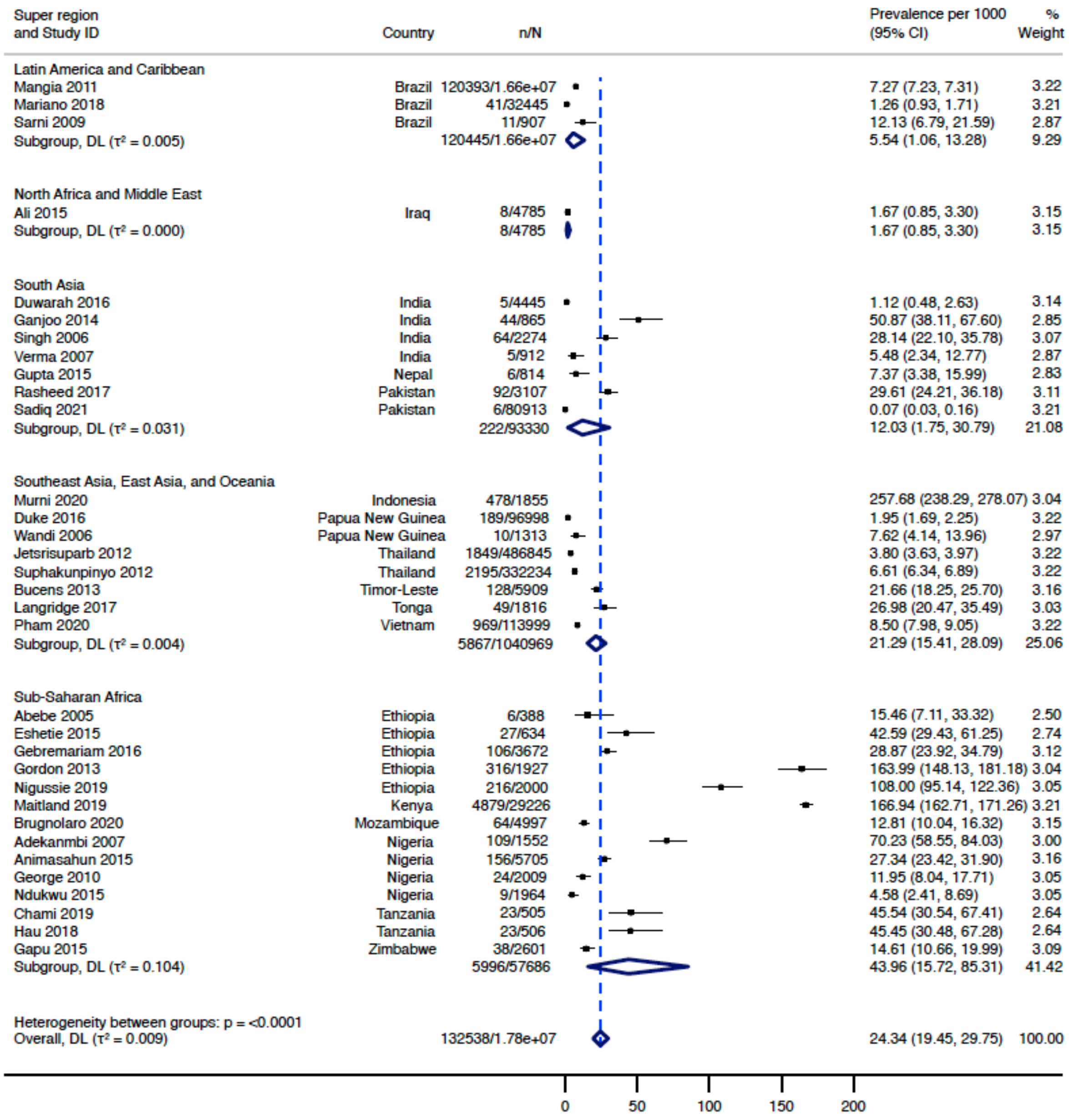

Supplementary Figure 6.2. Forest Plot for Causes of Hospital Admission: System Cardiovascular

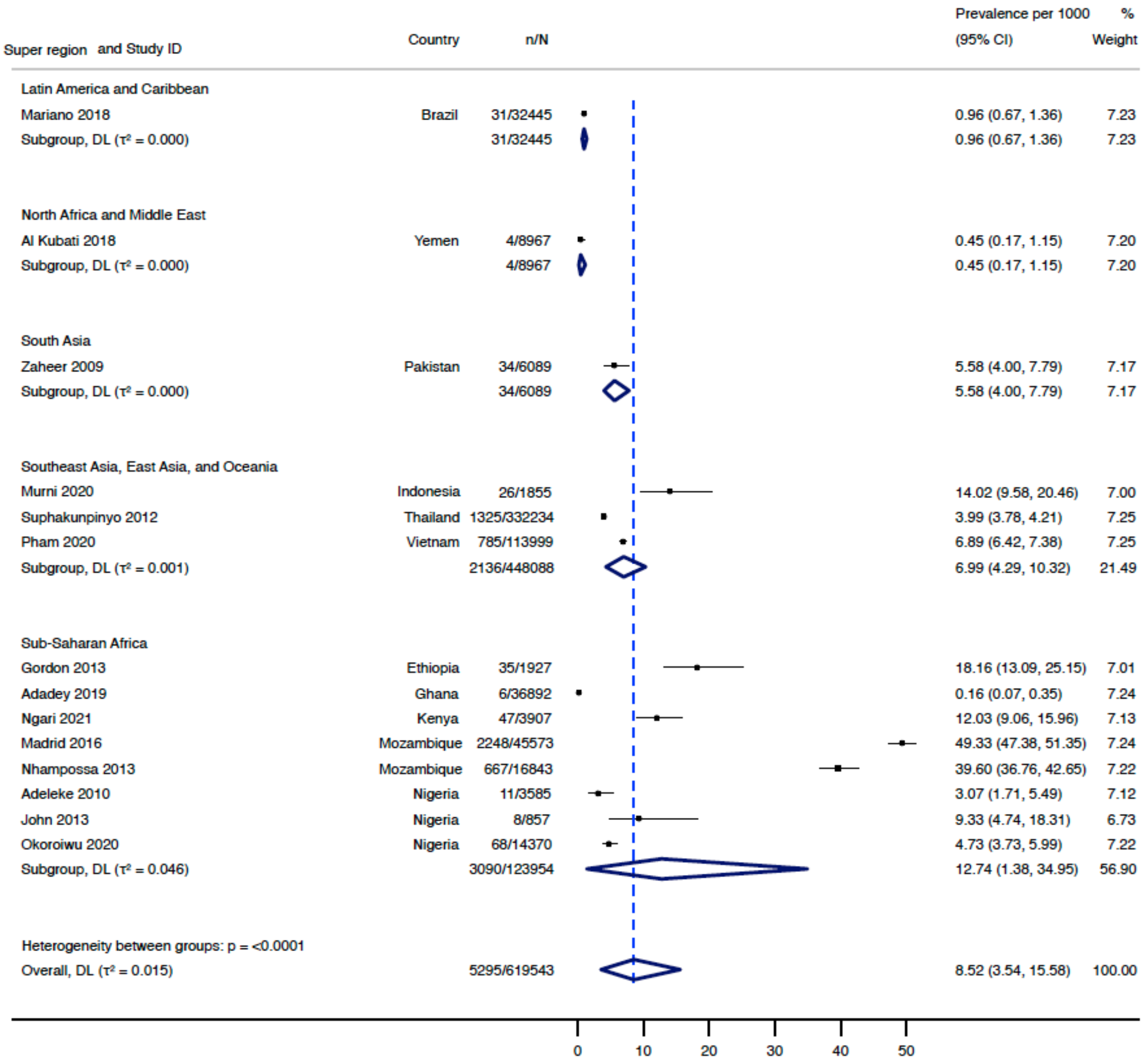

Supplementary Figure 6.3. Forest Plot for Causes of Hospital Admission: System Endocrine

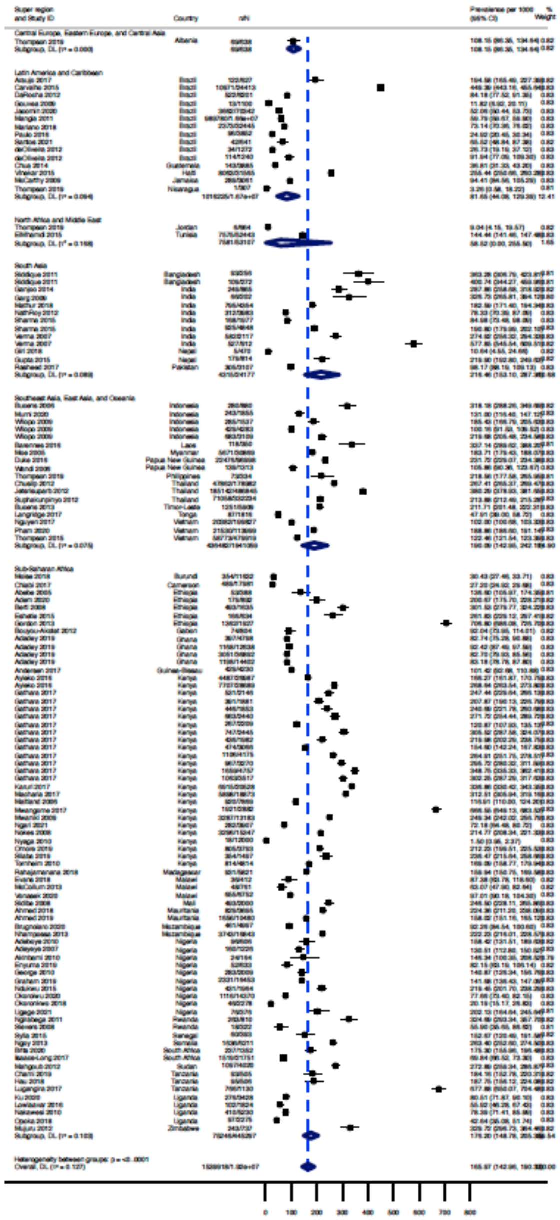

Supplementary Figure 6.4 Forest Plot for Causes of Hospital Admission: System Gastrointestinal

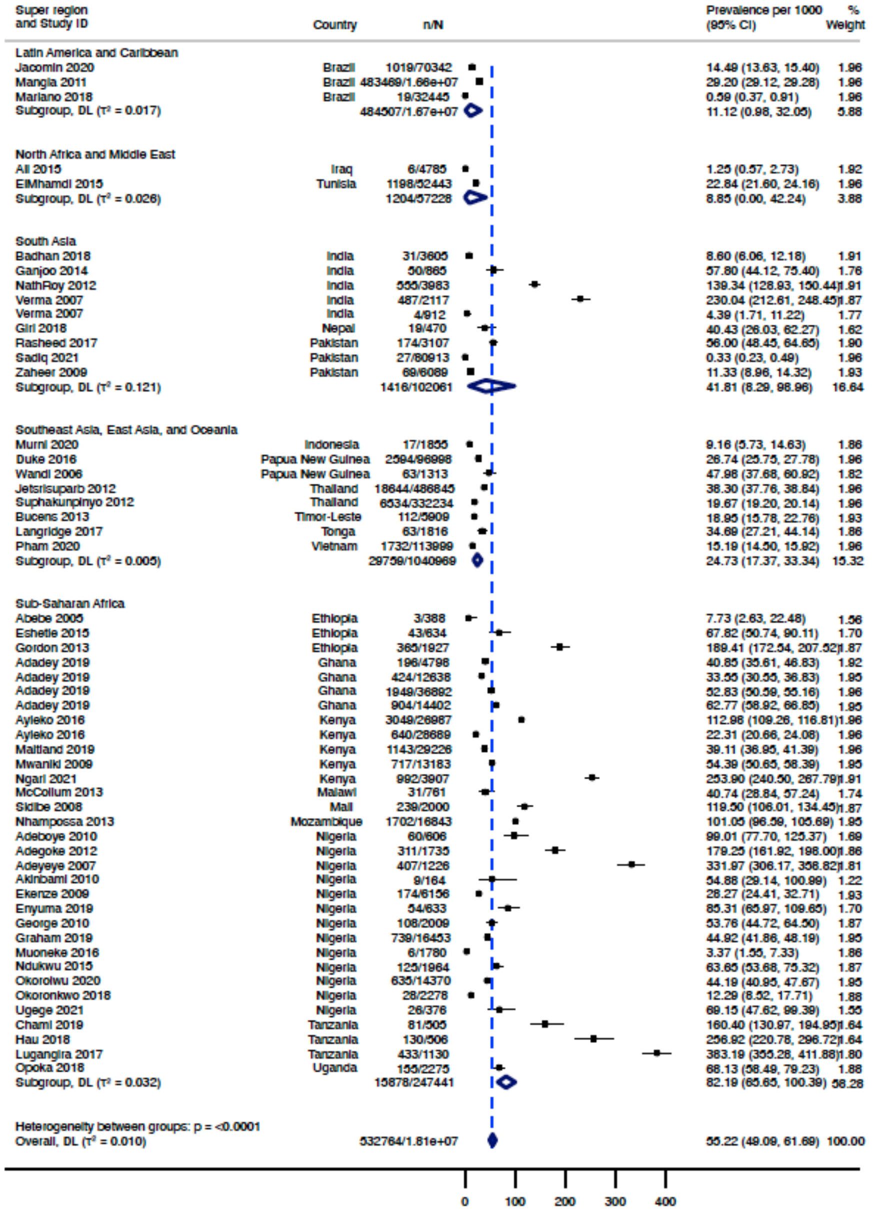

Supplementary Figure 6.5. Forest Plot for Causes of Hospital Admission: System Hematologic

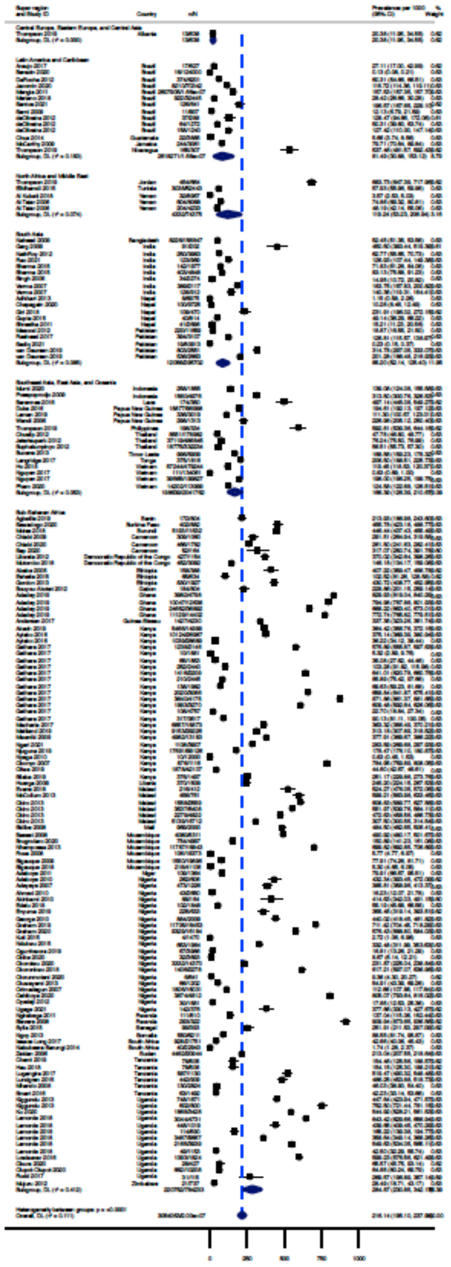

Supplementary Figure 6.6. Forest Plot for Causes of Hospital Admission: System Infection

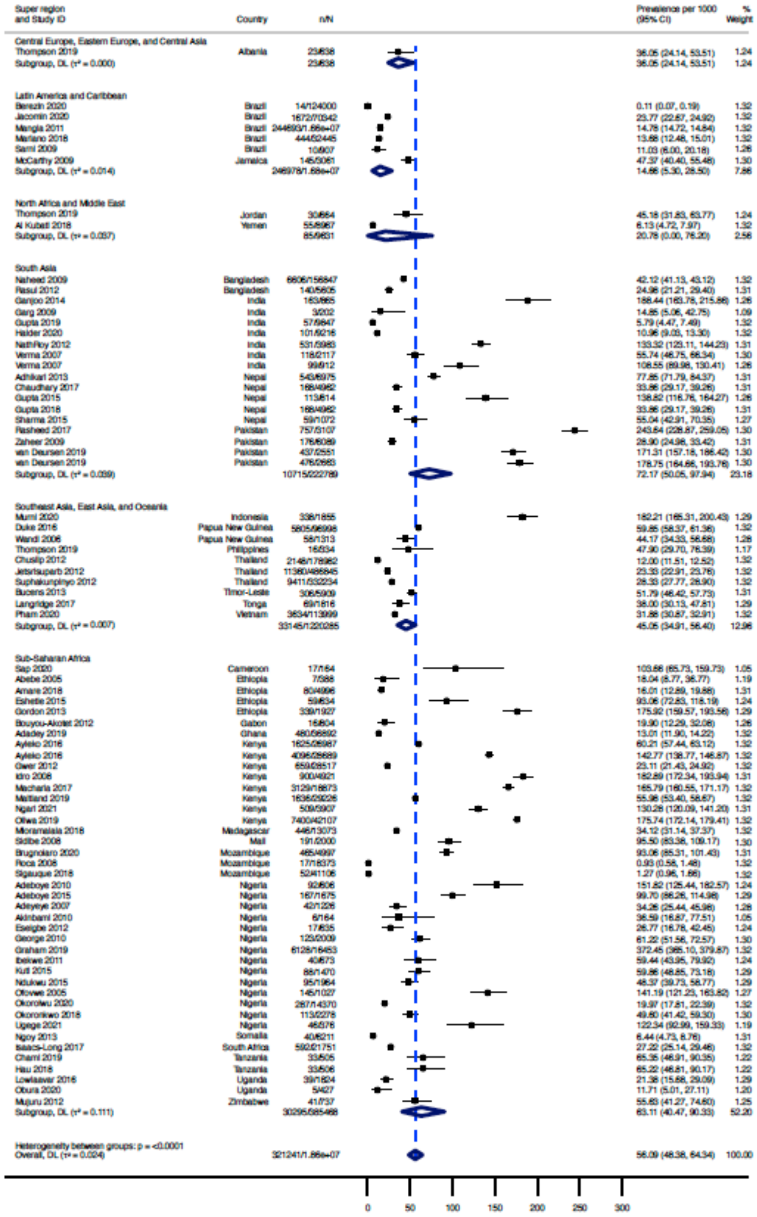

Supplementary Figure 6.7. Forest Plot for Causes of Hospital Admission: System Neurological

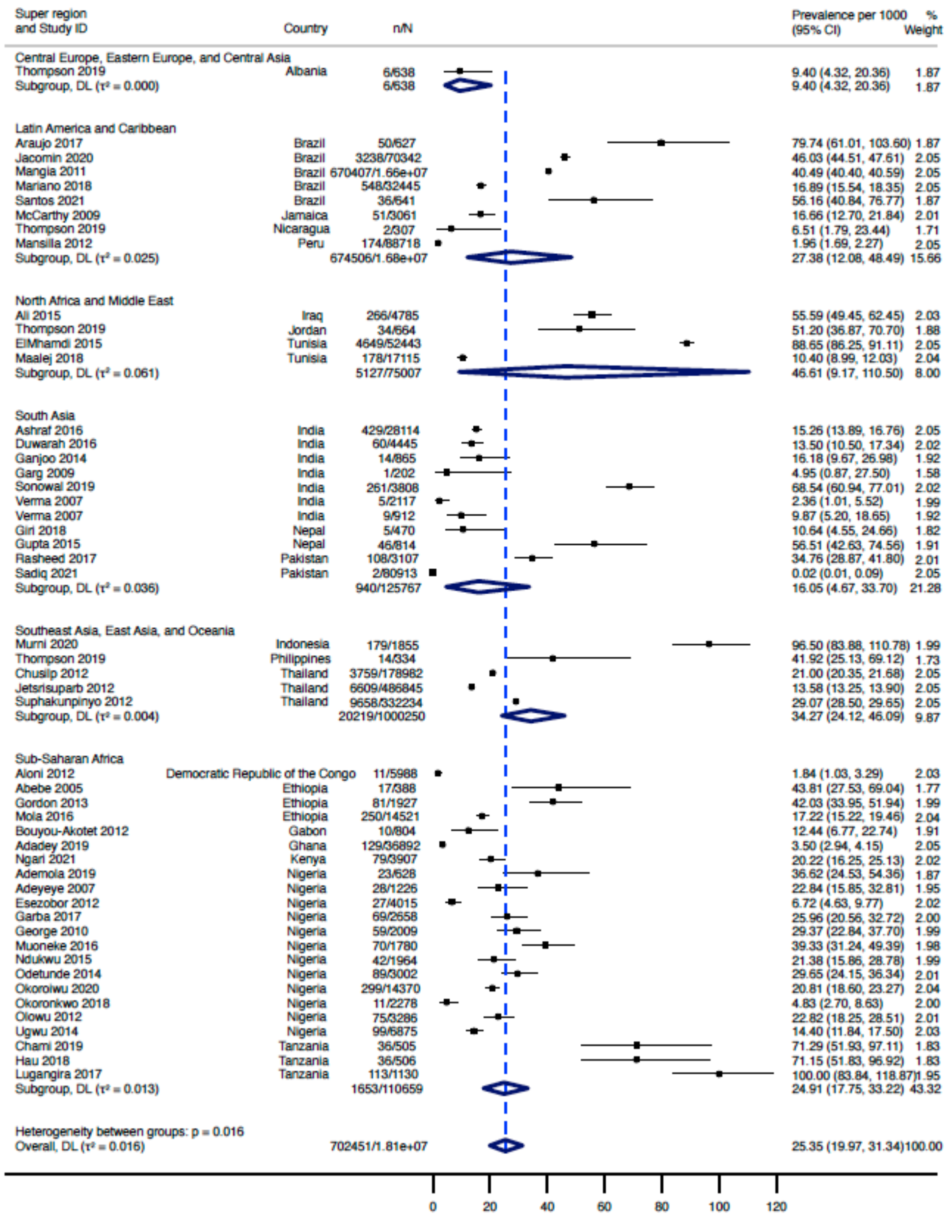

Supplementary Figure 6.8. Forest Plot for Causes of Hospital Admission: System Renal

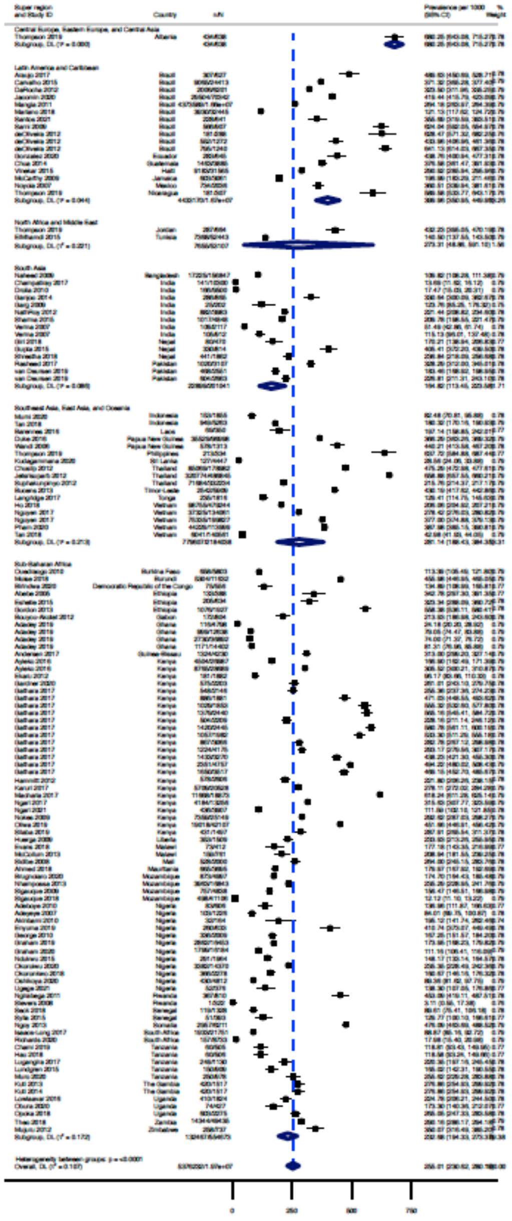

Supplementary Figure 6.9. Forest Plot for Causes of Hospital Admission: System Respiratory

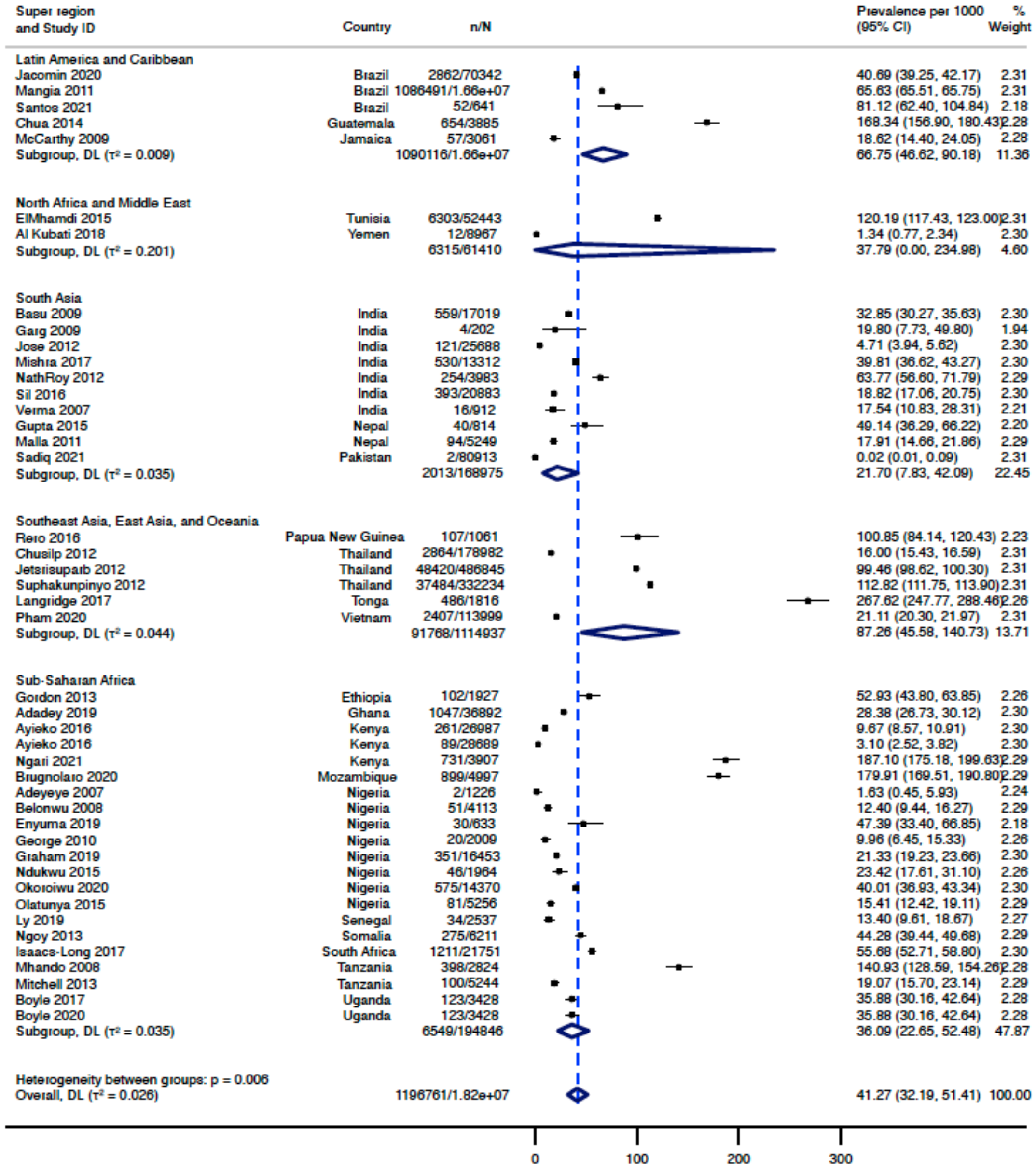

Supplementary Figure 6.10. Forest Plot for Causes of Hospital Admission: System Trauma

Appendix 1 . Protocol modifications

1) We originally stated that we would report outcomes by age; however, not all studies reported outcomes by age and for those that did, there was great heterogeneity between studies in age categories reported.
2) The vast majority of paediatric studies included either subjects <28 days and/or subjects older than 12 years. As such, we decided to include eligible studies with participants outside the 28 day to 12-year age range if participants were <18 years and >50% of participants were within the age range of interest.
3) We originally intended to report causes of hospital admission as “critical” and “non-critical”, unfortunately, an appropriate definition for critical illness in this context does not exist.
4) We originally stated that we would perform meta-regression to explore predictors for all-cause and cause-specific mortality. While not included in this publication, it may be included in a future publication from these data.
5) We originally stated that we would explore temporal trends in admission and mortality by age and region. While not included in this publication, it may be included in a future publication from these data.
6) We originally stated that we would examine sources of heterogeneity, including differences in methodology, setting (urban vs. rural), region, and patient population. Unfortunately, due to missing and unavailable data, we were unable to explore these potential sources of heterogeneity.
7) Risk of bias due to missing results (arising from reporting biases) could not be assessed given the available data in the included studies.
8) Included studies with a high proportion of reported neonatal mortality were included in all analyses except for the all-cause mortality estimate.

Appendix 2. List of excluded studies at full-text screening (N=2003)

Duplicate texts (N=116)

1. Abd Elmoneim A, Hassan IA, Abd EA, Elmagd AA. Epidemiology and outcome of childhood asthma: a clinical study in an Egyptian university medical centre. East Mediterr Health J 2013; 19(6): 520-6.
2. Adam S, Sama DH, Mouzou T, Akakpovi A, Tomta K, Bellier M. Problématique de la prise en charge des urgences en Afrique subsaharienne : exemple du service d'accueil des urgences chirurgicales (SAUC) du CHU Sylvanus Olympio de Lomé, Togo. Medecine et sante tropicales 2018; 28(3): 327-30.
3. Adeboye M, Ojuawo A, Adeniyi A, Ibraheem RM, Amiwero C. Febrile Convulsion among Hospitalized Children Aged Six Months to Five Years and Its Association With Haemoglobin Electrophoretic Pattern. 2015; 1(3): 251-6.
4. Ahmed BW, Hanoudi BM, Ibrahim BA. Risk factors in children with febrile seizures and their iron status. JPMA The Journal of the Pakistan Medical Association 2019; 69 3)(8): S22-S5.
5. Aiken AM, Mturi N, Njuguna P, et al. Risk and causes of paediatric hospital-acquired bacteraemia in Kilifi District Hospital, Kenya: A prospective cohort study. The Lancet 2011; 378(9808): 2021-7.
6. Akech S, Chepkirui M, Ogero M, et al. The clinical profile of severe paediatric malaria in an area targeted for routine RTS,S/AS01 malaria vaccination in Western Kenya. Clin Infect Dis 2019; 26: 26.
7. Alievi PcT, Carvalho PRA, Trotta EA, Mombelli Filho R. Impacto da internação em unidade de terapia intensiva pediátrica: avaliação por meio de escalas de desempenho cognitivo e global. J pediatr (Rio J) 2007; 83(6): 505-11.
8. Alrifai SB, Alsaadi A, Mahmood YA, Ali AA, Al-Kaisi LA. Prevalence and etiology of nosocomial diarrhoea in children < 5 years in Tikrit teaching hospital. East Mediterr Health J 2009; 15(5): 1111-8.
9. Alvis-Guzman N, Orozco-Africano J, Paternina-Caicedo A, et al. Treatment costs of diarrheal disease and all-cause pneumonia among children under-5 years of age in Colombia. Vaccine 2013; 31 Suppl 3: C58-62.
10. Antunes FP, Costa MCN, Paim JS, et al. Trends in hospitalizations for respiratory diseases in Salvador, Bahia state, Brazil, 1998-2009. Cadernos de Saude Publica 2012; 28(5): 869-77.
11. Arul GS, Reynolds J, DiRusso S, et al. Paediatric admissions to the British military hospital at Camp Bastion, Afghanistan. Annals of the Royal College of Surgeons of England 2012; 94(1): 52-7.
12. Atiq H, Siddiqui E, Bano S, et al. The pediatric disease spectrum in emergency departments across Pakistan: Data from a pilot surveillance system. BMC Emergency Medicine 2015; 15(2).
13. Bahloul M, Chelly H, Gargouri R, et al. Traumatic head injury in children in south Tunisia epidemiology, clinical manifestations and evolution. 454 cases. Tunis Med 2009; 87(1): 28-37.
14. Barreto JOM, Nery IS, Costa MSC. The Family Health Strategy and hospital admissions of children under five years in Piauí- State, Brazil. Cadernos de Saude Publica 2012; 28(3): 515-26.
15. Bar-On E, Lebel E, Blumberg N, Sagi R, Kreiss Y. Pediatric orthopedic injuries following an earthquake: Experience in an acute-phase field hospital. Journal of Trauma and Acute Care Surgery 2013; 74(2): 617-21.
16. Brent AJ, Ahmed I, Ndiritu M, et al. Incidence of clinically significant bacteraemia in children who present to hospital in Kenya: community-based observational study. Lancet 2006; 367(9509): 482-8.
17. Buys H, Muloiwa R, Westwood C, Richardson D, Cheema B, Westwood A. An adapted triage tool (ETAT) at Red Cross War Memorial Children's Hospital Medical Emergency Unit, Cape Town: an evaluation. Samj, S 2012; 103(3): 161-5.
18. Caballero A, Pinilla MI, Mendoza IC, Pena JR. Hospital readmission rate and associated factors among health services enrollees in Colombia. Cad Saude Publica 2016; 32(7): 21.
19. Carvalho SC, Mota E, Dourado I, Aquino R, Teles C, Medina MG. Hospitalizations of children due to primary health care sensitive conditions in Pernambuco State, Northeast Brazil. 2015; 1(4): 744-54.
20. Cavalini LT, Ponce de Leon AC. Correction approach for underreporting of deaths and hospital admissions due to ill-defined causes. [Portuguese]. 2007; 1(1): 85-93.
21. Ceesay SJ, Casals-Pascual C, Erskine J, et al. Changes in malaria indices between 1999 and 2007 in The Gambia: a retrospective analysis. Lancet 2008; 372 North American Edition(9649): 1545-54.
22. Collins J, Biaukula V, Faktaufon D, et al. An outbreak investigation of paediatric severe acute respiratory infections requiring admission to intensive care units - Fiji, May 2016. Western pac 2018; 9(2): 4-8.
23. Das MK, Arora NK, Poluru R, et al. Pediatric Appropriate Evaluation Protocol for India (PAEP-India): Tool for Assessing Appropriateness of Pediatric Hospitalization. 2018; 1(12): 1041-5.
24. De Barros ENC, Nunes AA, Abreu ADJLD, et al. Pertussis epidemiological pattern and disease burden in Brazil: an analysis of national public health surveillance data. Human Vaccines and Immunotherapeutics 2020; 16(1): 61-9.
25. Tendência temporal das Internações por Condições Sensíveis à Atenção Primária em crianças menores de cinco anos de idade no Ceará, 2000 a 2012. Epidemiol. serv. Saúde 2017; 26(1): 51-60.
26. Couto TB, Farhat SC, Reid T, Schvartsman C. Mortality in a pediatric secondary-care hospital in post-conflict Liberia in 2009. Einstein 2013; 11(4): 413-20.

27. Derby KS, Lucien MA, Leshem E, et al. Hospitalizations and deaths caused by diarrhea in children five years old and younger at four hospitals in Haiti, 2010-2012. Am J Trop Med Hyg 2014; 90(2): 291-3.
28. Duek OS, Ben Naftali Y, Pikkel YY, et al. Coffee break - Epidemiologic analysis, traditions & holidays that increase burn risk of pediatric patients. Burns 2020; 31: 31.
29. Eck C, Pierre RB, Hambleton IR. Medical paediatric admission patterns at the University Hospital of the West Indies: issues for future planning. West Indian med j 2006; 55(5): 340-5.
30. El Desoky S, Mashat S, Bana S, et al. Efficiency of Using Pediatrics Emergency Services and Triage Evaluation. Pediatr Emerg Care 2016; 02: 02.
31. El-Sherbini SA, Marzouk H, El-Sayed R, Hosam-ElDin S. Etiology of hyperglycemia in critically ill children and the impact of organ dysfunction. Rev 2018; 30(3): 286-93.
32. Elorza Parra M, Escobar González AsF, Cornejo Ochoa W, Quevedo Vélez A. Morbimortalidad en la unidad de cuidados intensivos pediátricos del Hospital Universitario San Vicente de Paúl, Medellín, Colombia, 2001-2005. Iatreia 2008; 21(1): 33-40.
33. Esparza-Aguilar M, Gastañaduy PA, Sánchez-Uribe E, et al. Diarrhoea-related hospitalizations in children before and after implementation of monovalent rotavirus vaccination in Mexico. Bulletin of the World Health Organization 2014; 92(2): 117-25.
34. Etuk IS, Anah MU, Eyong ME. Epidemiology and clinical features of acute glomerulonephritis in Calabar, Nigeria. Nigerian Journal of Physiological Sciences 2009; 24(2): 91-4.
35. Farias YN, Leite IDC, Siqueira M, Cardoso AM. Ethnic and racial inequalities in hospital admissions due to avoidable causes in under-five Brazilian children, 2009-2014. Cad Saude Publica 2019; 35Suppl 3(Suppl 3): e00001019.
36. Farhat SC, Paulo RL, Shimoda TM, et al. Effect of air pollution on pediatric respiratory emergency room visits and hospital admissions. 2005; 1(2): 227-35.
37. Feikin DR, Jagero G, Aura B, et al. High rate of pneumococcal bacteremia in a prospective cohort of older children and adults in an area of high HIV prevalence in rural western Kenya. BMC Infectious Diseases 2009; 10.
38. Fiestas Solorzano V, Sihuincha Maldonado M, Donaires Toscano F, et al. Clinical characteristics of patients admitted to hospital "Cesar Garayar Garcia", Iquitos during the dengue epidemic, January-February 2011. Rev 2011; 28(1): 78-82.
39. Franca JC, Ribeiro CE, Queiroz-Telles F. Candidemia in a Brazilian tertiary care hospital: incidence, frequency of different species, risk factors and antifungal susceptibility. [Portuguese]. 2008; 1(1): 23-8.
40. Freire KMS, Bresolin NL, Farah ACF, Carvalho FLgC, Góes JEC. Lesão renal aguda em crianças: incidência e fatores prognósticos em pacientes gravemente enfermos. Rev bras ter intensiva 2010; 22(2): 166-74.
41. Goncalez TT, Sabino EC, Capuani L, et al. Blood transfusion utilization and recipient survival at Hospital das Clinicas in Sao Paulo, Brazil. Transfusion 2012; 52(4): 729-38.
42. Graham H, Bakare AA, Ayede AI, et al. Diagnosis of pneumonia and malaria in Nigerian hospitals: A prospective cohort study. Pediatric Pulmonology 2020; 55(S1): S37-S50.
43. Grando LR, Schmitt TAB, Bakos RM. Severe cutaneous reactions to drugs in the setting of a general hospital. Anais Brasileiros de Dermatologia 2014; 89(5): 758-62.
44. Grellety E, Krause LK, Shams Eldin M, Porten K, Isanaka S. Comparison of weight-for-height and mid-upper arm circumference (MUAC) in a therapeutic feeding programme in South Sudan: is MUAC alone a sufficient criterion for admission of children at high risk of mortality? Public health nutrition 2015; 18(14): 2575-81.
45. Gulati S, Kalra V, Sridhar MR. Status epilepticus in Indian children in a tertiary care center. Indian Journal of Pediatrics 2005; 72(2): 105-8.
46. Hammitt LL, Kazungu S, Morpeth SC, et al. A preliminary study of pneumonia etiology among hospitalized children in Kenya. Clinical Infectious Diseases 2012; 54(SUPPL. 2): S190-S9.
47. Hinrichsen SL, Falcao E, Vilella TAS, et al. Candidemia in a tertiary hospital in northeastern Brazil. Revista da Sociedade Brasileira de Medicina Tropical 2008; 41(4): 394-8.
48. Hossain MI, Dodd NS, Ahmed T, et al. Experience in managing severe malnutrition in a government tertiary treatment facility in Bangladesh. Journal of health, population, and nutrition 2009; 27(1): 72-9.
49. Hyginus EO, Okechukwu UJ, I MV, Christian OC, Anthony U. I Epidemiology of admitted cases of childhood injuries in Nnamdi Azikiwe University Teaching Hospital Nnewi, Nigeria. Annals of Tropical Medicine & Public Health 2015; 8(6): 272-5.
50. Ilechie AA, Essuman VA, Enyionam S. Prevalence of congenital eye anomalies in a paediatric clinic in Ghana. East Mediterr Health J 2014; 19 Suppl 3: S76-80.
51. Jiraphongsa C, Bresee JS, Pongsuwanna Y, et al. Epidemiology and burden of rotavirus diarrhea in Thailand: results of sentinel surveillance. J Infect Dis 2005; 192 Suppl 1: S87-93.
52. Jofiro G, Jemal K, Beza L, Bacha Heye T. Prevalence and associated factors of pediatric emergency mortality at Tikur Anbessa specialized tertiary hospital: a 5 year retrospective case review study. BMC Pediatr 2018; 18(1): 316.
53. Kakembo N, Grabski DF, Fitzgerald TN, et al. Burden of Surgical Infections in a Tertiary-Care Pediatric Surgery Service in Uganda. Surg Infect (Larchmt) 2020; 21(2): 130-5.

54. Kapesa A, Kweka EJ, Atieli H, et al. The current malaria morbidity and mortality in different transmission settings in western Kenya. PLoS ONE 2018; 13(8).
55. Khagayi S, Burton DC, Onkoba R, et al. High burden of rotavirus gastroenteritis in young children in rural western Kenya, 2010-2011. Pediatr Infect Dis J 2014; 33 Suppl 1: S34-40.
56. Khan N, Perez-Nunez R, Shamim N, et al. Intentional and unintentional poisoning in Pakistan: a pilot study using the Emergency Departments surveillance project. BMC emerg 2015; 15 Suppl 2: S2.
57. Le NK, Hf W, Vu PD, et al. High prevalence of hospital-acquired infections caused by gram-negative carbapenem resistant strains in Vietnamese pediatric ICUs. Medicine (United States) 2016; 95(27).
58. Lee CH, Won YK, Roh EJ, Suh DI, Chung EH. A nationwide study of children and adolescents with pneumonia who visited South Korea Emergency Department in 2012. Korean Journal of Pediatrics 2016; 59(3): 132-8.
59. Magalhães ALA, Morais OL. Intra-urban differences in rates of admissions for ambulatory care sensitive conditions in Brazil's Center-West region. Ciencia & saude coletiva 2017; 22(6): 2049-62.
60. Mahajan V, Kaur A, Sharma A, Azad C, Guglani V. Modifiable factors for prevention of childhood mortality. 2014; 1(1): 45-7.
61. Mançaneira JF, Benedetti JR, Zhang L. Hospitalizations and deaths due to pertussis in children from 1996 to 2013&#9734. J pediatr (Rio J) 2016; 92(1): 40-5.
62. Mapaseka SL, Dewar JB, van der Merwe L, et al. Prospective hospital-based surveillance to estimate rotavirus disease burden in the Gauteng and North West Province of South Africa during 2003-2005. J Infect Dis 2010; 202 Suppl: S131-8.
63. Mariano TdSO, Nedel FlB. Hospitalização por Condições Sensíveis à Atenção Primária em menores de cinco anos de idade em Santa Catarina, 2012: estudo descritivo. Epidemiol. serv. saúde 2018; 27(3): e2017322-e.
64. Martins CBDG, De Andrade SM. Accidents with foreign bodies in children under 15 years of age: Epidemiological analysis of first aid services, hospitalizations, and deaths. Cadernos de Saude Publica 2008; 24(9): 1983-90.
65. Mathew A, Rao PS, Sowmyanarayanan TV, Kang G. Severity of rotavirus gastroenteritis in an Indian population: report from a 3 year surveillance study. Vaccine 2014; 32 Suppl 1: A45-8.
66. Matijasevich A, Cesar JA, Santos IS, et al. Hospitalizations during infancy in three population-based studies in Southern Brazil: Trends and differentials. Cadernos de Saude Publica 2008; 24(SUPPL.3): S437-S43.
67. Mehta P, Sinha A, Sami A, et al. Incidence of acute kidney injury in hospitalized children. Indian Pediatrics 2012; 49(7): 537-42.
68. Menezes AMB, Noal RB, Cesar JA, et al. Hospital admissions from birth to early adolescence and early-life risk factors: The 11-year follow-up of the 1993 Pelotas (Brazil) birth cohort study. Cadernos de Saude Publica 2010; 26(10): 1980-9.
69. Menif K, Bouziri A, Khaldi A, et al. Community-acquired methicillin-resistant Staphylococcus aureus infections requiring admission to a Tunisian pediatric intensive care unit. Tunis Med 2011; 89(10): 758-
70. Moe K, Hummelman EG, Oo WM, Lwin T, Htwe TT. Hospital-based surveillance for rotavirus diarrhea in children in Yangon, Myanmar. J Infect Dis 2005; 192 Suppl 1: S111-3.
71. Mukhtar B, Siddiqui NR, Haque A. Clinical characteristics and immediate-outcome of children mechanically ventilated in a pediatric intensive care units. Pakistan Journal of Medical Sciences 2014; 30(5).
72. Munoz Esparza NC, Vasquez-Garibay EM, Larrosa-Haro A, Romero-Velarde E. Socio-demographic variables and underlying pathologies associated to nutritional status of hospitalized children in a secondary-tertiary level hospital. Nutr Hosp 2018; 35(2): 286-93.
73. Muoneke VU, Ibekwe RC, Nebe-Agumadu HU, Ibe BC. Factors associated with mortality in under-five children with severe anemia in Ebonyi, Nigeria. Indian Pediatrics 2012; 49(2): 119-23.
74. Mwaniki MK, Nokes DJ, Ignas J, et al. Emergency triage assessment for hypoxaemia in neonates and young children in a Kenyan hospital: an observational study. 2009; 1(4): 263-70.
75. Naorat S, Chittaganpitch M, Thamthitiwat S, et al. Hospitalizations for acute lower respiratory tract infection due to respiratory syncytial virus in Thailand, 2008-2011. Journal of Infectious Diseases 2013; 208(SUPPL. 3): S238-S45.
76. Nascimento MS, de Souza AV, Ferreira AVS, Rodrigues JC, Abramovici S, Filho LVFS. High rate of viral identification and coinfections in infants with acute bronchiolitis. Clinics 2010; 65(11): 1133-7.
77. Nhampossa T, Sigauque B, Machevo S, et al. Severe malnutrition among children under the age of 5 years admitted to a rural district hospital in southern Mozambique. 2013; 1(9): 1565-74.
78. Noblat AC, Noblat LA, Toledo LA, et al. Prevalence of hospital admission due to adverse drug reaction in Salvador, Bahia. Rev Assoc Med Bras 2011; 57(1): 42-5.
79. Novaes HMD, Sartori AMC, de Soarez PC. Hospitalization rates for pneumococcal disease in Brazil, 2004 - 2006. Revista de Saude Publica 2011; 45(3): 539-47.
80. Nunthanid S, Tiawilai A. Dengue Patients at Photharam Hospital: A Clinical Trial Site of Dengue Vaccine. Southeast Asian J Trop Med Public Health 2015; 46 Suppl 1: 11-6.

81. Nupen TL, Argent AC, Morrow B. Characteristics and outcome of long-stay patients in a paediatric intensive care unit in Cape Town, South Africa. Samj, S 2016; 107(1): 70-5.
82. O'Meara WP, Bejon P, Mwangi TW, et al. Effect of a fall in malaria transmission on morbidity and mortality in Kilifi, Kenya. Lancet 2008; 372 North American Edition(9649): 1555-62.
83. de Oliveira BR, Viera CS, Furtado MC, de Mello DF, de Lima RA. Profile of morbidity of children hospitalized in a public hospital: implications for nursing. [Portuguese]. 2012; 1(4): 586-93.
84. de Oliveira RR, Costa JR, Mathias TA. Hospitalization of children under five years of age due to avoidable causes. 2012; 1(1): 135-42.
85. Omore R, Khagayi S, Ogwel B, et al. Rates of hospitalization and death for all-cause and rotavirus acute gastroenteritis before rotavirus vaccine introduction in Kenya, 2010-2013. 2019; 1(1): 47.
86. Onankpa B, Airede L, Paul I, Dorcas I. Pattern of pediatric HIV/AIDS: A five-year experience in a tertiary hospital. Journal of the National Medical Association 2008; 100(7): 821-5.
87. Onyango CO, Njeru R, Kazungu S, et al. Influenza surveillance among children with pneumonia admitted to a district hospital in coastal Kenya, 2007-2010. Journal of Infectious Diseases 2012; 206(SUPPL.1): S61-S7.
88. Páez P, Suárez-Obando F, Zarante I. Enfermedades de origen genético en pacientes pediátricos hospitalizados en la Provincia de Ubate, Colombia. Rev Salud Publica 2008; 10(3): 414-22.
89. Palihawadana P, Kang G, Navaratnasingam J, et al. Rotavirus infection among hospitalized children under five years of age with acute watery diarrhea in Sri Lanka. Vaccine 2018; 36(51): 7846-50.
90. Perry HB, Ross AG, Fernand F. Assessing the causes of under-five mortality in the Albert Schweitzer Hospital service area of rural Haiti. Revista Panamericana de Salud Publica/Pan American Journal of Public Health 2005; 18(3): 178-86.
91. Piñeros JG, Baquero H, Bastidas J, et al. Respiratory syncytial virus infection as a cause of hospitalization in population under 1 year in Colombia. J Pediatr (Rio J) 2013; 89(6): 544-8.
92. Rafai M, Mekkaoui N, Chouaib N, et al. Epidemiology of severe domestic accidents of children admitted in a versatile pediatric intensive care unit at the Children's Hospital of Rabat, Morocco. Pan African Medical Journal 2015; 20: 29-34.
93. Raihana S, Dunsmuir D, Huda T, et al. Erratum: Development and internal validation of a predictive model including pulse oximetry for hospitalization of under-five children in Bangladesh (PLoS ONE (2015) 10:11 (e0143213) DOI: 10.1371/journal.pone.0143213). PLoS ONE 2016; 11(1).
94. Rao S, Gavali V, Prabhu SS, Mathur R, Dabre LR. Outcome of Children Admitted With SARS-CoV-2 Infection: Experiences From a Pediatric Public Hospital. Indian Pediatr 2021; 19: 19.
95. Rathore V, Bansal A, Singhi SC, Singhi P, Muralidharan J. Survival and neurological outcome following in-hospital paediatric cardiopulmonary resuscitation in North India. Paediatr Int Child Health 2016; 03: 03.
96. Rodrigues A, De Carvalho M, Monteiro S, et al. Hospital surveillance of rotavirus infection and nosocomial transmission of rotavirus disease among children in Guinea-Bissau. Pediatric Infectious Disease Journal 2007; 26(3): 233-7.
97. Rodrigues LS, Motta FA, Picharski GL, Vasconcelos TM, Riccieri MC, Dalla-Costa LM. Invasive candidiasis: Risk factor for mortality in a pediatric tertiary care hospital in south of Brazil. 2019; 1(23): e15933.
98. Rodriguez-Martinez CE, Sossa-Briceno MP, Castro-Rodriguez JA. Predictors of hospitalization for asthma in children: results of a 1-year prospective study. Pediatr Pulmonol 2014; 49(11): 1058-64.
99. Rodriguez-Martinez CE, Sossa-Briceno MP, Castro-Rodriguez JA. Direct medical costs of pediatric asthma exacerbations requiring hospital attendance in a middle-income country. Allergol Immunopathol (Madr) 2019; 07: 07.
100. Rosa AM, Ignotti E, Hacon Sde S, Castro HA. Analysis of hospitalizations for respiratory diseases in Tangara da Serra, Brazil. 2008; 1(8): 575-82.
101. Roy SK, Buis M, Weersma R, et al. Risk factors of mortality in severely-malnourished children hospitalized with diarrhoea. Journal of Health, Population and Nutrition 2011; 29(3): 229-35.
102. Saha TK, Bose K, Sorkar UK, Chatterjee MK. Demographic profile and outcome analysis of a paecliatric intensive care unit in a paecliatric hospital in Kolkata. Journal of the Indian Medical Association 2013; 111(3): 160-2.
103. Santos CAD, Rosa CDOB, Franceschini SDCC, Firmino HH, Ribeiro AQ. Usefulness of the StrongKids Screening Tool in Detecting Anemia and Inflammation in Hospitalized Pediatric Patients. Journal of the American College of Nutrition 2021; 40(2): 155-63.
104. Santos CA, Rosa COB, Franceschini SCC, et al. StrongKids for pediatric nutritional risk screening in Brazil: a validation study. European Journal of Clinical Nutrition 2020; 74(9): 1299-305.
105. Scott JA, Berkley JA, Mwangi I, et al. Relation between falciparum malaria and bacteraemia in Kenyan children: a population-based, case-control study and a longitudinal study. Lancet 2011; 378(9799): 1316-23.
106. Sibomana H, Rugambwa C, Mwenda JM, et al. Impact of routine rotavirus vaccination on all-cause and rotavirus hospitalizations during the first four years following vaccine introduction in Rwanda. 2018; 1(47): 7135-41.
107. Sievers AC, Lewey J, Musafiri P, et al. Reduced paediatric hospitalizations for malaria and febrile illness patterns following implementation of a community-based malaria control programme in rural Rwanda. World Hosp Health Serv 2008; 44(4): 28-35.

108. Silva PmRS, Rosa AM, Hacon SS, Ignotti E. Hospitalização de crianças por asma na Amazônia brasileira: tendência e distribuição espacial. J pediatr (Rio J) 2009; 85(6): 541-6.
109. SoukAloun D, Douangbouphaa V, Phetsouvanh R, et al. Rotavirus diarrhea in hospitalized children under 5years of age in Vientiane, Lao PDR, 2009-2015. 2018; 1(51): 7878-82.
110. Tate JE, Rheingans RD, C.E OR, et al. Rotavirus disease burden and impact and cost- effectiveness of a rotavirus vaccination program in Kenya. Journal of Infectious Diseases 2009; 200(SUPPL. 1): S76-S84.
111. Tennant I, Harding H, Nelson M, Roye-Green K. Microbial isolates from patients in an intensive care unit, and associated risk factors. West Indian med j 2005; 54(4): 225-31.
112. Tette EM, Neizer ML, Nyarko MY, Sifah EK, Sagoe-Moses IA, Nartey ET. Observations from Mortality Trends at The Children's Hospital, Accra, 2003-2013. PLoS ONE 2016; 11(12): e0167947.
113. van Deursen B, Lenglet A, Ariti C, et al. Risks and seasonal pattern for mortality among hospitalized infants in a conflict-affected area of Pakistan, 2013-2016. A retrospective chart review. F1000Res 2019; 8: 954.
114. Wilopo SA, Soenarto Y, Bresee JS, et al. Rotavirus surveillance to determine disease burden and epidemiology in Java, Indonesia, August 2001 through April 2004. Vaccine 2009; 27 Suppl 5: F61-6.
115. Yameogo NV, Kologo KJ, Yameogo AA, et al. [Infective endocarditis in sub-Saharan african children, cross-sectional study about 19 cases in Ouagadougou at Burkina Faso]. Ann Cardiol Angeiol (Paris) 2014; 63(1): 7-10.
116. Young Infants Clinical Signs Study Group. Clinical signs that predict severe illness in children under age 2 months: a multicentre study. The Lancet 2008; 371(9607): 135-42.

Full text not found (19)

1. Abhulimhen-Iyoha BI, Israel-Aina YT. Emergency Blood Transfusion in Children in a Tertiary Hospital in Nigeria: Indications, Frequency and Outcome. West Afr J Med 2018; 35(1): 20-4.
2. Abhulimhen-Iyoha BI, Israel-Aina YT. Pattern of Accidental Childhood Poisoning at the Children Emergency Room of the University of Benin Teaching Hospital, Benin City, Nigeria. West Afr J Med 2018; 35(2): 85-9.
3. Adedeji TO, Bande SA. Epistaxis and its Management in a Tertiary Health Facility in Nigeria: A need for thorough Evaluation. West Afr J Med 2014; 33(3): 195-200.
4. Alarcon J, Palacio S, Restrepo RD. USE OF NONINVASIVE VENTILATION IN PEDIATRIC PATIENTS WITH PLEURAL EFFUSION ASSOCIATED WITH DENGUE VIRUS INFECTION: A RETROSPECTIVE REVIEW. Respiratory Care 2016; 61(10): OF40-OF.
5. Aramburú Miranda NtC. Factores asociados a evolución adversa en pacientes menores de 15 años con diagnóstico de estado epiléptico convulsivo hospitalizados en el Instituto Nacional de Salud del Niño durante el periodo 2008-2013. 2014: 42-.
6. Carlos Urtecho S, Carrera Aliaga D, Castro Castañeda K, Castro Fernández C, Castañeda Robles P. Características clinicas y epidemiológicas de pacientes menores de 5 años con diarrea aguda infecciosa y coprocultivo positivo a enteropatógenos bacterianos en el Instituto Nacional de Salud del Niño, en los años 2007-2008. 2009: 21-.
7. Elusiyan JB, Obiajunwa PO, Senbanjo IO, Anyanbolu HC. A-5 year review of cerebral malaria in nigerian children. Niger Postgrad Med J 2007; 14(1): 60-2.
8. Jalo I, Isaac EW, Alkali YS, Abubakar AM, Galadima UG. Spectrum of Congenital Malformations in Federal Teaching Hospital Gombe: A Ten-Year Review. West Afr J Med 2018; 35(3): 195-8.
9. Meherate A, Alissa A. Road traffic injuries and deaths among patients seen at the Emergency Department of King Hussein Medical Center in a one-year period. Journal of the Bahrain Medical Society 2008; 20(3): 110-3.
10. Merah NA, Okeke CI, Olatosi JO. An audit of surgical admissions to the intensive care unit of the Lagos University Teaching Hospital (1997 - 2002). Niger Postgrad Med J 2006; 13(2): 153-6.
11. Mobin ur R, Khan GS, Afridi U. Disease pattern and outcome among Afghan patients: Our experience in tertiary care hospital in Quetta. Medical Forum Monthly 2012; 23(5): 42-4.
12. Mohite VR, Hiremath P. To Assess the Under Five Mortality Pattern in Tertiary Care Hospital, Krishna Hospital, Karad. International Journal of Nursing Education 2015; 7(2): 215-7.
13. Obiora UJ, Ekpebe PA, Okoye C, David-Idiapho CG. Audit of Childhood Death in a Tertiary Care Center in Niger Delta Region of Nigeria. West Afr J Med 2020; 37(2): 113-7.
14. Okechukwu AA, Nwalozie C. Morbidity and mortality pattern of admissions into the Emergency Paediatric Unit of University of Abuja Teaching Hospital, Gwagwalada. Niger J Med 2011; 20(1): 109-13.
15. Oniyriuka AN. Gender differentials in rates of hospitalization in Benin city mortality and removal from hospital against medical advice among under fives. West African Journal of Nursing 2005; 16(1): 20-4.
16. Seyal T, Ishaaq F, Waheed M, Mannan J, Hanif A. Risk factors for severe protein energy malnutrition in children less than 5 years of age (hospital based study). Pakistan Paediatric Journal 2008; 32(4): 221-4.
17. Siyal AA, Kumar S, Kumar R. Outcome of acute bronchiolitis in hospitalized children at NMC Hospital Nawabshah. Medical Forum Monthly 2010; 21(4): 43-6.
18. Tarlovsky VF, Salmeán GGr. Situación nutricional en pacientes pediátricos internados en un hospital público de la Ciudad de México. Rev bras nutr clí-n 2008; 23(3): 178-83.

19. Veloza AL, Rodníguez PA, Henao AM, Bastos N, Ladino L. MON-P120: Screening Tool for the Assessment of Malnutrition in Paediatrics (STAMP) on Admission to A Tertiary Hospital in Bogotá, Colombia. Clinical Nutrition 2016; 35: S197-S.

Ineligible date range (N=8)

1. Amsalu S, Assefa A. Meningitis in children beyond the neonatal period in Gondar University hospital. Ethiop Med J 2005; 43(3): 175-80.
2. Derbew M, Ahmed E. The pattern of pediatric surgical conditions in Tikur Anbessa Unversity Hospital, Addis Ababa, Ethiopia. Ethiop Med J 2006; 44(4): 331-8.
3. Eck C, Pierre RB, Hambleton IR. Medical paediatric admission patterns at the University Hospital of the West Indies: issues for future planning. 2006; 1(5): 340-5.
4. Etuk IS, Anah MU, Ochighs SO, Eyong M. Pattern of paediatric renal disease in inpatients in Calabar, Nigeria. Tropical Doctor 2006; 36(4): 256.
5. Farhat SCL, Paulo RLP, Shimoda TM, et al. Effect of air pollution on pediatric respiratory emergency room visits and hospital admissions. Braz j med biol res 2005; 38(2): 227-35.
6. Martí-nez Torres E, Polanco Anaya Cn, Pleites Sandoval EBn. Por qué y cómo mueren los niños con dengue? Rev cuba med trop 2008; 60(1).
7. Oguche S, Bukbuk DN, Watila IM. Pattern of hospital admissions of children with poisoning in the Sudano-Sahelian North eastern Nigeria. Niger J Clin Pract 2007; 10(2): 111-5.
8. Veirum JE, Sodeman M, Biai S, Hedegard K, Aaby P. Increased mortality in the year following discharge from a paediatric ward in Bissau, Guinea-Bissau. Acta Paediatr 2007; 96(12): 1832-8.

Ineligible denominator (N=1112)

1. Ababulgu RZ, Tesfaye BT. Characteristics and Outcomes of Children with Type-I Diabetes Mellitus Hospitalized for Ketoacidosis. Curr Diabetes Rev 2020; 16(7): 779-86.
2. Abantanga FA, Nimako B, Amoah M. The range of abdominal surgical emergencies in children older than 1 year at the Komfo Anokye Teaching Hospital, Kumasi, Ghana. Ann Afr Med 2009; 8(4): 236-42.
3. Abate HK, Kidane SZ, Feyessa YM, Gebrehawariat EG. Mortality in children with severe acute malnutrition. Clin Nutr ESPEN 2019; 33: 98-104.
4. Abbas Q, Jamil MT, Haque A, Sayani R. Use of Interventional Radiology in Critically Injured Children Admitted in a Pediatric Intensive Care Unit of a Developing Country. Cureus 2019; 11(1): e3922.
5. Abbas Q, Jamil MT, Jafri L, Haque AU, Khetpal V. Hyperlactetemia And Its Trends In Critically Ill Children Admitted In Pediatric Intensive Care Unit Of A Developing Country. J Ayub Med Coll Abbottabad 2016; 28(4): 660-3.
6. Abbas Q, Merchant QU, Nasir B, Haque AU, Salam B, Javed G. Spectrum of Intracerebral Hemorrhage in Children: A Report from PICU of a Resource Limited Country. Crit Care Res Pract 2016; 2016: 9124245.
7. Abbas Q, Shabbir A, Siddiqui NR, Kumar R, Haque A. Burden of neurological illnesses in a pediatric intensive care unit of developing country. Pak 2014; 30(6): 1223-7.
8. Abd Elmoneim A, Hassan IA, Abd Elnaby A, Abou Elmagd A. Epidemiology and outcome of childhood asthma: A clinical study in an Egyptian university medical centre. Eastern Mediterranean Health Journal 2013; 19(6): X520-6.
9. AbdAllah NB, Zeitoun AED, Fattah MGEDA. Adherence to standard admission and discharge criteria and its association with outcome of pediatric intensive care unit cases in Al-Ahrar Hospital Zagazig. Egyptian Pediatric Association Gazette 2016; 64(3): 111-9.
10. Abdelaziz OS, Elwany MN, Abbassy MA, Mostafa MS. Profile of road traffic accidents'related head injuries in alexandria during the egyptian revolution 2011. Neurosurgery Quarterly 2015; 25(1): 1-5.
11. Abdelgadir J, Punchak M, Smith ER, et al. Pediatric traumatic brain injury at Mbarara Regional Referral Hospital, Uganda. J Clin Neurosci 2018; 47: 79-83.
12. Abdelraheem M, Ali el T, Osman R, et al. Outcome of acute kidney injury in Sudanese children - an experience from a sub-Saharan African unit. Perit Dial Int 2014; 34(5): 526-33.
13. Abdelrahim NA, Fadl-Elmula IM, Ali HM. Bacterial meningitis in Sudanese children; critical evaluation of the clinical decision using clinical prediction rules. BMC Pediatr 2019; 19(1): 319.
14. Abebe T, Girmay M, G GM, Tesfaye M. The epidemiological profile of pediatric patients admitted to the general intensive care unit in an Ethiopian university hospital. Int J Gen Med 2015; 8: 63-7.
15. Abeid KA, Jani B, Cortese MM, et al. Monovalent Rotavirus Vaccine Effectiveness and Impact on Rotavirus Hospitalizations in Zanzibar, Tanzania: Data From the First 3 Years After Introduction. J Infect Dis 2017; 215(2): 183-91.
16. Abouelnour A, Zaki ME, Hassan R, Elkannishy SMH. Phenotypic and genotypic identification of Staphylococcus aureus resistant to clindamycin in Mansoura University Children Hospital, Egypt. African Journal of Clinical and Experimental Microbiology 2020; 21(1): 30-5.

17. Abramczyk ML, Carvalho WB, Medeiros EA. Preventing catheter-associated infections in the Pediatric Intensive Care Unit: impact of an educational program surveying policies for insertion and care of central venous catheters in a Brazilian teaching hospital. Braz J Infect Dis 2011; 15(6): 573-7.
18. Abrar S, Ansari MJ, Mittal M, Kushwaha KP. Predictors of Mortality in Paediatric Myocarditis. J Clin Diagn Res 2016; 10(6): SC12-6.
19. Abu Faddan NH, Aly SA, Abou Faddan HH. Nosocomial Clostridium difficile-associated diarrhoea in Assiut University Children's Hospital, Egypt. Paediatr Int Child Health 2016; 36(1): 39-44.
20. Abu-Raya B, Goldfarb DM, Smieja M, et al. The prevalence and clinical characteristics of pertussis-associated pneumonia among infants in Botswana. BMC Pediatr 2019; 19(1): 444.
21. Abubakar U. Antibiotic use among hospitalized patients in northern Nigeria: A multicenter point-prevalence survey. BMC Infectious Diseases 2020; 20(1).
22. Abucejo-Ladesma E, Simoes EA, Lupisan SP, et al. Serious community-acquired paediatric infections in rural Asia (Bohol Island, Philippines): bacterial meningitis in children less than 5 years of age. Scand J Infect Dis 2007; 39(11-12): 983-9.
23. Abula T, Wondmikun Y. The pattern of acute poisoning in a teaching hospital, north-west Ethiopia. Ethiop Med J 2006; 44(2): 183-9.
24. Acherjya GK, Ali M, Alam ABMS, Rahman MM, Mowla SGM. The Scenario of Acute Poisoning in Jashore, Bangladesh. Journal of Toxicology 2020; 2020.
25. Achidi EA, Apinjoh TO, Anchang-Kimbi JK, Mugri RN, Ngwai AN, Yafi CN. Severe and uncomplicated falciparum malaria in children from three regions and three ethnic groups in Cameroon: prospective study. Malar J 2012; 11: 215.
26. Ackerman H, Ayestaran A, Olola CHO, et al. The effect of blood transfusion on outcomes among African children admitted to hospital with Plasmodium falciparum malaria: a prospective, multicentre observational study. Lancet Haematol 2020; 7(11): e789-e97.
27. Acquah SE, Quaye L, Sagoe K, Ziem JB, Bromberger PI, Amponsem AA. Susceptibility of bacterial etiological agents to commonly-used antimicrobial agents in children with sepsis at the Tamale Teaching Hospital. BMC Infect Dis 2013; 13: 89.
28. Adam MA, Adam NK, Mohamed BA. Prevalence of sickle cell disease and sickle cell trait among children admitted to Al Fashir Teaching Hospital North Darfur State, Sudan. BMC Res Notes 2019; 12(1): 659.
29. Adam MB, Muma S, Modi JA, et al. Paediatric and obstetric outcomes at a faith-based hospital during the 100-day public sector physician strike in Kenya. BMJ glob 2018; 3(2): e000665.
30. Adamou H, Magagi IA, Habou O, et al. Etiology and prognosis of acute mechanical intestinal obstructions at the National Hospital of Zinder: Cross-sectional study of 171 patients. Pan Afr Med J 2016; 24: 248.
31. Adamu AL, Aliyu MH, Galadanci NA, et al. Deaths during tuberculosis treatment among paediatric patients in a large tertiary hospital in Nigeria. PLoS ONE 2017; 12(8): e0183270.
32. Adedeji TO, Sogebi OA, Tobih JE. Pattern of Otorhinolaryngological Admissions via Emergency Unit in a Suburban Tertiary Center. Int J Biomed Sci 2015; 11(3): 146-51.
33. Adegbehingbe OO, Akinyoola AL, Oginni LM. Predictive factors for primary amputation in trauma patients in a Nigerian university teaching hospital. East Afr Med J 2006; 83(10): 539-44.
34. Adegbola RA, Hill PC, Secka O, et al. Serotype and antimicrobial susceptibility patterns of isolates of Streptococcus pneumoniae causing invasive disease in The Gambia 1996-2003. Trop Med Int Health 2006; 11(7): 1128-35.
35. Adel A, Boughoufalah A, Saegerman C, et al. Epidemiology of visceral leishmaniasis in Algeria: an update. PLoS ONE 2014; 9(6): e99207.
36. Adeleye AO, Olowookere KG, Olayemi OO. Clinicoepidemiological profiles and outcomes during first hospital admission of head injury patients in Ikeja, Nigeria. A prospective cohort study. Neuroepidemiology 2009; 32(2): 136-41.
37. Adem F, Tasew A, Siraj A, Mohammed M. Treatment Outcomes and Associated Factors among Children Hospitalized with Acute Bacterial Meningitis in Eastern Ethiopia: A Cross-Sectional Study. Patient relat 2020; 11: 241-8.
38. Ademuyiwa AO, Bode CO, Adesanya OA, Elebute OA. Non-trauma related paediatric abdominal surgical emergencies in Lagos, Nigeria: Epidemiology and indicators of survival. Niger Med J 2012; 53(2): 76-9.
39. Adimasu M, Sebsibie G, Abebe F, Baye G, Abere K. Recovery time from severe acute malnutrition and associated factors among under-5 children in Yekatit 12 Hospital, Addis Ababa, Ethiopia: a retrospective cohort study. Epidemiol Health 2020; 42: e2020003.
40. Adoga A, Okwori E, Yaro J, Iduh A. Pediatric otorhinolaryngology emergencies at the Jos University Teaching Hospital: Study of frequency, management, and outcomes. Annals of African Medicine 2017; 16(2): 81-4.
41. Adoga A, Ozoilo K. The epidemiology and type of injuries seen at the accident and emergency unit of a Nigerian referral center. Journal of Emergencies, Trauma and Shock 2014; 7(2): 77-82.

42. Adudu OP, Ogunrin OA, Adudu OG. Morbidity and mortality patterns among neurological patients in the intensive care unit of a tertiary health facility. Ann Afr Med 2007; 6(4): 174-9.
43. Afolabi BM, Clement CO, Ekundayo A, Dolapo D. A hospital-based estimate of major causes of death among under-five children from a health facility in Lagos, Southwest Nigeria: possible indicators of health inequality. Intern 2012; 11: 39.
44. Afonso ET, Minamisava R, Bierrenbach AL, et al. Effect of 10-valent pneumococcal vaccine on pneumonia among children, Brazil. Emerg Infect Dis 2013; 19(4): 589-97.
45. Afridi JM, Amir S, Munir A, Raheem F, Rehman Y. Types and subtypes of Leukaemia in newly diagnosed patients admitted in the department of child health in a tertiary care hospital. Journal of Medical Sciences (Peshawar) 2018; 26(2): 99-101.
46. Agbenorku P. Early childhood severe scalds in a developing country: A 3-year retrospective study. Burns Trauma 2013; 1(3): 122-7.
47. Agbenorku P. Modernized standards in burns management: a comparative study in Komfo Anokye Teaching Hospital, Kumasi, Ghana. Burns 2013; 39(5): 990-6.
48. Agossou J, Ebruke C, Noudamadjo A, et al. Declines in Pediatric Bacterial Meningitis in the Republic of Benin Following Introduction of Pneumococcal Conjugate Vaccine: Epidemiological and Etiological Findings, 2011-2016. Clinical Infectious Diseases 2019; 69: S140-S7.
49. Agulnik A, Cardenas A, Carrillo AK, et al. Clinical and organizational risk factors for mortality during deterioration events among pediatric oncology patients in Latin America: A multicenter prospective cohort. Cancer 2021; 01: 01.
50. Agweyu A, Lilford RJ, English M, Clinical Information Network Author G. Appropriateness of clinical severity classification of new WHO childhood pneumonia guidance: a multi-hospital, retrospective, cohort study. Lancet Glob Health 2018; 6(1): e74-e83.
51. Ahmad S, Ellis JC, Kamwendo H, Molyneux EM. Impact of HIV infection and exposure on survival in critically ill children who attend a paediatric emergency department in a resource-constrained setting. Emerg Med J 2010; 27(10): 746-9.
52. Ahmad S, Rafique T, Hussain N, Akbar A. Risk factors of malnutrition in under 5 year children admitted at DHQ teaching hospital, Dera Ghazi Khan. Pakistan Journal of Medical and Health Sciences 2018; 12(2): 546-50.
53. Ahmed A, Ali M, Khan M, Khan F. Perioperative cardiac arrests in children at a university teaching hospital of a developing country over 15 years. Paediatr Anaesth 2009; 19(6): 581-6.
54. Ahmed E, Chaka T. The pattern of orthopedic admissions in Tikur Anbessa University Hospital, Addis Ababa. Ethiop Med J 2005; 43(2): 85-91.
55. Ahmed R, Azim A, Nangialay A, Haque A, Jurair H. Frequency of Pediatric Acute Respiratory Distress Syndrome Based on Oxygen Saturation Index in Pediatric Intensive Care Unit of a Developing Country. Cureus 2019; 11(12): e6444.
56. Ahoyo TA, Martin-Odoom A, Bankole HS, et al. Epidemiology and prevention of nosocomial pneumonia associated with Panton-Valentine Leukocidin (PVL) producing Staphylococcus aureus in Departmental Hospital Centre of Zou Collines in Benin. Ghana Med J 2012; 46(4): 234-40.
57. Ahrens JO, Morrow BM, Argent AC. Influenza A(H1N1)pdm09 in critically ill children admitted to a paediatric intensive care unit, South Africa. Southern African Journal of Critical Care 2015; 31(1): 4-7.
58. Aiken AM, Mturi N, Njuguna P, et al. Risk and causes of paediatric hospital-acquired bacteraemia in Kilifi District Hospital, Kenya: a prospective cohort study. Lancet 2011; 378 North American Edition(9808): 2021-7.
59. Aikphaibul P, Theerawit T, Sophonphan J, Wacharachaisurapol N, Jitrungruengnij N, Puthanakit T. Risk factors of severe hospitalized respiratory syncytial virus infection in tertiary care center in Thailand. Influenza other respi 2021; 15(1): 64-71.
60. Aissi W, Ben Hellel K, Habboul Z, et al. Epidemiological, clinical and biological features of infantile visceral leishmaniasis at Kairouan hospital (Tunisia): about 240 cases. Bull Soc Pathol Exot 2015; 108(4): 265-71.
61. Ajetunmobi WA, Orimadegun AE, Brown BJ, et al. Haemoglobinuria among children with severe malaria attending tertiary care in Ibadan, Nigeria. Malar J 2012; 11: 336.
62. Akech SO, Hassall O, Pamba A, et al. Survival and haematological recovery of children with severe malaria transfused in accordance to WHO guidelines in Kilifi, Kenya. Malar J 2008; 7: 256.
63. Akech S, Ayieko P, Irimu G, Stepniewska K, English M. Magnitude and pattern of improvement in processes of care for hospitalised children with diarrhoea and dehydration in Kenyan hospitals participating in a clinical network. Tropical Medicine & International Health 2019; 24(1): 73-80.
64. Akeme Yamamoto AC, de Paula CR, Dias LB, et al. Epidemiological and clinical characteristics of nosocomial candidiasis in university hospitals in Cuiaba--Mato Grosso, Brazil. Rev Iberoam Micol 2012; 29(3): 164-8.
65. Akhter S, Ahmed T, Sarker SA, et al. Factors Associated with Klebsiella Bacteremia and Its Outcome in Under-Five Children Admitted with Diarrhea. Int J Pediatr 2016; 2016: 4760610.
66. Akinkugbe O, Cooke FJ, Pathan N. Healthcare-Associated bacterial infections in the paediatric ICU. JAC-Antimicrobial Resistance 2020; 2(3).

67. Akinkuotu AC, Purcell LN, Kayange L, Phillips MR, Hayes-Jordan A, Charles AG. Trends and outcomes following intentional injuries in pediatric patients in a resource-limited setting. Pediatr Surg Int 2021; 18: 18.
68. Akoua-Koffi C, Asse Kouadio V, Yao Atteby JJ. Hospital-based surveillance of rotavirus gastroenteritis among children under 5 years of age in the Republic of Ivory Coast: a cross-sectional study. BMJ Open 2014; 4(1): e003269.
69. Akram F, Pietroni MA, Bardhan PK, Bibi S, Chisti MJ. Prevalence, clinical features, and outcome of pseudomonas bacteremia in under-five diarrheal children in bangladesh. ISRN Microbiol 2014; 2014: 469758.
70. Akuffo R, Armah G, Clemens M, et al. Prevalence of enteric infections among hospitalized patients in two referral hospitals in Ghana. BMC Res Notes 2017; 10(1): 292.
71. Al Amad MA, Al Mahaqri AA, Al Serouri AA, Khader YS. Severe Acute Respiratory Infections With Influenza and Noninfluenza Respiratory Viruses: Yemen, 2011-2016. Inquiry 2019; 56: 46958019850731.
72. Al Jarousha AM, Al Afifi A. Epidemiology and Risk Factors Associated with Developing Bacterial Meningitis among Children in Gaza Strip. Iranian Journal of Public Health 2014; 43(9): 1176-83.
73. Al-Abdallat EM, Al-Ali RA, Salameh GA. Accidental genital trauma in the female children in Jordan and the role of forensic medicine. Saudi Med J 2013; 34(10): 1043-7.
74. Al-Abdallat M, Dawson P, Haddadin AJ, et al. Influenza hospitalization epidemiology from a severe acute respiratory infection surveillance system in Jordan, January 2008-February 2014. Influenza other respi 2016; 10(2): 91-7.
75. Al-Ali MK, Batchoun RG, Al-Nour TM. Etiology of community-acquired pneumonia in hospitalized patients in Jordan. Saudi Med J 2006; 27(6): 813-6.
76. Al-Jboor W, Almardini R, Al Bderat J, Frehat M, Al Masri H, Alajloni MS. Acute kidney injury in critically ill child. Saudi J Kidney Dis Transpl 2016; 27(4): 740-7.
77. Al-Ramahi R, Hmedat B, Alnjajrah E, Manasrah I, Radwan I, Alkhatib M. Medication dosing errors and associated factors in hospitalized pediatric patients from the South Area of the West Bank - Palestine. Saudi Pharm J 2017; 25(6): 857-60.
78. Al-Sultany ZK, Al-Charrakh AH. Antibiotic resistance patterns of coagulase negative Staphylococcus (CoNS) strains isolated from blood stream infections in Babylon province, Iraq. Annals of Tropical Medicine and Public Health 2020; 23(16).
79. Al-Zacko SM, Zubeer HG, Mohammad AS. Pediatric burns in Mosul: an epidemiological study. Ann 2014; 27(2): 70-5.
80. Alabi BS, Afolabi OA, Dunmade AD, et al. Indications and outcome of tracheostomy in Ilorin, North Central Nigeria: 10 years review. Ann Afr Med 2018; 17(1): 1-6.
81. Alam A, Qureshi SA, Vinje J, Zaidi A. Genetic characterization of norovirus strains in hospitalized children from Pakistan. J Med Virol 2016; 88(2): 216-23.
82. Alam T, Ahmed T, Sarmin M, et al. Risk Factors for Death in Bangladeshi Children Under 5 Years of Age Hospitalized for Diarrhea and Severe Respiratory Distress in an Urban Critical Care Ward. Glob 2017; 4: 2333794X17696685.
83. Alao MA, Ibrahim OR, Asinobi AO, Akinsola A. Long-term survival of children following acute peritoneal dialysis in a resource-limited setting. Kidney Res Clin Pract 2020; 39(4): 469-78.
84. Albin CB, Feema R, Aparna L, Darpanarayan H, Chandran J, Abhilash KPP. Paediatric trauma aetiology, severity and outcome. J 2020; 9(3): 1583-8.
85. Albirmawy OA, Elsheikh MN. Foreign body aspiration, a continuously growing challenge: Tanta University experience in Egypt. Auris Nasus Larynx 2011; 38(1): 88-94.
86. Alemayehu S, Kidanu K, Kahsay T, Kassa M. Risk factors of acute respiratory infections among under five children attending public hospitals in southern Tigray, Ethiopia, 2016/2017. BMC Pediatrics 2019; 19(1).
87. Alfalahi E, Assabri A, Khader Y. Pattern of road traffic injuries in Yemen: a hospital-based study. Pan Afr Med J 2018; 29: 145.
88. Ali AM, Maya E, Lakhoo K. Challenges in managing paediatric osteomyelitis in the developing world: analysis of cases presenting to a tertiary referral centre in Tanzania. Afr J Paediatr Surg 2014; 11(4): 308-11.
89. Ali A, Khowaja AR, Bashir MZ, Aziz F, Mustafa S, Zaidi A. Role of human metapneumovirus, influenza A virus and respiratory syncytial virus in causing WHO-defined severe pneumonia in children in a developing country. PLoS ONE 2013; 8(9): e74756.
90. Ali A, Yousafzai MT, Waris R, et al. RSV associated hospitalizations in children in Karachi, Pakistan: Implications for vaccine prevention strategies. J Med Virol 2017; 89(7): 1151-7.
91. Ali SA, Williams JV, Chen Q, et al. Human metapneumovirus in hospitalized children in Amman, Jordan. Journal of Medical Virology 2010; 82(6): 1012-6.
92. Alievi PT, Carvalho PR, Trotta EA, Mombelli Filho R. The impact of admission to a pediatric intensive care unit assessed by means of global and cognitive performance scales. J Pediatr (Rio J) 2007; 83(6): 505-11.
93. Almeida IA, Jeske S, Mesemburg MA, Berne MEA, Villela MM. Prevalence of and risk factors for intestinal parasite infections in pediatric patients admitted to public hospitals in Southern Brazil. Rev Soc Bras Med Trop 2017; 50(6): 853-6.

94. Almeida JP, Valente IF, Lordelo MDR. Association between pediatric Risk, Injury, Failure, Loss and End Stage Renal Disease score and mortality in a pediatric intensive care unit: a retrospective study. Rev 2018; 30(4): 429-35.
95. Alnawajha SK, Bakry GA, Aljeesh YI. Predictors of acute diarrhoea among hospitalized children in Gaza Governorates: a case-control study. J Health Popul Nutr 2015; 33(1): 1-8.
96. Aloni MN, Kadima BT, Ekulu PM, Budiongo AN, Ngiyulu RM, Gini-Ehungu JL. Acute Crises and Complications of Sickle Cell Anemia Among Patients Attending a Pediatric Tertiary Unit in Kinshasa, Democratic Republic Of Congo. Hematol Rep 2017; 9(2): 6952.
97. Alrifai SB, Alsaadi A, Mahmood YA, Ali AA, Al-Kaisi LA. Prevalence and etiology of nosocomial diarrhoea in children < 5 years in Tikrit teaching hospital. Eastern Mediterranean Health Journal 2009; 15(5): 1111-8.
98. Altmann M, Suarez-Bustamante M, Soulier C, Lesavre C, Antoine C. First Wave of the 2016-17 Cholera Outbreak in Hodeidah City, Yemen - ACF Experience and Lessons Learned. PLoS Curr 2017; 9: 13.
99. Aluisio AR, Umuhire OF, Mbanjumucyo G, et al. Epidemiologic Characteristics of Pediatric Trauma Patients Receiving Prehospital Care in Kigali, Rwanda. Pediatr Emerg Care 2019; 35(9): 630-6.
100. Alvarez-Hernandez G, Candia-Plata Mdel C, Delgado-de la Mora J, Acuna-Melendrez NH, Vargas-Ortega AP, Licona-Enriquez JD. Rocky Mountain spotted fever in Mexican children: Clinical and mortality factors. Salud Publica Mex 2016; 58(3): 385-92.
101. Alves Nery A, da Silva Alves M, Andrade Rios M, Neves de Assunção P, Arcanjo Matos Filho. EPIDEMIOLOGICAL PROFILE OF THE MORBIDITY AND MORTALITY BY EXTERNAL CAUSES IN A GENERAL HOSPITAL. Journal of Nursing UFPE / Revista de Enfermagem UFPE 2013; 7(2): 562-71.
102. Alvis-Guzman N, Pinzon-Redondo H, Ortiz Ibanez L, Orozco Africano J, Trucco Lemaitre J. Mortality In A University Pediatric Hospital In Colombia 2000 - 2014. Value Health 2015; 18(7): A833.
103. Alvis-Guzman N, Orozco-Africano J, Paternina-Caicedo A, et al. Treatment costs of diarrheal disease and all-cause pneumonia among children under-5 years of age in Colombia. Vaccine 2013; 31 Suppl 3: C58-62.
104. Amador JJ, Vasquez J, Orozco M, et al. Rotavirus disease burden, Nicaragua 2001-2005: defining the potential impact of a rotavirus vaccination program. Int J Infect Dis 2010; 14(7): e592-5.
105. Amancio CT, Nascimento LFC, Amancio TT. Poluentes ambientais e chance de internações por asma em crianças -São José dos Campos, Brasil, nos anos 2004-2005. Rev bras crescimento desenvolv hum 2012; 22(2): 202-8.
106. Amiri M, Lornejad HR, Barakati SH, Motlagh ME, Kelishadi R, Poursafa P. Mortality inequality in 1-59 months children across iranian provinces: Referring system and determinants of death based on hospital records. International Journal of Preventive Medicine 2013; 4(3): 265-70.
107. Amonkar P, N R, Gavhane J. A study of critically ill children presenting with seizures regardless of seizure duration admitted in the PICU of a tertiary hospital in India. Epilepsy Behav Rep 2020; 14: 100382.
108. Amouzou KS, Kouevi-Koko TE, Egbohou P, Tchetike P, Hounmenou AKF. Epidemiology and outcomes of in-hospital acute burn-patients in Togo: A retrospective analysis. Burns Open 2019; 3(4): 141-6.
109. Amsalu S, Hurrisa Z, Nuri S. Tuberculosis in children, Northwest Ethiopia. Ethiop Med J 2007; 45(2): 159-63.
110. Anders KL, Nguyet NM, Chau NV, et al. Epidemiological factors associated with dengue shock syndrome and mortality in hospitalized dengue patients in Ho Chi Minh City, Vietnam. Am J Trop Med Hyg 2011; 84(1): 127-34.
111. Andrade IG, Queiroz JW, Cabral AP, Lieberman JA, Jeronimo SM. Improved sanitation and income are associated with decreased rates of hospitalization for diarrhoea in Brazilian infants. Trans R Soc Trop Med Hyg 2009; 103(5): 506-11.
112. Andrade SSCdA, Jorge MHPdM. Estimativa de sequelas físicas em vítimas de acidentes de transporte terrestre internadas em hospitais do Sistema Único de Saúde. Rev bras epidemiol 2016; 19(1): 100-11.
113. Andrews JR, Leung DT, Ahmed S, et al. Determinants of severe dehydration from diarrheal disease at hospital presentation: Evidence from 22 years of admissions in Bangladesh. PLoS Neglected Tropical Diseases 2017; 11(4).
114. Andriatahirintsoa E, Raboba JL, Rahajamanana VL, et al. Impact of 10-Valent Pneumococcal Conjugate Vaccine on Bacterial Meningitis in Madagascar. Clin Infect Dis 2019; 69(Supplement_2): S121-S5.
115. Aneley Getahun S, Parry CM, Crump JA, et al. A retrospective study of patients with blood culture-confirmed typhoid fever in Fiji during 2014-2015: Epidemiology, clinical features, treatment and outcome. Transactions of the Royal Society of Tropical Medicine and Hygiene 2019; 113(12): 764-70.
116. Angkeabos N, Rin E, Vichit O, et al. Pediatric hospitalizations attributable to rotavirus gastroenteritis among Cambodian children: Seven years of active surveillance, 2010-2016. Vaccine 2018; 36(51): 7856-61.
117. Anigilaje EA, Adebayo AI, Ocheni SA. Acute kidney injury in children: A study of etiology, clinical profile, and short-term outcomes at the University of Abuja Teaching Hospital,Gwagwalada,Abuja, Nigeria
Saudi journal of kidney diseases and transplantation: an official publication of the Saudi Center for Organ Transplantation, Saudi Arabia 2019; 30(2): 421-39.
118. Animasahun BA, Gbelee OH, Ogunlana AT, Njokanma OF, Odusanya O. Profile and outcome of patients with post-neonatal tetanus in a tertiary centre in south west Nigeria: any remarkable reduction in the scourge? Pan Afr Med J 2015; 21: 254.
119. Anis ur R, Jahanzeb M, Siddiqui TS, Idris M. Frequency and clinical presentation of UTI among children of Hazara Division, Pakistan. J Ayub Med Coll Abbottabad 2008; 20(1): 63-5.

120. Anis ur R, Siddiqui TS, Idris M. Clinical outcome in measles patients hospitalized with complications. J Ayub Med Coll Abbottabad 2008; 20(2): 14-6.
121. Anjum R, Sharma V, Jindal R, Singh TP, Rathee N. Epidemiologic pattern of paediatric supracondylar fractures of humerus in a teaching hospital of rural India: A prospective study of 263 cases. Chin J Traumatol 2017; 20(3): 158-60.
122. Annamalay AA, Jroundi I, Bizzintino J, et al. Rhinovirus C is associated with wheezing and rhinovirus A is associated with pneumonia in hospitalized children in Morocco. J Med Virol 2017; 89(4): 582-8.
123. Annamalay AA, Lanaspa M, Khoo SK, et al. Rhinovirus species and clinical features in children hospitalised with pneumonia from Mozambique. Trop Med Int Health 2016; 21(9): 1171-80.
124. Anochie I, Eke F, Okpere A. Childhood acute glomerulonephritis in Port Harcourt, Rivers State, Nigeria. Niger J Med 2009; 18(2): 162-7.
125. Anochie PI, Onyeneke EC, Asowata EO, et al. The role of rotavirus associated with pediatric gastroenteritis in a general hospital in Lagos, Nigeria. Germs 2013; 3(3): 81-9.
126. Antunes FP, Costa Mda C, Paim JS, et al. Trends in hospitalizations for respiratory diseases in Salvador, Bahia State, Brazil, 1998-2009. 2012; 1(5): 869-77.
127. Anwari P, Safi N, Payne DC, et al. Rotavirus is the leading cause of hospitalizations for severe acute gastroenteritis among Afghan children <5 years old. Vaccine 2018; 36(51): 7765-8.
128. Araujo OR, Almeida CG, Lima-Setta F, Prata-Barbosa A, Colleti Junior J, Brazilian Research Network in Pediatric Intensive C. The Impact of the Novel Coronavirus on Brazilian PICUs. Pediatr Crit Care Med 2020; 21(12): 1059-63.
129. Araya S, Galeano F, Amarilla S, et al. Prognostic factors of severity of invasive community acquired Staphylococcus aureus infections in children. Arch Argent Pediatr 2019; 117(6): 381-7.
130. Araya S, Lovera D, Zarate C, et al. Application of a Prognostic Scale to Estimate the Mortality of Children Hospitalized with Community-acquired Pneumonia. Pediatr Infect Dis J 2016; 35(4): 369-73.
131. Araya S, Galeano F, Amarilla S, et al. Factores pronósticos de gravedad de las infecciones invasivas por Staphylococcus aureus adquiridas en la comunidad en niños. Arch argent pediatr 2019; 117(6): 381-7.
132. Arguni E, Karyanti MR, Satari HI, Hadinegoro SR. Diphtheria outbreak in Jakarta and Tangerang, Indonesia: Epidemiological and clinical predictor factors for death. PLoS ONE 2021; 16(2): e0246301.
133. Ariaratnam CA, Thuraisingam V, Kularatne SA, et al. Frequent and potentially fatal envenoming by hump-nosed pit vipers (Hypnale hypnale and H. nepa) in Sri Lanka: lack of effective antivenom. Trans R Soc Trop Med Hyg 2008; 102(11): 1120-6.
134. Arican P, Bozkurt O, Cavusoglu D, et al. Various neurological symptoms with vitamin B12 deficiency and posttreatment evaluation. Journal of Pediatric Neurosciences 2020; 15(4): 365-9.
135. Arranz J, Lundeby KM, Hassan S, et al. Clinical features of suspected Ebola cases referred to the Moyamba ETC, Sierra Leone: Challenges in the later stages of the 2014 outbreak. BMC Infectious Diseases 2016; 16(1).
136. Arshad MS, Anwar-Ul-Haq HM, Adnan M, Zulqarnain A. Frequency and pattern of Paediatric Heart Diseases: Five years experience at The Children's Hospital, Multan. Pak 2020; 36(6): 1308-12.
137. Asare H, Carboo J, Nel ED, et al. Mortality in relation to profiles of clinical features in Ghanaian severely undernourished children aged 0-59 months: an observational study. Br J Nutr 2020: 1-9.
138. Asbjornsdottir KH, Slyker JA, Maleche-Obimbo E, et al. Breastfeeding Is Associated with Decreased Risk of Hospitalization among HIV-Exposed, Uninfected Kenyan Infants. J Hum Lact 2016; 32(3): NP61-6.S
139. Ashraf M, Shahzad N, Hussain A, Tak SA, Bukhari ST, Kachru A. Incidence of pediatric acute kidney injury in hospitalized patients. Saudi J Kidney Dis Transpl 2016; 27(6): 1188-93.
140. Assefa Z, A Gy. Acute Appendicitis in Children Admitted to Zewditu Memorial Hospital. Ethiop Med J 2014; 52(4): 189-95.
141. Assogba K, Balaka B, Touglo FA, Apetse KM, Kombate D. Febrile seizures in one-five aged infants in tropical practice: Frequency, etiology and outcome of hospitalization. J 2015; 10(1): 9-12.
142. Assoumou A, Adoubryn KD, Aboum KS, Kouadio-Yapo CG, Ouhon J. Symptomatic and asymptomatic Plasmodium falciparum infection in children from 6 months to 6 years old in the Abobo general hospital (Abidjan, Cote d'Ivoire). Bull Soc Pathol Exot 2008; 101(1): 50-3.
143. Atwell K, Bartley C, Cairns B, Charles A. The epidemiologic characteristics and outcomes following intentional burn injury at a regional burn center. Burns 2020; 46(2): 441-6.
144. Aurangzeb B, Nisar YB, Hazir T, Burki F, Hassan M. Clinical outcome in children hospitalized with complicated measles. J Coll Physicians Surg Pak 2005; 15(9): 547-51.
145. Avcu Gl, Bal ZS, Saz EU, Aydemir S, Vardar Fl. Prevalence of Bacterial Agents in Children with Acute Gastroenteritis in the Pediatric Emergency Department of Ege University School of Medicine. Journal of Pediatric Infection / Cocuk Enfeksiyon Dergisi 2016; 10(2): 49-53.
146. Awad S, Hatim R, Khader Y, et al. Bronchiolitis clinical practice guidelines implementation: surveillance study of hospitalized children in Jordan. Multidiscip 2020; 15(1): 673.

147. Awad S, Khader Y, Mansi M, et al. Viral Surveillance of Children with Acute Respiratory Infection in Two Main Hospitals in Northern Jordan, Irbid, during Winter of 2016. J 2020; 15(1): 1-10.
148. Awasthi S. A composite management intervention plus financial incentives reduced mortality from malaria in children less than 5 years of age. Evidence Based Medicine 2008; 13(2): 59-.
149. Awasthi S, Agarwal GG, Mishra V, et al. Four-country surveillance of intestinal intussusception and diarrhoea in children. J Paediatr Child Health 2009; 45(3): 82-6.
150. Awasthi S, Pandey CM, Verma T, Mishra N. Incidence of community acquired pneumonia in children aged 2-59 months of age in Uttar Pradesh and Bihar, India, in 2016: An indirect estimation. PLoS ONE 2019; 14(3).
151. Awasthi S, Rastogi T, Mishra N, et al. Chest radiograph findings in children aged 2-59 months hospitalised with community-acquired pneumonia, prior to the introduction of pneumococcal
conjugate vaccine in India: a prospective multisite observational study. BMJ Open 2020; 10(5): e034066.
152. Ayana B, Mekonen B, Lollino N. The 'hit by a stick' disease: an epidemiologic study of the causes of trauma in a non-profit hospital in rural Ethiopia. Trop Doct 2012; 42(1): 1-4.
153. Ayieko P, Akumu AO, Griffiths UK, English M. The economic burden of inpatient paediatric care in Kenya: household and provider costs for treatment of pneumonia, malaria and meningitis. Cost Eff Resour Alloc 2009; 7: 3.
154. Ayieko P, Okiro EA, Edwards T, Nyamai R, English M. Variations in mortality in children admitted with pneumonia to Kenyan hospitals. PLoS ONE 2012; 7(11): e47622.
155. Azmi S, Aljunid SM, Maimaiti N, et al. Assessing the burden of pneumonia using administrative data from Malaysia, Indonesia, and the Philippines. Int J Infect Dis 2016; 49: 87-93.
156. Azziz-Baumgartner E, Alamgir A, Rahman M, et al. Incidence of influenza-like illness and severe acute respiratory infection during three influenza seasons in Bangladesh, 2008-2010. Bulletin of the World Health Organization 2012; 90(1): 12-9.
157. Ba ID, Ba A, Faye PM, et al. Particularities of liver abscesses in children in Senegal: Description of a series of 26 cases. Arch Pediatr 2016; 23(5): 491-6.
158. Babakazo P, Lubula L, Disasuani W, et al. The national and provincial burden of medically attended influenza-associated influenza-like illness and severe acute respiratory illness in the Democratic Republic of Congo, 2013-2015. 2018; 1(6): 695-705.
159. Babar TF, Zaman M, Khan MN, Khan MD. Risk factors of preseptal and orbital cellulitis. J Coll Physicians Surg Pak 2009; 19(1): 39-42.
160. Babatola AO, Olatunya OS, Ojo TO, Taiwo AB, Fadare JO. Profile of children admitted for severe acute malnutrition in a tertiary hospital in southwestern nigeria. Journal of Nepal Paediatric Society 2019; 39(1): 42-8.
161. Bagahirwa I, Mukeshimana M, Cherian T, et al. Presentation of pediatric unintentional injuries at rural hospitals in rwanda: A retrospective study. Annals of Global Health 2020; 86(1): 1-8.
162. Bagattini AM, Policena G, Minamisava R, et al. The data used to build the models: Pertussis morbidity and mortality burden considering various Brazilian data sources. Vaccine 2021; 39(1): 137-46.
163. Baggett HC, Chittaganpitch M, Thamthitiwat S, et al. Incidence and epidemiology of hospitalized influenza cases in rural Thailand during the influenza A (H1N1)pdm09 pandemic, 2009-2010. PLoS ONE 2012; 7(11): e48609.
164. Baggett HC, Peruski LF, Olsen SJ, et al. Incidence of pneumococcal bacteremia requiring hospitalization in rural Thailand. Clin Infect Dis 2009; 48 Suppl 2: S65-74.
165. Bahloul M, Ben Hamida C, Chelly H, et al. Severe head injury among children: prognostic factors and outcome. Injury 2009; 40(5): 535-40.
166. Bahloul M, Chaari A, Chabchoub I, et al. Outcome analysis and outcome predictors of traumatic head injury in childhood: Analysis of 454 observations. J 2011; 4(2): 198-206.
167. Bahloul M, Chelly H, Gargouri R, et al. Traumatic head injury in children in south Tunisia epidemiology, clinical manifestations and evolution. A propos 454 cases. Tunisie Medicale 2009; 87(1): 28-37.
168. Baidya S, Hazra A, Datta S, Das AK. A study of antimicrobial use in children admitted to pediatric medicine ward of a tertiary care hospital. Indian J Pharmacol 2017; 49(1): 10-5.
169. Bajracharya L. Scrub Typhus in Children at Tribhuvan University Teaching Hospital in Nepal. Pediatric health med 2020; 11: 193-202.
170. Bajwala VR, John D, Rajasekar TD, Murhekar MV. Severity and costs associated with hospitalization for dengue in public and private hospitals of Surat city, Gujarat, India, 2017-2018. Trans R Soc Trop Med Hyg 2019; 113(11): 661-9.
171. Baker JM, Alonso WJ. Rotavirus vaccination takes seasonal signature of childhood diarrhea back to pre-sanitation era in Brazil. J Infect 2018; 76(1): 68-77.
172. Ballot DE, Davies VA, Cooper PA, Chirwa T, Argent A, Mer M. Retrospective cross-sectional review of survival rates in critically ill children admitted to a combined paediatric/neonatal intensive care unit in Johannesburg, South Africa, 2013-2015. BMJ Open 2016; 6(6): e010850.
173. Banajeh SM, Ashoor O, Al-Magramy AS. Childhood very severe pneumonia and meningitis-related hospitalization and death in Yemen, before and after introduction of H. influenzae type b (Hib) vaccine. Eastern Mediterranean Health Journal 2014; 20(7): 431-41.

174. Banga D, Baren M, Ssonko NV, et al. Comorbidities and Factors Associated with Mortality among Children under Five Years Admitted with Severe Acute Malnutrition in the Nutritional Unit of Jinja Regional Referral Hospital, Eastern Uganda. Int J Pediatr 2020; 2020: 7809412.
175. Banga S, Azad C, Gupta R, et al. Changing Clinicoradiologic Spectrum of Intracranial Neurotuberculosis in Children: A Cross-sectional Study. J Child Neurol 2020; 35(13): 879-88.
176. Bansal A, Singhi SC, Singhi PD, et al. Non traumatic coma. Indian Journal of Pediatrics 2005; 72(6): 467-73.
177. Banstola A. The epidemiology of hospitalization for pneumonia in children under five in the rural western region of Nepal: a descriptive study. PLoS ONE 2013; 8(8): e71311.
178. Baqui AH, Rahman M, Zaman K, et al. A population-based study of hospital admission incidence rate and bacterial aetiology of acute lower respiratory infections in children aged less than five years in Bangladesh. J Health Popul Nutr 2007; 25(2): 179-88.
179. Bari A, Mushtaq A, Naz S, Baneen U, Khan MZ, Ahmad TM. Preventable Diseases - Leading Cause of Death in Developing Countries. Pakistan Paediatric Journal 2013; 37(2): 95-100.
180. Barreira ER, Munoz GO, Cavalheiro PO, et al. Epidemiology and outcomes of acute respiratory distress syndrome in children according to the Berlin definition: a multicenter prospective study. Critical care medicine 2015; 43(5): 947-53.
181. Barrera HP, Lozano LC, Gutiérrez JA, Tierradentro M, Castro CA. Prevalence of child abuse seen in the Pediatric Department of San José Hospital, 2010-2013. Pediatria 2014; 47(4): 90-5.
182. Basak M, Chaudhuri SB, Ishore K, Bhattacherjee S, Das DK. Pattern and trend of morbidity in the infectious disease ward of North Bengal medical college and hospital. Journal of Clinical and Diagnostic Research 2015; 9(11): LC01-LC4.
183. Basnet S, Shrestha S, Ghimire A, et al. Development of a PICU in Nepal: the experience of the first year. Pediatr Crit Care Med 2014; 15(7): e314-20.
184. Batista NOW, Coelho MCdR, Trugilho SM, Pinasco GC, Santos EFdS, Ramos-Silva V. Perfil clínico-epidemiológico de pacientes internados em unidade de cuidados intensivos pediátricos. Rev bras crescimento desenvolv hum 2015; 25(2): 187-93.
185. Batouche DD, Kerboua K. Prognosis of acute renal failure in children in intensive care unit: A pilot study. Current Pediatric Research 2016; 20(1-2): 193-8.
186. Bavdekar SB, Pawar M. Evaluation of an Internet delivered pediatric diagnosis support system (ISABEL) in a tertiary care center in India. Indian Pediatr 2005; 42(11): 1086-91.
187. Bayu D, Mekonnen A, Mohammed J, Bodena D. Magnitude of streptococcus pneumoniae among under-five children with symptom of acute respiratory infection at hiwot fana specialized university hospital, harar, Ethiopia: Associated risk factors and antibacterial susceptibility patterns. Risk Management and Healthcare Policy 2020; 13: 2919-25.
188. Bekhit OESM, Algameel AA, Eldash HH. Application of pediatric index of mortality version 2: score in pediatric intensive care unit in an African developing country. The Pan African medical journal 2014; 17: 185.
189. Ben Fredj M, Dhouib W, Kacem M, et al. Trends and health burden of hospitalized acute respiratory infections and impact of Haemophilus influenza immunization in a Tunisian university hospital: a twelve-year study. Libyan j 2020; 15(1): 1783048.
190. Ben Salah A, El Mhamdi S, Ben Fredj M, et al. Hospital cost of invasive pneumococcal disease in children aged under 15 years old in Tunisia. East Mediterr Health J 2019; 25(12): 861-71.
191. Benabdellah FZ, Soulaymani A, Mokhtari A, Soulaymani-Bencheikh R, Khadmaoui A, Hami H. Economic evaluation of the direct cost resulting from childhood poisoning in Morocco: micro-costing analysis. Arch 2020; 78: 59.
192. Benchekroun I, Boubkraoui MEM, Mekaoui N, Karboubi L, Mahraoui C, Dakhama BSB. Epidemiological profile of respiratory diseases in children hospitalized at the Rabat Children's Hospital, Morocco. Pan Afr Med J 2017; 28: 288.
193. Berezin EN, de Moraes JC, Hong T, Todd M, Seljan MP. Pneumonia hospitalization in Brazil from 2003 to 2007. Int J Infect Dis 2012; 16(8): e583-90.
194. Bezerra de Menezes M, Ponte EV, Bertagni Mingotti CF, et al. Provision of inhaled corticosteroids is associated with decrease in hospital admissions in Brazil: A longitudinal nationwide study. Respir Med 2020; 166: 105950.
195. Bharat A, Mehta A, Tiwari HC, Sharma B, Singh A, Singh V. Spectrum and Immediate Outcome of Acute Kidney Injury in a Pediatric Intensive Care Unit: A Snapshot Study from Indian Subcontinent. Indian J 2019; 23(8): 352-5.
196. Bhatta S, Pant PR, Mytton J. Usefulness of hospital emergency department records to explore access to injury care in Nepal. International Journal of Emergency Medicine 2016; 9(1).
197. Bhattacharya D, Angurana SK, Nallasamy K, Iyer R, Jayashree M. Severe Dengue and Associated Hemophagocytic Lymphohistiocytosis in PICU. Indian J Pediatr 2019; 86(12): 1094-8.
198. Bhattacharya D, Dash N, Kavitha TK, Sharma M, Gautam V, Verma S. Lurking Infantile Pertussis: Experience from a Tertiary Care Center in Northern India. Journal of Pediatric Infectious Diseases 2020; 15(5): 257-61.

199. Bhatti JA, Farooq U, Majeed M, Khan JS, Razzak JA, Khan MM. Fall-related injuries in a low-income setting: Results from a pilot injury surveillance system in Rawalpindi, Pakistan. J Epidemiol Glob Health 2015; 5(3): 283-90.
200. Bibi S, Gilani SY, Shah SR, ul Haq A, Billo AG. Frequency of Hospital Acquired Hyponatremia in a Pediatric Tertiary Care Setting. J Ayub Med Coll Abbottabad 2015; 27(3): 560-3.
201. Bilal JA, Eltahir HG, Al-Nafeesah A, Al-Wutayd O, Adam I. Acute severe malnutrition treatment outcomes in children ages 6-59 months admitted to Singa Hospital, Sudan. Trans R Soc Trop Med Hyg 2020; 114(8): 612-7.
202. Bjornstad EC, Muronya W, Smith ZH, et al. Incidence and epidemiology of acute kidney injury in a pediatric Malawian trauma cohort: a prospective observational study. BMC Nephrol 2020; 21(1): 98.
203. Blanco BP, Branas P, Yoshioka CRM, Ferronato AE. Pediatric bacterial meningitis and meningococcal disease profile in a Brazilian General Hospital. Braz J Infect Dis 2020; 24(4): 337-42.
204. Blomberg B, Manji KP, Urassa WK, et al. Antimicrobial resistance predicts death in Tanzanian children with bloodstream infections: a prospective cohort study. BMC Infect Dis 2007; 7: 43.
205. Boniface R, Museru L, Munthali V, Lett R. Injury Experience in Tanzania - Need for Intervention. East Afr Med J 2013; 90(5): 171-9.
206. Bortolote GS, Brêtas JRdS, Rodrigo GJ, et al. O ambiente estimulador ao desenvolvimento da criança hospitalizada Fatores associados à mortalidade em pacientes hospitalizados por asma
aguda grave em 1994, 1999 e 2004 na Espanha e América Latina. Rev Esc Enferm USP 2008; 42(3): 546-51.
207. Botero G PA, Arango P CA, Castaño DM, et al. Morbimortalidad en la unidad de cuidados intensivos pediátricos del Hospital Infantil Universitario de Manizales durante los años 2006 y 2007. Rev Fac Med (Bogotá) 2010; 58(1): 3-14.
208. Boushab BM, Savadogo M, Sow MS, Dao S. Epidemiological, clinical, and prognostic study of the measles in the Aioun regional hospital in Mauritania. Med 2015; 25(2): 180-3.
209. Bradshaw CJ, Bandi AS, Muktar Z, et al. International Study of the Epidemiology of Paediatric Trauma: PAPSA Research Study. World J Surg 2018; 42(6): 1885-94.
210. Brata Ghosh V, Jhamb U, Singhal R, Krishnan R. Common childhood poisonings and their outcome in a tertiary care center in Delhi. Indian J Pediatr 2013; 80(6): 516-8.
211. Brenes-Chacon H, Gutierrez JM, Camacho-Badilla K, et al. Long-term sequelae secondary to snakebite envenoming: a single centre retrospective study in a Costa Rican paediatric hospital. BMJ paediatr 2020; 4(1): e000735.
212. Brits H, Joubert G. An investigation into the dramatic increase in deaths from gastroenteritis during the summer of 2007/08 at National District Hospital, Bloemfontein, Free State. South African Family Practice 2009; 51(5): 418-22.
213. Broadis E, Chokotho T, Mackay D, Germeni E. First aid management of paediatric burn and scald injuries in Southern Malawi: A mixed methods study. Burns 2020; 46(3): 727-36.
214. Broor S, Dawood FS, Pandey BG, et al. Rates of respiratory virus-associated hospitalization in children aged <5 years in rural northern India. J Infect 2014; 68(3): 281-9.
215. Bundotich JK, Gichuhib MM. Acute poisoning in the Rift Valley Provincial General Hospital, Nakuru, Kenya: January to June 2012. South African Family Practice 2015; 57(3): 214-8.
216. Bunn J, Thindwa M, Kerac M. Features associated with underlying HIV infection in severe acute childhood malnutrition: a cross sectional study. Malawi Med J 2009; 21(3): 108-12.
217. Cáceres DC, Peláez D, Sierra N, Estrada E, Sánchez L. La carga de la enfermedad por rotavirus en niños menores de cinco años, Colombia, 2004. Rev panam salud pública 2006; 20(1): 9-21.
218. Caggiano S, Ullmann N, De Vitis E, et al. Factors That Negatively Affect the Prognosis of Pediatric Community-Acquired Pneumonia in District Hospital in Tanzania. Int 2017; 18(3): 13.
219. Calvo-Salazar RA, David M, Zapata-Mesa MI, Rodriguez-Naranjo CM, Valencia-Acosta NY. Problemas relacionados con medicamentos que causan ingresos por urgencias en un hospital de alta complejidad. Farm 2018; 42(6): 228-33.
220. Cano Londono EM, Mejia Gil IC, Uribe Hernandez K, et al. Delirium during the first evaluation of children aged five to 14years admitted to a paediatric critical care unit. Intensive Crit Care Nurs 2018; 45: 37-43.
221. Carboo JA, Asare H, Nel E, Ricci C, Lombard M, Dolman R. Treatment outcomes and determinants of mortality in children aged 0-59 months diagnosed with complicated severe acute malnutrition in two referral hospitals in Ghana. Vulnerable Children & Youth Studies 2020; 15(4): 329-43.
222. Cardoso AM, Coimbra CE, Jr., Tavares FG. Hospital morbidity among Guarani Indians in Southeastern and Southern Brazil. Rev 2010; 13(1): 21-34.
223. Carlotti APCP, Bachette LG, Carmona F, Manso PH, Vicente WVA, Ramalho FS. Discrepancies between clinical diagnoses and autopsy findings in critically ill children a prospective study. American Journal of Clinical Pathology 2016; 146(6): 701-8.
224. Carneiro VSM, Vila VCDS, Vieira MADS. Trends in pediatric hospitalizations for ambulatory care sensitive respiratory diseases in Brazil. Public Health Nursing 2020.
225. Carrol ED, Guiver M, Nkhoma S, et al. High pneumococcal DNA loads are associated with mortality in Malawian children with invasive pneumococcal disease. Pediatr Infect Dis J 2007; 26(5): 416-22.

226. Carrol ED, Mankhambo LA, Jeffers G, et al. The diagnostic and prognostic accuracy of five markers of serious bacterial infection in Malawian children with signs of severe infection. PLoS ONE 2009; 4(8).
227. Carter MJ, Gurung P, Jones C, et al. Assessment of an Antibody-in-Lymphocyte Supernatant Assay for the Etiological Diagnosis of Pneumococcal Pneumonia in Children. Frontiers in Cellular and Infection Microbiology 2020; 9.
228. Carvalho FHG, Lula JF, Teles LF, Caldeira AP, Carvalho SFG. Hemophagocytic lymphohistiocytosis secondary to visceral leishmaniasis in an endemic area in the north of Minas Gerais, Brazil. Rev Soc Bras Med Trop 2020; 53: e20190491.
229. Cavallin F, Pisani L, Stancari L, et al. Risk Factors for Mortality in Children Admitted for Suspected Malaria to a Pediatric Emergency Ward in a Low-Resource Setting: A Case-Control Study. Pediatr Crit Care Med 2018; 19(9): e479-e85.
230. Cawood S, Naidoo S, Okudo G, Velaphi S, Verwey C. Outcomes of paediatric patients ventilated in a high-care area outside an intensive care unit. S Afr Med J 2020; 110(9): 903-9.
231. Ceesay SJ, Casals-Pascual C, Erskine J, et al. Changes in malaria indices between 1999 and 2007 in The Gambia: a retrospective analysis. Lancet 2008; 372(9649): 1545-54.
232. Centers for Disease Control and Prevention. Post-earthquake injuries treated at a field hospital -- Haiti, 2010. MMWR: Morbidity & Mortality Weekly Report 2011; 59(51/52): 1673-7.
233. Cerqueira Santos A, Bezerra Góes FG, Pereira-Ávila FM, Amazonas Camilo L, Souza Bonifácio MC, de Azevedo Oliveira Knupp VnM. Clinical and epidemiological profile of children admitted to a pediatric unit. Revista Enfermagem UERJ 2020; 28: 1-9.
234. Chadha MS, Hirve S, Dawood FS, et al. Burden of Seasonal and Pandemic Influenza-Associated Hospitalization during and after 2009 A(H1N1)pdm09 Pandemic in a Rural Community in India. PLoS ONE 2013; 8(5).
235. Chami N, Kabyemera R, Ambrose E, et al. Prevalence and factors associated with renal dysfunction in children admitted to two hospitals in northwestern Tanzania. BMC Nephrology 2019; 20(1).
236. Chatterjee S, Verma V, Hazra A, Pal J. An observational study on acute poisoning in a tertiary care hospital in West Bengal, India. Perspectives in Clinical Research 2020; 11(2): 75-80.
237. Chavez D, Gonzales-Armayo V, Mendoza E, et al. Estimation of influenza and respiratory syncytial virus hospitalizations using sentinel surveillance data- La Paz, Bolivia. 2012-2017. Influenza and other Respiratory Viruses 2019; 13(5): 477-83.
238. Chekhlabi N, El Kettani C, Haoudar A, et al. The epidemiological and clinical profile of COVID-19 in children: Moroccan experience of the Cheikh Khalifa University Center. Pan Afr Med J 2020; 35(Suppl 2): 57.
239. Chelo D, Nguefack F, Mbassi Awa HD, et al. Premature death of children aged 2 months to 5 years: The case of the Mother and Child Center of the Chantal Biya Foundation, Yaounde, Cameroon. Translational Pediatrics 2016; 5(1): 23-30.
240. Chetan G, Rathisharmila R, Narayanan P, Mahadevan S. Acute respiratory distress syndrome in pediatric intensive care unit. Indian J Pediatr 2009; 76(10): 1013-6.
241. Cheung M, Kakembo N, Rizgar N, et al. Epidemiology and mortality of pediatric surgical conditions: insights from a tertiary center in Uganda. Pediatr Surg Int 2019; 35(11): 1279-89.
242. Chiesa PL, Aloi A, Andriani M, et al. Challenges, constraints and failures that are related to the posterior sagittal anorectoplasty approach to anorectal malformations in a low-resource context: An experience from a sudanese tertiary referral centre. Afr J Paediatr Surg 2020; 17(3 & 4): 79-84.
243. Chimhuya S, Kambarami RA, Mujuru H. The levels of malnutrition and risk factors for mortality at Harare Central Hospital-Zimbabwe: an observation study. Cent Afr J Med 2007; 53(5-8): 30-4.
244. Chouchane S, Fehri H, Chouchane C, et al. Hypernatremic dehydration in children: retrospective study of 105 cases. Arch Pediatr 2005; 12(12): 1697-702.
245. Chowdhury AN, Banerjee S, Brahma A, Biswas MK. A study on mortality and morbidity pattern of acute childhood poisoning cases admitted in block primary health centres of Sundarban, West Bengal. Indian J Public Health 2008; 52(1): 40-2.
246. Citron I, Amundson J, Saluja S, et al. Assessing burn care in Brazil: An epidemiologic, cross-sectional, nationwide study. Surgery 2018; 163(5): 1165-72.
247. Clifton DC, Ramadhani HO, Msuya LJ, et al. Predicting mortality for paediatric inpatients where malaria is uncommon. Arch Dis Child 2012; 97(10): 889-94.
248. Coetzee S, Morrow BM, Argent AC. Measles in a South African paediatric intensive care unit: again! J Paediatr Child Health 2014; 50(5): 379-85.
249. Collins J, Biaukula V, Faktaufon D, et al. An outbreak investigation of paediatric severe acute respiratory infections requiring admission to intensive care units - Fiji, May 2016. 2018; 1(2): 4-8.
250. Collins-Andrews B, McQuilkin P, Udhayashankar K, Adu E, Moormann A. Presentation and Treatment Outcomes of Liberian Children Age 5 Years and Under Diagnosed With Severe Malaria. Glob 2019; 6: 2333794X19884818.

251. Congdon M, Hong H, Young RR, et al. Effect of Haemophilus influenzae type b and 13-valent pneumococcal conjugate vaccines on childhood pneumonia hospitalizations and deaths in Botswana. Clinical infectious diseases: an official publication of the Infectious Diseases Society of America 2020.
252. Corten L, Morrow BM. Use of airway clearance therapy in children hospitalised with acute lower respiratory tract infections in a South African paediatric hospital. S 2020; 76(1): 1367.
253. Cortes J, Arvelo W, Lopez B, et al. Rotavirus disease burden among children <5 years of age--Santa Rosa, Guatemala, 2007-2009. Tropical Medicine & International Health 2012; 17(2): 254-9.
254. Costa de Santana M, Duarte Mello Amoedo C, Nascimento-Carvalho CM. Clinical and epidemiological characteristics of children admitted with fever in emergency department with or without sepsis. J 2017; 11(8): 597-603.
255. da Silva Cezar RD, da Silva Castanha PM, Matos Freire N, et al. Association between interferon lambda 3 rs12979860 polymorphism and clinical outcome in dengue virus-infected children. Int J Immunogenet 2020; 47(4): 351-8.
256. Daga SR, Klonin H, Ayarekar MD, Joshi SK, Janwale SB. Comparison of sepsis definitions in a resource poor environment. J Pediatr Intensive Care 2012; 1(3): 135-41.
257. Daga SR, Verma B, Gosavi DV, Kapse V, Shinde N. Declining seroprevalence of HIV infection among paediatric inpatients. Trop Doct 2007; 37(4): 233-5.
258. Daga S, Verma B, Shahane S, Janged S, Vachagan MM. Syndromic management of common illnesses in hospitalized children and neonates: a cost identification study. Indian J Pediatr 2010; 77(12): 1383-6.
259. Dagvadorj A, Ota E, Shahrook S, et al. Hospitalization risk factors for children's lower respiratory tract infection: A population-based, cross-sectional study in Mongolia. Sci 2016; 6: 24615.
260. Dangor Z, Izu A, Hillier K, et al. Impact of the antiretroviral treatment program on the burden of hospitalization for culture-confirmed tuberculosis in South African children: a time-series analysis. Pediatr Infect Dis J 2013; 32(9): 972-7.
261. Das MK, Arora NK, Poluru R, et al. Pediatric Appropriate Evaluation Protocol for India (PAEP-India): Tool for Assessing Appropriateness of Pediatric Hospitalization. Indian Pediatrics 2018; 55(12): 1041-5.
262. Das P, Sazzad HMS, Aleem MA, et al. Hospital-based zoonotic disease surveillance in Bangladesh: design, field data and difficulties. Philosophical transactions of the Royal Society of London Series B, Biological sciences 2019; 374(1782): 20190019.
263. Das SK, Reddy KS, Tamkeen SS, Chaudhary D, Mathew SE. Clinical study of lower respiratory tract infections (Lrti) in pediatrics attending a tertiary care teaching hospital. International Journal of Pharmaceutical Research 2020; 12(2): 529-35.
264. Dayanand KK, Kishore P, Chandrashekar V, et al. Malaria Severity in Mangaluru City in the Southwestern Coastal Region of India. Am J Trop Med Hyg 2019; 100(2): 275-9.
265. De Barros ENC, Nunes AA, Abreu ADJLD, et al. Pertussis epidemiological pattern and disease burden in Brazil: an analysis of national public health surveillance data. Human Vaccines and Immunotherapeutics 2019.
266. De Jesus MCS, Santos VS, Storti-Melo LM, et al. Impact of a twelve-year rotavirus vaccine program on acute diarrhea mortality and hospitalization in Brazil: 2006-2018. Expert Rev Vaccines 2020; 19(6): 585-93.
267. De Leon AL, Romero-Gutierrez G, Valenzuela CA, Gonzalez-Bravo FE. Simplified PRISM III score and outcome in the pediatric intensive care unit. Pediatr Int 2005; 47(1): 80-3.
268. de Lourdes Teixeira Masukawa M, Mayumi Moriwaki A, Getirana Santana R, Shozo Uchimura N, Teruya Uchimura T. Impact of oral human rotavirus vaccine on hospitalization rates for children. Acta Paulista de Enfermagem 2015; 28(3): 243-9.
269. de Luna D, Sánchez JJ, López M, et al. Antibiotic resistance profile in intrahospital pediatric services at third level centers in Dominican Republic. Infectio 2020; 24(2): 66-70.
270. de Martino Mota A, Carvalho-Costa FA. Varicella zoster virus related deaths and hospitalizations before the introduction of universal vaccination with the tetraviral vaccine. J Pediatr (Rio J) 2016; 92(4): 361-6.
271. de Moraes Bernal H, de Abreu LC, Bezerra IMP, et al. Incidence of hospitalization and mortality due to stroke in young adults, residents of developed regions in Brazil, 2008-2018. PLoS ONE 2020; 15(11 November).
272. de Oliveira Fernandes MA, Andreao WL, Maciel FM, de Almeida Albuquerque TT. Avoiding hospital admissions for respiratory system diseases by complying to the final Brazilian air quality standard: an estimate for Brazilian southeast capitals. Environ Sci Pollut Res Int 2020; 27(29): 35889-907.
273. de Oliveira TCR, Latorre MRDO. Trends in hospital admission and infant mortality from diarrhea: Brazil, 1995-2005. Revista de Saude Publica 2010; 44(1): 102-11.
274. de Rovetto Restrepo C, Mora JA, Cardona SA, Marmolejo AF, Paz JF, de Castaño I. Acute kidney injury applying pRifle scale in Children of Hospital Universitario del Valle in Cali, Colombia: Clinical features, management and evolution. Colombia Medica 2012; 43(3): 200-5.de Sena LWP, Mello A, Ferreira MVD, de Ataide MA, Dias RM, Vieira JLF. Doses of chloroquine in the treatment of malaria by Plasmodium vivax in patients between 2 and 14 years of age from the Brazilian Amazon basin. Malar J 2019; 18(1): 439.

275. de Siqueira Alves Lopes A, Fontes Vieira SC, Lima Santos Porto R, et al. Coronavirus disease-19 deaths among children and adolescents in an area of Northeast, Brazil: why so many? Trop Med Int Health 2021; 26(1): 115-9.
276. de Souza DC, Shieh HH, Barreira ER, et al. Epidemiology of Sepsis in Children Admitted to PICUs in South America. Pediatric Critical Care Medicine 2016; 17(8): 727-34.
277. De AMPS, Da CRJH, Mendes CMC. Temporal trends of overall mortality and hospital morbidity due to diarrheal disease in Brazilian children younger than 5 years from 2000 to 2010. Jornal de Pediatria 2013; 89(3): 315-25.
278. de-Paris F, Beck C, Pires MR, dos Santos RP, Kuchenbecker Rde S, Barth AL. Viral epidemiology of respiratory infections among children at a tertiary hospital in Southern Brazil. 2014; 1(2): 223-6.
279. Degiuseppe JI. Tendencia de los egresos hospitalarios por enfermedades infecciosas intestinales en ninos en Argentina, 2005-2013. Arch Argent Pediatr 2017; 115(4): 350-6.
280. Delgado Romero AK, Salazar Palechor YM, Dí-az R, et al. Factores pronósticos de la infección respiratoria aguda baja grave en menores de 5 años en Colombia. Revista Ciencias de la Salud 2017; 15(3): 313-24.
281. Descalzo MA, Clara W, Guzman G, et al. Estimating the burden of influenza-associated hospitalizations and deaths in Central America. Influenza other respi 2016; 10(4): 340-5.
282. Deshmukh T, Varma A, Damke S, Meshram R. Predictive Efficacy of Pediatric Logistic Organ Dysfunction-2 Score in Pediatric Intensive Care Unit of Rural Hospital. Indian J 2020; 24(8): 701-4.
283. Desyibelew HD, Baraki AG, Dadi AF. Mortality rate and predictors of time to death in children with severe acute malnutrition treated in Felege-Hiwot Referral Hospital Bahir Dar, Northwest Ethiopia. BMC Res Notes 2019; 12(1): 409.
284. Desyibelew HD, Fekadu A, Woldie H. Recovery rate and associated factors of children age 6 to 59 months admitted with severe acute malnutrition at inpatient unit of Bahir Dar Felege Hiwot Referral hospital therapeutic feeding unite, northwest Ethiopia. PLoS ONE 2017; 12(2): e0171020.
285. Deva A, Prasad SR, Madappa BP, Junjegowda K, Narayanaswaaswamy Bachu RP. Pneumococcal infections at a rural tertiary care hospital: A seven year study study on isolation rate, clinical spectrum and antibiogram. Journal of Clinical and Diagnostic Research 2014; 8(2): 50-2.
286. Dewi R, Silitonga FGM, Mangunatmadja I. Impact of albumin levels on clinical outcomes in children underwent abdominal surgery. Paediatrica Indonesiana 2020; 60(3): 148-52.
287. Diakite A, Bedi LA, Dano DS, Fall M. Acute poisoning in pediatric population in the Ivory Coast: A multicentre hospital-based study in Abidjan. Toxicologie Analytique et Clinique 2018; 30(2): 106-13.
288. Diallo T, Diallo A, Ouro-Gnagba A, Djagadou KA, Diara A. Profile of acute poisoning in the CHU-SO in Lomé (Togo). Toxicologie Analytique et Clinique 2020; 32(4): 258-65.
289. Dias CS, Mingoti SA, Ceolin APR, Dias MAS, Friche AAL, Caiaffa WT. The influence of climatic conditions on hospital admissions for asthma in children and adolescents living in Belo Horizonte, Minas Gerais, Brazil. Cienc 2020; 25(5): 1979-90.
290. Dini S, Douglas NM, Poespoprodjo JR, et al. The risk of morbidity and mortality following recurrent malaria in Papua, Indonesia: a retrospective cohort study. BMC Med 2020; 18(1): 28.
291. Diniz LMO, Maia MMM, De Oliveira YV, et al. Study of complications of varicella-zoster virus infection in hospitalized children at a reference hospital for infectious disease treatment. Hospital Pediatrics 2018; 8(7): 419-25.
292. Diop A, Thiongane A, Mwenda JM, et al. Impact of rotavirus vaccine on acute gastroenteritis in children under 5 years in Senegal: Experience of sentinel site of the Albert Royer Children's Hospital in Dakar. Vaccine 2018; 36(47): 7192-7.
293. Divecha C, Tullu MS, Chaudhary S. Burden of respiratory illnesses in pediatric intensive care unit and predictors of mortality: Experience from a low resource country. Pediatr Pulmonol 2019; 54(8): 1234-41.
294. Djadou KE, Kanassoua K, Douti K, Sédédji A, Atakouma DY, Agbr AD. Morbidity and mortality in teenagers at Tsévié's hospital (Togo). Journal de Pediatrie et de Puericulture 2010; 23(4): 191-7.
295. Dodo R, Zohoun A, Baglo T, Mehou J, Anani L. Emergency treatment of sickle cell diseases in the Blood Diseases Department at the Koutoukou Maga National Teaching Hospital, Cotonou, Benin. Pan Afr Med J 2018; 30: 192.
296. Dominguez Rojas J, Estupinan Vigil M, Garces-Ghilardi R, et al. Cross-sectional study of the clinical characteristics and outcomes of children hospitalized with COVID-19 in Lima, Peru. Medwave 2021; 21(1): e8107.
297. Dosi R, Jain G, Mehta A. Clinical Characteristics, Comorbidities,and Outcome among 365 Patients of Coronavirus Disease 2019 at a Tertiary Care Centre in Central India. J Assoc Physicians India 2020; 68(9): 20-3.
298. Douglas NM, Kenangalem E, Hasanuddin A, et al. Malaria-related hospitalization during childhood in Papua, Indonesia: A retrospective cohort study. PLoS ONE 2020; 15(1): e0228018.
299. Draz IH, Halawa EF, Wahby G, Ismail DK, Meligy BS. Pneumococcal infection among hospitalized Egyptian children. J Egypt Public Health Assoc 2015; 90(2): 52-7.
300. Drobac PC, Shin SS, Huamani P, et al. Risk factors for in-hospital mortality among children with tuberculosis: the 25-year experience in Peru. Pediatrics 2012; 130(2): e373-9.
301. Droussi H, Benchamkha Y, Ouahbi S, et al. Epidemiology and treatment of paediatric burns in a large children's hospital in Morocco: Analysis of 394 cases. African Journal of Emergency Medicine 2013; 3(3): 110-5.

302. du Toit-Prinsloo L, Saayman G. Fatal head injuries in children under the age of 5 years in Pretoria. Am J Forensic Med Pathol 2014; 35(3): 212-7.
303. Duangmala T, Lumbiganon P, Kosalaraksa P. Unusual clinical manifestations of dengue infection in children in a tertiary care hospital in northeast Thailand. Asian Biomedicine 2014; 8(1): 97-103.
304. Duarte MC, Amorim MR, Cuevas LE, Cabral-Filho JE, Correia JB. Risk factors for death from meningococcal infection in Recife, Brazil. J Trop Pediatr 2005; 51(4): 227-31.
305. Duffy C, Kenga DB, Gebretsadik T, et al. Multiple Concurrent Illnesses Associated with Anemia in HIV-Infected and HIV-Exposed Uninfected Children Aged 6-59 Months, Hospitalized in Mozambique. Am J Trop Med Hyg 2020; 102(3): 605-12.
306. Duke T, Pulsan F, Panauwe D, et al. Solar-powered oxygen, quality improvement and child pneumonia deaths: a large-scale effectiveness study. Arch Dis Child 2021; 106(3): 224-30.
307. Duong VT, The HC, Nhu TDH, et al. Genomic Serotyping, Clinical Manifestations, and Antimicrobial Resistance of Nontyphoidal Salmonella Gastroenteritis in Hospitalized Children in Ho Chi Minh City, Vietnam. J Clin Microbiol 2020; 58(12): 18.
308. E.J ALZ. Epidemiological and clinical features of hospitalized patients with febrile seizures in Ramadi, West of Iraq. Journal of Pediatric Neurology 2007; 5(4): 311-5.
309. Ebenezer K, Job V, Antonisamy B, et al. Serum Vitamin D Status and Outcome among Critically Ill Children Admitted to the Pediatric Intensive Care Unit in South India. Indian Journal of Pediatrics 2016; 83(2): 120-5.
310. Eberly LA, Rusingiza E, Park PH, et al. Understanding the Etiology of Heart Failure Among the Rural Poor in Sub-Saharan Africa: A 10-Year Experience From District Hospitals in Rwanda. J Card Fail 2018; 24(12): 849-53.
311. Ebissa G, Deyessa N, Biadgilign S. Predictors of early mortality in a cohort of HIV-infected children receiving high active antiretroviral treatment in public hospitals in Ethiopia. AIDS Care 2015; 27(6): 723-30.
312. Echavarria Medina A, Morales Uribe CH, Echavarria RL, Velez Marin VM, Martinez Montoya JA, Aguillon DF. Associated factors to non-operative management failure of hepatic and splenic lesions secondary to blunt abdominal trauma in children. Rev Chil Pediatr 2017; 88(4): 470-7.
313. Eckert MJ, Wertin TM, Tyner SD, Nelson DW, Izenberg S, Martin MJ. Tranexamic acid administration to pediatric trauma patients in a combat setting: the pediatric trauma and tranexamic acid study (PED-TRAX). J Trauma Acute Care Surg 2014; 77(6): 852-8; discussion 8.
314. Edwards D, Heard J, Latenser BA, Quinn KY, van Bruggen J, Jovic G. Burn injuries in eastern Zambia: impact of multidisciplinary teaching teams. J Burn Care Res 2011; 32(1): 31-8.
315. Egbohou P, Mouzou T, Tchetike P, et al. Epidemiology of Pediatric Traumatic Brain Injury at Sylvanus Olympio University Hospital of Lome in Togo. Anesthesiol Res Pract 2019; 2019: 4038319.
316. Eid R, Bakr A, Elmougy A, et al. Postdiarrheal hemolytic uremic syndrome in Egyptian children: An 11-year single-center experience. Saudi J Kidney Dis Transpl 2020; 31(6): 1376-87.
317. Ekere AU, Yellowe BE, Umune S. Mortality patterns in the accident and emergency department of an urban hospital in Nigeria. Niger J Clin Pract 2005; 8(1): 14-8.
318. El Fakiri K, Nassih H, Ait Sab I, Draiss G, Bouskraoui M. Epidemiology and Clinical Features of Coronavirus Disease 2019 in Moroccan Children. Indian Pediatrics 2020; 57(9): 808-10.
319. El Halal MG, Barbieri E, Filho RM, Trotta Ede A, Carvalho PR. Admission source and mortality in a pediatric intensive care unit. Indian J 2012; 16(2): 81-6.
320. El-Amin EO, Elbashir MI, Elamin OE, et al. The underlying aetiologies of coma in febrile Sudanese children. Trans R Soc Trop Med Hyg 2013; 107(5): 307-12.
321. El-Kholy A, Saied T, Gaber M, et al. Device-associated nosocomial infection rates in intensive care units at Cairo University hospitals: first step toward initiating surveillance programs in a resource-limited country. Am J Infect Control 2012; 40(6): e216-20.
322. El-Sherbini SA, Marzouk H, El-Sayed R, Hosam-ElDin S. Etiologia da hiperglicemia em crianças críticas e o impacto da disfunção de órgãos. Rev bras ter intensiva 2018; 30(3): 286-93.
323. Enawgaw B, Workineh Y, Tadesse S, Mekuria E, Addisu A, Genetu M. Prevalence of Anemia and Associated Factors Among Hospitalized Children Attending the University of Gondar Hospital, Northwest Ethiopia. eJIFCC 2019; 30(1): 35-47.
324. Ernest SK, Johnson AWBR, Mokuolu OA, et al. Acute respiratory infections in the middle-belt region of Nigeria. African Journal of Clinical and Experimental Microbiology 2014; 15(2): 51-9.
325. Esen AB, Erol M, Kafadar D, et al. Progression of Disease and Viral Agents in Infants Hospitalized for Lower Respiratory Tract Infections. Journal of Pediatric Research 2019; 6(4): 314-21.
326. Eskander AE, Sawires HK, Ebeid BA. Foreign body ingestion in Egyptian children: A 10-year experience of endoscopic intervention in a tertiary hospital. Minerva Pediatrica 2019; 71(5): 443-8.
327. Etuk IS, Anah MU, Eyong ME. Epidemiology and clinical features of glomerulonephritis in Calabar, Nigeria. Niger 2009; 24(2): 91-4.
328. Eyong K, Torty C, Asindi A, Ekanem E. Acute hemiplegia: aetiology and outcome in Nigerian children. Pan Afr Med J 2020; 36: 155.

329. Ezeofor IO, Garcia AL, Wright CM. Criteria for undernutrition screening in hospitalised infants under 6 months: a diagnostic accuracy study in a resource-poor setting. Arch Dis Child 2020; 105(6): 524-9.
330. Fardah Athiyyah A, Shigemura K, Kitagawa K, et al. Clinical manifestation of norovirus infection in children aged less than five years old admitted with acute diarrhea in Surabaya, Indonesia: a cross-sectional study. F1000Res 2019; 8: 2130.
331. Fardah Athiyyah A, Shigemura K, Kitagawa K, et al. Norovirus genogroup correlation with acute diarrhea severity in Indonesian pediatric patients aged 1-60 months: a cross-sectional study. F1000Res 2019; 8: 2130.
332. Faye PM, Sonko MA, Diop A, et al. Impact of 13-Valent Pneumococcal Conjugate Vaccine on Meningitis and Pneumonia Hospitalizations in Children aged <5 Years in Senegal, 2010-2016. Clin Infect Dis 2019; 69(Supplement_2): S66-S71.
333. Feasey NA, Everett D, Faragher EB, et al. Modelling the contributions of malaria, HIV, malnutrition and rainfall to the decline in paediatric invasive non-typhoidal Salmonella disease in Malawi. PLoS Neglected Tropical Diseases 2015; 9(7).
334. Feikin DR, Jagero G, Aura B, et al. High rate of pneumococcal bacteremia in a prospective cohort of older children and adults in an area of high HIV prevalence in rural western Kenya. BMC Infect Dis 2010; 10: 186.
335. Feikin DR, Ope MO, Aura B, et al. The population-based burden of influenza-associated hospitalization in rural western Kenya, 2007-2009. Bulletin of the World Health Organization 2012; 90(4): 256-63A.
336. Fekih Hassen A, Zayani MC, Friaa M, Trifa M, Ben Khalifa S. Epidemiology of pediatric traumatic brain injury at the Children's Hospital of Tunisia, 2007. Tunis Med 2012; 90(1): 25-30.
337. Feris-Iglesias J, Fernandez J, Sanchez J, et al. Aetiology of paediatric pneumonia with effusion in the Dominican Republic and the potential impact of pneumococcal conjugate vaccines. Pneumonia (Nathan) 2014; 4: 8-15.
338. Fernandes A, Avendanha FA, Viana MB. Hospitalizations of children with sickle cell disease in the Brazilian Unified Health System in the state of Minas Gerais. J Pediatr (Rio J) 2017; 93(3): 287-93.
339. Fernandes EG, Sato HK, Leshem E, et al. Impact of rotavirus vaccination on diarrhea-related hospitalizations in Sao Paulo State, Brazil. Vaccine 2014; 32(27): 3402-8.
340. Fernandes FM, Torquato IM, Dantas MS, Pontes Júnior FA, Ferreira JA, Collet N. Burn injuries in children and adolescents: clinical and epidemiological characterization. Revista gaúcha de enfermagem / EENFUFRGS 2012; 33(4): 133-41.
341. Fernandes MT, Danti GV, Garcia DM, Ferraro AA. Excess body weight in children may increase the length of hospital stay. Clinics 2015; 70(2): 87-90.
342. Fernandez JM, Dobrick JB, Jadavji A, Adam RD. Staphylococcus aureus bacteremia at a referral medical center in Kenya: A retrospective review of cases from 2010 to 2018. PLoS ONE 2020; 15(6): e0234914.
343. Fernández Ra, Lezama PM. Validación de dos escalas de valor pronóstico en niños que ingresan a UCI. Arch venez pueric pediatr 2010; 73(4): 3-7.
344. Ferrer Castro JE, Sánchez Hernández E, Poulout Mendoza A, del Río Caballero G, Figueredo Sánchez D. Caracterización clí-nica y epidemiológica de pacientes confirmados con la COVID-19 en la provincia de Santiago de Cuba. Medisan 2020; 24(3): 473-85.
345. Ferrer APS, Brentani AVM, Sucupira ACSL, Navega ACB, Cerqueira ES, Grisi SJFE. The effects of a people-centred model on longitudinality of care and utilization pattern of healthcare services--Brazilian evidence. Health Policy & Planning 2014; 29(suppl_2): ii107-ii13.
346. Fiestas Solórzano V, Sihuincha Maldonado M, Donaires Toscano F, et al. Caracterí-sticas clí-nicas de pacientes internados en el Hospital de Apoyo de Iquitos "César Garayar García" durante la epidemia de dengue, enero-febrero de 2011. Rev peru med exp salud publica 2011; 28(1): 78-82.
347. Finlayson H, Smit L, Esterhuizen TM, Kruger M. The impact of a change in referral pathway on a paediatric short-stay ward in Cape Town, South Africa. SAJCH South African Journal of Child Health 2016; 10(2): 134-8.
348. Firomsa T, Teferra M, Tadesse A. Trends and Outcomes of Emergency Pediatric Surgical Admissions from a Tertiary Hospital in Ethiopia. Ethiop 2018; 28(3): 251-8.
349. Fitzgerald E, Mlotha-Mitole R, Ciccone EJ, et al. A pediatric death audit in a large referral hospital in Malawi. BMC Pediatrics 2018; 18: 1-7.
350. Fitzgerald F, Wing K, Naveed A, et al. Development of a pediatric ebola predictive score, Sierra Leone. Emerging Infectious Diseases 2018; 24(2): 311-9.
351. Flasche S, Takahashi K, Vu DT, et al. Early indication for a reduced burden of radiologically confirmed pneumonia in children following the introduction of routine vaccination against Haemophilus influenzae type b in Nha Trang, Vietnam. Vaccine 2014; 32(51): 6963-70.
352. Fonseca JG, Ferreira AR. Application of the Pediatric Index of Mortality 2 in pediatric patients with complex chronic conditions. J Pediatr (Rio J) 2014; 90(5): 506-11.
353. Foran M, Ahn R, Novik J, et al. Prevalence of undiagnosed hypoxemia in adults and children in an under-resourced district hospital in Zambia. Int J Emerg Med 2010; 3(4): 351-6.
354. Forbinake NA, Ohandza CS, Fai KN, et al. Mortality analysis of burns in a developing country: a CAMEROONIAN experience. BMC Public Health 2020; 20(1): 1269.

355. Fortaleza C, Padoveze MC, Kiffer CRV, et al. Multi-state survey of healthcare-associated infections in acute care hospitals in Brazil. J Hosp Infect 2017; 96(2): 139-44.
356. Franciozi CEdS, Tamaoki MJS, Araújo EFAd, et al. Trauma na infância e adolescência: epidemiologia, tratamento e aspectos econômicos em um hospital público. Acta ortop bras 2008; 16(5): 261-5.
357. Freire KM, Bresolin NL, Farah AC, Carvalho FL, Goes JE. Acute kidney injury in children: incidence and prognostic factors in critical ill patients. Rev 2010; 22(2): 166-74.
358. Friedrich F, Ongaratto R, Scotta MC, et al. Early Impact of social distancing in response to COVID-19 on hospitalizations for acute bronchiolitis in infants in Brazil. Clin Infect Dis 2020; 28: 28.
359. Fuenmayor-Oramas F, González-Andrade F, la Cueva XPD. Moving Toward Non-invasive Hemodynamic and Echocardiographic Monitoring in Critical Pediatric Patients in the Emergency Department: Setting Standards. SN Comprehensive Clinical Medicine 2021.
360. Furia FF, Godfrey E, Mwamanenge N, Swai P. Spectrum of paediatric rheumatic disorders at a tertiary hospital in Tanzania. Pediatr 2020; 18(1): 30.
361. Galindo-Fraga A, Ortiz-Hernandez AA, Ramirez-Venegas A, et al. Clinical characteristics and outcomes of influenza and other influenza-like illnesses in Mexico City. Int J Infect Dis 2013; 17(7): e510-7.
362. Gallaher JR, Mjuweni S, Cairns BA, Charles AG. Burn care delivery in a sub-saharan african unit: A cost analysis study. Int J Surg 2015; 19: 116-20.
363. Gallaher JR, Wildfire B, Mabedi C, Cairns BA, Charles AG. Intentional injury against children in Sub-Saharan Africa: A tertiary trauma centre experience. Injury 2016; 47(4): 837-41.
364. Gallaher J, Purcell LN, Banda W, Reid T, Charles A. Re-evaluation of the Effect of Age on In-hospital Burn Mortality in a Resource-Limited Setting. J Surg Res 2021; 258: 265-71.
365. Gandhi J, Sangareddi S, Varadarajan P, Suresh S. Pediatric index of mortality 2 score as an outcome predictor in pediatric Intensive Care Unit in India. Indian J 2013; 17(5): 288-91.
366. Ganesh R, Janakiraman L, Vasanthi T, Sathiyasekeran M. Profile of typhoid fever in children from a tertiary care hospital in Chennai-South India. Indian J Pediatr 2010; 77(10): 1089-92.
367. Garrido Salazar DI, Fuseau M, Garrido S, Vivas G, Gutiérrez M. Prevalence of anaemia in children diagnosed with pneumonia in a tertiary hospital in Quito, Ecuador. Journal of Nepal Paediatric Society 2018; 38(2): 102-9.
368. Gathara D, Irimu G, Kihara H, et al. Hospital outcomes for paediatric pneumonia and diarrhoea patients admitted in a tertiary hospital on weekdays versus weekends: a retrospective study. BMC Pediatr 2013; 13: 74.
369. Gauchan E, Malla T, Basnet S, Rao KS. Variability of presentations and CT-scan findings in children with neurocysticercosis. Kathmandu Univ 2011; 9(34): 17-21.
370. Gavhi F, Kuonza L, Musekiwa A, Motaze NV. Factors associated with mortality in children under five years old hospitalized for Severe Acute Malnutrition in Limpopo province, South Africa, 2014-2018: A cross-sectional analytic study. PLoS ONE 2020; 15(5): e0232838.
371. Gawryszewski VP, Rodrigues EMS. The burden of injury in Brazil, 2003. São Paulo med j 2006; 124(4): 208-13.
372. Gay-Andrieu F, Adehossi E, Lacroix V, et al. Epidemiological, clinical and biological features of malaria among children in Niamey, Niger. Malar J 2005; 4: 10.
373. Gebregziabher Kahsay A, Abebew D. Occurrence of zoonotic and other infectious bacterial organisms in Dessie Regional Health Laboratory, South Wollo, Ethiopia: A retrospective study on medical records. International Journal of Tropical Medicine 2013; 8(2): 39-43.
374. George EC, Walker AS, Kiguli S, et al. Predicting mortality in sick African children: the FEAST Paediatric Emergency Triage (PET) Score. BMC Med 2015; 13: 174.
375. Ghani AS, Morrow BM, Hardie DR, Argent AC. An investigation into the prevalence and outcome of patients admitted to a pediatric intensive care unit with viral respiratory tract infections in Cape Town, South Africa. Pediatr Crit Care Med 2012; 13(5): e275-81.
376. Ghazawy ER, Bebars GM, Eshak ES. Survival status and mortality predictors among severely malnourished under 5 years of age children admitted to Minia University maternity and children hospital. BMC Pediatrics 2020; 20(1).
377. Gheshlaghi F, Piri-Ardakani MR, Yaraghi M, Shafiei F, Behjati M. Acute poisoning in children; a population study in isfahan, iran, 2008-2010. Iran 2013; 23(2): 189-93.
378. Giorno EPC, De Paulis M, Sameshima YT, et al. Point-of-care lung ultrasound imaging in pediatric COVID-19. Ultrasound J 2020; 12(1): 50.
379. Giri P, Roy J, Saha A. Scrub Typhus - A Major Cause of Pediatric Intensive Care Admission and Multiple Organ Dysfunction Syndrome: A Single-Center Experience from India. Indian Journal of Critical Care Medicine 2018; 22(2): 107-10.
380. Godlwana L, Gounden P, Ngubo P, Nsibande T, Nyawo K, Puckree T. Incidence and profile of spinal tuberculosis in patients at the only public hospital admitting such patients in KwaZulu-Natal. Spinal Cord 2008; 46(5): 372-4.

381. Goel AK, Chawla S, Dhingra A, Thiyagarajan V, Nair NP. Rotavirus Diarrhea and its Determinants Among Under-Five Children Admitted in a Tertiary Care Hospital of Southern Haryana, India. Indian J Pediatr 2021; 27: 27.
382. Goossens S, Bekele Y, Yun O, Harczi G, Ouannes M, Shepherd S. Mid-upper arm circumference based nutrition programming: evidence for a new approach in regions with high burden of acute malnutrition. PLoS ONE 2012; 7(11): e49320.
383. Grando LR, Schmitt TA, Bakos RM. Severe cutaneous reactions to drugs in the setting of a general hospital. 2014; 1(5): 758-62.
384. Gray DM, Davies MA, Githinji L, et al. COVID-19 and Pediatric Lung Disease: A South African Tertiary Center Experience. Front 2020; 8: 614076.
385. Gregianini TS, Varella IRS, Fisch P, Martins LG, Veiga ABG. Dual and Triple Infections with Influenza A and B Viruses: A Case-Control Study in Southern Brazil. Journal of Infectious Diseases 2019; 220(6): 961-8.
386. Grellety E, Krause LK, Shams Eldin M, Porten K, Isanaka S. Comparison of weight-for-height and mid-upper arm circumference (MUAC) in a therapeutic feeding programme in South Sudan: is MUAC alone a sufficient criterion for admission of children at high risk of mortality? 2015; 1(14): 2575-81.
387. Grenov B, Lanyero B, Nabukeera-Barungi N, et al. Diarrhea, Dehydration, and the Associated Mortality in Children with Complicated Severe Acute Malnutrition: A Prospective Cohort Study in Uganda. J Pediatr 2019; 210: 26-33.e3.
388. Griffiths MJ, Lemon JV, Rayamajhi A, et al. The functional, social and economic impact of acute encephalitis syndrome in Nepal--a longitudinal follow-up study. PLoS Negl Trop Dis 2013; 7(9): e2383.
389. Groome MJ, Madhi SA. Five-year cohort study on the burden of hospitalisation for acute diarrhoeal disease in African HIV-infected and HIV-uninfected children: potential benefits of rotavirus vaccine. Vaccine 2012; 30 Suppl 1: A173-8.
390. Groome MJ, Moyes J, Cohen C, et al. Human metapneumovirus-associated severe acute respiratory illness hospitalisation in HIV-infected and HIV-uninfected South African children and adults. J Clin Virol 2015; 69: 125-32.
391. Groome MJ, Zell ER, Solomon F, et al. Temporal Association of Rotavirus Vaccine Introduction and Reduction in All-Cause Childhood Diarrheal Hospitalizations in South Africa. Clinical Infectious Diseases 2016; 62: S188-S95.
392. Guelmo E, Jones A, Ojiegbe J, Vanterpool H, Williams C, Mungrue K. Epidemiological trends of Kawasaki disease among paediatric patients admitted to the Wendy Fitzwilliam Paediatric Hospital, Trinidad, during 2004-2007. Trans R Soc Trop Med Hyg 2009; 103(8): 823-6.
393. Guerrier G, Goyet S, Chheng ET, et al. Acute viral lower respiratory tract infections in Cambodian children: clinical and epidemiologic characteristics. Pediatr Infect Dis J 2013; 32(1): e8-13.
394. Guevara JN, López Ó, González G. Impact of rotavirus vaccine introduction on hospital admissions for severe acute gastroenteritis at the Children's Hospital in Panama City. Revista Panamericana de Salud Publica 2008; 24(3): 189-94.
395. Guigñan O, Centritto C C, Arias F, Reyes Á. Aplicación de la escala de riesgo de mortalidad pediátrica (PRISM): en una unidad de terapia intensiva pediátrica venezolana. Arch venez pueric pediatr 2007; 70(4): 126-9.
396. Gupt A, Bhatnagar T, Murthy BN. Epidemiological profile and management of snakebite cases - A cross sectional study from Himachal Pradesh, India. Clinical Epidemiology and Global Health 2015; 3: S96-S100.
397. Gupta A, Kapil A, Lodha R, et al. Burden of healthcare-associated infections in a paediatric intensive care unit of a developing country: a single centre experience using active surveillance. J Hosp Infect 2011; 78(4): 323-6.
398. Gupta ML, Gothwal S, Gupta RK, et al. Duration of Viral Clearance in Children With SARS-CoV-2 Infection in Rajasthan, India. Indian pediatrics 2020.
399. Gupta ML, Gothwal S, Gupta RK, et al. Duration of Viral Clearance in Children With SARS-CoV-2 Infection in Rajasthan, India. Indian Pediatr 2021; 58(2): 123-5.
400. Gupta M, Kanojia R, Singha R, et al. Intussusception Rate Among Under-Five-Children Before Introduction of Rotavirus Vaccine in North India. J Trop Pediatr 2018; 64(4): 326-35.
401. Gupta PP, Malla GB, Bhandari R, Kalawar RPS, Mandal M. Patterns of Injury and Mortality in Pediatric Patients Attending Emergency Department in a Tertiary Care Center in Eastern Nepal. Jnm 2017; 56(207): 331-4.
402. Gupta V, Aggarwal P, Kumar B, Nair NP, Thiyagarajan V, Lingam R. Rotavirus Gastroenteritis in Eastern Uttar Pradesh, India. Indian J Pediatr 2021; 18: 18.
403. Gurgel RQ, Alvarez Ade J, Rodrigues A, et al. Incidence of rotavirus and circulating genotypes in Northeast Brazil during 7 years of national rotavirus vaccination. PLoS ONE 2014; 9(10): e110217.
404. Gurgel RQ, Ilozue C, Correia JB, Centenari C, Oliveira SM, Cuevas LE. Impact of rotavirus vaccination on diarrhoea mortality and hospital admissions in Brazil. Trop Med Int Health 2011; 16(9): 1180-4.
405. Gutiérrez-Vázquez I, Burguete-Cabañas T, Zacarías-Castillo R, Mata-Miranda P. Mortalidad por diabetes mellitus: experiencia de 11 años en un hospital de segundo nivel. Salud pública Méx 2006; 48(6): 445-
406. Guzmán MaG, Peláez O, Kourí- G, et al. Caracterización final y lecciones de la epidemia de dengue 3 en Cuba, 2001-2002. Rev panam salud publica 2006; 19(4): 282-9.
407. Habib MI, Khan KMA. Profile and outcomes of critically ill children in a lower middle-income country. Emerg Med J 2018; 35(1): 52-5.

408. Habouchi S, Bouamra A, Bezzaoucha A, Joucdar S. Estimation of survival rate in electrical injuries, experience in Algerian Burn Centers. Burns Open 2020; 4(4): 141-5.
409. Haddadin Z, Chappell J, McHenry R, et al. Coronavirus Surveillance in a Pediatric Population in Jordan from 2010 to 2013: A Prospective Viral Surveillance Study. Pediatric Infectious Disease Journal 2020: 12-7.
410. Haddadin Z, Chappell J, McHenry R, et al. Coronavirus Surveillance in a Pediatric Population in Jordan From 2010 to 2013: A Prospective Viral Surveillance Study. Pediatr Infect Dis J 2021; 40(1): e12-e7.
411. Hafiz MG, Mannan MA, Amin SK, Islam A, Rahman F. Immune thrombocytopenic purpura among the children attending at two teaching hospitals. Bangladesh Med Res Counc Bull 2008; 34(3): 94-8.
412. Haftu H, Hailu T, Medhaniye A, T GT. Assessment of pattern and treatment outcome of patients admitted to pediatric intensive care unit, Ayder Referral Hospital, Tigray, Ethiopia, 2015. BMC Res Notes 2018; 11(1): 339.
413. Hagos M. Pattern of acute appendicitis in Mekelle, Ethiopia. Ethiop Med J 2014; 52(3): 113-8.
414. Haidar NA, Emran MY, Al Muslemani EA. Snakebites in Hajjah, Yemen Republic: Epidemiology, management and the relation of the degree of acuity at presentation with outcome. Journal of Emergency Medicine, Trauma and Acute Care 2012.
415. Haider N, Nagi AG, Khan KM. Frequency of nutritional rickets in children admitted with severe pneumonia. JPMA J Pak Med Assoc 2010; 60(9): 729-32.
416. Haider N, Shaikh AS, Wazir R, Atiq M. Clinicodemographic features and outcome of acute myocarditis in children admitted at tertiary care hospital. Int J Cardiol 2016; 221: 42-5.
417. Hall-Parkinson D, Tapper J, Melbourne-Chambers R. Parent and caregiver knowledge, beliefs, and responses to convulsive seizures in children in Kingston, Jamaica - A hospital-based survey. Epilepsy Behav 2015; 51: 306-11.
418. Hallbauer UM, Joubert G, Goosen Y. Pertussis in children in Bloemfontein, South Africa: A 7-year retrospective review. Samj, S 2016; 106(10): 1042-6.
419. Hamele M, Aden JK, Borgman MA. Tranexamic acid in pediatric combat trauma requiring massive transfusions and mortality. J Trauma Acute Care Surg 2020; 89(2S Suppl 2): S242-S5.
420. Hansson HH, Turner L, Moller L, et al. Haplotypes of the endothelial protein C receptor (EPCR) gene are not associated with severe malaria in Tanzania. Malar J 2015; 14: 474.
421. Haque A, Bano S. Clinical profile and outcome in a paediatric intensive care unit in Pakistan. J Coll Physicians Surg Pak 2009; 19(8): 534-5.
422. Haque A, Bano S. Improving outcome in pediatric intensive care unit in academic hospital in Pakistan. Pakistan Journal of Medical Sciences 2009; 25(4): 605-8.
423. Haque A, Saleem AF. On admission hypomagnesemia in critically ill children: Risk factors and outcome. Indian J Pediatr 2009; 76(12): 1227-30.
424. Haque A, Siddiqui NR, Jafri SK, Hoda M, Bano S, Mian A. Clinical profiles and outcomes of children admitted to the pediatric intensive care unit from the emergency department. J Coll Physicians Surg Pak 2015; 25(4): 301-3.
425. Haque A, Siddiqui NU, Kumar R, Hoda M, Lakahni G, Hooda K. Cost of care in a paediatric intensive care unit of a tertiary-care university hospital of Pakistan. JPMA J Pak Med Assoc 2015; 65(6): 651-4.
426. Haroun HM, Mahfouz MS, Elhaj AM. Patterns of childhood cancer in children admitted to the institute of nuclear medicine, molecular biology and oncology (inmo), wad medani, gezira state. J Family Community Med 2006; 13(2): 71-4.
427. Harunani S, Imbaya K, Kuremu T. Non-traumatic abdominal surgical emergencies in children. Annals of African Surgery 2020; 17(1): 3-6.
428. Hasan J, Das SK. Early Hospitalization Reduces the Length-of-Hospital Stay and Antibiotic Use in Children With Moderate Diarrhea: A Study in Bangladesh. Journal of Pediatrics Review 2021; 9(1): 71-9.
429. Hassan R, El-Gilany AH, Abd elaal AM, El-Mashad N, Azim DA. An overview of healthcare-associated infections in a tertiary care hospital in Egypt. Infection Prevention in Practice 2020; 2(3).
430. Hategeka C, Shoveller J, Tuyisenge L, Lynd LD. Assessing process of paediatric care in a resource-limited setting: a cross-sectional audit of district hospitals in Rwanda. Paediatr Int Child Health 2018; 38(2): 137-45.
431. Hawa Cheikh D, Aboussaleh Y, Sbaibi R, Bengueddour R. Evaluation of the care of infants and children under 5 years with severe undernutrition at the CRENAS of Nouakchott, Mauritania. Nutritional Therapy & Metabolism 2014; 32(3): 140-5.
432. Hawkes MT, Opoka RO, Conroy AL, et al. Anemia and transfusion requirements among Ugandan children with severe malaria treated with intravenous artesunate. Pediatr Hematol Oncol 2020; 37(2): 140-52.
433. Hazard RH, Chowdhury HR, Adair T, et al. The quality of medical death certification of cause of death in hospitals in rural Bangladesh: impact of introducing the International Form of Medical Certificate of Cause of Death. BMC Health Serv Res 2017; 17(1): 688.
434. He S, Lunnen JC, Puvanachandra P, Amar S, Zia N, Hyder AA. Global childhood unintentional injury study: multisite surveillance data. Am J Public Health 2014; 104(3): e79-84.
435. Hengstermann S, Mantaring 3rd JB, Sobel HL, et al. Formula feeding is associated with increased hospital admissions due to infections among infants younger than 6 months in Manila,

Philippines. Journal of human lactation : official journal of International Lactation Consultant Association 2010; 26(1): 19-25.
436. Hercik C, Cosmas L, Mogeni OD, et al. A diagnostic and epidemiologic investigation of acute febrile illness (AFI) in Kilombero, Tanzania. PLoS ONE 2017; 12(12): e0189712.
437. Herdman MT, Maude RJ, Chowdhury Md S, et al. The relationship between poverty and healthcare seeking among patients hospitalized with acute febrile illnesses in chittagong, Bangladesh. PLoS ONE 2016; 11(4).
438. Herle K, Jehangir S, Thomas R. Stricture urethra in children: An Indian perspective. Journal of Indian Association of Pediatric Surgeons 2018; 23(4): 192-7.
439. Herr W, Krumkamp R, Hogan B, et al. A cross-sectional study on risk factors for infection with Parvovirus B19 and the association with anaemia in a febrile paediatric population in Ghana. Sci 2020; 10(1): 15695.
440. Hiddou A, Hamdani H, Elmouaych I, et al. Evaluation of the prescription of antibiotics in the pediatric emergencies at the university hospital of Marrakech. Journal de Pediatrie et de Puericulture 2018; 31(1): 34-9.
441. Hirve S, Krishnan A, Dawood FS, et al. Incidence of influenza-associated hospitalization in rural communities in western and northern India, 2010-2012: a multi-site population-based study. J Infect 2015; 70(2): 160-70.
442. Hodkinson P, Argent A, Wallis L, et al. Pathways to Care for Critically Ill or Injured Children: A Cohort Study from First Presentation to Healthcare Services through to Admission to Intensive Care or Death. PLoS ONE 2016; 11(1): e0145473.
443. Hoffmann-Santos HD, Paula CR, Yamamoto AC, Tadano T, Hahn RC. Six-year trend analysis of nosocomial candidemia and risk factors in two intensive care hospitals in Mato Grosso, midwest region of Brazil. Mycopathologia 2013; 176(5-6): 409-15.
444. Hogan B, Ammer L, Zimmermann M, et al. Burden of influenza among hospitalized febrile children in Ghana. Influenza other respi 2017; 11(6): 497-501.
445. Homaira N, Luby SP, Hossain K, et al. Respiratory Viruses Associated Hospitalization among Children Aged <5 Years in Bangladesh: 2010-2014. PLoS ONE 2016; 11(2): e0147982.
446. Hooli S, King C, Zadutsa B, et al. The Epidemiology of Hypoxemic Pneumonia among Young Infants in Malawi. Am J Trop Med Hyg 2020; 102(3): 676-83.
447. Hossain MI, Dodd NS, Ahmed T, et al. Experience in managing severe malnutrition in a government tertiary treatment facility in Bangladesh. 2009; 1(1): 72-9.
448. Houngbadji MSTS, Thiongane A, Faye PM, et al. Cardio-respiratory cessation of child. Epidemiological, therapeutic and prognostic aspects in Senegal. Medecine d'Afrique Noire 2018; 65(3): 137-45.
449. Hsia RY, Ozgediz D, Jayaraman S, Kyamanywa P, Mutto M, Kobusingye OC. Epidemiology of child injuries in Uganda: challenges for health policy. J 2011; 2(1): e15.
450. Hsia RY, Ozgediz D, Mutto M, Jayaraman S, Kyamanywa P, Kobusingye OC. Epidemiology of injuries presenting to the national hospital in Kampala, Uganda: implications for research and policy. Int J Emerg Med 2010; 3(3): 165-72.
451. Hyginus EO, Okechukwu UJ, Victor IM, Christian OC, Anthony U. Epidemiology of admitted cases of childhood injuries in Nnamdi Azikiwe University Teaching Hospital Nnewi, Nigeria. Annals of Tropical Medicine and Public Health 2015; 8(6): 272-5.
452. Ifa AC. Trend in malaria prevalence among children under five years of age in the Hadiya zone, southern Ethiopia: A five-year retrospective study. Family Medicine and Primary Care Review 2018; 20(4): 337-40.
453. Ikealumba NV, Couper ID. Suicide and attempted suicide: the Rehoboth experience. Rural Remote Health 2006; 6(4): 535.
454. Iliyasu Z, Abubakar IS, Gajida AU. Magnitude and leading causes of in-hospital mortality at Aminu Kano Teaching Hospital, Kano, northern Nigeria: a 4-year prospective analysis. Niger J Med 2010; 19(4): 400-6.
455. Imani P, Odiit A, Hingorani S, Weiss N, Eddy A. Acute kidney injury and its association with in-hospital mortality among children with acute infections. Pediatric Nephrology 2013; 28(11): 2199-206.
456. Iqbal T, Saaiq M, Ali Z. Epidemiology and outcome of burns: early experience at the country's first national burns centre. Burns 2013; 39(2): 358-62.
457. Irola Moya JC, Garro Morales M. Pediatric palliative care, Costa Rica's experience. Am J Hosp Palliat Care 2010; 27(7): 456-64.
458. Ishak AA, Al-Jayefy NH, Raja'a YA. Profile of paediatric emergency cases in Yemen: the need for evidence-based guidelines. East Mediterr Health J 2008; 14(2): 366-75.
459. Isiguzo CM, Opara C, Nnadozie UU, Opara K. Burn injury in tertiary health facility in South East Nigeria: A 2 year prospective study. Burns Open 2020; 4(4): 153-7.
460. Ismail Y, Ahamed HN, Vijaya Vara Prasad M. Retrospective analysis of symptoms and outcomes of snakebite cases. International Journal of Research in Pharmaceutical Sciences 2020; 11(1): 1051-4.
461. Issiaka D, Barry A, Traore T, et al. Impact of seasonal malaria chemoprevention on hospital admissions and mortality in children under 5 years of age in Ouelessebougou, Mali. Malar J 2020; 19(1): 103.
462. Iyer S, Patel G. Study of risk factors, clinical spectrum, and outcome for head injury in pediatric age group in Western India. Afr J Paediatr Surg 2020; 17(1-2): 26-32.

463. Jacomin Vr, Cruz Shibukawa BM, Ieda Harumi H. INFANT HOSPITALIZATION BY PRIMARY CARE'S SENSITIVE CONDITIONS IN A SOUTHERN BRAZILIAN STATE. Revista de Pesquisa: Cuidado e Fundamental 2020; 12(1): 958-64.
464. Jaffry Z, Chokotho LC, Harrison WJ, Mkandawire NC. The burden of trauma at a district hospital in Malawi. Trop Doct 2017; 47(4): 286-91.
465. Jagnoor J, Keay L, Ganguli A, et al. Fall related injuries: a retrospective medical review study in North India. Injury 2012; 43(12): 1996-2000.
466. Jaime-Perez JC, Fernandez LT, Jimenez-Castillo RA, et al. Hospitalization rate and costs in acute lymphoblastic leukemia of childhood in a low-income group: Financial impact in Northeast Mexico. Pediatr Blood Cancer 2017; 64(12).
467. Jain A, Shah D, Das S, Saha R, Gupta P. Aetiology and outcome of acute diarrhoea in children with severe acute malnutrition: a comparative study. Public Health Nutr 2020; 23(9): 1563-8.
468. Jaipuria J, Sagar S, Singhal M, et al. Paediatric extremity vascular injuries - experience from a large urban trauma centre in India. Injury 2014; 45(1): 176-82.
469. Jaiswal N, Singh M, Thumburu KK, et al. Burden of invasive pneumococcal disease in children aged 1 month to 12 years living in South Asia: a systematic review. PLoS ONE 2014; 9(5): e96282.
470. Jakoet MS, Burger M, Van Heukelum M, et al. The epidemiology and orthopaedic burden of civilian gunshot injuries over a four-year period at a level one trauma unit in Cape Town, South Africa. International Orthopaedics 2020; 44(10): 1897-904.
471. Jali AGP, Nkambule BB. Prevalence and aetiology of moderate and severe thrombocytopenia in a tertiary and quaternary centre in KwaZulu-Natal. Afr 2020; 9(1): 799.
472. Jamil MT, Dhanani Z, Abbas Q, Jurair H, Mahar FK, Haque A. Transfusion-Related Acute Lung Injury In A Paediatric Intensive Care Unit Of Pakistan. J Ayub Med Coll Abbottabad 2017; 29(4): 702-5.
473. Jarnheimer A, Kantor G, Bickler S, Farmer P, Hagander L. Frequency of surgery and hospital admissions for communicable diseases in a high- and middle-income setting. Br J Surg 2015; 102(9): 1142-9.
474. Veeraraghavan B, Chethrapilly Purushothaman GK, et al. Burden of bacterial meningitis in India: Preliminary data from a hospital based sentinel surveillance network. PLoS ONE 2018; 13(5).
475. Jayashree M, Singhi S. Changing trends and predictors of outcome in patients with acute poisoning admitted to the intensive care. J Trop Pediatr 2011; 57(5): 340-6.
476. Jeandron A, Cumming O, Rumedeka BB, Saidi JM, Cousens S. Confirmation of cholera by rapid diagnostic test amongst patients admitted to the cholera treatment centre in Uvira, Democratic Republic of the Congo. PLoS ONE 2018; 13(8): e0201306.
477. Jegin P, Dinesh Kumar M, Dayana J, Elakiya E, Subbulakshmi S. A retrospective study to compute the prevalence of pneumonia and its associated risk factors among children in selected tertiary care hospital, Kelambakkam, Kancheepuram, Tamilnadu, India. Indian Journal of Public Health Research and Development 2020; 11(3): 136-8.
478. Jenney A, Tikoduadua L, Buadromo E, et al. The burden of hospitalised rotavirus infections in Fiji. Vaccine 2009; 27(SUPPL. 5): F108-F11.
479. Jerome E, Laing GL, Bruce JL, Sartorius B, Brysiewicz P, Clarke DL. An audit of traumatic brain injury (TBI) in a busy developing-world trauma service exposes a significant deficit in resources available to manage severe TBI. Samj, S 2017; 107(7): 621-5.
480. Jhamb U, Chawla V, Khanna S. Clinical profile of group A meningococcal outbreak in Delhi. Indian Pediatr 2009; 46(9): 794-6.
481. Jimbo Sotomayor R, Toscano CM, Sanchez Choez X, et al. Impact of pneumococcal conjugate vaccine on pneumonia hospitalization and mortality in children and elderly in Ecuador: Time series analyses. Vaccine 2020; 38(45): 7033-9.
482. Jimbo-Sotomayor R, Armijos-Acurio L, Proano-Espinosa J, Segarra-Galarza K, Sanchez-Choez X. Morbidity and Mortality Due to Pneumococcal Disease in Children in Ecuador from 2005 to 2015. J Glob Infect Dis 2020; 12(3): 124-8.
483. Jiraphongsa C, Bresee JS, Pongsuwanna Y, et al. Epidemiology and burden of rotavirus diarrhea in Thailand: Results of sentinel surveillance. Journal of Infectious Diseases 2005; 192(SUPPL. 1): S87-S93.
484. Joshi P, Agrawal S, Sah UP. Study of morbidity and mortality pattern of children admitted in paediatric intensive care unit of tertiary care children’s hospital. Journal of Nepal Paediatric Society 2020; 40(3): 232-40.
485. Jroundi I, Benmessaoud R, Mahraoui C, et al. Antibiotic Usage Prior and During Hospitalization for Clinical Severe Pneumonia in Children under Five Years of Age in Rabat, Morocco. Antibiotics (Basel) 2013; 2(4): 450-64.
486. Jugmohan B, Loveland J, Doedens L, Moore RL, Welthagen A, Westgarth-Taylor CJ. Mortality in paediatric burns victims: A retrospective review from 2009 to 2012 in a single centre. Samj, S 2016; 106(2): 189-92.
487. Jullien S, Pradhan D, Tshering T, et al. Pneumonia in children admitted to the national referral hospital in Bhutan: A prospective cohort study. Int J Infect Dis 2020; 95: 74-83.

488. Junqueira RM, Duarte EC. Hospitalizations due to ambulatory care-sensitive conditions in the Federal District, Brazil, 2008. [Portuguese]. 2012; 1(5): 761-8.
489. Junqueira RMP, Duarte EC. Internações hospitalares por causas sensíveis à atenção primária no Distrito Federal, 2008. Rev saúde pública 2012; 46(5): 761-8.
490. Justin-Temu M, Rimoy G, Premji Z, Matemu G. Causes, magnitude and management of burns in under-fives in district hospitals in Dar es Salaam, Tanzania. East Afr J Public Health 2008; 5(1): 38-42.
491. Kahl G, Hallbauer UM, Joubert G. Clinical presentation of infants hospitalised with pertussis. SAJCH South African Journal of Child Health 2016; 10(3): 176-80.
492. Kakembo N, Grabski DF, Fitzgerald TN, et al. Burden of Surgical Infections in a Tertiary-Care Pediatric Surgery Service in Uganda. Surg Infect (Larchmt) 2019; 27: 27.
493. Kakupa DK, Muenze PK, Byl B, Wilmet MD. Study of the prevalence of nosocomial infections and associated factors in the two university hospitals of Lubumbashi, Democratic Republic of Congo. Pan Afr Med J 2016; 24: 275.
494. Kamath SR, Ranjit S. Clinical features, complications and atypical manifestations of children with severe forms of dengue hemorrhagic fever in South India. Indian journal of pediatrics 2006; 73(10): 889-95.
495. Kambale RM, Kasengi JB, Kivukuto JM, Cubaka LM, Mungo BM, Balaluka GB. Infectious profile and mortality of children aged 0-5 years admitted with severe acute malnutrition: a retrospective cohort study conducted in the Therapeutic Nutritional Center in Bukavu, Republic of Congo. Pan Afr Med J 2016; 23: 139.
496. Kambale RM, Ngaboyeka GA, Ntagazibwa JN, et al. Severe acute malnutrition in children admitted in an Intensive Therapeutic and Feeding Centre of South Kivu, Eastern Democratic Republic of Congo: Why do our patients die? PLoS ONE 2020; 15(7 July).
497. Kampondeni S, Seydel KB, Zhang B, et al. Amount of Brain Edema Correlates With Neurologic Recovery in Pediatric Cerebral Malaria. Pediatr Infect Dis J 2020; 39(4): 277-82.
498. Kang JM, Lee J, Kim YK, et al. Pediatric intensive care unit admission due to respiratory syncytial virus: Retrospective multicenter study. Pediatrics International 2019; 61(7): 688-96.
499. Kannan VC, Andriamalala CN, Reynolds TA. The burden of acute disease in Mahajanga, Madagascar - a 21 month study. PLoS ONE 2015; 10(3): e0119029.
500. Kanwal SK, Bando A, Kumar V. Clinical profile of diabetic ketoacidosis in Indian children. Indian J Pediatr 2012; 79(7): 901-4.
501. Kapesa A, Basinda N, Nyanza EC, et al. Malaria Morbidities Following Universal Coverage Campaign for Long-Lasting Insecticidal Nets: A Case Study in Ukerewe District, Northwestern Tanzania. Res 2020; 11: 53-60.
502. Kapesa A, Kweka EJ, Atieli H, et al. The current malaria morbidity and mortality in different transmission settings in Western Kenya. 2018; 1(8): e0202031.
503. Kariuki SM, Chengo E, Ibinda F, et al. Burden, causes, and outcomes of people with epilepsy admitted to a rural hospital in Kenya. Epilepsia 2015; 56(4): 577-84.
504. Karki B, Rai SM, Nakarmi KK, et al. Clinical Epidemiology of Acute Burn Injuries at Nepal Cleft and Burn Centre, Kathmandu, Nepal. Ann Plast Surg 2018; 80(3 Suppl 2): S95-S7.
505. Kastor A, Mohanty SK. Disease and age pattern of hospitalisation and associated costs in India: 1995-2014. BMJ Open 2018; 8(1): e016990.
506. Kasundriya SK, Dhaneria M, Mathur A, Pathak A. Incidence and risk factors for severe pneumonia in children hospitalized with Pneumonia in Ujjain, India. International Journal of Environmental Research and Public Health 2020; 17(13): 1-15.
507. Kavitha TK, Samprathi M, Jayashree M, Gautam V, Sangal L. Clinical Profile of Critical Pertussis in Children at a Pediatric Intensive Care Unit in Northern India. Indian Pediatr 2020; 57(3): 228-31.
508. Kayima P, Kitya D, Punchak M, Anderson GA, Situma M. Patterns and treatment outcomes of anorectal malformations in Mbarara Regional Referral Hospital, Uganda. J Pediatr Surg 2019; 54(4): 838-44.
509. Kazembe LN, Kleinschmidt I, Sharp BL. Patterns of malaria-related hospital admissions and mortality among Malawian children: an example of spatial modelling of hospital register data. Malar J 2006; 5: 93.
510. Kelly DF, Thorson S, Maskey M, et al. The burden of vaccine-preventable invasive bacterial infections and pneumonia in children admitted to hospital in urban Nepal. Int J Infect Dis 2011; 15(1): e17-23.
511. Kerac M, Bunn J, Chagaluka G, et al. Follow-up of post-discharge growth and mortality after treatment for severe acute malnutrition (FuSAM study): a prospective cohort study. PLoS ONE 2014; 9(6): e96030.
512. Kesici S, Bayrakci B. Impact of Leadership of Trained Intensivist in Pediatric Intensive Care Unit in a Middle Income Country: An Uncontrolled Before-After Study. Indian J Pediatr 2020; 87(4): 262-7.
513. Ketharanathan N, McCulloch M, Wilson C, et al. Fluid overload in a South African pediatric intensive care unit. J Trop Pediatr 2014; 60(6): 428-33.
514. Khagayi S, Omore R, Otieno GP, et al. Effectiveness of Monovalent Rotavirus Vaccine Against Hospitalization With Acute Rotavirus Gastroenteritis in Kenyan Children. Clinical Infectious Diseases 2020; 70(11): 2298-305.
515. Khalid F, Chong LA. National pediatric palliative care needs from hospital deaths. Indian Journal of Palliative Care 2019; 25(1): 135-41.
516. Khan Sial GZ, Khan SJ. Pediatric Cancer Outcomes in an Intensive Care Unit in Pakistan. J 2019; 5: 1-5.

517. Khan MA, Kumar RV, Pandey K, Murti K, Gupta AK. Epidemiological survey of visceral leishmaniasis: An evidence based report. American Journal of Infectious Diseases 2013; 9(2): 59-62.
518. Khan MB, Riaz M, Javed G, Hashmi FA, Sanaullah M, Ahmed SI. Surgical management of traumatic extra dural hematoma in children: Experiences and analysis from 24 consecutively treated patients in a developing country. Surg Neurol Int 2013; 4: 103.
519. Khan MH, Irshad M, Ullah I, Aurakzai AA. Complications and outcome of diphtheria in admitted pediatric patients at a tertiary care setting in Peshawar. Journal of Postgraduate Medical Institute 2018; 32(3): 241-5.
520. Khan MR, Maheshwari PK, Iram S, Haque A. Readmission to paediatric intensive care unit: Frequency, causes and outcome. Journal of the College of Physicians and Surgeons Pakistan 2014; 24(3): 216-7.
521. Khan MR, Maheshwari PK, Masood K, Qamar FN, Haque AU. Epidemiology and outcome of sepsis in a tertiary care PICU of Pakistan. Indian J Pediatr 2012; 79(11): 1454-8.
522. Khan NU, Razzak JA, Alam SM, Ahmad H. Emergency department deaths despite active management: experience from a tertiary care centre in a low-income country. Emerg Med Australas 2007; 19(3): 213-7.
523. Khan N, Pérez-Núñez R, Shamim N, et al. Intentional and unintentional poisoning in Pakistan: a pilot study using the Emergency Departments surveillance project. BMC Emergency Medicine 2015; 15(1): S2-S.
524. Khan UR, Bhatti JA, Zia N, Farooq U. School-based injury outcomes in children from a low-income setting: results from the pilot injury surveillance in Rawalpindi city, Pakistan. BMC Res Notes 2013; 6: 86.
525. Khanam A, Abadeen ZUL, Mushtaq R. Frequency of bronchopneumonia in patients admitted at D.H.Q Teaching Hospital Sargodha. Pakistan Journal of Medical and Health Sciences 2018; 12(2): 679-80.
526. Khatun H, Islam SB, Naila NN, et al. Clinical profile, antibiotic susceptibility pattern of bacterial isolates and factors associated with complications in culture-proven typhoid patients admitted to an urban hospital in Bangladesh. Trop Med Int Health 2018; 23(4): 359-66.
527. Khuri-Bulos N, Williams JV, Shehabi AA, et al. Burden of respiratory syncytial virus in hospitalized infants and young children in Amman, Jordan. Scand J Infect Dis 2010; 42(5): 368-74.
528. Kiguli S, Maitland K, George EC, et al. Anaemia and blood transfusion in African children presenting to hospital with severe febrile illness. BMC Med 2015; 13: 21.
529. Kilani MM, Odeh MM, Shalabi M, Al Qassieh R, Al-Tamimi M. Clinical and laboratory characteristics of SARS-CoV2-infected paediatric patients in Jordan: serial RT-PCR testing until discharge. Paediatr Int Child Health 2020: 1-10.
530. Kingu HJ, Longo-Mbenza B, Dhaffala A, Mazwai EL. Survival function and protein malnutrition in burns patients at a rural hospital in Africa. World J Surg 2011; 35(7): 1546-52.
531. Kini S, Bhat R, Handattu K, Kousika P, Thunga C. Spectrum of influenza B viral infection in indian children: A tertiary centre experience. Journal of Nepal Paediatric Society 2018; 38(3): 170-5.
532. Kini S, Kalal BS, Chandy S, Shamsundar R, Shet A. Prevalence of respiratory syncytial virus infection among children hospitalized with acute lower respiratory tract infections in Southern India. World j 2019; 8(2): 33-42.
533. Kinikar AA, Kulkarni RK, Valvi CT, et al. Predictors of mortality in hospitalized children with pandemic H1N1 influenza 2009 in Pune, India. Indian J Pediatr 2012; 79(4): 459-66.
534. Kiran N, Shobha Rani RH, JaiPrakash V, Vanaja K. Pattern of poisoning reported at south Indian tertiary care hospital. Indian Journal of Forensic Medicine and Toxicology 2008; 2(2): 17-9.
535. Kishamawe C, Rumisha SF, Mremi IR, et al. Trends, patterns and causes of respiratory disease mortality among inpatients in Tanzania, 2006-2015. Trop Med Int Health 2019; 24(1): 91-100.
536. Kissani N, Cherkaoui Rhazouani O, Berrhoute L, Chraa M, Louhab N. An update on epidemiology, diagnosis & management of NMO-SD in the tertiary neurology department of Marrakech (Morocco). Revue Neurologique 2021; 177(1-2): 80-4.
537. Kittigul L, Pitakarnjanakul P, Sujirarat D, Siripanichgon K. The differences of clinical manifestations and laboratory findings in children and adults with dengue virus infection. J Clin Virol 2007; 39(2): 76-81.
538. Kittisakmontri K, Sukhosa O. The financial burden of malnutrition in hospitalized pediatric patients under five years of age. Clin Nutr ESPEN 2016; 15: 38-43.
539. Kochar DK, Tanwar GS, Khatri PC, et al. Clinical features of children hospitalized with malaria--a study from Bikaner, northwest India. Am J Trop Med Hyg 2010; 83(5): 981-9.
540. Kohli U, Kuttiat VS, Lodha R, Kabra SK. Profile of childhood poisoning at a tertiary care centre in North India. Indian J Pediatr 2008; 75(8): 791-4.
541. Komomo E, Chimaeze T, Emmanuel E, Asindi A. Non-accidental broom sticks injury as a cause of post-neonatal tetanus. Pan African Medical Journal 2019; 34.
542. Kool B, Raj N, Wainiqolo I, Kafoa B, McCaig E, Ameratunga S. Hospitalised and fatal head injuries in Viti Levu, Fiji: findings from an island-wide trauma registry (TRIP 4). Neuroepidemiology 2012; 38(3): 179-85.
543. Kortz TB, Axelrod DM, Chisti MJ, Kache S. Clinical outcomes and mortality before and after implementation of a pediatric sepsis protocol in a limited resource setting: A retrospective cohort study in Bangladesh. PLoS ONE 2017; 12(7): e0181160.

544. Krishnamurthy S, Narayanan P, Prabha S, et al. Clinical profile of acute kidney injury in a pediatric intensive care unit from Southern India: A prospective observational study. Indian J 2013; 17(4): 207-13.
545. Kruger I, Gie R, Kruger M, Harvey J. Outcome of children admitted to a general high-care unit in a regional hospital in the Western Cape, South Africa. SAJCH South African Journal of Child Health 2016; 10(3): 156-60.
546. Krumkamp R, Struck NS, Lorenz E, et al. Classification of invasive bloodstream infections and Plasmodium falciparum malaria using autoantibodies as biomarkers. Sci 2020; 10(1): 21168.
547. Kulkarni KP, Marwaha RK, Trehan A, Bansal D. Survival outcome in childhood ALL: experience from a tertiary care centre in North India. Pediatr Blood Cancer 2009; 53(2): 168-73.
548. Kulkarni R, Rajput U, Dawre R, et al. Severe Malnutrition and Anemia Are Associated with Severe COVID in Infants. J Trop Pediatr 2020; 14: 14.
549. Kumaira Fonseca M, Trindade EN, Costa Filho OP, Nacul MP, Seabra AP. Impact of COVID-19 Outbreak on the Emergency Presentation of Acute Appendicitis. Am Surg 2020; 86(11): 1508-12.
550. Kumar D, Singh MV, Yadav RK, Singh DK, Siddiqui SA. Vitamin D levels in paediatric intensive care unit patients and its relation to severity of illness: An Indian experience. Trop Doct 2021: 49475521992999.
551. Kumar D, Srivastava S, Tevatia MS, et al. Hepatitis B vaccination in Indian children: Seroprotection and age-related change in antibody titres. Medical Journal Armed Forces India 2020.
552. Kumar N, Gupta AK, Najotra DK, et al. Incidence and pattern of acute poisoning among children aged 1 month to 18 years: An experience from Jammu. Indian Journal of Forensic Medicine and Toxicology 2013; 7(2): 135-8.
553. Kumar R, Canarie MF. Developing Pediatric Critical Care in Kenya. Pediatr Crit Care Med 2019; 20(12): e538-e45.
554. Kumar R, Singh J, Joshi K, Singh HP, Bijesh S. Co-morbidities in hospitalized children with severe acute malnutrition. Indian Pediatrics 2014; 51(2): 125-7.
555. Kumar R, Tripathi P, Singh S, Bannerji G. Clinical features in children hospitalized during the 2005 epidemic of Japanese encephalitis in Uttar Pradesh, India. Clin Infect Dis 2006; 43(2): 123-31.
556. Kundal VK, Debnath PR, Sen A. Epidemiology of Pediatric Trauma and its Pattern in Urban India: A Tertiary Care Hospital-Based Experience. J 2017; 22(1): 33-7.
557. Kuti BP, Adegoke SA, Ebruke BE, Howie S, Oyelami OA, Ota M. Determinants of oxygen therapy in childhood pneumonia in a resource-constrained region. ISRN pediatr 2013; 2013: 435976.
558. Kwizera A, Kissoon N, Musa N, et al. A Machine Learning-Based Triage Tool for Children with Acute Infection in a Low Resource Setting. Pediatric Critical Care Medicine 2019; 20(12): e524-e30.
559. Kyeyagalire R, Tempia S, Cohen AL, et al. Hospitalizations associated with influenza and respiratory syncytial virus among patients attending a network of private hospitals in South Africa, 2007-2012. BMC Infect Dis 2014; 14: 694.
560. Labib JR, Ibrahim SK, Salem MR, Youssef MRL, Meligy B. Infection with gram-negative bacteria among children in a tertiary pediatric hospital in Egypt. Am J Infect Control 2018; 46(7): 798-801.
561. Lai J, Nguyen C, Tabwaia B, et al. Temporal decline in diarrhea episodes and mortality in Kiribati children two years following rotavirus vaccine introduction, despite high malnutrition rates: a retrospective review. BMC Infect Dis 2020; 20(1): 207.
562. Lakshmi KS, Jayashree M, Singhi S, Ray P. Study of nosocomial primary bloodstream infections in a pediatric intensive care unit. J Trop Pediatr 2007; 53(2): 87-92.
563. Lalwani S, Hasan F, Khurana S, Mathur P. Epidemiological trends of fatal pediatric trauma: A single-center study. Medicine (Baltimore) 2018; 97(39): e12280.
564. Lambisia AW, Onchaga S, Murunga N, Lewa CS, Nyanjom SG, Agoti CN. Epidemiological Trends of Five Common Diarrhea-Associated Enteric Viruses Pre- and Post-Rotavirus Vaccine Introduction in Coastal Kenya. Pathogens 2020; 9(8): 15.
565. Landi M, Swakin E, Minijihau C, Welch H, Tefuarani N, Duke T. Severe malnutrition in children in Papua New Guinea: effect of a multi-faceted intervention to improve quality of care and nutritional outcomes. Paediatr Int Child Health 2017; 37(1): 21-8.
566. Lang HJ, Amito J, Dönser MW, Giera R, Towey R. Intensive-care management of snakebite victims in rural sub-saharan Africa: An experience from Uganda. Southern African Journal of Critical Care 2020; 36(1): 39-45.
567. Lazzerini M, Seward N, Lufesi N, et al. Mortality and its risk factors in Malawian children admitted to hospital with clinical pneumonia, 2001-12: a retrospective observational study. Lancet Glob Health 2016; 4(1): e57-68.
568. Le Geyt J, Hauck S, Lee M, et al. Respiratory syncytial virus prevalence in children admitted to five Kenyan district hospitals: a cross-sectional study. BMJ paediatr 2019; 3(1): e000409.
569. Le NK, Hf W, Vu PD, et al. High prevalence of hospital-acquired infections caused by gram-negative carbapenem resistant strains in Vietnamese pediatric ICUs: A multi-centre point prevalence survey. Medicine (Baltimore) 2016; 95(27): e4099.
570. Lelei LK, Chebor AK, Mwangi HR. Burns injuries among in-patients at moi teaching and referral hospital, eldoret, Kenya. Annals of African Surgery 2011; 8: 12-5.

571. Leoncio JM, Almeida VFd, Ferrari RAP, Capobiango JD, Kerbauy G, Tacla MTGM. Impacto das infecções relacionadas à assistência à saúde nos custos da hospitalização de crianças. Rev Esc Enferm USP 2019; 53: e03486-e.
572. Leotte J, Trombetta H, Faggion HZ, et al. Impact and seasonality of human rhinovirus infection in hospitalized patients for two consecutive years. J Pediatr (Rio J) 2017; 93(3): 294-300.
573. Leung DT, Das SK, Malek MA, et al. Concurrent pneumonia in children under 5 years of age presenting to a diarrheal hospital in Dhaka, Bangladesh. American Journal of Tropical Medicine and Hygiene 2015; 93(4): 831-5.
574. Lilyasari O, Prakoso R, Kurniawati Y, et al. Clinical Profile and Management of Rheumatic Heart Disease in Children and Young Adults at a Tertiary Cardiac Center in Indonesia. Front 2020; 7: 47.
575. Lochindarat S, Bunnag T. Clinical presentations of pandemic 2009 influenza A (H1N1) virus infection in hospitalized Thai children. J Med Assoc Thai 2011; 94 Suppl 3: S107-12.
576. Lonngren C, Morrow BM, Haynes S, Yusri T, Vyas H, Argent AC. North-South divide: distribution and outcome of respiratory viral infections in paediatric intensive care units in Cape Town (South Africa) and Nottingham (United Kingdom). J Paediatr Child Health 2014; 50(3): 208-15.
577. Lopes Merino MdFG, Rosseto de Oliveira R, Ramos da Silva PLdA, de Barros Carvalho MD, Pelloso SM, Ieda Harumi H. Hospitalization and mortality by diabetes mellitus in children: analysis of temporal series. Revista Brasileira de Enfermagem 2019; 72(3): 147-53.
578. Lopes CL, Pinheiro PP, Barberena LS, Eckert GU. Diabetic ketoacidosis in a pediatric intensive care unit. J Pediatr (Rio J) 2017; 93(2): 179-84.
579. Lopes JM, Sanchis GJB, Medeiros JLAd, Dantas FG. Hospitalização por acidente vascular encefálico isquêmico no Brasil: estudo ecológico sobre possível impacto do Hiperdia. Rev bras epidemiol 2016; 19(1): 122-34.
580. Lopez AL, Raguindin PF, Esparagoza J, et al. Impact of rotavirus vaccine on diarrheal hospitalization and outpatient consultations in the Philippines: First evidence from a middle-income Asian country. Vaccine 2018; 36(23): 3308-14.
581. Loren Rebouças Santos H, Diego de Brito Sousa J, Arthur Alcântara J, et al. Rattlesnakes bites in the Brazilian Amazon: Clinical epidemiology, spatial distribution and ecological determinants. Acta Tropica 2019; 191: 69-76.
582. Louriz M, Mahraoui C, Azzouzi A, et al. Clinical features of the initial cases of 2009 pandemic influenza A (H1N1) virus infection in an university hospital of Morocco. International Archives of Medicine 2010; 3(1).
583. Lovato-Salas F, Matienzo-Serment L, Monjaras-Avila C, et al. Pandemic influenza A(H1N1) 2009 and respiratory syncytial virus associated hospitalizations. J Infect 2010; 61(5): 382-90.
584. Lovera D, Arbo A. Risk factors for mortality in Paraguayan children with pneumococcal bacterial meningitis. Trop Med Int Health 2005; 10(12): 1235-41.
585. Lovera D, De Cuellar CM, Araya S, et al. Clinical characteristics and risk factors of dengue shock syndrome in children. Pediatric Infectious Disease Journal 2016; 35(12): 1294-9.
586. Lozano-Espinosa DA, Huertas-Quinones VM, Rodriguez-Martinez CE. Impact of pulmonary hypertension and congenital heart disease with hemodynamic repercussion on the severity of acute respiratory infections in children under 5 years of age at a pediatric referral center in Colombia, South America. Cardiol Young 2020; 30(12): 1866-73.
587. Lucumay EM, Gilyoma JM, Rambau PF, Chalya PL. Paediatric neck masses at a University teaching hospital in northwestern Tanzania: a prospective analysis of 148 cases. BMC Res Notes 2014; 7: 772.
588. Luong LTM, Dang TN, Thanh Huong NT, et al. Particulate air pollution in Ho Chi Minh city and risk of hospital admission for acute lower respiratory infection (ALRI) among young children. Environ Pollut 2020; 257: 113424.
589. Lyamuya F, Michael F, Jani B, et al. Trends in diarrhea hospitalizations among infants at three hospitals in Tanzania before and after rotavirus vaccine introduction. Vaccine 2018; 36(47): 7157-64.
590. Ma C, Gunaratnam LC, Ericson A, et al. Handheld Point-of-Care Lactate Measurement at Admission Predicts Mortality in Ugandan Children Hospitalized with Pneumonia: A Prospective Cohort Study. Am J Trop Med Hyg 2019; 100(1): 37-42.
591. Mabiala-Babela JR, Pandzou N, Koutaba E, Ganga-Zandzou S, Senga P. Retrospective study of visceral surgical emergencies in children at the University Hospital Center of Brazzaville (Congo). Med Trop (Mars) 2006; 66(2): 172-6.
592. Mackenzie G, Ceesay SJ, Hill PC, et al. A decline in the incidence of invasive non-typhoidal Salmonella infection in The Gambia temporally associated with a decline in malaria infection. PLoS ONE 2010; 5(5): e10568.
593. Madondo A, Macintyre UE, Ntuli B. The clinical and anthropometric profile of undernourished children aged under 5 admitted to Nyangabgwe referral hospital in Botswana. SAJCH South African Journal of Child Health 2012; 6(4): 123-7.
594. Magalhães ALA, Morais Neto OLd. Desigualdades intraurbanas de taxas de internações por condições sensíveis à atenção primária na região central do Brasil. Ciência & Saúde coletiva 2017; 22(6): 2049-62.
595. Magalhães LS, Policena GM, Carneiro VSM, Costa LDC, Costa MSND, Vieira MADS. Analysis of the trend of hospitalizations for asthma in children and adolescents in Brazil. Jornal de Pediatria 2020.
596. Magesa AS, Magesa PM. Association between anaemia and infections (HIV, malaria and hookworm) among children admitted at Muhimbili National Hospital. East Afr J Public Health 2012; 9(3): 96-100.

597. Maghendji-Nzondo S, Nzoughe H, Lemamy GJ, et al. Prevalence of malaria, prevention measures, and main clinical features in febrile children admitted to the Franceville Regional Hospital, Gabon. Parasite 2016; 23: 32.
598. Magree HC, Russell FM, Sa'aga R, et al. Chest X-ray-confirmed pneumonia in children in Fiji. Bull World Health Organ 2005; 83(6): 427-33.
599. Mahajan V, Arora S, Kaur T, Gupta S, Guglani V. Unexpected hospitalisations at a 23-hour observation unit in a paediatric emergency department of northern India. J Clin Diagn Res 2013; 7(7): 1418-20.
600. Maine RG, Kajombo C, Purcell L, Gallaher JR, Reid TD, Charles AG. Effect of in-hospital delays on surgical mortality for emergency general surgery conditions at a tertiary hospital in Malawi. BJS Open 2019; 3(3): 367-75.
601. Maior M, Osorio-de-Castro CGS, Andrade CLT. Demographics, deaths and severity indicators in hospitalizations due to drug poisoning among children under age five in Brazil. Rev 2020; 23: e200016.
602. Majola NF, Kong VY, Mangray H, Govindasamy V, Laing GL, Clarke DL. An audit of ingested and aspirated foreign bodies in children at a university hospital in South Africa: The Pietermaritzburg experience. Samj, S 2018; 108(3): 205-9.
603. Makgatho E, Patel F, Izu A, et al. Trends in diarrhoeal disease hospitalisation in a paediatric short-stay ward at a tertiary-level hospital in Soweto: 2002-2016. SAJCH South African Journal of Child Health 2019; 13(4): 154-7.
604. Malamba S, Hladik W, Reingold A, et al. The effect of HIV on morbidity and mortality in children with severe malarial anaemia. Malar J 2007; 6: 143.
605. Malande OO, Nuttall J, Pillay V, Bamford C, Eley B. A ten-year review of ESBL and non-ESBL Escherichia coli bloodstream infections among children at a tertiary referral hospital in South Africa. PLoS ONE 2019; 14(9): e0222675.
606. Malik AH, Saima BD, Wani MY. Management of hepatobiliary and pancreatic ascariasis in children of an endemic area. Pediatr Surg Int 2006; 22(2): 164-8.
607. Malik A, Earhart K, Mohareb E, et al. Dengue hemorrhagic fever outbreak in children in Port Sudan. J Infect Public Health 2011; 4(1): 1-6.
608. Malik HS, Cheema HA, Hashmi MA, Waheed N, Anwar A, Anjum MN. Frequency of hepato-biliary disorders in children presenting at the children’s hospital & the institute of child health, lahore, a tertiary care referral center. Pakistan Paediatric Journal 2017; 41(1): 42-8.
609. Malik MA, Zia-Ur-Rehman M, Nadeem MM, et al. Childhood arterial ischemic stroke (c AIS): Evaluation, management and prognostic factors. Pakistan Paediatric Journal 2010; 34(4): 213-20.
610. Malla T, Malla KK, Thapalial A, Shaw C. Enteric fever: a retrospective 6-year analysis of 82 paediatric cases in a teaching hospital. Kathmandu Univ 2007; 5(2): 181-7.
611. Maltha J, Guiraud I, Kabore B, et al. Frequency of severe malaria and invasive bacterial infections among children admitted to a rural hospital in Burkina Faso. PLoS ONE 2014; 9(2): e89103.
612. Mammen VG, Dangor Z, Moore DP, Izu A, Beylis N, Madhi SA. Hospitalization for Culture-confirmed Pulmonary Tuberculosis in the Era of Childhood Pneumococcal Conjugate Vaccine Immunization. Pediatr Infect Dis J 2017; 36(1): e14-e21.
613. Manabe T, Higuera Iglesias AL, Vazquez Manriquez ME, et al. Socioeconomic factors influencing hospitalized patients with pneumonia due to influenza A(H1N1)pdm09 in Mexico. PLoS ONE 2012; 7(7): e40529.
614. Mancaneira JF, Benedetti JR, Zhang L. Hospitalizations and deaths due to pertussis in children from 1996 to 2013. J Pediatr (Rio J) 2016; 92(1): 40-5.
615. Maneekarn N, Khamrin P. Rotavirus associated gastroenteritis in Thailand. Virusdisease 2014; 25(2): 201-7.
616. Manuyakorn W, Benjaponpitak S, Kamchaisatian W, Vilaiyuk S, Sasisakulporn C, Jotikasthira W. Pediatric anaphylaxis: triggers, clinical features, and treatment in a tertiary-care hospital. Asian Pac J Allergy Immunol 2015; 33(4): 281-8.
617. Maphalala G, Phungwayo N, Masona G, et al. Early impact of rotavirus vaccine in under 5 year old children hospitalized due to diarrhea, Swaziland. Vaccine 2018; 36(47): 7210-4.
618. Marete I, Simba J, Gisore P, et al. Nutritional Status of Children Admitted for Diarrhoeal Diseases in a Referral Hospital in Western Kenya. East Afr Med J 2013; 90(7): 222-5.
619. Marombwa NR, Sawe HR, George U, et al. Performance characteristics of a local triage tool and internationally validated tools among under-fives presenting to an urban emergency department in Tanzania. BMC Pediatr 2019; 19(1): 44.
620. Martin SM, Balestracci A, Aprea V, et al. Acute kidney injury in critically ill children: incidence and risk factors for mortality. Arch argent pediatr 2013; 111(5): 411-6.
621. Martinez-Medina MA, Rascon-Alcantar A. Complications and cause of death in mexican children with rocky mountain spotted fever. Gac Med Mex 2016; 152(6): 789-95.
622. Martins Faria Faddul Alves MV, de Oliveira Bissiguini P, Trevizani Nitsche MJ, Leite Rosa Olbrich SR, Bronzato Luppi CH, Ramos Toso LA. PROFILE OF PATIENTS ADMITTED IN A PEDIATRIC INTENSIVE CARE UNIT OF A HOSPITAL SCHOOL IN THE COUNTRYSIDE OF SAO PAULO. Ciencia, Cuidado e Saude 2014; 13(2): 294-301.

623. Martins CBDG, De Andrade SM. Violence in childhood and youth: Hospital attendances and mortality in a city of southern Brazil. Acta Scientiarum - Health Sciences 2006; 28(1): 29-35.
624. Martins CBG, Andrade SM. Burns in children and adolescents: hospital morbidity and mortality analysis. Acta Paulista de Enfermagem 2007; 20(4): 464-9.
625. Mascarenhas MDM, Vieira LC, Lanzieri TM, Leal APPR, Duarte AF, Hatch DL. Poluição atmosférica devida à queima de biomassa florestal e atendimentos de emergência por doença respiratória em Rio Branco, Brasil - Setembro, 2005. J bras pneumol 2008; 34(1): 42-6.
626. Masood Z, Wardug GM, Ashraf J. Spinal injuries: Experience of a local neurosurgical centre. Pakistan Journal of Medical Sciences 2008; 24(3): 368-71.
627. Masukawa MdLT, Souza EMd, Gimenes E, Uchimura NS, Moriwaki AM, Uchimura TT. Time series investigation of changes in seasonality of acute diarrhea hospitalizations before and after rotavirus vaccine in Southern Brazil. Cad saúde pública 2016; 32(10): e00080515-e.
628. Matamoros M, Rodriguez R, Callejas A, et al. In-hospital pediatric cardiac arrest in Honduras. Pediatric Emergency Care 2015; 31(1): 31-5.
629. Math RS, Sharma G, Kothari SS, et al. Prospective study of infective endocarditis from a developing country. Am Heart J 2011; 162(4): 633-8.
630. Mathangi Ramakrishnan K, Mathivanan Y, Sankar J. Profile of children abused by burning. Ann 2010; 23(1): 8-12.
631. Mathew A, Sundar Rao PS, Sowmyanarayanan TV, Kang G. Severity of rotavirus gastroenteritis in an Indian population: Report from a 3 year surveillance study. Vaccine 2014; 32(S1): A45-A8.
632. Mathew R, Jamshed N, Aggarwal P, Patel S, Pandey RM. Profile of acute poisoning cases and their outcome in a teaching hospital of north India. J 2019; 8(12): 3935-9.
633. Matsuno AK, Gagliardi TB, Paula FE, et al. Human coronavirus alone or in co-infection with rhinovirus C is a risk factor for severe respiratory disease and admission to the pediatric intensive care unit: A one-year study in Southeast Brazil. PLoS ONE 2019; 14(6): e0217744.
634. Matta L, Barbosa MM, Morales-Plaza CD. Clinical profile of dengue in patients consulting a tertiary hospital in the city of Cali, Colombia, 2013. Biomedica (Bogota) 2016; 36(1): 133-9.
635. Mattioli G, Osnel L, Wong MC, Palo F, Faticato MG, Petralia P. Non-standard approach to infants and children with megacolon: laparotomy and endorectal pull-through (ERPT) for diagnosis
and treatment in difficult countries with low resources in a non-profit setting: return to the past Soave's ERPT. Minerva Pediatr 2019; 21: 21.
636. Matute SED, Donadi EA, Nunes AA, Martinez EZ. Clinical response to antibiotics in indigenous versus non-indigenous children under 5 years old with community-acquired pneumonia in Otavalo, Ecuador. Rev Soc Bras Med Trop 2020; 53: e20200038.
637. Maude RJ, Hasan MU, Hossain MA, et al. Temporal trends in severe malaria in Chittagong, Bangladesh. Malar J 2012; 11: 323.
638. Maude RR, Ghose A, Samad R, et al. A prospective study of the importance of enteric fever as a cause of non-malarial febrile illness in patients admitted to Chittagong Medical College Hospital, Bangladesh. BMC Infect Dis 2016; 16(1): 567.
639. Mayxay M, Douangdala P, Vilayhong C, et al. Outcome of Japanese encephalitis virus (JEV) infection in pediatric and adult patients at Mahosot Hospital, Vientiane, Lao PDR. American Journal of Tropical Medicine and Hygiene 2021; 104(2): 567-75.
640. Mayxay M, Douangdala P, Vilayhong C, et al. Outcome of Japanese Encephalitis Virus (JEV) Infection in Pediatric and Adult Patients at Mahosot Hospital, Vientiane, Lao PDR. Am J Trop Med Hyg 2020; 21: 21.
641. Mazhar MB, Hamid MH. Validity of Pediatric Index of Mortality 2 score as an Outcome Predictor in Pediatric ICU of aPublic Sector Tertiary Care Hospital in Pakistan. Journal of Pediatric Intensive Care 2021.
642. Mazhar M, Ahmad I, Karim A. Frequency and outcome of hypoglycemia in acute watery diarrhea complicated by dehydration in 6-59 months children. Pakistan Journal of Medical and Health Sciences 2018; 12(1): 101-3.
643. Mazumdar J, Chawla-Sarkar M, Rajendran K, et al. Burden of respiratory tract infections among paediatric in and out-patient units during 2010-11. Eur Rev Med Pharmacol Sci 2013; 17(6): 802-8.
644. Mazzillo Vega L, Cabrera Bravo N. Uso racional de antibióticos y tecnología FilmArray para identificación rápida de bacteriemias en unidad de cuidados intensivos pediátrica. Rev chil pediatr 2020; 91(4): 553-60.
645. Márquez Costa MV, Alberici Pastore C. Herramienta de cribado nutricional versus valoración nutricional antropométrica de niños hospitalizados: ¿Cuál método se asocia mejor con la evolución clínica? Arch latinoam nutr 2015; 65(1): 12-20.
646. Mbethe AP, Mda S. Incidence of refeeding syndrome and its associated factors in South African children hospitalized with severe acute malnutrition. Iranian Journal of Pediatrics 2017; 27(3).
647. Mbevi G, Ayieko P, Irimu G, Akech S, English M. Prevalence, aetiology, treatment and outcomes of shock in children admitted to Kenyan hospitals. BMC Medicine 2016; 14: 184-.

648. Mboera LEG, Rumisha SF, Lyimo EP, et al. Cause-specific mortality patterns among hospital deaths in Tanzania, 2006-2015. PLoS ONE 2018; 13(10): e0205833.
649. McCracken JP, Prill MM, Arvelo W, et al. Respiratory syncytial virus infection in Guatemala, 2007-2012. J Infect Dis 2013; 208 Suppl 3: S197-206.
650. McHaile DN, Philemon RN, Kabika S, et al. Prevalence and genotypes of Rotavirus among children under 5 years presenting with diarrhoea in Moshi, Tanzania: a hospital based cross sectional study. BMC Res Notes 2017; 10(1): 542.
651. McMorrow ML, Tempia S, Walaza S, et al. The role of human immunodeficiency virus in influenza- and respiratory syncytial virus-associated hospitalizations in south african children, 2011-2016. Clinical Infectious Diseases 2019; 68(5): 773-80.
652. Medici Olaso C, Crapuchetti Hurtado C, Mattiauda Paradizo A, et al. Investigación de metapneumovirus humano en pacientes hospitalizados: estudio multicéntrico. Arch pediatr Urug 2010; 81(4).
653. Medina Morales JB, Medina Camus B, Mayorga Giannone R. Evaluación del estado nutricional en pacientes menores de 5 años con neumoní-a grave internados en la Unidad de Cuidados Intensivos en el Instituto de Salud Nacional del Niño durante el año 2010. 2011: 20-.
654. Mehta P, Sinha A, Sami A, et al. Incidence of acute kidney injury in hospitalized children. 2012; 1(7): 537-42.
655. Mehta RL, Burdmann EA, Cerdá J, et al. Recognition and management of acute kidney injury in the International Society of Nephrology 0by25 Global Snapshot: a multinational cross-sectional study. Lancet 2016; 384 North American Edition(10032): 2017-25.
656. Mendes PSdA, Ribeiro Jr HC, Mendes CMC. Tendência temporal da mortalidade geral e morbidade hospitalar por doença diarréica em crianças brasileiras menores de cinco anos no período de 2000 a 2010. J pediatr (Rio J) 2013; 89(3): 315-25.
657. Mendonca JG, Guimaraes MJB, Mendonca VG, Portugal JL, Mendonca CG. Profile of hospitalizations in Pediatric Intensive Care Units of the Brazilian Unified Health System in the state of Pernambuco, Brazil. Cienc 2019; 24(3): 907-16.
658. Menezes AM, Noal RB, Cesar JA, et al. Hospital admissions from birth to early adolescence and early-life risk factors: the 11-year follow-up of the 1993 Pelotas (Brazil) birth cohort study. 2010; 1(10): 1980-9.
659. Mengist B, Tesfa M, Kassie B. Time to recovery and predictors of severe community-acquired pneumonia among pediatric patients in Debre Markos referral hospital, North West Ethiopia: A retrospective follow-up study. PLoS ONE 2020; 15(9): e0239655.
660. Menif K, Bouziri A, Khaldi A, et al. Community-associated methicillin-resistant Staphylococcus aureus infections in a pediatric intensive care unit. J 2011; 5(8): 587-91.
661. Mergani A, Khamis AH, Fatih Hashim EL, et al. Pattern and predictors of neurological morbidities among childhood cerebral malaria survivors in central Sudan. J Vector Borne Dis 2015; 52(3): 239-44.
662. Merida-Vieyra J, Aquino-Andrade A, Palacios-Reyes D, Murata C, Ribas-Aparicio RM, De Colsa Ranero A. Detection of Mycoplasma pneumoniae in Mexican children with community-acquired pneumonia: experience in a tertiary care hospital. Infect 2019; 12: 925-35.
663. Meyers DJ, Ozonoff A, Baruwal A, et al. Combining healthcare-based and participatory approaches to surveillance: Trends in diarrheal and respiratory conditions collected by a mobile phone system by community health workers in Rural Nepal. PLoS ONE 2016; 11(4).
664. Mghanga FP, Genge CM, Yeyeye L, et al. Magnitude, Severity, and Morphological Types of Anemia in Hospitalized Children Under the Age of Five in Southern Tanzania. Cureus 2017; 9(7): e1499.
665. Milucky JL, Compaore T, Obulbiga F, Cowman G, Whitney CG, Bicaba B. Estimating the catchment population and incidence of severe acute respiratory infections in a district hospital in Bousse, Burkina Faso. J 2020; 10(1): 010422.
666. Miranda-Choque E, Candela-Herrera J, Díaz-Pera J, Farfán-Ramos S, Muñoz-Junes EM, Escalante-Santivañez IR. Complicated chickenpox in a national pediatric Peruvian hospital, 2001-2011. Revista Peruana de Medicina Experimental y Salud Publica 2013; 30(1): 45-8.
667. Miranda-Choque E, Candela-Herrera J, Dí-az- Pera J, Farfán-Ramos S, Muñoz-Junes EMa, Escalante-Santivañez IR. Varicela complicada en un hospital pediítrico de referencia, Perú 2001-2011. Rev peru med exp salud publica 2013; 30(1): 45-8.
668. Mirkarimi M, Alisamir M, Saraf S, Heidari S, Barouti S, Mohammadi S. Clinical and Epidemiological Determinants of Lower Respiratory Tract Infections in Hospitalized Pediatric Patients. Int J Pediatr 2020; 2020: 8844420.
669. Mishra K, Basu S, Kumar D, Dutta AK, Kumar P, Rath B. Tetanus - still a scourge in the 21st century: a paediatric hospital-based study in India. Trop Doct 2012; 42(3): 157-9.
670. Mishra OP, Gupta AK, Pooniya V, Prasad R, Tiwary NK, Schaefer F. Peritoneal dialysis in children with acute kidney injury: a developing country experience. Perit Dial Int 2012; 32(4): 431-6.
671. Mishra P, Nayak L, Das RR, Dwibedi B, Singh A. Viral Agents Causing Acute Respiratory Infections in Children under Five: A Study from Eastern India. Int J Pediatr 2016; 2016: 7235482.

672. Mishra S, Mallick A, Mohanty G, Nayak P. Cerebral venous sinus thrombosis in children: A study from a tertiary care hospital of eastern India. Journal of Pediatric Neurosciences 2020; 15(4): 370-4.
673. Mishra V, Awasthi S, Nag VL, Tandon R. Genomic diversity of group A rotavirus strains in patients aged 1-36 months admitted for acute watery diarrhoea in northern India: a hospital-based study. Clin Microbiol Infect 2010; 16(1): 45-50.
674. Misra RN, Bawa KS, Magu SK, Bhandari S, Nagendra A, Menon PK. Outbreak of multi-drug resistant Salmonella typhi enteric fever in Mumbai garrison. Medical Journal Armed Forces India 2005; 61(2): 148-50.
675. Misra UK, Kalita J, Bhoi SK. Spectrum and outcome predictors of central nervous system infections in a neurological critical care unit in India: a retrospective review. Trans R Soc Trop Med Hyg 2014; 108(3): 141-6.
676. Misra UK, Kalita J, Bhoi SK, Dubey D. Cost of status epilepticus in a tertiary care hospital in India. Seizure 2015; 31: 94-8.
677. Mital AK, Choudhary P, Jain RB. Prevalence and risk factors for neurocysticercosis in children with a first-onset seizure in rural North India. Paediatr Int Child Health 2020; 40(3): 158-65.
678. Mital G, Vegad MM, Soni ST, Patel KJ, Khyati P. Clinical predictors of associated with influenza A (H1N1) during epidemic 2015 at tertiary care hospital, Ahmedabad. Indian Journal of Public Health Research and Development 2017; 8(1): 80-5.
679. Mittal M, Jain R, Talukdar B, Kumar M, Kapoor K. Emerging new trends of malaria in children: a study from a tertiary care centre in northern India. J Vector Borne Dis 2014; 51(2): 115-8.
680. Moges T, Gedlu E, Isaakidis P, et al. Infective endocarditis in Ethiopian children: a hospital based review of cases in Addis Ababa. Pan Afr Med J 2015; 20: 75.
681. Moges T, Haidar J. Management and outcome of severely malnourished children admitted to Zewditu Memorial Hospital, Ethiopia. East Afr J Public Health 2009; 6(2): 162-7.
682. Mohammed M, Mekonen Y, Berhe H. Clinical Profile of Pediatric Burn Patients in Burn Care Unit of Halibet Hospital in 2018: Asmara, Eritrea. Pediatric health med 2021; 12: 13-21.
683. Mohanty P, Kumar D, Mansingh A, Thiyagarajan V, N SR, Ray RK. Rotavirus Gastroenteritis Hospitalizations Among Under-Five Children in Bhubaneswar, Odisha, India. Indian J Pediatr 2021; 08: 08.
684. Mohy-Eldeen ZM, Ahmad AR, Mahran HH, Saad K. Comparative study of various therapeutic modalities for Guillain Barre syndrome in Assiut University Children Hospital. Turk J Pediatr 2020; 62(6): 962-9.
685. Mondal R, Sarkar S, Hazra A, et al. Clinico-anthropometric profile of children from Darjeeling tea garden. Indian J Pediatr 2014; 81(4): 350-5.
686. Moodley Y, Moodley VM, Mashele SS, Kiran RP, Madiba TE. Geospatial distribution of severe paediatric intussusception in KwaZulu-Natal province, South Africa. Pan Afr Med J 2020; 36: 320.
687. Mopeli RK, Ballot DE, White DA. An audit of primary medical conditions in children admitted to the paediatric intensive care unit of charlotte maxeke johannesburg academic hospital. SAJCH South African Journal of Child Health 2016; 10(4): 221-6.
688. Morales-Garcia M, Mata-Martinez JL, Gonzalez-Flores D, Herrera-Arellano A. Standardized mortality with PIM2 score in a pediatric intensive care unit in Morelos, Mexico. Rev 2020; 58(1): 11-20.
689. Moreira LL, Netto EM, Nascimento-Carvalho CM. Risk factors for nosocomial rotavirus infection in a paediatric hospital: the potential role for rotavirus vaccine use. Vaccine 2009; 27(3): 416-20.
690. Moreno YM, Hauschild DB, Barbosa E, Bresolin NL, Mehta NM. Problems With Optimal Energy and Protein Delivery in the Pediatric Intensive Care Unit. Nutr Clin Pract 2016; 31(5): 673-80.
691. Morgan OW, Chittaganpitch M, Clague B, et al. Hospitalization due to human parainfluenza virus-associated lower respiratory tract illness in rural Thailand. Influenza and other Respiratory Viruses 2013; 7(3): 280-5.
692. Morpeth SC, Deloria Knoll M, Scott JAG, et al. Detection of Pneumococcal DNA in Blood by Polymerase Chain Reaction for Diagnosing Pneumococcal Pneumonia in Young Children From Low- and Middle-Income Countries. Clin Infect Dis 2017; 64(suppl_3): S347-S56.
693. Morrow BM, Argent AC. Ventilator-associated pneumonia in a paediatric intensive care unit in a developing country with high HIV prevalence. J Paediatr Child Health 2009; 45(3): 104-11.
694. Morrow BM, Hatherill M, Smuts HE, Yeats J, Pitcher R, Argent AC. Clinical course of hospitalised children infected with human metapneumovirus and respiratory syncytial virus. J Paediatr Child Health 2006; 42(4): 174-8.
695. Morrow BM, Hsaio NY, Zampoli M, Whitelaw A, Zar HJ. Pneumocystis pneumonia in South African children with and without human immunodeficiency virus infection in the era of highly active antiretroviral therapy. Pediatr Infect Dis J 2010; 29(6): 535-9.
696. Moss WJ, Fisher C, Scott S, et al. HIV type 1 infection is a risk factor for mortality in hospitalized Zambian children with measles. Clin Infect Dis 2008; 46(4): 523-7.
697. Mostert S, Njuguna F, Kemps L, et al. Epidemiology of diagnosed childhood cancer in Western Kenya. Arch Dis Child 2012; 97(6): 508-12.
698. Moyo SJ, Gro N, Matee MI, et al. Age specific aetiological agents of diarrhoea in hospitalized children aged less than five years in Dar es Salaam, Tanzania. BMC Pediatr 2011; 11: 19.

699. Moyo S, Hanevik K, Blomberg B, et al. Genetic diversity of norovirus in hospitalised diarrhoeic children and asymptomatic controls in Dar es Salaam, Tanzania. Infect Genet Evol 2014; 26: 340-7.
700. Mpabalwani EM, Lukwesa-Musyani C, Imamba A, et al. Declines in Pneumonia and Meningitis Hospitalizations in Children Under 5 Years of Age After Introduction of 10-Valent Pneumococcal Conjugate Vaccine in Zambia, 2010-2016. Clin Infect Dis 2019; 69(Supplement_2): S58-S65.
701. Msanga DR, Masoza TS, Mahamba D, et al. Adenovirus Infection Is Predicted by Prolonged Duration of Diarrhea among Rotavirus-Vaccinated Children below Five Years of Age in Mwanza, Tanzania. Int J Pediatr 2020; 2020: 9303216.
702. Msangeni HA, Kamugisha ML, Sembuche SH, et al. Prospective study on severe malaria among in-patients at Bombo regional hospital, Tanga, north-eastern Tanzania. BMC Infect Dis 2011; 11: 256.
703. Msefula CL, Olgemoeller F, Jambo N, et al. Ascertaining the burden of invasive Salmonella disease in hospitalised febrile children aged under four years in Blantyre, Malawi. PLoS Negl Trop Dis 2019; 13(7): e0007539.
704. Mtove G, Amos B, Nadjm B, et al. Decreasing incidence of severe malaria and community-acquired bacteraemia among hospitalized children in Muheza, north-eastern Tanzania, 2006-2010. Malar J 2011; 10: 320.
705. Muhammad A, Khan SN, Jamal T, Haq IU, Jabbar A, An I. Bacterial Spectrum and Antimicrobial Profile of Pediatric Blood Stream Infection at a Tertiary Care Hospital in Pakistan. Chinese Medical Sciences Journal 2020; 35(4): 315-22.
706. Muhsin M, Sarhat AR, Ghani MFA. Urinary tract infection in malnourished children under 5 years in Tikrit - Iraq. Indian Journal of Public Health Research and Development 2019; 10(1): 653-7.
707. Mukai Ade O, Alves Kde S, Nascimento LF. Spatial analysis of hospitalizations for pneumonia in the Vale do Paraiba region of Brazil. 2009; 1(8): 753-8.
708. Mukendi D, Lilo Kalo JR, Mpanya A, et al. Clinical Spectrum, Etiology, and Outcome of Neurological Disorders in the Rural Hospital of Mosango, the Democratic Republic of Congo. Am J Trop Med Hyg 2017; 97(5): 1454-60.
709. Mukhida K, Sharma MR, Shilpakar SK. Pediatric neurotrauma in Kathmandu, Nepal: implications for injury management and control. Childs Nerv Syst 2006; 22(4): 352-62.
710. Mukhtar B, Siddiqui NR, Haque A. Clinical Characteristics and Immediate-Outcome of Children Mechanically Ventilated in PICU of Pakistan. Pak 2014; 30(5): 927-30.
711. Mulyani NS, Prasetyo D, Karyana IPG, et al. Diarrhea among hospitalized children under five: A call for inclusion of rotavirus vaccine to the national immunization program in Indonesia. 2018; 1(51): 7826-31.
712. Munthali T, Chabala C, Chama E, et al. Tuberculosis caseload in children with severe acute malnutrition related with high hospital based mortality in Lusaka, Zambia. BMC Res Notes 2017; 10(1): 206.
713. Munthali T, Jacobs C, Sitali L, Dambe R, Michelo C. Mortality and morbidity patterns in under-five children with severe acute malnutrition (SAM) in Zambia: a five-year retrospective review of hospital-based records (2009-2013). Arch 2015; 73(1): 23.
714. Munube D, Katabira E, Ndeezi G, et al. Prevalence of stroke in children admitted with sickle cell anaemia to Mulago Hospital. BMC Neurol 2016; 16: 175.
715. Munywoki PK, Ohuma EO, Ngama M, Bauni E, Scott JA, Nokes DJ. Severe lower respiratory tract infection in early infancy and pneumonia hospitalizations among children, Kenya. Emerg Infect Dis 2013; 19(2): 223-9.
716. Muoneke VU, Ibekwe RC, Nebe-Agumadu HU, Ibe BC. Factors associated with mortality in under-five children with severe anemia in Ebonyi, Nigeria. 2012; 1(2): 119-23.
717. Murigo-Shumba D, Bhimma R, Naicker E. Severe hypertension in children at a central referral hospital in kwazulu-natal province, south africa. SAJCH South African Journal of Child Health 2019; 13(1): 44-8.
718. Murithi I, Gichuhi S, Njuguna MW. Ocular injuries in children. East Afr Med J 2008; 85(1): 39-45.
719. Murni IK, Duke T, Daley AJ, Kinney S, Soenarto Y. Predictors of mortality in children with nosocomial bloodstream infection. Paediatr Int Child Health 2019; 39(2): 119-23.
720. Murray J, Soenarto SY, Mulyani NS, et al. Multicountry Analysis of Spectrum of Clinical Manifestations of Children <5 Years of Age Hospitalized with Diarrhea. Emerg Infect Dis 2019; 25(12): 2253-6.
721. Murray J, Yati Soenarto S, Mulyani NS, et al. Multicountry analysis of spectrum of clinical manifestations in children <5 years of age hospitalized with diarrhea. Emerging Infectious Diseases 2019; 25(12): 2253-6.
722. Musoma SN, Omar A, Mutai BC, Laigong P. Outcomes of Children and Adolescents Admitted with Diabetic Ketoacidosis at Kenyatta National Hospital (KNH), Kenya. J Diabetes Res 2020; 2020: 8987403.
723. Mustafa F, Koekemoer LA, Green RJ, Turner AC, Becker P, van Biljon G. Successful antibiotic stewardship in hospitalised children in a developing nation. J Glob Antimicrob Resist 2020; 23: 217-20.
724. Mustafa ZU, Salman M, Rao AZ, et al. Assessment of antibiotics use for children upper respiratory tract infections: a retrospective, cross-sectional study from Pakistan. Infect Dis (Lond) 2020; 52(7): 473-8.
725. Mutalima N, Molyneux E, Jaffe H, et al. Associations between Burkitt lymphoma among children in Malawi and infection with HIV, EBV and malaria: results from a case-control study. PLoS ONE 2008; 3(6): e2505.
726. Mutiso VM, Muoki AS, Kimeu MM. Patterns of Poisoning among Patients Aged 0-13 Years at a Paediatric Hospital in Nairobi. East Afr Med J 2014; 91(11): 379-84.Mutto M, Lawoko S, Nansamba C, Ovuga E, Svanstrom L.

Unintentional childhood injury patterns, odds, and outcomes in Kampala City: an analysis of surveillance data from the National Pediatric Emergency Unit. J Inj Violence Res 2011; 3(1): 13-8.
727. Mutucumarana CP, Bodinayake CK, Nagahawatte A, et al. Geospatial analysis of dengue emergence in rural areas in the Southern Province of Sri Lanka. Transactions of the Royal Society of Tropical Medicine and Hygiene 2020; 114(6): 408-14.
728. Muzigaba M, Puoane T, Sartorius B, Sanders D. Independent and interactive effects of hiv infection, clinical stage and other comorbidities on survival of children treated for severe malnutrition in rural South Africa: A retrospective multicohort study. SAJCH South African Journal of Child Health 2017; 11(1): 46-53.
729. Mwangome MK, Fegan G, Prentice AM, Berkley JA. Are diagnostic criteria for acute malnutrition affected by hydration status in hospitalized children? A repeated measures study. Nutr J 2011; 10: 92.
730. Myers S, Dinga P, Anderson M, et al. Use of bubble continuous positive airway pressure (bCPAP) in the management of critically ill children in a Malawian paediatric unit: an observational study. BMJ Open Respir Res 2019; 6(1): e000280.
731. Nabukeera-Barungi N, Grenov B, Lanyero B, et al. Predictors of mortality among hospitalized children with severe acute malnutrition: a prospective study from Uganda. Pediatr Res 2018; 84(1): 92-8.
732. Nadjm B, Mtove G, Amos B, et al. Blood glucose as a predictor of mortality in children admitted to the hospital with febrile illness in Tanzania. Am J Trop Med Hyg 2013; 89(2): 232-7.
733. Nafi O. Rotavirus gastroenteritis among children aged under 5 years in Al Karak, Jordan. East Mediterr Health J 2010; 16(10): 1064-9.
734. Nagaraj S, Kalal BS, Manoharan A, Shet A. Streptococcus pneumoniae serotype prevalence and antibiotic resistance among young children with invasive pneumococcal disease: experience from a tertiary care center in South India. Germs 2017; 7(2): 78-85.
735. Nahar B, Ahmed T, Brown KH, Hossain MI. Risk factors associated with severe underweight among young children reporting to a diarrhoea treatment facility in Bangladesh. 2010; 1(5): 476-83.
736. Naidoo N, Muckart DJ. The wrong and wounding road: Paediatric polytrauma admitted to a level 1 trauma intensive care unit over a 5-year period. Samj, S 2015; 105(10): 823-6.
737. Naik AC, Bhat S, Kholkute SD. Hospitalisation due to infectious and parasitic diseases in District Civil Hospital, Belgaum, Karnataka. Indian J Public Health 2008; 52(3): 161-3.
738. Naisaki A, Wainiqolo I, Kafoa B, et al. Fatal and hospitalised childhood injuries in Fiji (TRIP Project-3). Journal of Paediatrics and Child Health 2013; 49(1): 63-7.
739. Nakarmi KK, Pathak BD. Prevalence of acute pediatric burns in a tertiary care hospital. Journal of the Nepal Medical Association 2020; 58(231): 862-5.
740. Nallasamy K, Angurana SK, Jayashree M, et al. Clinical Profile, Hospital Course and Outcome of Children with COVID-19. Indian Journal of Pediatrics 2021.
741. Nallasamy K, Gupta S, Bansal A, et al. Clinical Profile and Predictors of Intensive Care Unit Admission in Pediatric Scrub Typhus: A Retrospective Observational Study from North India. Indian J 2020; 24(6): 445-50.
742. Nalwanga D, Musiime V, Kizito S, et al. Mortality among children under five years admitted for routine care of severe acute malnutrition: a prospective cohort study from Kampala, Uganda. BMC Pediatr 2020; 20(1): 182.
743. Nandan D, Agarwal S, Bidhuri N, Shrivastava K, Nanda P, Lata S. Role of Intrapleural Urokinase in Empyema Thoracis. Indian J Pediatr 2019; 86(12): 1099-104.
744. Nandwani S, Bhakhri BK, Singh N, Rai R, Singh DK. Early hematological parameters as predictors for outcomes in children with dengue in northern India: A retrospective analysis. Rev Soc Bras Med Trop 2021; 54: e05192020.
745. Nansera D, Max I, Annet K, Gessner BD. Bacterial meningitis among children under the age of 2 years in a high human immunodeficiency virus prevalence area after Haemophilus influenzae type b vaccine introduction. J Paediatr Child Health 2012; 48(4): 324-8.
746. Nansseu JR, Alima Yanda AN, Chelo D, et al. The Acute Chest Syndrome in Cameroonian children living with sickle cell disease. BMC Pediatr 2015; 15: 131.
747. Nantanda R, Ostergaard MS, Ndeezi G, Tumwine JK. Clinical outcomes of children with acute asthma and pneumonia in Mulago hospital, Uganda: a prospective study. BMC Pediatr 2014; 14: 285.
748. Nantongo JM, Wobudeya E, Mupere E, et al. High incidence of pulmonary tuberculosis in children admitted with severe pneumonia in Uganda. BMC Pediatr 2013; 13: 16.
749. Naorat S, Chittaganpitch M, Thamthitiwat S, et al. Hospitalizations for acute lower respiratory tract infection due to respiratory syncytial virus in Thailand, 2008-2011. J Infect Dis 2013; 208 Suppl 3: S238-45.

750. Narang M, Khurana A, Gomber S, Choudhary N. Epidemiological trends of tetanus from East Delhi, India: a hospital-based study. J Infect Public Health 2014; 7(2): 121-4.
751. Nariadhara MR, Sawe HR, Runyon MS, Mwafongo V, Murray BL. Modified systemic inflammatory response syndrome and provider gestalt predicting adverse outcomes in children under 5 years presenting to an urban emergency department of a tertiary hospital in Tanzania. Trop 2019; 47: 13.
752. Nascimento LF, Medeiros AP. Admissions due to pneumonia and biomass burning: a spatial approach. J Pediatr (Rio J) 2012; 88(2): 177-83.
753. Nascimento MS, Souza AV, Ferreira AV, Rodrigues JC, Abramovici S, Silva Filho LV. High rate of viral identification and coinfections in infants with acute bronchiolitis. 2010; 1(11): 1133-7.
754. Nascimento-Carvalho AC, Ruuskanen O, Nascimento-Carvalho CM. Comparison of the frequency of bacterial and viral infections among children with community-acquired pneumonia hospitalized across distinct severity categories: a prospective cross-sectional study. BMC Pediatr 2016; 16: 105.
755. Nascimento-Carvalho CM, Araujo-Neto CA, Ruuskanen O. Association between bacterial infection and radiologically confirmed pneumonia among children. Pediatr Infect Dis J 2015; 34(5): 490-3.
756. Nascimento-Carvalho CM, Cardoso MA, Barral A, et al. Procalcitonin is useful in identifying bacteraemia among children with pneumonia. Scandinavian Journal of Infectious Diseases 2010; 42(9): 644-9.
757. Nascimento-Carvalho CM, Cardoso MR, Ruuskanen O, Lappalainen M. Sole infection by human metapneumovirus among children with radiographically diagnosed community-acquired pneumonia in a tropical region. Influenza other respi 2011; 5(4): 285-7.
758. Nascimento-Carvalho EC, Vasconcellos AG, Clarencio J, et al. Evolution of cytokines/chemokines in cases with community-acquired pneumonia and distinct etiologies. Pediatr Pulmonol 2020; 55(1): 169-76.
759. Nasio NA. Profiles and outcomes of head injury at nakuru level 5 hospital. Annals of African Surgery 2017; 14(2).
760. Nasir AA, Bello JO, Ofoegbu CKP, Abdur-Rahman LO, Yakub S, Solagberu BA. Childhood motorcycle-related injuries in a Nigerian city - prevalence, spectrum and strategies for control. SAJCH South African Journal of Child Health 2011; 5(2): 48-50.
761. Nasreen S, Luby SP, Brooks WA, et al. Population-based incidence of severe acute respiratory virus infections among children aged <5 years in rural Bangladesh, June-October 2010. PLoS ONE 2014; 9(2): e89978.
762. Natali RMT, dos Santos DSPS, da Fonseca AMC, et al. Hospital admissions due to respiratory diseases in children and adolescents of São Paulo city, 2000-2004. Revista Paulista de Pediatria 2011; 29(4): 584-90.
763. Natali RMdT, Santos DSPSd, Fonseca AMCd, et al. Perfil de internações hospitalares por doenças respiratórias em crianças e adolescentes da cidade de São Paulo, 2000-2004. Rev paul pediatr 2011; 29(4): 584-90.
764. Nataprawira HM, Ruslianti V, Solek P, et al. Outcome of tuberculous meningitis in children: the first comprehensive retrospective cohort study in Indonesia. Int J Tuberc Lung Dis 2016; 20(7): 909-14.
765. Naveen KN, Pai VV, Rai V, Athanikar SB. Retrospective analysis of Steven Johnson syndrome and toxic epidermal necrolysis over a period of 5 years from northern Karnataka, India. Indian J Pharmacol 2013; 45(1): 80-2.
766. Naz A, Rehman EU, Bai S, Hamid R, Ghaffar MS, Kumari G. Various causes of bleeding per rectum in children presenting at a tertiary care hospital. Rawal Medical Journal 2019; 44(4): 717-20.
767. Ndadane N, Maharaj RC. The epidemiology of sepsis in a district hospital emergency centre in Durban, KwaZulu natal. African Journal of Emergency Medicine 2019; 9(3): 123-6.
768. Ndayishimiye E, Ross AJ. An audit of the screen-and-treat intervention to reduce cryptococcal meningitis in HIV-positive patients with low CD4 count. Afr 2018; 10(1): e1-e7.
769. Ndong IC, van Reenen M, Boakye DA, Mbacham WF, Grobler AF. Trends in malaria admissions at the Mbakong Health Centre of the North West Region of Cameroon: a retrospective study. Malar J 2014; 13: 328.
770. Nduhukire T, Atwine D, Rachel L, Byonanebye JE. Predictors of in-hospital mortality among under-five children with severe acute malnutrition in South-Western Uganda. PLoS ONE 2020; 15(6): e0234343.
771. Neira RAQ, Hamacher S, Japiassu AM. Epidemiology of sepsis in Brazil: Incidence, lethality, costs, and other indicators for Brazilian Unified Health System hospitalizations from 2006 to 2015. PLoS ONE 2018; 13(4).
772. Ngabo F, Gatera M, Karema C, et al. Can routinely collected national data on childhood morbidity and mortality from diarrhea be used to monitor health impact of rotavirus vaccination in Africa?: Examination of pre-vaccine baseline data from Rwanda. Pediatric Infectious Disease Journal 2014; 33(SUPPL. 1): S89-S93.
773. Ngabo F, Mvundura M, Gazley L, et al. The Economic Burden Attributable to a Child's Inpatient Admission for Diarrheal Disease in Rwanda. PLoS ONE 2016; 11(2): e0149805.
774. Ngabo F, Tate JE, Gatera M, et al. Effect of pentavalent rotavirus vaccine introduction on hospital admissions for diarrhoea and rotavirus in children in Rwanda: a time-series analysis. Lancet Glob Health 2016; 4(2): e129-36.
775. Nguefack F, Mah E, Kinkela MN, et al. Mortality pattern in children aged 3-59 months hospitalized in the Intensive Care Unit at a Paediatric Center in Yaounde-Cameroon. Pan Afr Med J 2020; 36: 246.
776. Nguyen NT, Pham HV, Hoang CQ, et al. Epidemiological and clinical characteristics of children who died from hand, foot and mouth disease in Vietnam, 2011. BMC Infect Dis 2014; 14: 341.

777. Nguyen PTK, Tran HT, Fitzgerald DA, Tran TS, Graham SM, Marais BJ. Characterisation of children hospitalised with pneumonia in central Vietnam: a prospective study. Eur Respir J 2019; 54(1).
778. Nguyen PTK, Tran HT, Tran TS, Fitzgerald DA, Graham SM, Marais BJ. Predictors of 'unlikely bacterial pneumonia' and 'adverse pneumonia outcome' in children admitted to hospital in central Vietnam. Clin Infect Dis 2019; 27: 27.
779. Nguyen PTK, Tran HT, Tran TS, Fitzgerald DA, Graham SM, Marais BJ. Predictors of Unlikely Bacterial Pneumonia and Adverse Pneumonia Outcome in Children Admitted to a Hospital in Central Vietnam. Clin Infect Dis 2020; 70(8): 1733-41.
780. Nguyen PT, Tran HT, Fitzgerald DA, Graham SM, Marais BJ. Antibiotic use in children hospitalised with pneumonia in Central Vietnam. Arch Dis Child 2020; 105(8): 713-9.
781. Nguyen P, Binns CW, Ha AVV, et al. Prelacteal and early formula feeding increase risk of infant hospitalisation: a prospective cohort study. Arch Dis Child 2020; 105(2): 122-6.
782. Nguyen TKP, Bui BBS, Ngo QC, Fitzgerald DA, Graham SM, Marais BJ. Applying lessons learnt from research of child pneumonia management in Vietnam. Paediatr Respir Rev 2020; 08: 08.
783. Nguyen TL, Nguyen TH, Morita S, Sakamoto J. Injury and pre-hospital trauma care in Hanoi, Vietnam. Injury 2008; 39(9): 1026-33.
784. Nicol MP, Schumacher SG, Workman L, et al. Accuracy of a novel urine test, Fujifilm SILVAMP TB LAM, for the diagnosis of pulmonary tuberculosis in children. Clin Infect Dis 2020; 06: 06.
785. Nielsen KR, Becerra MR, Mallma G, et al. Nasal high flow therapy introduction lowers reintubation risk in a Peruvian paediatric intensive care unit. Acta Paediatr 2020; 109(12): 2748-54.
786. Nielsen MV, Sarpong N, Krumkamp R, et al. Incidence and characteristics of bacteremia among children in rural Ghana. PLoS ONE 2012; 7(9): e44063.
787. Njuguna HN, Zaki SR, Roberts DJ, et al. Determining the Cause of Death Among Children Hospitalized With Respiratory Illness in Kenya: Protocol for Pediatric Respiratory Etiology Surveillance Study (PRESS). JMIR Res Protoc 2019; 8(1): e10854.
788. Njunda AL, Fon SG, Assob JC, Nsagha DS, Kwenti TD, Kwenti TE. Coinfection with malaria and intestinal parasites, and its association with anaemia in children in Cameroon. Infect 2015; 4: 43.
789. Nkosi-Gondwe T, Calis J, Boele van Hensbroek M, Bates I, Blomberg B, Phiri KS. A cohort analysis of survival and outcomes in severely anaemic children with moderate to severe acute malnutrition in Malawi. PLoS ONE 2021; 16(2): e0246267.
790. Novaes HM, Sartori AM, Soarez PC. Hospitalization rates for pneumococcal disease in Brazil, 2004 - 2006. 2011; 1(3): 539-47.
791. Noyola DE, Alpuche-Solis AG, Herrera-Diaz A, Soria-Guerra RE, Sanchez-Alvarado J, Lopez-Revilla R. Human metapneumovirus infections in Mexico: epidemiological and clinical characteristics. J Med Microbiol 2005; 54(Pt 10): 969-74.
792. Nukiwa N, Burmaa A, Kamigaki T, et al. Evaluating influenza disease burden during the 2008-2009 and 2009-2010 influenza seasons in Mongolia. Western pac 2011; 2(1): 16-22.
793. Nunthanid S, Tiawilai A. DENGUE PATIENTS AT PHOTHARAM HOSPITAL: A CLINICAL TRIAL SITE OF DENGUE VACCINE. The Southeast Asian journal of tropical medicine and public health 2015; 46: 11-6.
794. Nupen TL, Argent AC, Morrow BM. Characteristics and outcome of long-stay patients in a paediatric intensive care unit in Cape Town, South Africa. South African Medical Journal 2017; 107(1): 70-5.
795. Nurleila S, Syafruddin D, Elyazar IRF, Baird EJK. Serious and fatal illness associated with falciparum and vivax malaria among patients admitted to hospital at West Sumba in Eastern Indonesia. American Journal of Tropical Medicine and Hygiene 2012; 87(1): 41-9.
796. Nuzhat S, Ahmed T, Kawser CA, et al. Age specific fast breathing in under-five diarrheal children in an urban hospital: Acidosis or pneumonia? PLoS ONE 2017; 12(9): e0185414.
797. Nyambat B, Dang DA, Nguyen HA, et al. Rapid assessment of Hib disease burden in Vietnam. BMC Public Health 2011; 11: 260.
798. Nyamwange CI, Mkoji G, Mpoke S, Nyandieka HS. Cryptosporidiosis and Its Genotypes among Children Attending Moi Teaching and Referral Hospital in Eldoret, Kenya. East Afr Med J 2012; 89(1): 11-9.
799. Nyirasafari R, Corden MH, Karambizi AC, et al. Predictors of mortality in a paediatric intensive care unit in Kigali, Rwanda. Paediatr Int Child Health 2017; 37(2): 109-15.
800. Nzioki C, Irimu G, Musoke R, English M. Audit of care for children aged 6 to 59 months admitted with severe malnutrition at kenyatta national hospital, kenya. Int Health 2009; 1(1): 91-6.
801. O'Brien KL, Baggett HC, Brooks WA, et al. Causes of severe pneumonia requiring hospital admission in children without HIV infection from Africa and Asia: the PERCH multi-country case-control study. The Lancet 2019; 394(10200): 757-79.
802. O'Callaghan-Gordo C, Bassat Q, Morais L, et al. Etiology and epidemiology of viral pneumonia among hospitalized children in rural Mozambique: a malaria endemic area with high prevalence of human immunodeficiency virus. Pediatr Infect Dis J 2011; 30(1): 39-44.

803. Obichukwu CC, Odetunde OI, Chinawa JM, Okafor HU, Adiele DK, Ibe BC. Community-Acquired acute kidney injury in critically Ill children as seen in the emergency unit of a tertiary hospital in Enugu, Southeast Nigeria. Niger J Clin Pract 2017; 20(6): 746-53.
804. Obidike E, Oguonu T, Eze J, et al. Misdiagnosis of pneumonia, bronchiolitis and reactive airway disease in children: A retrospective case review series in South East, Nigeria. Current Pediatric Research 2018; 22(2): 166-71.
805. Odhiambo WA, Guthua SW, Chindia ML, Macigo FG. Pattern and clinical characteristics of firearm injuries. East Afr Med J 2008; 85(3): 107-12.
806. Odondi RN, Shitsinzi R, Emarah A. Clinical patterns and early outcomes of burn injuries in patients admitted at the Moi Teaching and Referral Hospital in Eldoret, Western Kenya. Heliyon 2020; 6(3): e03629.
807. Ofoegbu CK, Odi T, Ogundipe O, Taiwo J, Solagberu BA. Epidemiology of non-trauma surgical deaths. West Afr J Med 2005; 24(4): 321-4.
808. Oke OJ, Ajite A, Oluwayemi OI, Olatunya OS, Ogundare EO, Abdulraheem FO. Malaria Infection as a Leading Cause of Febrile Seizures among Children with Seizures in Ekiti State University Teaching Hospital, Ado Ekiti, Nigeria. Journal of Pediatric Neurology 2021; 19(1): 7-13.
809. Okemwa MP, Rogena EA, Rana F, Gatei DG. Pattern of road traffic fatalities in Nairobi. Annals of African Surgery 2008; 3: 15-20.
810. Oketcho R, Karimuribo ED, Nyaruhucha CNM, Taybali S. Epidemiological factors in admissions for diarrhoea in 6 - 60-month-old children admitted to Morogoro Regional Hospital, Tanzania. SAJCH South African Journal of Child Health 2012; 6(3): 81-4.
811. Okiro EA, Alegana VA, Noor AM, Mutheu JJ, Juma E, Snow RW. Malaria paediatric hospitalization between 1999 and 2008 across Kenya. BMC Med 2009; 7: 75.
812. Okiro EA, Alegana VA, Noor AM, Snow RW. Changing malaria intervention coverage, transmission and hospitalization in Kenya. Malar J 2010; 9: 285.
813. Okugbo SU, Anyanhun GA, Efobi CA, Okugbo OT. Presentation and management outcome of childhood corrosive oesophageal injury in Benin City. Afr J Paediatr Surg 2020; 17(3 & 4): 74-8.
814. Oliveira TC, Latorre Mdo R. Trends in hospital admission and infant mortality from diarrhea: Brazil, 1995-2005. 2010; 1(1): 102-11.
815. Oliveira TCRd, Latorre MdRDO. Tendências da interação e da mortalidade infantil por diarréia: Brasil, 1995 a 2005. Rev saúde pública 2010; 44(1): 102-11.
816. Olle-Goig JE. Tuberculosis in rural Uganda. Afr Health Sci 2010; 10(3): 226-9.
817. Oloifana-Polosovai H, Gwala J, Harrington H, et al. A marked decline in the incidence of malaria in a remote region of Malaita, Solomon Islands, 2008 to 2013. Western pac 2014; 5(3): 30-9.
818. Olsen SJ, Campbell AP, Supawat K, et al. Infectious causes of encephalitis and meningoencephalitis in Thailand, 2003-2005. Emerging Infectious Diseases 2015: 280-9.
819. Olson D, Lamb MM, Gaensbauer JT, Todd JK, Halsey NA, Asturias EJ. Risk factors for death and major morbidity in guatemalan children with acute bacterial meningitis. Pediatric Infectious Disease Journal 2015; 34(7): 724-8.
820. Olugbuyi O, Swaby K, Roberts P, Christie C, Kissoon N. Burden of paediatric sepsis in a tertiary centre from a developing country. West Indian Medical Journal 2018; 67(2): 137-42.
821. Oluwayemi IO, Oyedeji OA, Adeniji EO, et al. A Ten-Year Review of the Pattern and Outcome of Childhood Diabetes in Two State Teaching Hospitals in South-West Nigeria. Diabetes Metab Syndr Obes 2020; 13: 4051-7.
822. Onakpoya OH, Adegbehingbe BO, Adeoye AO, Adewara BA. Ophthalmic admissions in a tertiary hospital in Nigeria. Afr Health Sci 2010; 10(1): 54-7.
823. Onanuga A, Lamikanra A. Association of ABO blood group and Plasmodium falciparum malaria among children in the Federal Capital Territory, Nigeria. African Journal of Biomedical Research 2016; 19(1).
824. Ongeti K, Saidi H, Ogeng'O J, Tharao M. Experience with hirschsprung's disease at a tertiary hospital in Kenya. Annals of African Surgery 2009; 4: 8-12.
825. Oniyangi O, Awani B, Iregbu KC. The pattern of paediatric HIV/AIDS as seen at the National Hospital Abuja Nigeria. Niger J Clin Pract 2006; 9(2): 153-8.
826. Onyango CO, Njeru R, Kazungu S, et al. Influenza surveillance among children with pneumonia admitted to a district hospital in coastal Kenya, 2007-2010. J Infect Dis 2012; 206 Suppl 1: S61-7.
827. Onyemaechi NO, Urube SU, Ekenze SO. Pattern of surgical emergencies in a Nigerian tertiary hospital. Afr Health Sci 2019; 19(1): 1768-77.
828. Opoka RO, Xia Z, Bangirana P, John CC. Inpatient mortality in children with clinically diagnosed malaria as compared with microscopically confirmed malaria. Pediatr Infect Dis J 2008; 27(4): 319-24.
829. Opondo C, Allen E, Todd J, English M. Association of the Paediatric Admission Quality of Care score with mortality in Kenyan hospitals: a validation study. Lancet Glob Health 2018; 6(2): e203-e10.
830. Ortiz-Prado E, Mascialino G, Paz C, et al. A Nationwide Study of Incidence and Mortality Due to Traumatic Brain Injury in Ecuador (2004-2016). Neuroepidemiology 2020; 54(1): 33-44.

831. Otieno GP, Bottomley C, Khagayi S, et al. Impact of the introduction of rotavirus vaccine on hospital admissions for diarrhoea among children in Kenya: A controlled interrupted time series analysis. Clin Infect Dis 2019; 23: 23.
832. Ouma BJ, Ssenkusu JM, Shabani E, et al. Endothelial Activation, Acute Kidney Injury, and Cognitive Impairment in Pediatric Severe Malaria. Crit Care Med 2020; 48(9): e734-e43.
833. Owor BE, Masankwa GN, Mwango LC, Njeru RW, Agoti CN, Nokes DJ. Human metapneumovirus epidemiological and evolutionary patterns in Coastal Kenya, 2007-11. BMC Infect Dis 2016; 16: 301.
834. Oyenusi EE, Oduwole AO, Aronson AS, Jonsson BG, Albertsson-Wikland K, Njokanma OF. Hyperglycemia in Acutely Ill Non-diabetic Children in the Emergency Rooms of 2 Tertiary Hospitals in Lagos, Nigeria. Pediatr Emerg Care 2016; 32(9): 608-13.
835. Oyenusi EE, Oduwole AO, Oladipo OO, Njokanma OF, Esezobor CI. Hypoglycaemia in children aged 1 month to 10 years admitted to the children's emergency centre of Lagos University Teaching Hospital, Nigeria. SAJCH South African Journal of Child Health 2014; 8(3): 107-11.
836. Page N, Groome MJ, Murray T, et al. Sapovirus prevalence in children less than five years of age hospitalised for diarrhoeal disease in South Africa, 2009-2013. J Clin Virol 2016; 78: 82-8.
837. Palacio-Mejia LS, Rojas-Botero M, Molina-Velez D, et al. Overview of acute diarrheal disease at the dawn of the 21st century: The case of Mexico. Salud Publica Mex 2020; 62(1): 14-24.
838. Palihawadana P, Kang G, Navaratnasingam J, et al. Rotavirus infection among hospitalized children under five years of age with acute watery diarrhea in Sri Lanka. 2018; 1(51): 7846-50.
839. Palma SM, Palma RT, Catapani WR, Waisberg J. Predictive factors of hospitalization in children with acute asthma at a university emergency care unit. Pediatr Emerg Care 2013; 29(11): 1175-9.
840. Parakh A, Kumar A, Kumar V, Dutta AK, Khare S. Pediatric hospitalizations associated with 2009 pandemic influenza A (H1N1): an experience from a tertiary care center in north India. Indian J Pediatr 2010; 77(9): 981-5.
841. Paratz ED, Mock N, Gutman SJ, et al. Taking the pulse of Timor-Leste's cardiac needs: a 10-year descriptive time-trend analysis. Internal Medicine Journal 2020; 50(7): 838-45.
842. Parra ME, Gonzílez AFE, Ochoa WC, Vélez AQ. Morbidity and mortality at a pediatric intensive care unit in Medellí-n, Colombia, 2001-2005. Iatreia 2008; 21(1): 33-40.
843. Patel KK, Patel AK, Mehta PM, et al. Clinical outcome of novel H1N1 (Swine Flu)-infected patients during 2009 pandemic at tertiary referral hospital in western India. Journal of Global Infectious Diseases 2013; 5(3): 93-7.
844. Patel ND, Desai KJ, Sarvaiya J, Malek S. Clinico-laboratory profile of dengue patients at sir t. Hospital, Bhavnagar, India. Acta Medica Iranica 2020; 58(2): 69-72.
845. Patel N, Khofi-Phiri I, Mathiva LR, Grieve A, Loveland J, Nethathe GD. Trauma related admissions to the PICU at Chris Hani Baragwanath Academic Hospital, Johannesburg. Pediatr Surg Int 2017; 33(9): 1013-8.
846. Patel SM, Spees L, Smieja M, et al. Predictors of Poor Outcomes Among Infants With Respiratory Syncytial Virus-associated Acute Lower Respiratory Infection in Botswana. Pediatr Infect Dis J 2019; 38(5): 525-7.
847. Paudyal BP. Poisoning: pattern and profile of admitted cases in a hospital in central Nepal. Jnm 2005; 44(159): 92-6.
848. Paulin-Prado P, Nishimura K, Freimanis-Hance L, et al. Clinical characteristics of asthmatic patients with influenza-like illness and risk of severe exacerbations in Mexico. Annals of Allergy, Asthma & Immunology 2016; 116(5): 402-7.
849. Peña-Juírez RA, Medina-Andrade MA. Current situation of congenital heart diseases in two public hospitals in the state of Jalisco. Arch cardiol Méx 2020; 90(2): 124-9.
850. Peiffer S, Ssentongo AE, Keeney L, et al. Predictors of Poor Postoperative Outcomes in Pediatric Surgery Patients in Rural Ghana. BMC surg 2020; 20(1): 211.
851. Peiris-John R, Kafoa B, Wainiqolo I, Krishnan Reddy R, McCaig E, Ameratunga SN. Population-based characteristics of fatal and hospital admissions for poisoning in Fiji: TRIP Project-11. Injury Prevention (1353-8047) 2013; 19(5): 355-7.
852. Peters SM, Davies MA, Van As AB. Repeat injuries in childhood. S Afr Med J 2020; 110(12): 1218-25.
853. Phasuk N, Nurak A. Etiology, Treatment, and Outcome of Children Aged 3 to 36 Months With Fever Without a Source at a Community Hospital in Southern Thailand. J 2020; 11: 2150132720915404.
854. Phung NTN, Dinh QN, Trinh TH, Tran DT. Organ dysfunction in severe dengue among children in a Vietnamese hospital. Systematic Reviews in Pharmacy 2020; 11(2): 22-6.
855. Phung NTN, Vo TTT, Hon KLE. The Role of Lung Ultrasonography in Etiologic Diagnosis of Acute Dyspnea in a Resource Limited Setting. Bull 2020; 8(2): 121-4.
856. Piña Borrego CE, Fernídez Fernídez MdL, Piña Rodrí-guez LK. Caracterización clínico epidemiológica de la leishmaniosis visceral infantil. Rev cuba pediatr 2020; 92(3): e771-e.
857. Piñeros JG, Baquero H, Bastidas J, et al. Infecção por vírus sincicial respiratório como causa de internação na população com menos de 1 ano na Colômbia. J pediatr (Rio J) 2013; 89(6): 544-8.

858. Pina JC, Alves LS, Arroyo LH, et al. Using geo-spatial analysis for assessing the risk of hospital admissions due to community-acquired pneumonia in under-5 children and its association with socially vulnerable areas (Brazil). BMC Pediatr 2020; 20(1): 502.
859. Piyaphanee N, Chaiyaumporn S, Phumeetham S, Lomjansook K, Sumboonnanonda A. Acute kidney injury without previous renal disease in critical care unit. Pediatr Int 2020; 62(7): 810-5.
860. Plucinski MM, Guilavogui T, Sidikiba S, et al. Effect of the Ebola-virus-disease epidemic on malaria case management in Guinea, 2014: a cross-sectional survey of health facilities. Lancet Infect Dis 2015; 15(9): 1017-23.
861. Polita NB, Ferrari RAP, Moraes PS, Sant, Anna FL, Tacla MTGM. Anomalias congênitas: internações em unidade pediátrica. Rev paul pediatr 2013; 31(2): 205-10.
862. Ponte EV, Rasella D, Souza-Machado C, Stelmach R, Barreto ML, Cruz AA. Reduced asthma morbidity in endemic areas for helminth infections: a longitudinal ecological study in Brazil. J Asthma 2014; 51(10): 1022-7.
863. Porto JP, Mantese OC, Arantes A, Freitas C, Filho PPG, Ribas RM. Nosocomial infections in a pediatric intensive care unit of a developing country: NHSN surveillance. Revista da Sociedade Brasileira de Medicina Tropical 2012; 45(4): 475-9.
864. Pradhan R, Shrestha U, Gautam SC, et al. Bloodstream infection among children presenting to a general hospital outpatient clinic in urban Nepal. PLoS ONE 2012; 7(10): e47531.
865. Pradhan SK, Panigrahi S, Padhi PS, et al. Clinical Profile, Risk-Factors, and Outcome of Rotaviral Diarrhea and Non-rotaviral Diarrhea Among Under-Five Children at Cuttack, Odisha, India. Indian Journal of Pediatrics 2021.
866. Prado S MA, Perret P C, Montecinos P L, et al. Metapneumovirus humano como causa de hospitalización en niños bajo 3 años de edad, con infección respiratoria aguda, durante el año 2004. Rev chil infectol 2007; 24(1): 19-26.
867. Prakash Raju KNJ, Jagdish S, Krishna Kumar G, Anandhi D, Antony J. Profile of pediatric trauma among the patients attending emergency department in a tertiary care hospital in South India. Journal of Emergencies, Trauma and Shock 2020; 13(1): 62-7.
868. Prasad AN. Disease profile of children in Kabul: the unmet need for health care. J Epidemiol Community Health 2006; 60(1): 20-3.
869. Prasadi GAM, Mohamed F, Senarathna L, Cairns R, Pushpakumara P, Dawson AH. Paediatric poisoning in rural Sri Lanka: an epidemiological study. BMC Public Health 2018; 18(1): 1349.
870. Prasertsan P, Anuntaseree W, Ruangnapa K, Saelim K, Geater A. Severity and Mortality Predictors of Pediatric Acute Respiratory Distress Syndrome According to the Pediatric Acute Lung Injury Consensus Conference Definition. Pediatr Crit Care Med 2019; 20(10): e464-e72.
871. Prasetyo RV, Saraswati PD, Kurniawan MR, et al. The use of PELOD score in predicting acute kidney injury in critically ill children. Journal of Nepal Paediatric Society 2016; 36(2): 165-9.
872. Prashanth GP, Pai VV. A retrospective cohort study of dermatological problems observed in paediatric intensive care unit. J Eur Acad Dermatol Venereol 2012; 26(9): 1105-8.
873. Prata-Barbosa A, Lima-Setta F, Santos GRD, et al. Pediatric patients with COVID-19 admitted to intensive care units in Brazil: a prospective multicenter study. Jornal de Pediatria 2020; 96(5): 582-92.
874. Preacely N, Biya O, Gidado S, et al. Hospital-based mortality in Federal Capital Territory hospitals--Nigeria, 2005-2008. Pan Afr Med J 2012; 11: 66.
875. Pulsan F, Sobi K, Duke T. Continuous positive airway pressure in children with severe pneumonia and hypoxaemia in Papua New Guinea: an evaluation of implementation. Acta Paediatrica, International Journal of Paediatrics 2019; 108(10): 1887-95.
876. Punpanich W, Chirapanyanon P, Srisarang S. Clinical characteristics and hospital charges among Thai children hospitalized with influenza. Southeast Asian J Trop Med Public Health 2014; 45(1): 75-84.
877. Purcell LN, Banda W, Williams B, Gallaher J, Charles A. The Effect of Surgical Intervention on Pediatric Burn Injury Survival in a Resource-Poor Setting. J Surg Res 2020; 253: 86-91.
878. Purcell LN, Prin M, Sincavage J, Kadyaudzu C, Phillips MR, Charles A. Outcomes Following Intensive Care Unit Admission in a Pediatric Cohort in Malawi. J Trop Pediatr 2020; 66(6): 621-9.
879. Purcell LN, Reiss R, Eaton J, Kumwenda KK, Quinsey C, Charles A. Survival and Functional Outcomes at Discharge After Traumatic Brain Injury in Children versus Adults in Resource-Poor Setting. World Neurosurg 2020; 137: e597-e602.
880. Purcell LN, Sincavage J, Banda W, et al. The effect of burn mechanism on pediatric mortality in Malawi: A propensity weighted analysis. Burns 2021; 47(1): 222-7.
881. Purcell LN, Yohann A, Banda W, Gallaher J, Charles A. Sex dimorphism in pediatric burn mortality in Malawi: A propensity matched analysis. Burns 2021; 47(1): 228-33.
882. Purcell L, Mabedi CE, Gallaher J, et al. Variations in injury characteristics among paediatric patients following trauma: A retrospective descriptive analysis comparing pre-hospital and in-hospital deaths at Kamuzu Central Hospital, Lilongwe, Malawi. Malawi Med J 2017; 29(2): 146-50.
883. Qadri I, Malik I, Ahmed K. Staphylococcus aureus Infection in a Pediatric ICU: A Hospital Based Prospective Observational Study. Indian J 2019; 23(5): 210-2.

884. Quang Do TVTT, Nguyen NT. Factors related to severe hand, foot, and mouth disease among Vietnamese children patients. Systematic Reviews in Pharmacy 2020; 11(1): 7-13.
885. Quinonez-Lopez D, Patino-Hernandez D, Zuluaga CA, Garcia AA, Munoz-Velandia OM. Comparison of Performance of the Pediatric Index of Mortality (PIM)-2 and PIM-3 Scores in the Pediatric Intensive Care Unit of a High Complexity Institution. Indian J 2020; 24(11): 1095-102.
886. Quintanar-Solares M, Yen C, Richardson V, Esparza-Aguilar M, Parashar UD, Patel MM. Impact of rotavirus vaccination on diarrhea-related hospitalizations among children < 5 years of age in Mexico. Pediatr Infect Dis J 2011; 30(1 Suppl): S11-5.
887. Qureshi AU, Ali AS, Ahmad TM. Comparison of three prognostic scores (PRISM, PELOD and PIM 2) at pediatric intensive care unit under Pakistani circumstances. J Ayub Med Coll Abbottabad 2007; 19(2): 49-53.
888. Qureshi S, Mir F, Junejo S, et al. The spectrum of primary immunodeficiencies at a tertiary care hospital in Pakistan. World Allergy Organiza 2020; 13(7): 100133.
889. Rabie H, de Boer A, van den Bos S, Cotton MF, Kling S, Goussard P. Children with human immunodeficiency virus infection admitted to a paediatric intensive care unit in South Africa. J Trop Pediatr 2007; 53(4): 270-3.
890. Rafai M, Mekaoui N, Chouaib N, et al. Epidemiology of severe domestic accidents of children admitted in pediatric intensive care unit of Children Hospital of Rabat-Morocco. Pan Afr Med J 2015; 20: 28.
891. Rafique I, Akhtar U, Farooq U, Khan M, Bhatti JA. Emergency care outcomes of acute chemical poisoning cases in Rawalpindi. Journal of Acute Disease 2016; 5(1): 37-40.
892. Rahmani Z, Dhoubhadel BG, Rauf FA, et al. Risk for Death among Children with Pneumonia, Afghanistan. Emerging Infectious Diseases 2017; 23(8): 1404-8.
893. Rajani HS, Narayanappa D, Sunilkumar S. Spectrum of congenital heart diseases in a tertiary care centre, Mysuru, Karnataka. Indian Journal of Public Health Research and Development 2020; 11(7): 689-93.
894. Rajasekaran J, Geminiganesan S, Jayapalan DK, Padmanaban R, Saminathan V. Serum zinc levels in children 1 - 59 months of age with pneumonia: A single-center surveillance in India from 2014 to 2016. Archives of Pediatric Infectious Diseases 2020; 8(2).
895. Rajatonirina S, Razanajatovo NH, Ratsima EH, et al. Outcome risk factors during respiratory infections in a paediatric ward in Antananarivo, Madagascar 2010-2012. PLoS ONE 2013; 8(9): e72839.
896. Rajesh NT, Alagarasu K, Patil JA, et al. Serotype-specific differences in the laboratory parameters among hospitalized children with dengue and genetic diversity of dengue viruses circulating in Tamil Nadu, India during 2017. J Med Virol 2020; 92(8): 1013-22.
897. Rajper S, Moazzam M, Zeeshan A, Abbas Q. Acute symptomatic seizures in critically ill children: frequency, etiology and outcomes. Journal of Pediatric Neurosciences 2020; 15(4): 375-8.
898. Ramanathan P, Rathnasamy R. Infections in a rheumatology setting - Chennai experience. Indian Journal of Rheumatology 2011; 6(1 SUPPL.): 31-5.
899. Ramirez Prada D, Delgado G, Hidalgo Patino CA, Perez-Navero J, Gil Campos M. Using of WHO guidelines for the management of severe malnutrition to cases of marasmus and kwashiorkor in a Colombia children's hospital. Nutr Hosp 2011; 26(5): 977-83.
900. Ramos GF, Ribeiro VP, Mercadante MP, et al. Mortality in adolescents and young adults with chronic diseases during 16 years: a study in a Latin American tertiary hospital. J Pediatr (Rio J) 2019; 95(6): 667-73.
901. Ramos JM, Reyes F, Tesfamariam A, Gil-Fournier N. Outcome of severely malnourished children treated according to UNICEF 2004 guidelines in a rural hospital in Ethiopia. Giornale Italiano di Medicina Tropicale 2009; 14(1-4): 13-6.
902. Ranque S, Poudiougou B, Traore A, et al. Life-threatening malaria in African children: a prospective study in a mesoendemic urban setting. Pediatr Infect Dis J 2008; 27(2): 130-5.
903. Rashid MM, Chisti MJ, Akter D, Sarkar M, Chowdhury F. Antibiotic use for pneumonia among children under-five at a pediatric hospital in Dhaka city, Bangladesh. Patient Prefer Adherence 2017; 11: 1335-42.
904. Rattanamongkolgul S, Plitponkarnpim A. Incidence and patterns of registered injuries among children in Ongkharak District, Nakhon Nayok, Thailand. J Med Assoc Thai 2012; 95 Suppl 12: S118-24.
905. Rattanavong S, Dubot-Peres A, Mayxay M, et al. Spatial epidemiology of Japanese encephalitis virus and other infections of the central nervous system infections in Lao PDR (2003-2011): A retrospective analysis. PLoS Negl Trop Dis 2020; 14(5): e0008333.
906. Ray S, Ansari Z, Kumar D, Jana K, Khamrui S. Short- and long-term outcome of surgery for chronic pancreatitis in children: a single surgeon experience. Pediatr Surg Int 2020; 36(9): 1087-92.
907. Raza M, Kumar S, Ejaz M, Azim D, Azizullah S, Hussain A. Electrolyte Imbalance in Children With Severe Acute Malnutrition at a Tertiary Care Hospital in Pakistan: A Cross-Sectional Study. Cureus 2020; 12(9): e10541.
908. Reddy A, Ramkumar J, Kavin K, Shanmugasundaram R, Gopinathan G, Thangavel P. Surgical correction of isolated total anomalous pulmonary venous connection: A prospective longitudinal study. Journal of Clinical and Diagnostic Research 2019; 13(11): PC01-PC4.

909. Rees CA, Kisenge R, Manji KP, Liu E, Fawzi WW, Duggan CP. Identifying Infants and Young Children at Risk of Unplanned Hospital Admissions and Clinic Visits in Dar es Salaam, Tanzania. Pediatr Infect Dis J 2020; 39(12): e428-e34.
910. Rene labib Youssef M, Mosleh H, Rene Labib J. Assessment of the performance of the Pediatric Index of Mortality 2 (PIM2) among Egyptian pediatric patients admitted to the intensive care. Egyptian Pediatric Association Gazette 2014; 62(3-4): 65-71.
911. Restrepo JM, Mondragon MV, Forero-Delgadillo JM, et al. Acute renal failure in children. Multicenter prospective cohort study in medium-complexity intensive care units from the Colombian southeast. PLoS ONE 2020; 15(8): e0235976.
912. Richards D, Hunter L, Forey K, et al. Demographics and predictors of mortality in children undergoing resuscitation at Khayelitsha Hospital, Western Cape, South Africa. SAJCH South African Journal of Child Health 2018; 12(3): 127-31.
913. Richardson-Lopez Collada V, Bautista-Marquez A, Sanchez-Uribe E, Esparza-Aguilar M. Population impact of rotavirus vaccination in Mexico after 10 years. Salud Publica Mex 2020; 62(1): 6-13.
914. Rocha GA, Rocha EJM, Martins CV. The effects of hospitalization on the nutritional status of children. J pediatr (Rio J) 2006; 82(1): 70-4.
915. Rocha NA, Silva GB, Oliveira MJ, et al. Visceral leishmaniasis in children: a cohort of 120 patients in a metropolitan city of Brazil. Turk J Pediatr 2011; 53(2): 154-60.
916. Rodrí-guez VA, Campaña A, Bustos CD, et al. Infección por adenovirus en el Hospital de niños Roberto del Rí-o 2016. Rev pediatr electrón 2017; 14(1): 55-8.
917. Rodríguez-Martínez CE, Sossa-Briceño MP, Castro-Rodriguez JA. Predictors of hospitalization for asthma in children: Results of a 1-year prospective study. Pediatric Pulmonology 2013.
918. Rodrigues A, de Carvalho M, Monteiro S, et al. Hospital surveillance of rotavirus infection and nosocomial transmission of rotavirus disease among children in Guinea-Bissau. 2007; 1(3): 233-7.
919. Rodrigues CN, Rosa CdOB, Ribeiro SMR, Santos CAd, Firmino HH. Perfil nutricional e fatores associados ao baixopeso e à redução do consumo alimentar emcrianças hospitalizadas. Mundo saúde (Impr) 2016; 40(1): [61-72].
920. Rodrigues LS, Motta FA, Picharski GL, et al. Invasive candidiasis: Risk factor for mortality in a pediatric tertiary care hospital in south of Brazil. Medicine (United States) 2019; 98(23).
921. Rodrigues S,Conceição T, Silva IS, de Lencastre H, Aires-de-Sousa M. Frequent MRSA nasal colonization among hospitalized children and their parents in Angola and São Tomé and Príncipe. Journal of Hospital Infection 2018; 100(3): 344-9.
922. Rodriguez DA, Rodriguez-Martinez CE, Cardenas AC, et al. Predictors of severity and mortality in children hospitalized with respiratory syncytial virus infection in a tropical region. Pediatr Pulmonol 2014; 49(3): 269-76.
923. Rodriguez-Auad JP, Nava-Frias M, Casasola-Flores J, et al. The epidemiology and clinical characteristics of respiratory syncytial virus infection in children at a public pediatric referral hospital in Mexico. Int J Infect Dis 2012; 16(7): e508-13.
924. Rodriguez-Martinez CE, Sossa-Briceno MP, Castro-Rodriguez JA. Direct medical costs of RSV-related bronchiolitis hospitalizations in a middle-income tropical country. Allergol Immunopathol (Madr) 2019; 21: 21.
925. Rodriguez-Martinez CE, Sossa-Briceno MP, Castro-Rodriguez JA. Direct medical costs of pediatric asthma exacerbations requiring hospital attendance in a middle-income country. Allergol Immunopathol (Madr) 2020; 48(2): 142-8.
926. Roine I, Weisstaub G, Peltola H. Influence of malnutrition on the course of childhood bacterial meningitis. Pediatric Infectious Disease Journal 2010; 29(2): 122-5.
927. Rojas JP, Leal AL, Patino J, et al. Characterization of patients who died of invasive pneumococcal disease in the child population of Bogota, Colombia. Rev Chil Pediatr 2016; 87(1): 48-52.
928. Rolsma SL, Rankin DA, Haddadin Z, et al. Assessing the epidemiology and seasonality of influenza among children under two hospitalized in Amman, Jordan, 2010-2013. Influenza other respi 2021; 15(2): 284-92.
929. Romero C, Herníndez H, Falcón N. Costos de hospitalización de los accidentes por mordedura de canes en un hospital de niños en Lima-Perú, periodo 2006-2010. Salud tecnol vet 2013; 1(2): 26-32.
930. Romero-Espinoza JA, Moreno-Valencia Y, Coronel-Tellez RH, et al. Virome and bacteriome characterization of children with pneumonia and asthma in Mexico City during winter seasons 2014 and 2015. PLoS ONE 2018; 13(2): e0192878.
931. Rosa AM, Ignotti E, Hacon SDS, De Castro HA. Analysis of hospitalizations for respiratory diseases in Tangarí da Serra, Brazil. Jornal Brasileiro de Pneumologia 2008; 34(8): 575-82.
932. Rose AM. Late presentation to hospital services necessitates greater community-based care for malnourished children. J Trop Pediatr 2015; 61(1): 61-4.
933. Roshania R, Mallow M, Dunbar N, et al. Successful Implementation of a Multicountry Clinical Surveillance and Data Collection System for Ebola Virus Disease in West Africa: Findings and Lessons Learned. Glob 2016; 4(3): 394-409.

934. Rosyidi RM, Priyanto B, Al Fauzi A, Sutiono AB. Toward zero mortality in acute epidural hematoma: A review in 268 cases problems and challenges in the developing country. Interdisciplinary Neurosurgery: Advanced Techniques and Case Management 2019; 17: 12-8.
935. Rouhani SA, Eliacin HC, Edmond MC, Checkett KA, Rimpel L, Marsh RH. Epidemiology of traumatic injuries presenting to an ED in Central Haiti: a retrospective cohort study. Emerg Med J 2019; 36(7): 389-94.
936. Rowlinson E, Dueger E, Mansour A, et al. Incidence and etiology of hospitalized acute respiratory infections in the Egyptian Delta. Influenza other respi 2017; 11(1): 23-32.
937. Rowlinson E, Dueger E, Taylor T, et al. Incidence and clinical features of respiratory syncytial virus infections in a population-based surveillance site in the nile delta region. Journal of Infectious Diseases 2013; 208: S189-96.
938. Roy SK, Buis M, Weersma R, et al. Risk factors of mortality in severely-malnourished children hospitalized with diarrhoea. 2011; 1(3): 229-35.
939. Rurangwa J, Rujeni N. Decline in Child Hospitalization and Mortality After the Introduction of the 7-Valent Pneumococcal Conjugative Vaccine in Rwanda. Am J Trop Med Hyg 2016; 95(3): 680-2.
940. Russell FM, Reyburn R, Chan J, et al. Impact of the change in WHO's severe pneumonia case definition on hospitalized pneumonia epidemiology: case studies from six countries. Bulletin of the World Health Organization 2019; 97(6): 386-93.
941. Rustagi RS, Arora K, Das RR, Pooni PA, Singh D. Incidence, risk factors and outcome of acute kidney injury in critically ill children - a developing country perspective. Paediatr Int Child Health 2017; 37(1): 35-41.
942. Saad K, Mohamed SA, Metwalley KA. Recurrent/Persistent Pneumonia among Children in Upper Egypt. Mediterr 2013; 5(1): e2013028.
943. Saavedra PA, deBrito ES, Areda CA, Escalda PM, Galato D. Burns in the Brazilian Unified Health System: a review of hospitalization from 2008 to 2017. Int 2019; 9(5): 88-98.
944. Sadarangani M, Seaton C, Scott JA, et al. Incidence and outcome of convulsive status epilepticus in Kenyan children: a cohort study. Lancet neurol 2008; 7(2): 145-50.
945. Sadoh AE, Ofili A. Hepatitis B infection among Nigerian children admitted to a children's emergency room. Afr Health Sci 2014; 14(2): 377-83.
946. Saeed AA, Usman S, Fadoo Z, Abbas Q. Outcome of sepsis in pediatric oncology patients admitted in pediatric intensive care unit: A developing country perspective. Pediatric Hematology Oncology Journal 2019; 4(2): 35-8.
947. Saha S, Ishtiaque Sayeed KM, Islam MS, et al. Hospitalization of Pediatric Enteric Fever Cases, Dhaka, Bangladesh, 2017-2019: Incidence and Risk Factors. Clinical Infectious Diseases 2020; 71: S196-S204.
948. Saha S, Ramesh A, Kalantar K, et al. Unbiased metagenomic sequencing for pediatric meningitis in bangladesh reveals neuroinvasive chikungunya virus outbreak and other unrealized pathogens. mBio 2019; 10(6).
949. Saha TK, Bose K, Sorkar UK, Chatterjee MK. Demographic profile and outcome analysis of a paediatric intensive care unit in a paediatric hospital in Kolkata. J Indian Med Assoc 2013; 111(3): 160-2.
950. Sahiledengle B, Seyoum F, Abebe D, et al. Incidence and risk factors for hospital-acquired infection among paediatric patients in a teaching hospital: a prospective study in southeast Ethiopia. BMJ Open 2020; 10(12): e037997.
951. Sahoo B, Jain MK, Thakur B, Mishra R, Patnaik S. Demographic profile and outcome of mechanically ventilated children in a tertiary care hospital of a developing country. Journal of Nepal Paediatric Society 2018; 38(1): 14-8.
952. Sahoo B, Patnaik S, Mishra R, Jain MK. Morbidity pattern of children admitted to a paediatric intensive care unit of india. International Journal of Tropical Medicine 2020; 15(4): 80-3.
953. Sakajiki AM, Ilah GB, Lukman AAS, Yakasai AM. Snake bite envenomation seen at a specialist hospital in Zamfara state, North-Western Nigeria. Annals of Tropical Medicine and Public Health 2017; 10(2): 391-5.
954. Saleh NY, Abo El Fotoh WMM, El-Hawy MA. Serum Neutrophil Gelatinase-Associated Lipocalin: A Diagnostic Marker in Pediatric Sepsis. Pediatric Critical Care Medicine 2017; 18(6): e245-e52.
955. Salih O, Nail A, Modawe G, et al. Risk factors of inpatients mortality of visceral leishmaniasis, Khartoum State, Sudan. Journal of Global Infectious Diseases 2020; 12(3): 135-40.
956. Salim H, Karyana IPG, Sanjaya-Putra IGN, Budiarsa S, Soenarto Y. Risk factors of rotavirus diarrhea in hospitalized children in Sanglah Hospital, Denpasar: a prospective cohort study. BMC Gastroenterology 2014; 14(1): 54-.
957. Samanta T, Ganguly S. Aetiology, clinical profile and prognostic indicators for children with acute liver failure admitted in a teaching hospital in Kolkata. Trop Gastroenterol 2007; 28(3): 135-9.
958. Samdan A, Ganbold S, Guntev O, et al. Hospital-based surveillance for rotavirus diarrhea in Ulaanbaatar, Mongolia, April 2009 through March 2016. Vaccine 2018; 36(51): 7883-7.
959. Samra JA, Hagood NL, Summer A, Medina MT, Holden KR. Clinical Features and Neurologic Complications of Children Hospitalized With Chikungunya Virus in Honduras. J Child Neurol 2017; 32(8): 712-6.
960. Samransamruajkit R, Hiranrat T, Chieochansin T, et al. Prevalence, clinical presentations and complications among hospitalized children with influenza pneumonia. Jpn J Infect Dis 2008; 61(6): 446-9.
961. Samuels RJ, Moon TD, Starnes JR, et al. Lassa fever among children in eastern province, Sierra Leone: A 7-year retrospective analysis (2012-2018). American Journal of Tropical Medicine and Hygiene 2021; 104(2): 585-92.

962. Sanchez-Uribe E, Esparza-Aguilar M, Parashar UD, Richardson V. Sustained Reduction of Childhood Diarrhea-Related Mortality and Hospitalizations in Mexico After Rotavirus Vaccine Universalization. Clin Infect Dis 2016; 62 Suppl 2: S133-9.
963. Sand A, Kumar R, Shaikh BT, Somrongthong R, Hafeez A, Rai D. Determinants of severe acute malnutrition among children under five years in a rural remote setting: A hospital based study from district Tharparkar-Sindh, Pakistan. Pak 2018; 34(2): 260-5.
964. Santana DP, Santos VM, da Silva AMC, Shimoya-Bittencourt W. Influence of air pollutants on pneumonia hospitalizations among children in a town in the Brazilian legal amazon region: A time series study. Sao Paulo Medical Journal 2020; 138(2): 126-32.
965. Santos JV, Souza J, Amarante J, Freitas A. Burden of Burns in Brazil from 2000 to 2014: A Nationwide Hospital-Based Study. World J Surg 2017; 41(8): 2006-12.
966. Sara H, Bouchra O, Angéla FK, Samira EF, Nehemie N, Samir A. Acute rheumatic fever in children: Experience at the hospital Hassan II of Fez, Morocco. Clinical Epidemiology and Global Health 2020; 8(4): 1062-6.
967. Sasa M, Segura Cano SE. New insights into snakebite epidemiology in Costa Rica: A retrospective evaluation of medical records. Toxicon: X 2020; 7.
968. Satapathy A, Biswal B, Priyadarshini L, et al. Clinicopathological Profile and Outcome of Childhood Urticaria Vasculitis: An Observational Study. Cureus 2020; 12(11): e11489.
969. Sawe HR, Mfinanga JA, Lidenge SJ, et al. Disease patterns and clinical outcomes of patients admitted in intensive care units of tertiary referral hospitals of Tanzania. BMC Int Health Hum Rights 2014; 14: 26.
970. Sayed SZ, Mahmoud MM, Moness HM, Mousa SO. Admission platelet count and indices as predictors of outcome in children with severe Sepsis: a prospective hospital-based study. BMC Pediatr 2020; 20(1): 387.
971. Schmidt WP, Suzuki M, Thiem VD, et al. User fee exemption does not affect lower rates of hospital admission of girls in Vietnam. Health policy and planning 2012; 27(7): 582-9.
972. Schwarz NG, Sarpong N, Hunger F, et al. Systemic bacteraemia in children presenting with clinical pneumonia and the impact of non-typhoid salmonella (NTS). BMC Infect Dis 2010; 10: 319.
973. Scott JA, Berkley JA, Mwangi I, et al. Relation between falciparum malaria and bacteraemia in Kenyan children: a population-based, case-control study and a longitudinal study. Lancet 2011; 378 North American Edition(9799): 1316-23.
974. Scotta MC, Veras TN, Klein PC, et al. Impact of 10-valent pneumococcal non-typeable Haemophilus influenzae protein D conjugate vaccine (PHiD-CV) on childhood pneumonia hospitalizations in Brazil two years after introduction. Vaccine 2014; 32(35): 4495-9.1744.
975. Secka F, Herberg JA, Sarr I, et al. Bacteremia in Childhood Life-Threatening Infections in Urban Gambia: EUCLIDS in West Africa. Open forum infect 2019; 6(9): ofz332.
976. Sehabiague Rigau G, Leonardi Capelo DD, Ibañez S, Etchevarren Ricca VnL, Hortal Palma MHe, Bello Pedrosa O. Infecciones respiratorias agudas graves de niño y su impacto en la demanda asistencial. Arch pediatr Urug 2012; 83(1): 7-12.
977. Serem GK, Newton CR, Kariuki SM. Incidence, causes and phenotypes of acute seizures in Kenyan children post the malaria-decline period. BMC Neurol 2015; 15: 180.
978. Serra JsA, Gonzílez-Dambrauskas Sn, Vísquez Hoyos P, et al. Variabilidad terapéutica en lactantes con bronquiolitis hospitalizados en unidades de cuidados intensivos latinoamericanas. Rev chil pediatr 2020; 91(2): 216-25.
979. Sevilla-Acosta F, Gutiérrez-Mata A, Yock-Corrales A, Bogantes-Ledezma S, Pérez-Corrales C, Camacho-Badilla K. Epidemiology, Etiology and Clinical Aspects of Childhood Acute Encephalitis in a Tertiary Pediatric Hospital in Costa Rica. The Pediatric infectious disease journal 2021; 40(3): 186-90.
980. Seyi-Olajide JO, Ezidiegwu U, Ameh EA. Burden of Complicated Intra-Abdominal Infections in Children in Nigeria: Recent Experience and Systematic Review. Surg Infect (Larchmt) 2020; 21(6): 501-8.
981. Sfaihi L, Elloumi S, Fourati H, Kamoun T, Mnif Z, Hachicha M. Arterial ischemic stroke in children: 22 cases from southern Tunisia. Fetal Pediat Pathol 2013; 32(4): 271-5. Zhonghua Er Ke Za Zhi 2011; 49(7): 539-44.
982. Shah AA, Shakoor A, Zogg CK, et al. Influence of sub-specialty surgical care on outcomes for pediatric emergency general surgery patients in a low-middle income country. Int J Surg 2016; 29: 12-8.
983. Shah S, Kaul A, Jadhav Y, Shiwarkar G. Clinical outcome of severe sepsis and septic shock in critically ill children. Trop Doct 2020; 50(3): 186-90.
984. Shaikh AS, Memon IA, Khatwani NR, Memon SI, Memon AI, Saleem AF. Clinical spectrum and outcome of children presented with different malarial species in Karachi, Pakistan. Pakistan Paediatric Journal 2014; 38(2): 84-90.
985. Shakoor I, Ghazal R, Fareeduddin M, Noor M. Mortality pattern in children in general pediatric ward of Abbasi Shaheed hospital Karachi. Medical Forum Monthly 2014; 25(5): 55-8.
986. Shankar G, Kalburgi EB, Hunshikatti S. Analysis of burn injuries admitted in a tertiary care teaching hospital in Bagalkot, Karnataka, India. Medico-Legal Update 2012; 12(2): 62-5.
987. Shankar G, Kalburgi EB, Srinivas G, Hunshikatti S. Epidemiology and outcome of hospitalized burn patients in a tertiary care teaching hospital in south India. Indian Journal of Public Health Research and Development 2015; 6(2): 137-9.

988. Sharif M, Saxena A, Singh S, Manchala S, Jafri N. Blood Component Transfusion in a Tertiary Care Hospital. Indian J Pediatr 2020; 87(5): 339-44.
989. Sharma AK, Chapagain RH, Bista KP, et al. Epidemiological and clinical profile of covid-19 in nepali children: An initial experience. Journal of Nepal Paediatric Society 2020; 40(3): 202-9.
990. Sharma A, Mathur A, Lundborg CS, Pathak A. Incidence and risk factors for severe dehydration in hospitalized children in ujjain, india. International Journal of Environmental Research and Public Health 2020; 17(2).
991. Sharma J, Chaudhary S, Bajaj M, Nair NP, Thiyagarajan V. Rotavirus Gastroenteritis Hospitalizations Among Under-5 Children in Northern India. Indian Journal of Pediatrics 2021.
992. Sharma R, Gohain S, Chandra J, et al. Plasmodium vivax malaria admissions and risk of mortality in a tertiary-care children's hospital in North India. Paediatr Int Child Health 2012; 32(3): 152-7.
993. Sharma V, Verma PK, Gupta S, Sharma A. Mortality from Influenza A/H1N1 in a tertiary care teaching institution in North India. J 2010; 4(8): 468-71.
994. Sharma Y, Kaur M, Singh S, Pant L, Kudesia M, Jain S. Seroprevalence and trend of dengue cases admitted to a government hospital, delhi - 5-year study (2006-2010): a look into the age shift. Int J Prev Med 2012; 3(8): 537-43.
995. Sharmin M, Chowdhury AM, Ali MA, et al. Clinical Profile and Immediate Outcome of Children Admitted With Acute Glomerulonephritis in Pediatrics Department of A Tertiary Level Hospital. Mymensingh Med J 2020; 29(1): 5-15.
996. Sherchand JB, Cunliffe NA, Tandukar S, et al. Rotavirus disease burden and molecular epidemiology in children with acute diarrhoea age less than 5 years in Nepal. Journal of Nepal Paediatric Society 2011; 31(3): 209-15.
997. Sheriff A, Rahim A, Lailabi MP, Gopi J. Unintentional injuries among children admitted in a tertiary care hospital in North Kerala. Indian J Public Health 2011; 55(2): 125-7.
998. Shetty RS, Kamath VG, Nayak DM, Hegde A, Saluja T. Rotavirus associated acute gastroenteritis among under-five children admitted in two secondary care hospitals in southern Karnataka, India. Clinical Epidemiology and Global Health 2017; 5(1): 28-32.
999. Shrestha AK, Bhattarai S, Paudel P, Basel PL. Morbidity and mortality pattern of patients admitted into paediatric intensive care unit of tertiary level hospital of Nepal. Journal of Nepal Paediatric Society 2020; 40(1): 28-33.
1000. Shrestha R, Koju P, Silwal S, Karmacharya BM, Shakya Shrestha S. Analysis of antimicrobial drugs usage pattern and it’s cost analysis in the pediatric department of a tertiary care hospital. Kathmandu University Medical Journal 2020; 18(71): 289-95.
1001. Shrestha SR. Burn injuries in pediatric population. Jnm 2006; 45(163): 300-5.
1002. Shrestha SR, Yadhav B, Shrestha S. Wheeze among pneumonia patients in paediatrics department of Patan hospital. Journal of Nepal Paediatric Society 2011; 31(2): 116-20.
1003. Shrestha S, Chaudhary N, Pathak S, Sharma A, Shrestha L, Kurm OP. Clinical predictors of radiological pneumonia: A cross-sectional study from a tertiary hospital in Nepal. PLoS ONE 2020; 15(7 July).
1004. Shukla V, Nimbalkar S, Ganjiwale J, et al. Direct Cost of Critical Illness Associated Healthcare Expenditures among Children Admitted in Pediatric Intensive Care Unit in Rural India. Indian Journal of Pediatrics 2016; 83(10): 1065-70.
1005. Shuxratovich MS, Erkinovich SK, O’g’li ASF, Qizi NMI. Patterns of paediatric maxillofacial fractures: A twelve-year retrospective study. Medico-Legal Update 2021; 21(1): 624-6.
1006. Sibomana H, Rugambwa C, Mwenda JM, et al. Impact of routine rotavirus vaccination on all-cause and rotavirus hospitalizations during the first four years following vaccine introduction in Rwanda. Vaccine 2018; 36(47): 7135-41.
1007. Siddiqui NU, Ashraf Z, Jurair H, Haque A. Mortality patterns among critically ill children in a Pediatric Intensive Care Unit of a developing country. Indian J 2015; 19(3): 147-50.
1008. Siddiqui TS, Anis ur R, Jan MA, Wazeer MS, Burki MK. Status epilepticus: aetiology and outcome in children. J Ayub Med Coll Abbottabad 2008; 20(3): 51-3.
1009. Silva PR, Rosa AM, Hacon SS, Ignotti E. Hospitalization of children for asthma in the Brazilian Amazon: trend and spatial distribution. J Pediatr (Rio J) 2009; 85(6): 541-6.
1010. Silva PSLd, Fonseca MCM. Which children account for repeated admissions within 1 year in a Brazilian pediatric intensive care unit? J pediatr (Rio J) 2019; 95(5): 559-66.
1011. Singh B, Unnikrishnan B. A profile of acute poisoning at Mangalore (South India). J 2006; 13(3): 112-6.
1012. Singh M, Sankar J, Kumar A, Kumar UV, Lodha R, Kabra SK. Predictors of Mortality in Children Admitted to the Pediatric Intensive Care Unit with Acute Gastroenteritis with Severe Dehydration. Indian J Pediatr 2019; 86(12): 1142-5.
1013. Singh SK, Poddar U, Mishra R, Srivastava A, Yachha SK. Ascitic fluid infection in children with liver disease: time to change empirical antibiotic policy. Hepatol Int 2020; 14(1): 138-44.
1014. Sinha S, Nunez Martinez CM, Hartley RL, et al. Epidemiological analysis of pediatric burns in the Dominican Republic reveals a demographic profile at significant risk for electrical burns. Burns 2019; 45(2): 471-8.

1015. Siqueira MS, Rosa RDS, Bordin R, Nugem RC. Hospitalizations due to diseases associated with poor sanitation in the public health care network of the metropolitan region of Porto Alegre, Rio Grande do Sul State, Brazil, 2010-2014. Epidemiol 2017; 26(4): 795-806.
1016. Siqueira MS, Rosa RdS, Bordin R, Nugem RdC. Internações por doenças relacionadas ao saneamento ambiental inadequado na rede pública de saúde da região metropolitana de Porto Alegre, Rio Grande do Sul, 2010-2014. Epidemiol serv saúde 2017; 26(4): 795-806.
1017. Soltani MS, Salah AB, Bouanene I, et al. Epidemiology and medical cost of hospitalization due to rotavirus gastroenteritis among children under 5 years of age in the central-east of Tunisia. East Mediterr Health J 2015; 21(8): 584-90.
1018. Sood A, Suthar R, Sahu JK, et al. Etiologic Profile of Childhood Stroke from North India: Is It Different from Developed World? J Child Neurol 2021: 883073821991291.
1019. Soto-Martinez M, Avila L, Soto N, Chaves A, Celedon JC, Soto-Quiros ME. Trends in hospitalizations and mortality from asthma in Costa Rica over a 12- to 15-year period. J Allergy Clin Immunol Pract 2014; 2(1): 85-90.
1020. SoukAloun D, Douangbouphaa V, Phetsouvanh R, et al. Rotavirus diarrhea in hospitalized children under 5 years of age in Vientiane, Lao PDR, 2009-2015. Vaccine 2018; 36(51): 7878-82.
1021. Souza ECO, Santos ESD, Rosa AM, Botelho C. Socioeconomic factors and risk for hospitalisation due to asthma in children in the municipalities of Mato Grosso State, Brazil. Cienc 2018; 23(8): 2523-32.
1022. Spiegel DA, Shrestha OP, Rajbhandary T, et al. Epidemiology of surgical admissions to a children's disability hospital in Nepal. World J Surg 2010; 34(5): 954-62.
1023. Stewart RJ, Ly S, Sar B, et al. Using a hospital admission survey to estimate the burden of influenza-associated severe acute respiratory infection in one province of Cambodia-methods used and lessons learned. Influenza other respi 2018; 12(1): 104-12.
1024. Stoesser N, Moore CE, Pocock JM, et al. Pediatric bloodstream infections in Cambodia, 2007 to 2011. Pediatr Infect Dis J 2013; 32(7): e272-6.
1025. Strabelli TMB, Botura CA, Maciel MA, Mazzutti C, Bridi A, Freitas LP. Socioeconomic profile of children hospitalized by community acquired pneumonia. Acta Scientiarum - Health Sciences 2013; 35(2): 175-9.
1026. Studart-Botto P, Martins-Junior DF, Sarmento S, Argolo L, Galvao-de-Almeida A, Miranda-Scippa A. Self-injurious behavior and related mortality in children under 10 years of age: a retrospective health record study in Brazil. Rev Bras Psiquiatr 2020; 42(1): 40-5.
1027. Sundet M, Grudziak J, Charles A, Banza L, Varela C, Young S. Paediatric road traffic injuries in Lilongwe, Malawi: an analysis of 4776 consecutive cases. Trop Doct 2018; 48(4): 316-22.
1028. Suriadji D, Wati DK, Sidiartha IGL, Suparyatha IB, Hartawan INB. Prevalence and association of cost and hospital malnutrition in Pediatric Intensive Care Unit Sanglah Hospital during 2015. Critical Care & Shock 2017; 20(1): 10-6.
1029. Susilarini NK, Haryanto E, Praptiningsih CY, et al. Estimated incidence of influenza-associated severe acute respiratory infections in Indonesia, 2013-2016. Influenza other respi 2018; 12(1): 81-7.
1030. Sutherland EK, Ansa GA, Baiden F, Quaye ENB, Amoabeng AA, Amenuveve C. Causes of death at the University of Ghana Hospital in Accra-a 37-year review (1979-2015). Int Health 2018; 10(4): 228-36.
1031. Tadesse M, Mengesha M, Tayachew A, et al. Burden and seasonality of medically attended influenza like illness (ILI) in Ethiopia, 2012 to 2017. BMC Infect Dis 2020; 20(1): 148.
1032. Takassi OE, Djadou E, Adi P, Guedenon KM, Fiawoo M. Child mortality infected with HIV1 followed in 40 pediatric care sites in Togo. Tunis Med 2017; 95(1): 23-8.
1033. Tanaka G, Faruque AS, Luby SP, Malek MA, Glass RI, Parashar UD. Deaths from rotavirus disease in Bangladeshi children: estimates from hospital-based surveillance. Pediatr Infect Dis J 2007; 26(11): 1014-8.
1034. Taniuchi M, Islam K, Sayeed MA, et al. Etiology of diarrhea requiring hospitalization in Bangladesh by quantitative PCR, 2014-2018. Clinical infectious diseases: an official publication of the Infectious Diseases Society of America 2020.
1035. Tantawy AA, El Sherif NH, Ebeid FS, El-Desouky ED. Survival analysis after diagnosis with malignancy of Egyptian adolescent patients: a single-center experience. J Pediatr Hematol Oncol 2014; 36(6): e346-52.
1036. Tantawy AA, Sallam TH, Ibrahim DM, Sallam MT, Ragab IA. Pathogenesis and prognosis of neutropenia in infants and children admitted in a university children hospital in Egypt. Pediatr Hematol Oncol 2013; 30(1): 51-9.
1037. Tassew SG, Alebachew Woldu M, Amogne Degu W, Shibeshi W. Management of hospital-acquired infections among patients hospitalized at Zewditu memorial hospital, Addis Ababa, Ethiopia: A prospective cross-sectional study. PLoS ONE 2020; 15(4): e0231949.
1038. Tate JE, Rheingans RD, O'Reilly CE, et al. Rotavirus disease burden and impact and cost-effectiveness of a rotavirus vaccination program in kenya. J Infect Dis 2009; 200 Suppl 1: S76-84.
1039. Tchidjou HK, Vescio F, Boros S, et al. Seasonal pattern of hospitalization from acute respiratory infections in Yaounde, Cameroon. J Trop Pediatr 2010; 56(5): 317-20.
1040. Teawtrakul N, Chansung K, Sirijerachai C, Wanitpongpun C, Thepsuthammarat K. The impact and disease burden of thalassemia in Thailand: a population-based study in 2010. J Med Assoc Thai 2012; 95 Suppl 7: S211-6.

1041. Teeratakulpisarn J, Uppala R, Thepsuthammarat K, Sutra S. Burden of acute lower respiratory infection in children in Thailand in 2010: have we achieved the national target in under-five morbidity and mortality? J Med Assoc Thai 2012; 95 Suppl 7: S87-96.
1042. Tefera TK, Abebe SM, Hunegnaw MT, Mekasha FG. Time to Recovery and Its Predictors among Children 6-59 Months Admitted with Severe Acute Malnutrition to East Amhara Hospitals, Northeast Ethiopia: A Multicenter Prospective Cohort Study. J Nutr Metab 2020; 2020: 5096201.
1043. Teferi M, Desta M, Yeshitela B, et al. Acute Febrile Illness Among Children in Butajira, South-Central Ethiopia During the Typhoid Fever Surveillance in Africa Program. Clinical Infectious Diseases 2019; 69: S483-S91.
1044. Tekalign S, Adera C, den Boer M, et al. Clinical features and treatment outcomes of visceral leishmaniasis patients admitted to three centers in Oromia, Ethiopia. Journal of Infection in Developing Countries 2020; 14(6.1): 42S-7S.
1045. Teles E, Moscovici L, Monteiro RA, Alves D, Laprega MR, Bellissimo-Rodrigues F. The effectiveness of a rotavirus vaccine in preventing hospitalizations and deaths presumably due to acute infectious diarrhea in Brazilian children: A quasi-experimental study. Revista da Sociedade Brasileira de Medicina Tropical 2015; 48(2): 129-35.
1046. Tesfay W, Abay M, Hintsa S, Zafu T. Length of stay to recover from severe acute malnutrition and associated factors among under-five years children admitted to public hospitals in Aksum, Ethiopia. PLoS ONE 2020; 15(9): e0238311.
1047. Teshager NW, Amare AT, Tamirat KS. Incidence and predictors of mortality among children admitted to the pediatric intensive care unit at the University of Gondar comprehensive specialised hospital, northwest Ethiopia: a prospective observational cohort study. BMJ Open 2020; 10(10): e036746.
1048. Thabet AA, Al-Kohani A, Shadoul A, et al. Characteristics of severe acute respiratory infectionassociated hospitalization in Yemen, 2014/15. East Mediterr Health J 2016; 22(7): 440-4.
1049. Thalagala N, Tissera H, Palihawadana P, et al. Costs of Dengue Control Activities and Hospitalizations in the Public Health Sector during an Epidemic Year in Urban Sri Lanka. PLoS Negl Trop Dis 2016; 10(2): e0004466.
1050. Thanni LO, Shonubi AM, Akiode O. A retrospective audit of paediatric surgical admission in a sub-urban tertiary hospital. West Afr J Med 2005; 24(1): 10-2.
1051. Thapa B, Roguski K, Azziz-Baumgartner E, et al. The burden of influenza-associated respiratory hospitalizations in Bhutan, 2015-2016. Influenza and other Respiratory Viruses 2019; 13(1): 28-35.
1052. Thean LJ, Jenney A, Engelman D, et al. Hospital admissions for skin and soft tissue infections in a population with endemic scabies: A prospective study in Fiji, 2018-2019. PLoS Negl Trop Dis 2020; 14(12): e0008887.
1053. Thiem VD, Schmidt WP, Suzuki M, et al. Animal livestock and the risk of hospitalized diarrhoea in children under 5 years in Vietnam. Trop Med Int Health 2012; 17(5): 613-21.
1054. Thomas L, Anandan S, Verghese VP, et al. Clinical characteristics, laboratory profile and outcome of children with Vibrio cholerae gastroenteritis and Vibrio cholerae bacteraemia-aretrospective single centre study. Journal of Clinical and Diagnostic Research 2020; 14(4): SC01-SC6.
1055. Thompson DC, Crooks RJ, Clasper JC, Lupu A, Stapley SA, Cloke DJ. The pattern of paediatric blast injury in Afghanistan. BMJ Mil Health 2020; 166(3): 151-5.
1056. Thriemer K, Poespoprodjo JR, Kenangalem E, et al. The risk of adverse clinical outcomes following treatment of Plasmodium vivax malaria with and without primaquine in Papua, Indonesia. PLoS Negl Trop Dis 2020; 14(11): e0008838.
1057. Thukral A, Kohli U, Lodha R, Kabra SK, Arora NK. Validation of the PELOD score for multiple organ dysfunction in children. Indian Pediatrics 2007; 44(9): 683-6.
1058. Thukral A, Lodha R, Irshad M, Arora NK. Performance of Pediatric Risk of Mortality (PRISM), Pediatric Index of Mortality (PIM), and PIM2 in a pediatric intensive care unit in a developing country. Pediatr Crit Care Med 2006; 7(4): 356-61.
1059. Tolera M, Marami D, Abate D, Dheresa M. Are Invasive Procedures and a Longer Hospital Stay Increasing the Risk of Healthcare-Associated Infections among the Admitted Patients at Hiwot Fana Specialized University Hospital, Eastern Ethiopia? Adv Prev Med 2020; 2020: 6875463.
1060. Tomczyk S, McCracken JP, Contreras CL, et al. Factors associated with fatal cases of acute respiratory infection (ARI) among hospitalized patients in Guatemala. BMC Public Health 2019; 19(1): 499.
1061. Tonial CT, Costa CAD, Andrades GRH, et al. Performance of prognostic markers in pediatric sepsis. J Pediatr (Rio J) 2020; 28: 28.
1062. Tonial CT, Costa CAD, Andrades GRH, et al. Prediction of Poor Outcomes for Septic Children According to Ferritin Levels in a Middle-Income Setting. Pediatr Crit Care Med 2020; 21(5): e259-e66.
1063. Tornheim JA, Manya AS, Oyando N, Kabaka S, Breiman RF, Feikin DR. The epidemiology of hospitalized pneumonia in rural Kenya: the potential of surveillance data in setting public health priorities. Int J Infect Dis 2007; 11(6): 536-43.
1064. Toso BRGO, Ross Cu, Sotti CW, Brisch SV, Cardoso JM. Profile of children hospitalizations by primary care sensitive conditions. Acta sci, Health sci 2016; 38(2): 231-8.

1065. Tovar MF, Ortiz W, Valderrama MA, et al. Prevalence of urinary cotinine levels in children under 5 years of age during consultations for acute respiratory disease at the emergency department of the Universidad de La Sabana clinic. BMC Pediatr 2020; 20(1): 296.
1066. Traiber C, Piva JP, Fritsher CC, et al. Profile and consequences of children requiring prolonged mechanical ventilation in three Brazilian pediatric intensive care units. Pediatr Crit Care Med 2009; 10(3): 375-80.
1067. Trainor E, Lopman B, Iturriza-Gomara M, et al. Detection and molecular characterisation of noroviruses in hospitalised children in Malawi, 1997-2007. J Med Virol 2013; 85(7): 1299-306.
1068. Tran DN, Trinh QD, Pham NTK, et al. Human rhinovirus infections in hospitalized children: clinical, epidemiological and virological features. Epidemiology & Infection 2016; 144(2): 346-54.
1069. Tran DN, Trinh QD, Pham NTK, et al. Clinical and epidemiological characteristics of acute respiratory virus infections in Vietnamese children. Epidemiology & Infection 2016; 144(3): 527-36.
1070. Tran KQ, Pham VH, Nguyen PM, et al. Lobar pneumonia and bacterial pathogens in vietnamese children. Current Pediatric Research 2020; 24(7): 247-53.
1071. Traynor MD, Hernandez MC, Clarke DL, et al. Utilization of age-adjusted shock index in a resource-strained setting. Journal of Pediatric Surgery 2019; 54(12): 2621-6.
1072. Tripathi P, Kumar R, Jain A, Ramteke PW. Hospital based surveillance for Japanese encephalitis in Lucknow, India - 2011-2013: Need for more JE vaccination? Clinical Epidemiology and Global Health 2016; 4(1): 40-4.
1073. Trotman H, Olugbuyi O, Barton M, McGregor D, Thomas S. Pneumococcal meningitis in Jamaican children. West Indian Med J 2009; 58(6): 585-8.
1074. Tuan TA, Thanh TT, Hai N, et al. Characterization of hospital and community-acquired respiratory syncytial virus in children with severe lower respiratory tract infections in Ho Chi Minh City, Vietnam, 2010. Influenza other respi 2015; 9(3): 110-9.
1075. Turner P, Turner C, Watthanaworawit W, et al. Respiratory virus surveillance in hospitalised pneumonia patients on the Thailand-Myanmar border. BMC Infect Dis 2013; 13: 434.
1076. Tuti T, Agweyu A, Mwaniki P, Peek N, English M. An exploration of mortality risk factors in non-severe pneumonia in children using clinical data from Kenya. BMC Medicine 2017; 15(1).
1077. Tyson AF, Gallaher J, Mjuweni S, Cairns BA, Charles AG. The effect of seasonality on burn incidence, severity and outcome in Central Malawi. Burns 2017; 43(5): 1078-82.
1078. Uddin Jamro B, Muhammad Channa S, Mal Makheja P, Ahmed Soomro A. Etiology, outcome and risk factors for fulminant hepatic failure in children at a tertiary care hospital, Sukkur, Pakistan. Rawal Medical Journal 2013; 38(3): 219-22.
1079. Udoh DO, Adeyemo AA. Traumatic brain injuries in children: A hospital-based study in Nigeria. Afr J Paediatr Surg 2013; 10(2): 154-9.
1080. Ujjan B, Waqas M, Khan MB, Bakhshi SK, Bari ME. Paediatric traumatic brain injury: Presentation, prognostic indicators and Outcome analysis from a tertiary care center in a developing country. JPMA J Pak Med Assoc 2016; 66(Suppl 3)(10): S65-S7.
1081. Uleanya ND, Aniwada EC, Nwokoye IC, Ndu IK, Eke CB. Relationship between Glycemic Levels and Treatment Outcome among Critically Ill Children admitted into Emergency Room in Enugu. BMC Pediatr 2017; 17(1): 126.
1082. Urena-Castro K, Avila S, Gutierrez M, Naumova EN, Ulloa-Gutierrez R, Mora-Guevara A. Seasonality of Rotavirus Hospitalizations at Costa Rica's National Children's Hospital in 2010-2015. Int J Environ Res Public Health 2019; 16(13): 30.
1083. Vashishtha VM, Nair NP, Ahmad M, Vashishtha I, Thiyagarajan V. Rotavirus Gastroenteritis in Western Uttar Pradesh, India. Indian J Pediatr 2021; 29: 29.
1084. Vísquez Hoyos P, Soto F, Pinzón D, Gonzílez D, Peña C. Description of pediatric patients with positive blood cultures from the pediatric intensive care unit at Hospital San José Bogotí, April 2012 to 2017. Infectio 2019; 23(2): 183-8.
1085. Vekaria-Hirani V, Kumar R, Musoke R. Antibiotic prescribing practices in paediatric septic shock in a tertiary care hospital in a resource limited setting: An audit. Pan African Medical Journal 2019; 34.
1086. Ventura JC, Hauschild DB, Barbosa E, et al. Undernutrition at PICU Admission Is Predictor of 60-Day Mortality and PICU Length of Stay in Critically Ill Children. J Acad Nutr Diet 2019; 12: 12.
1087. Ventura JC, Hauschild DB, Barbosa E, et al. Undernutrition at PICU Admission Is Predictor of 60-Day Mortality and PICU Length of Stay in Critically Ill Children. J Acad Nutr Diet 2020; 120(2): 219-29.
1088. Venunathan A, Philip B, Muthugounder K, Ismail R, Prakasam P. Influence of socio-demographic and clinical variables on the frequency of acute respiratory infections among under-five children. Indian Journal of Public Health Research and Development 2019; 10(9): 82-5.
1089. Verani JR, McCracken J, Arvelo W, et al. Surveillance for hospitalized acute respiratory infection in Guatemala. PLoS ONE 2013; 8(12): e83600.
1090. Vidanapathirana G, Angulmaduwa S, Munasinghe T, et al. Pneumococcal colonization among healthy and hospitalized vaccine-naive Sri Lankan children. Vaccine 2020; 38(46): 7308-15.

1091. Vieira ILV, Kupek E. Impacto da vacina pneumocócica na redução das internações hospitalares por pneumonia em crianças menores de 5 anos, em Santa Catarina, 2006 a 2014. Epidemiol serv saúde 2018; 27(4): e2017378-e.
1092. Vilaca L, Volpe FM, Ladeira RM. Accidental Poisoning in Children and Adolescents Admitted to a Referral Toxicology Department of a Brazilian Emergency Hospital. Rev 2020; 38: e2018096.
1093. Vong S, Guillard B, Borand L, et al. Acute lower respiratory infections in >= 5 year -old hospitalized patients in Cambodia, a low-income tropical country: clinical characteristics and pathogenic etiology. BMC Infect Dis 2013; 13: 97.
1094. Vosloo R, Breytenbach WJ, Salie S. An assessment of critically ill children admitted to a general high-care unit in a regional hospital in western cape, south africa. SAJCH South African Journal of Child Health 2020; 14(4): 192-9.
1095. Waitayangkoon P, Thongkam A, Benjamungkalarak T, et al. Hospital epidemiology and antimicrobial susceptibility of isolated methicillin-resistant Staphylococcus aureus: a one-year retrospective study at a tertiary care center in Thailand. Pathog Glob Health 2020; 114(4): 212-7.
1096. Warren JL, Shioda K, Kurum E, et al. Impact of Pneumococcal Conjugate Vaccines on Pneumonia Hospitalizations in High- and Low-Income Subpopulations in Brazil. Clin Infect Dis 2017; 65(11): 1813-8.
1097. Wesson HKH, Bachani AM, Mtambeka P, et al. Changing state of pediatric injuries in South Africa: An analysis of surveillance data from a Pediatric Emergency Department from 2007 to 2011. Surgery 2017; 162(6S): S4-S11.
1098. Winkler AS, Tluway A, Slottje D, Schmutzhard E, Hartl R, East African Neurosurgical Research C. The pattern of neurosurgical disorders in rural northern Tanzania: a prospective hospital-based study. World Neurosurg 2010; 73(4): 264-9.
1099. Wismawan PAR, Setiawan A, Nugraha J. Relationship between procalcitonin and c-reactive protein on pelod score in pediatric surgery sepsis patients at Dr Soetomo General Hospital Surabaya. Indian Journal of Public Health Research and Development 2021; 12(1): 370-8.
1100. Wong A, Prin M, Purcell LN, Kadyaudzu C, Charles A. Intensive Care Unit Bed Utilization and Head Injury Burden in a Resource-Poor Setting. Am Surg 2020; 86(12): 1736-40.
1101. Wong JY, Zhang W, Kargbo D, et al. Assessment of the severity of Ebola virus disease in Sierra Leone in 2014-2015. Epidemiology & Infection 2016; 144(7): 1473-81.
1102. Woori B, Kyunghoon K, Jong-Seo Y. Mortality of Children Treated in A Pediatric Intensive Care Unit Versus Other Intensive Care Units. Iranian Journal of Pediatrics 2020; 30(2): 1-6.
1103. Yadav B, Bansal A, Jayashree M. Clinical Profile and Predictors of Outcome of Pediatric Acute Respiratory Distress Syndrome in a PICU: A Prospective Observational Study. Pediatr Crit Care Med 2019; 20(6): e263-e73.
1104. Yameogo NV, Kologo KJ, Yameogo AA, et al. Annales de Cardiologie et d'Angeiologie 2014; 63(1): 7-10.
1105. Yang H, Yan C, Li M, et al. Short term effects of air pollutants on hospital admissions for respiratory diseases among children: A multi-city time-series study in China. Int J Hyg Environ Health 2021; 231: 113638.
1106. Yoshida L-M, Nguyen H-A, Watanabe K, et al. Incidence of radiologically-confirmed pneumonia and Haemophilus influenzae type b carriage before Haemophilus influenzae type b conjugate vaccine introduction in Central Vietnam. Journal of Pediatrics 2013; 163: S38-43.
1107. Yoshihara K, Le MN, Toizumi M, et al. Influenza B associated paediatric acute respiratory infection hospitalization in central vietnam. Influenza and other Respiratory Viruses 2019; 13(3): 248-61.
1108. Yucel H, Akcaboy M, Oztek-Celebi FZ, et al. Analysis of Refugee Children Hospitalized in a Tertiary Pediatric Hospital. Journal of Immigrant & Minority Health 2021; 23(1): 11-8.
1109. Zadran N, Kumar J, Ibrar A, et al. Sociodemographic and Clinical Features in Patients Presented With Accidental and Deliberate Self-Poisoning: A Comparative Study from Lady Reading Hospital Medical Teaching Institution, Peshawar, Pakistan. Cureus 2020; 12(10): e10935.
1110. Zhao Q, Li S, Coelho M, et al. Assessment of Intraseasonal Variation in Hospitalization Associated With Heat Exposure in Brazil. JAMA netw 2019; 2(2): e187901.
1111. Zuechner A, Mhada T, Majani NG, Sharau GG, Mahalu W, Freund MW. Spectrum of heart diseases in children presenting to a paediatric cardiac echocardiography clinic in the Lake Zone of Tanzania: a 7-years overview. BMC Cardiovascular Disorders 2019; 19(1): 1-6.
1112. Zurita-Cruz JN, Gutierrez-Gonzalez A, Manuel-Apolinar L, et al. Hospitalizations for viral respiratory infections in children under 2 years of age: epidemiology and in-hospital complications. BMC Pediatr 2020; 20(1): 285.

Ineligible language (not English, French, or Spanish) (69)

1. Akar T, Derinöz O, Demirel B. Drug intoxications and hospital costs. Turk Pediatri Arsivi 2007; 42(3): 103-6.
2. Altay A, Bozdayi G, Meral M, et al. [Investigation of norovirus infection incidence among 0-5 years old children with acute gastroenteritis admitted to two different hospitals in ankara, Turkey]. Mikrobiyol Bul 2013; 47(1): 98-108.
3. Andrade da Silva J, Rodrigues de Souza A, Rodrigues Feitoza A, Colletti Cavalcante TdM. TRAUMATISMO CRANIOENCEFÃLICO NO MUNICÃPIO DE FORTALEZA. Enfermagem em Foco 2017; 8(1): 22-6.

4. Andrade SSS. Estratégia saúde da família e sua relação com as internações por condições sensíveis à atenção primária em Pernambuco, no período de 2000-2014. 2016: 91-.
5. Araujo TE, Vieira SM, Carvalho PR. Stress ulcer prophylaxis in pediatric intensive care units. J Pediatr (Rio J) 2010; 86(6): 525-30.
6. Arcêncio RA, De Oliveira MF, Villa TCS. Hospitalizations for pulmonary tuberculosis in the State of São Paulo in 2004. Ciencia e Saude Coletiva 2007; 12(2): 409-17.
7. Azevedo MAFd. Hospitalizações por diarreia infecciosa em crianças menores de cinco anos e fatores associados no estado do Piauí. 2015: 66-.
8. Azevedo ZMAd, Silva DR, Dutra MVP, Elsas MICG, Barbosa-Silva MCG, Fonseca VM. Associação entre ângulo de fase, PRISM I e gravidade da sepse. Rev bras ter intensiva 2007; 19(3): 297-303.
9. Barreto JO, Nery IS, Costa MS. The Family Health Strategy and hospital admissions of children under five years in Piaui State, Brazil. [Portuguese]. 2012; 1(3): 515-26.
10. Bego BS, Pereira ML, Nogueira LS. Hospitalization due to drug poisoning in under-five-year-old children in Minas Gerais/Brazil, 2009 - 2018. Medicina (Brazil) 2020; 53(4): 370-8.
11. Bíscaro Valera R, Turrini RNT. Fatores relacionados á readmissao de pacientes em serviço hospitalar de emergência. Cienc enferm 2008; 14(2): 87-95.
12. Borges MGL, Lopes GL, Nascimento GARLd, Xavier MlB. O cuidado hospitalar na hanseníase: um perfil do estado do Pará de 2008 a 2014. Hansen int 2015; 40(1): 25-32.
13. Cardozo dos Santos Brito L, Almeida Pacheco HS, Aguiar Nascimento BS, et al. HOSPITALIZATIONS DUE TO DIARRHEA IN CHILDREN UNDER FIVE YEARS OLD. Journal of Nursing UFPE / Revista de Enfermagem UFPE 2021; 158: 309-18.
14. Catarino CF, Gomes M, Gomes S, Magluta C. Records of congenital heart disease in infants under one year in information systems on birth, hospitalization and death in Rio de Janeiro State, Brazil, 2006-2010. Epidemiol 2017; 26(3): 535-43.
15. Cavalcante DM, de Oliveira MR, Rehem TC. Hospitalizations for ambulatory care-sensitive conditions: validation study at a Hospital Information System (SIH) in the Federal District, Brazil, in 2012. Cad Saude Publica 2016; 32(3): e00169914.
16. Cruz LB, Rodrigues FO, Maksud M, Zambrano M, Raimundo F, Dornelles CTL. Estado nutricional e fatores de risco para desnutrição no atendimento nutricional pediátrico da admissão hospitalar. Rev HCPA & Fac Med Univ Fed Rio Gd do Sul 2009; 29(3): 194-9.
17. de Campos AZ, Theme-Filha MM. Hospitalization for primary care-sensitive conditions in Campo Grande, Mato Grosso do Sul state, Brazil, 2000-2009. Cadernos de Saude Publica 2012; 28(5): 845-55.
18. de Oliveira BR, Viera CS, Furtado MC, de Mello DF, de Lima RA. Profile of morbidity of children hospitalized in a public hospital: implications for nursing. [Portuguese]. 2012; 1(4): 586-93.
19. Doğan M. Tekirdağ Yöresinde Kene Tutunmuş Hastalarda Zoonotik Patojenlerin Araştırılması. Klimik Journal / Klimik Dergisi 2019; 32(3): 324-8.
20. Farias YN, Leite IDC, Siqueira MAMT, Cardoso AM. Ethnic and racial inequalities in hospital admissions due to avoidable causes in under-five Brazilian children, 2009-2014. Cadernos de saude publica 2019; 35(1S Suppl 1): e00001019.
21. Feitas JP, Ribeiro LA, Jorge MT. Pediatric victims of traffic accidents admitted to a university hospital: epidemiological and clinical aspects. [Portuguese]. 2007; 1(12): 3055-60.
22. França JCB, Ribeiro CEL, De Queiroz-Telles F. Candidemia in a Brazilian tertiary care hospital: Incidence, frequency of different species, risk factors and antifungal susceptibility. [Portuguese]. 2008; 1(1): 23-8.
23. Freitas JPP, Ribeiro LA, Jorge MT. Pediatric victims of traffic accidents admitted to a university hospital: Epidemiological and clinical aspects. Cadernos de Saude Publica 2007; 23(12): 3055-60.
24. Granzotto JA, Fonseca SS, Steffen MS, et al. Fatores relacionados à internação pediátrica em um hospital universitário da região sul do Brasil. Pediatria Pediatria (São Paulo) 2010; 32(1): 15-9.
25. Grupo Técnico de Prevenção de Acidentes e Violências. Hospital admissions due to external causes in the State of São Paulo in 2005. Revista de Saude Publica 2007; 41(1): 163-6.
26. Hanashiro M, Franco AO, Ferraro AA, Troster EJ. Care alternatives for pediatric chronic mechanical ventilation. J Pediatr (Rio J) 2011; 87(2): 145-9.
27. Hashemian H, Karambin MM, Bolokimoghadam C, Mirzazadeh M, Yahyapour R. Causes of Death in Neonates and Children in 17-Shahrivar Training Hospital of Rasht. Journal of Guilan University of Medical Sciences 2014; 23(90): 42-9.
28. Henriques TRP. Determinantes dos óbitos infantis hospitalares e não hospitalares nos municípios do Vale do Jequitinhonha, Minas Gerais. 2019: 79-.
29. Hinrichsen S, Falcão É, De Aguiar Santos Vilella T, et al. Candidemia in two tertiary care hospitals. Salud(i)Ciencia 2011; 18(7): 618-22.
30. Hinrichsen SL, Falcão É, Vilella TA, et al. Candidemia in a tertiary hospital in northeastern Brazil. [Portuguese]. 2008; 1(4): 394-8.

31. Kömürlüoğlu A, Arıkan K, Öncel EK, Kara A, Ceyhan M, Cengiz AB. Kene Tutunması ile Başvuran Çocuk Hastaların Klinik ve Laboratuvar Bulgularının Değerlendirilmesi. Journal of Pediatric Infection / Cocuk Enfeksiyon Dergisi 2017; 11(1): 7-14.
32. Kondolot M, Öztürk MK. Evaluation of the children hospitalized with pandemic H1N1 influenza. Cocuk Enfeksiyon Dergisi 2010; 4(4): 143-7.
33. Leite CEA. Leishmaniose visceral humana em Pernambuco: epidemiologia e gastoscom internações hospitalares. 2016.
34. Liu CY, Xiao Y, Xie ZD, et al. Viral etiology of acute respiratory tract infection among pediatric inpatients and outpatients from 2010 to 2012 in Beijing, China. Zhonghua Er Ke Za Zhi 2013; 51(4): 255-9.
35. Lôbo IKV, Konstantyner T, Areco KCN, Vianna RPT, Taddei JAAC Primary Health Care-Sensitive Hospitalizations in children under one year of age, from 2008 to 2014, in the State of São Paulo, Brazil. Ciencia & saude coletiva 2019; 24(9): 3213-26.
36. Loureiro MM, Rozenfeld S. Epidemiology of sickle cell disease hospital admissions in Brazil. [Portuguese]. 2005; 1(6): 943-9.
37. Lunardi R, Santos RV, Coimbra Júnior CEA. Morbidade hospitalar de indígenas Xavante, Mato Grosso, Brasil (2000-2002). Rev bras epidemiol 2007; 10(4): 441-52.
38. Macedo MP, Souza LCD, Corrêa RGCF, Lopes FF. Aspects of dental care for patients hospitalized in a medical clinic from a university hospital. ABCS health sci 2020; 45: [1-5].
39. Martins CBdG, Andrade SMd. Acidentes com corpo estranho em menores de 15 anos: análise epidemiológica dos atendimentos em pronto-socorro, internações e óbitos. Cad. saúde pública 2008; 24(9): 1983-90.
40. Mattei A, Angelone AM, Michetti M, et al. Epidemiological impact of RV gastroenteritis in the Abruzzo Region: SDO analysis. Ann Ig 2009; 21(1): 41-9.
41. Melo WAd, Marcon SS, Uchimura TT. A hospitalização de crianças na perspectiva de seus acompanhantes. Rev enferm UERJ 2010; 18(4): 565-71.
42. Milanesi K, Collet N, Oliveira BRG, Vieira CS. Sofrimento psíquico da família de crianças hospitalizadas. Rev bras enferm 2006; 59(6): 769-74.
43. Molina RCM, Marcon SS, Uchimura TT, Lopes EP. Caracterização das internações em uma unidade de terapia intensiva pediátrica, de um hospital-escola da região sul do Brasil. Ciênc. cuid. Saúde 2008; 7(supl.1): 112-20.
44. Mota AdM. Óbitos e hospitalizações associados à infecção pelo vírus varicela zoster no período anterior à introdução da vacina tetraviral no Programa Nacional de Imunizações no Brasil. 2014: xiv,68-xiv,
45. Nardocci AC, de Freitas CU, de Leon ACMP, Junger WL, Gouveia NC, Air pollution and respiratory and cardiovascular diseases: A time series study in Cubatão, São Paulo State, Brazil. 2013. p. 1867-7
46. Nascimento LF, Pereira LA, Braga AL, Modolo MC, Carvalho JA, Jr. Effects of air pollution on children's health in a city in Southeastern Brazil. [Portuguese]. 2006; 1(1): 77-82.
47. Nunes Da Silva E, Ribeiro H. Urban atmospheric environment and hospital admission for children in the city of Sao Paulo, Brazil. Revista de Salud Ambiental 2013; 13(1): 30-6.
48. Onder JD, Lopes CLS, Sechi FL, Sander MB, Eckert GU. Perfil dos pacientes internados por bronquiolite viral aguda em uma unidade de terapia intensiva em hospital infantil do sul do Brasil. Rev AMRIGS 2014; 58(2): 130-4.
49. Pacheco MD. Evolução das taxas de hospitalização e mortalidade por doença pneumocócica invasiva em municípios da Região Sudeste do Brasil, no período de 2005 a 2015. 2018: 52 f- f.
50. Paesi S, Magrini FE. Incidence of acute diarrheal disease in Caxias do Sul, Rio Grande do Sul, Brazil, in a 10-year historical series. Scientia Medica 2015; 25(2).
51. Pazó RG, Frauches DdO, Galvêas DbP, Stefenoni AV, Cavalcante ELB, Pereira-Silva FH. Internações por condições sensíveis à atenção primária no Espírito Santo: estudo ecológico descritivo no período 2005-2009. Epidemiol. serv. saúde 2012; 21(2): 275-82.
52. Pedraza DF, Araujo EMNd. Internações das crianças brasileiras menores de cinco anos: revisão sistemática da literatura. Epidemiol. serv. saúde 2017; 26(1): 169-82.
53. Polita NB, Ferrari RA, de Moraes PS, Sant'Anna FL, Tacla MT. Congenital anomalies: hospitalization in a pediatric unit. Rev 2013; 31(2): 205-10.
54. Riccetto AGL, Ribeiro JD, Da Silva MTN, Baracat ECE. Prognostic factors for mechanical ventilation in infants with acute lower respiratory disease. Revista da Associacao Medica Brasileira 2006; 52(5): 342-6.
55. Rossini FP, Ferraz CA. Study of urgency admissions focusing on demographic-epidemiology in a public hospital. Rev Rene 2009; 10(4): 77-86.
56. Salomão Jr JB, Gardinassi LGA, Simas PVM, et al. Human respiratory syncytial virus in children hospitalized for acute lower respiratory infection. Jornal de Pediatria 2011; 87(3): 219-24.
57. Santos ILFd, Gaíva MAM, Abud SM, Ferreira SMB. Hospitalização de crianças por condições sensíveis à atenção primária. Cogitare enferm 2015; 20(1): 171-9.
58. Sarni RO, Carvalho MdF, Monte CM, Albuquerque ZP, Souza FI. Anthropometric evaluation, risk factors for malnutrition, and nutritional therapy for children in teaching hospitals in Brazil. J Pediatr (Rio J) 2009; 85(3): 223-8.

59. Sha L, Zhu RN, Cao L, et al. Analysis of clinical manifestations of hospitalized children infected with seasonal influenza A virus and 2009 novel influenza A (H1N1) virus in Beijing. Zhonghua Er Ke Za Zhi 2011; 49(7): 539-44.
60. Silva Cardoso C, Menezes Pádua C, Rodrigues-Júnior AA, et al. Contribution of hospitalizations for primary care-sensitive conditions to the profile admissions in the public health care system. Pan American Journal of Public Health 2013; 34(4): 227-34.
61. Silva AC, Cota FV, T H, Dutra JS, Santos YRd, Romano MCC. Hospitalização por asma em crianças no município de Divinópolis, Minas Gerais. Rev enferm Cent-Oeste Min 2014; 4(3): 1290-9.
62. Silva MAI, Pan R, Melo L, de Bortoli PS, Nascimento LC. Profile of care delivered to children and adolescents victims of external causes of morbimortality, 2000-2006. Revista Gaucha de Enfermagem 2010; 31(2): 351-8.
63. Silva SR, Mello LM, Silva AS, Nunes AA. Impact of the pneumococcal 10-valent vaccine on reducing hospitalization for community-acquired pneumonia in children. Rev 2016; 34(4): 418-24.
64. Silva YOM, Lima MG. Incidence of hospitalizations due to adverse drug events in Minas Gerais, Brazil. Scientia Medica 2017; 27(1).
65. Silveira EC. Profile of hospitalizations for viral encephalitis in Brazil in 2018 per state, sex, and age group. Medicina (Brazil) 2020; 53(3): 268-74.
66. Slompo Freitas J, Nolasco Chaves MM, Raksa VP, Muller Larocca L. HOSPITAL ADMISSIONS OF ADOLESCENTS DUE TO PRIMARY CARE SENSITIVE CONDITIONS IN A HEALTH REGION. Cogitare Enfermagem 2018; 23(4): 48-57.
67. Teixeira CLDS, Klein CH, Bloch KV, Coeli CM. Probable cause of death after reclassification of ill-defined causes on hospital admissions forms in the Unified National Health System, Rio de Janeiro, Brazil. Cadernos de Saude Publica 2006; 22(6): 1315-24.
68. Teixeira CL, Klein CH, Bloch KV, Coeli CM. Probable cause of death after reclassification of ill-defined causes on hospital admissions forms in the Unified National Health System, Rio de Janeiro, Brazil. [Portuguese]. 2006; 1(6): 1315-24.
69. Veras TN, Sandim G, Mundim K, Petrauskas R, Cardoso G, Agostin J. Perfil epidemiológico de pacientes pediátricos internados com pneumonia. Sci med 2010; 20(4).

Ineligible numerator (n=134)

1. Abejew AA, Tamir AS, Kerie MW. Retrospective analysis of mortalities in a tertiary care hospital in Northeast Ethiopia. BMC Res Notes 2014; 7: 46.
2. Agrawal S, Sachdev A, Gupta D, Chugh K. Platelet counts and outcome in the pediatric intensive care unit. Indian Journal of Critical Care Medicine 2008; 12(3): 102-8.
3. Ali AA, Al-Hasnawy HH, Nabeel N. Molecular detection of M. Pneumoniae in pneumonia patients in Al-Hilla city, Iraq. Indian Journal of Public Health Research and Development 2019; 10(8): 2520-3.
4. Ameyaw E, Amponsah-Achiano K, Yamoah P, Chanoine JP. Abnormal blood glucose as a prognostic factor for adverse clinical outcome in children admitted to the paediatric emergency unit at komfo anokye teaching hospital, kumasi, ghana. Int J Pediatr 2014; 2014: 149070.
5. Andrade PHS, Lobo IMF, da Silva WB. Risk factors for adverse drug reactions in pediatric inpatients: A cohort study. PLoS ONE 2017; 12(8): e0182327.
6. Anwar H, Saleem AF, Zaidi S, Haider SR. Experience of pediatric rapid response team in a tertiary care hospital in Pakistan. Indian J Pediatr 2010; 77(3): 273-6.
7. Appiah J, Salie S, Argent A, Morrow B. Characteristics, course and outcomes of children admitted to a paediatric intensive care unit after cardiac arrest. Southern African Journal of Critical Care 2018; 34(2): 58-64.
8. Araujo da Silva AR, Jaszkowski E, Schober T, et al. Patterns of antimicrobial consumption in neonatal and pediatric intensive care units in Germany and Brazil. Eur J Clin Microbiol Infect Dis 2019; 31: 31.
9. Azevedo ZM, Moore DB, Lima FC, et al. Tumor necrosis factor (TNF) and lymphotoxin-alpha (LTA) single nucleotide polymorphisms: importance in ARDS in septic pediatric critically ill patients. Hum Immunol 2012; 73(6): 661-7.
10. Babu A, Rattan A, Ranjan P, et al. Are falls more common than road traffic accidents in pediatric trauma? Experience from a Level 1 trauma centre in New Delhi, India. Chin J Traumatol 2016; 19(2): 75-8.
11. Bachou H, Tumwine JK, Mwadime RK, Tylleskar T. Risk factors in hospital deaths in severely malnourished children in Kampala, Uganda. BMC Pediatr 2006; 6: 7.
12. Bains HS, Kumar Soni R. A Simple Clinical Score 'TOPRS' to Predict Outcome in Pediatric Emergency Department in a Teaching Hospital in India. Iranian Journal of Pediatrics 2012; 22(1): 97-101.
13. Bar-On E, Lebel E, Blumberg N, Sagi R, Kreiss Y. Pediatric Orthopedic Injuries Following an Earthquake. Journal of Trauma Nursing 2015; 22(4): 223-8.
14. Barbosa AP, Cunha AJLAd. Neonatal and pediatric intensive care in Rio de Janeiro State, Brazil: an analysis of bed distribution, 1997 and 2007. Cad saúde pública 2011; 27(supl.2): s263-s71.

15. Ben Jaballah N, Bouziri A, Mnif K, Hamdi A, Khaldi A, Kchaou W. Epidemiology of hospital-acquired bloodstream infections in a Tunisian pediatric intensive care unit: a 2-year prospective study. Am J Infect Control 2007; 35(9): 613-8.
16. Bergkvist E, Zimunhu T, Mbanje C, Hagander L, Muguti GI. Nutritional status and outcome of surgery: A prospective observational cohort study of children at a tertiary surgical hospital in Harare, Zimbabwe. J Pediatr Surg 2021; 56(2): 368-73.
17. Bhattacharyya A, Chatterjee C, Chatterjee S, Dasgupta S. A Comparative Study of the Management Decisions by IMNCI Algorithm and by Pediatricians of a Teaching Hospital for the Children Between 2 Months to 5 Years. Indian J 2012; 37(3): 174-9.
18. Bishop-Williams KE, Berrang-Ford L, Sargeant JM, et al. Understanding Weather and Hospital Admissions Patterns to Inform Climate Change Adaptation Strategies in the Healthcare Sector in Uganda. Int J Environ Res Public Health 2018; 15(11): 29.
19. Blanke CH, Naisabha GB, Lange CR. Traditional medicine, delays and hospital mortality of children under five years of age in the rural district of Kigoma, western Tanzania: an observational hospital-based study. Trop Doct 2008; 38(3): 150-2.
20. Bowley DM, Rogers TN, Meyers T, Pitcher G. Surgeons are failing to recognize children with HIV infection. J Pediatr Surg 2007; 42(2): 431-4.
21. Briegleb C, Sudfeld CR, Smith ER, et al. Predictors of Hospitalization During the First Year of Life among 31999 Tanzanian Infants. J Trop Pediatr 2015; 61(5): 317-28.
22. Burle SS, Jain S. Unusual clustering of diseases and manifestations in pediatric admissions of a tertiary care center. Indian Pediatr 2006; 43(12): 1033-41.
23. Buys H, Muloiwa R, Westwood C, Richardson D, Cheema B, Westwood A. An adapted triage tool (ETAT) at Red Cross War Memorial Children's Hospital Medical Emergency Unit, Cape Town: An evaluation. South African Medical Journal 2013; 103(3): 161-5.
24. Cabral FC, Garcia PCR, Mattiello R, et al. Influence of acute kidney injury defined by the pediatric risk, injury, failure, loss, end-stage renal disease score on the clinical course of PICU patients. Pediatric Critical Care Medicine 2015; 16(8): e275-e82.
25. Campos-Mino S, Sasbon JS, von Dessauer B. Pediatric intensive care in Latin America. Med 2012; 36(1): 3-10.
26. Carter K, Tovu V, Langati JT, et al. Causes of death in Vanuatu. Popul Health Metr 2016; 14: 7.
27. Chew Beb FJ, Pacheco P, Juírez AB, et al. Caracterí-sticas clí-nicas de la niñez menor de cinco años con desnutrición aguda complicada hospitalizados en 36 hospitales de la red de servicios de Salud Pública, año 2014. Guatem pediatr 2015; 1(2): 27-35.
28. Costa LQ, Pinto EPJ, Silva MG. Time trends in hospitalizations for Ambulatory Care Sensitive Conditions among children under five years old in Ceara, Brazil, 2000-2012. Epidemiol 2017; 26(1): 51-60.
29. Crump JA, Ramadhani HO, Morrissey AB, et al. Invasive bacterial and fungal infections among hospitalized HIV-infected and HIV-uninfected children and infants in northern Tanzania. Trop Med Int Health 2011; 16(7): 830-7.
30. Cuervo-Suarez MI, Claros-Hulbert A, Manzano-Nunez R, Munoz M, Garcia X. Pediatric Palliative Care During End of Life: A Privilege of a Few in a Tertiary Referral Hospital From Colombia. Am J Hosp Palliat Care 2020; 37(8): 636-40.
31. Daga S, Daga A, Mhatre S. Antibiotic Therapy for Children with Diarrhea in a Low-Resource Setting: A Syndromic Approach. Pediatric health med 2020; 11: 95-100.
32. Dame JA, Beylis N, Nuttall J, Eley B. Pseudomonas aeruginosa bloodstream infection at a tertiary referral hospital for children. BMC Infect Dis 2020; 20(1): 729.
33. de Mello MJ, de Albuquerque MF, Ximenes RA, et al. Factors associated with time to acquisition of bloodstream infection in a pediatric intensive care unit. Infect Control Hosp Epidemiol 2010; 31(3): 249-55.
34. Dekker-Boersema J, Hector J, Jefferys LF, et al. Triage conducted by lay-staff and emergency training reduces paediatric mortality in the emergency department of a rural hospital in Northern Mozambique. African Journal of Emergency Medicine 2019.
35. Diouf E, Beye MD, Diop Ndoye M, et al. Nosocomial ventilator-associated pneumonia in a tropical intensive care unit. Dakar Med 2006; 51(2): 81-8.
36. Dramowski A, Coovadia A, Meyers T, Goga A. Identifying missed opportunities for early intervention among HIV-infected paediatric admissions at Chris Hani Baragwanath hospital, Soweto, South Africa. Southern African Journal of HIV Medicine 2011; (42): 16-23.
37. Dramowski A, Whitelaw A, Cotton MF. Burden, spectrum, and impact of healthcare-associated infection at a South African children's hospital. J Hosp Infect 2016; 94(4): 364-72.
38. El-Nawawy A, Ashraf GA, Antonios MAM, Meheissen MA, El-Alfy MMR. Incidence of Multidrug-Resistant Organism Among Children Admitted to Pediatric Intensive Care Unit in a Developing Country. Microb Drug Resist 2018; 24(8): 1198-206.

39. El-Sahrigy SAF, Shouman MG, Ibrahim HM, et al. Prevalence and Anti-Microbial Susceptibility of Hospital Acquired Infections in Two Pediatric Intensive Care Units in Egypt. Open Access Maced J Med Sci 2019; 7(11): 1744-9.
40. Elusiyan JB, Adejuyigbe EA, Adeodu OO. Hypoglycaemia in a Nigerian paediatric emergency ward. J Trop Pediatr 2006; 52(2): 96-102.
41. Ferrer APS, Brentani A, Grisi SJFE. Primary care evaluation in the Brazilian context: Effects of the health care model transition. Current Pediatric Research 2016; 20(1-2): 118-25.
42. Ferrer AP, Brentani AV, Sucupira AC, Navega AC, Cerqueira ES, Grisi SJ. The effects of a people-centred model on longitudinality of care and utilization pattern of healthcare services--Brazilian evidence. Health Policy Plan 2014; 29 Suppl 2: ii107-13.
43. Fonseca GA, Normando PG, Loureiro LVM, et al. Reduction in the Number of Procedures and Hospitalizations and Increase in Cancer Mortality during the COVID-19 Pandemic in Brazil. Journal of Global Oncology 2021; 7: 4-9.
44. Ganesan RG, Das S, Parameswara N, Biswal N, Pabhu A. Survival after in-hospital cardiac arrest among paediatric patients-A descriptive study. Journal of Clinical and Diagnostic Research 2018; 12(2): SC04-SC9.
45. Garcí-a Munitis P, de Abreu M, Antonietti L, et al. Referral and counter-referral system between secondary and tertiary care facilities in a children's hospital network of Greater Buenos Aires. Arch argent pediatr 2013; 111(5): 404-10.
46. Garcia PC, Eulmesekian P, Branco RG, et al. External validation of the paediatric logistic organ dysfunction score. Intensive Care Med 2010; 36(1): 116-22.
47. Garde A, Zhou G, Raihana S, et al. Respiratory rate and pulse oximetry derived information as predictors of hospital admission in young children in Bangladesh: a prospective observational study. BMJ Open 2016; 6(8): e011094.
48. Gonzalez-Velez AE, Mejia CCC, Padilla EL, et al. Ambulatory care sensitive conditions hospitalization for emergencies rates in Colombia. Rev Saude Publica 2019; 53: 36.
49. Haque A, Rizvi A, Bano S. Outcome of in-hospital pediatric cardiopulmonary arrest from a single center in Pakistan. Indian J Pediatr 2011; 78(11): 1356-60.
50. Hassan MH, Zaghloul AA, Mokhtar SA. The probability distribution of attendance to hospital emergency units for school students in Alexandria. J Egypt Public Health Assoc 2005; 80(1-2): 127-51
51. Haven N, Dobson AE, Yusuf K, et al. Community-Based Health Insurance Increased Health Care Utilization and Reduced Mortality in Children Under-5, Around Bwindi Community Hospital, Uganda Between 2015 and 2017. Front 2018; 6: 281.
52. Hearn P, Miliya T, Seng S, et al. Prospective surveillance of healthcare associated infections in a Cambodian pediatric hospital. Antimicrob 2017; 6: 16.
53. Henry O, Amata AO. A two-year review of admissions to the Intensive Care Unit of the Georgetown Public Hospital Corporation, Guyana. West Indian Medical Journal 2017; 66(6).
54. Herbert G, Ndiritu M, Idro R, Makani JB, Kitundu J. Analysis of the indications for routine lumbar puncture and results of cerebrospinal fluid examination in children admitted to the paediatric wards of two hospitals in East Africa. Tanzan 2006; 8(1): 7-10.
55. Hill PC, Onyeama CO, Ikumapayi UN, et al. Bacteraemia in patients admitted to an urban hospital in West Africa. BMC Infect Dis 2007; 7: 2.
56. Hogan B, Eibach D, Krumkamp R, et al. Malaria Coinfections in Febrile Pediatric Inpatients: A Hospital-Based Study From Ghana. Clinical Infectious Diseases 2018; 66(12): 1838-45.
57. Hongsuwan M, Srisamang P, Kanoksil M, et al. Increasing incidence of hospital-acquired and healthcare-associated bacteremia in northeast Thailand: a multicenter surveillance study. PLoS ONE 2014; 9(10): e109324.
58. Hoque MM, Ahmed ASM, Quaderi H. Prevalence and risk factors of iron deficiency anaemia in children admitted in a tertiary care hospital of Bangladesh. Journal of Nepal Paediatric Society 2015; 35(1): 38-43.
59. Ijaz N, Strehlow M, Ewen Wang N, et al. Epidemiology of patients presenting to a pediatric emergency department in Karachi, Pakistan. BMC emerg 2018; 18(1): 22.
60. Ikefuna AN, Emodi IJ. Some characteristics of paediatric admissions at the University of Nigeria Teaching Hospital Enugu-Nigeria. Niger J Clin Pract 2007; 10(3): 216-9.
61. Ilesanmi OS, Oladimeji A, Adewuyi PA, et al. Trends in mortality at Federal Medical Centre, Owo, Ondo State, Nigeria 2006-2014. Pan Afr Med J 2019; 32(Suppl 1): 16.
62. Jacinto A, Masembe V, Tumwesigye NM, Harding R. The prevalence of life-limiting illness at a Ugandan National Referral Hospital: a 1-day census of all admitted patients. BMJ support 2015; 5(2): 196-9.
63. Jain R, Awasthi S, Awasthi A. IMCI approach in tertiary hospitals, India. Indian J Pediatr 2009; 76(7): 725-7.
64. Jan AZ, Zahid B, Ahmad S, Gul Z. Pancytopenia in children: A 6-year spectrum of patients admitted to Pediatric Department of Rehman Medical Institute, Peshawar. Pakistan Journal of Medical Sciences 2013; 29(5).
65. Jiménez Garcí-a R, Alfonso Novo Lz, Santana Porbén S, Piñeiro Ferníndez E, Pérez Martí-nez E, Domí-nguez Jiménez R. Evolución de la desnutrición hospitalaria. Rev cuba pediatr 2014; 86(3): 298-307.
66. Johnson T, Gaus D, Herrera D. Emergency department of a rural hospital in Ecuador. Western Journal of Emergency Medicine 2016; 17(1): 66-72.

67. Keerthiwansa J, Gajealan S, Sivaraja S, Subashini KY. Malnutrition and anaemia among hospitalised children in Vavuniya. Ceylon Med J 2014; 59(4): 141-3.
68. Khadka A, Rayamajhi H, Shrestha S. Evaluation of the prescription pattern of antimicrobial agents in hospitalised paediatric patients in a tertiary care hospital of kathmandu, Nepal. Journal of Nepal Paediatric Society 2018; 38(3): 157-62.
69. Khan NU, Razzak JA, Saleem AF, Khan UR, Mir MU, Aashiq B. Unplanned return visit to emergency department: a descriptive study from a tertiary care hospital in a low-income country. Eur J Emerg Med 2011; 18(5): 276-8.
70. Krishnamurthy S, Mondal N, Narayanan P, Biswal N, Srinivasan S, Soundravally R. Incidence and etiology of acute kidney injury in southern India. Indian J Pediatr 2013; 80(3): 183-9.
71. Kumar D, Swarnim S, Sikka G, et al. Factors Associated with Readmission of Pediatric Patients in a Developing Nation. Indian J Pediatr 2019; 86(3): 267-75.
72. Lopes JM, Goulart EM, Siqueira AL, Fonseca IK, Brito MV, Starling CE. Nosocomial infections in Brazilian pediatric patients: using a decision tree to identify high mortality groups. Braz J Infect Dis 2009; 13(2): 111-7.
73. Lozano C J, Yíñez P L, Lapadula A M, et al. Infección por metapneumovirus humano en niños hospitalizados por una enfermedad respiratoria aguda grave: descripción clínico- epidemiológica. Rev chil enferm respir 2009; 25(4): 211-7.
74. Macias-Rosales R, Vasquez-Garibay EM, Larrosa-Haro A, Rojo-Chavez M, Bernal-Virgen A, Romo-Rubio H. Secondary malnutrition and overweight in a pediatric referral hospital: associated factors. J Pediatr Gastroenterol Nutr 2009; 48(2): 226-32.
75. Magwenzi MT, Gudza-Mugabe M, Mujuru HA, Dangarembizi-Bwakura M, Robertson V, Aiken AM. Carriage of antibiotic-resistant Enterobacteriaceae in hospitalised children in tertiary hospitals in Harare, Zimbabwe. Antimicrob 2017; 6: 10.
76. Mandomando I, Bassat Q, Sigaúque B, et al. Invasive salmonella infections among children from Rural Mozambique, 2001-2014. Clinical Infectious Diseases 2015; 61: S339-S45.
77. Mandomando I, Macete E, Sigauque B, et al. Invasive non-typhoidal Salmonella in Mozambican children. Trop Med Int Health 2009; 14(12): 1467-74.
78. Mato CN, Onwuchekwa AC, Aggo AT. Pattern of admissions to the University of Port Harcourt Teaching Hospital intensive care unit - A 10-year analysis. Southern African Journal of Critical Care 2009; 25(1): 10-5.
79. Mazhar A, Rehman A, Sheikh MA, Naeem MM, Qaisar I, Mazhar M. Neonates--a neglected paediatric age group. JPMA J Pak Med Assoc 2011; 61(7): 625-8.
80. Mehta Y, Jaggi N, Rosenthal VD, et al. Device-Associated Infection Rates in 20 Cities of India, Data Summary for 2004-2013: Findings of the International Nosocomial Infection Control Consortium. Infection Control & Hospital Epidemiology 2016; 37(2): 172-81.
81. Memon SF, Khan AA, Mustafa S, Farooqui MT, Khan BS, Jalees S. Intensive Care Units of a Tertiary Care Hospital: A Detailed Study of Patient Symptomatology. Cureus 2019; 11(1): e3988.
82. Méndez E, Benigno M, Duffau T, Gastón. Triage, temperatura, frecuencia cardíaca y saturación de O2, en la clasificación de pacientes en un servicio de urgencia pediátrico. Rev chil pediatr 2009; 80(6): 528-33.
83. Meyers T, Dramowski A, Schneider H, Gardiner N, Kuhn L, Moore D. Changes in pediatric HIV-related hospital admissions and mortality in Soweto, South Africa, 1996-2011: light at the end of the tunnel? J Acquir Immune Defic Syndr 2012; 60(5): 503-10.
84. Misganaw A, Mariam DH, Araya T, Ayele K. Patterns of mortality in public and private hospitals of Addis Ababa, Ethiopia. BMC Public Health 2012; 12: 1007.
85. Moïsi JC, Gatakaa H, Berkley JA, et al. Excess child mortality after discharge from hospital in Kilifi, Kenya: a retrospective cohort analysis. Bulletin of the World Health Organization 2011; 89(10): 725-32A.
86. Mogeni P, Twahir H, Bandika V, et al. Diagnostic performance of visible severe wasting for identifying severe acute malnutrition in children admitted to hospital in Kenya. Bull World Health Organ 2011; 89(12): 900-6.
87. Morales-Rios O, Cicero-Oneto C, Garcia-Ruiz C, et al. Descriptive study of adverse drug reactions in a tertiary care pediatric hospital in Mexico from 2014 to 2017. PLoS ONE 2020; 15(3): e0230576.
88. Mpimbaza A, Sears D, Sserwanga A, et al. Admission Risk Score to Predict Inpatient Pediatric Mortality at Four Public Hospitals in Uganda. PLoS ONE 2015; 10(7): e0133950.
89. Munoz Esparza NC, Vasquez-Garibay EM, Larrosa-Haro A, Romero-Velarde E. Socio-demographic variables and underlying pathologies associated to nutritional status of hospitalized children in a secondary-tertiary level hospital. Nutr Hosp 2018; 35(2): 286-93.
90. Mwale K, Siziya S. Intestinal Infestations in Under-Five Children in Zambia. Int 2015; 4(2): 40-6.
91. Nadjm B, Amos B, Mtove G, et al. WHO guidelines for antimicrobial treatment in children admitted to hospital in an area of intense Plasmodium falciparum transmission: prospective study. Bmj 2010; 340: c1350.
92. Ndegwa LK, Katz MA, McCormick K, et al. Surveillance for respiratory health care-associated infections among inpatients in 3 Kenyan hospitals, 2010-2012. Am J Infect Control 2014; 42(9): 985-90.

93. Ndiaye O, Fall FT, Faye PM, Thiongane A, Fall AL. Impact of COVID-19 pandemic on health care activities in the Department of Pediatrics at the Albert Royer National Children's Hospital: preliminary study comparing the first quarter of 2019 and 2020. Pan Afr Med J 2020; 36: 162.
94. Ngwalangwa F, Phiri CHA, Dube Q, Langton J, Hildenwall H, Baker T. Risk Factors for Mortality in Severely Ill Children Admitted to a Tertiary Referral Hospital in Malawi. Am J Trop Med Hyg 2019; 101(3): 670-5.
95. Nhabomba C, Chicumbe S, Muquingue H, et al. Clinical and operational factors associated with low pediatric inpatient HIV testing coverage in Mozambique. Public health action 2019; 9(3): 113-9.
96. Ntuli ST, Malangu N, Alberts M. Causes of deaths in children under-five years old at a tertiary hospital in Limpopo province of South Africa. Glob J Health Sci 2013; 5(3): 95-100.
97. O'Meara WP, Bejon P, Mwangi TW, et al. Effect of a fall in malaria transmission on morbidity and mortality in Kilifi, Kenya. Lancet 2008; 372(9649): 1555-62.
98. Okiro EA, Bitira D, Mbabazi G, et al. Increasing malaria hospital admissions in Uganda between 1999 and 2009. BMC Med 2011; 9: 37.
99. Olson D, Preidis GA, Milazi R, et al. Task shifting an inpatient triage, assessment and treatment programme improves the quality of care for hospitalised Malawian children. Trop Med Int Health 2013; 18(7): 879-86.
100. Onankpa B, Airede L, Paul I, Dorcas I. Pattern of pediatric HIV/AIDS: a five-year experience in a tertiary hospital. 2008; 1(7): 821-5.
101. Onyiriuka AN. Discharge of hospitalized under-fives against medical advice in Benin City, Nigeria. Niger J Clin Pract 2007; 10(3): 200-4.
102. Onyiriuka AN, Adeniran KA, Onyiriuka EP. Prevalence of hypoglycemia among patients presenting with cholestasis of infancy in a nigerian teaching hospital. Oman med 2012; 27(4): 329-32.
103. Pedrosa AK, Pinto FJ, Lins LD, Deus GM. Blood transfusion reactions in children: associated factors. J Pediatr (Rio J) 2013; 89(4): 400-6.
104. Ponnarmeni S, Angurana SK, Singhi S, et al. Vitamin D deficiency in critically ill children with sepsis. Paediatrics and International Child Health 2016; 36(1): 15-21.
105. Preidis GA, McCollum ED, Mwansambo C, Kazembe PN, Schutze GE, Kline MW. Pneumonia and malnutrition are highly predictive of mortality among African children hospitalized with human immunodeficiency virus infection or exposure in the era of antiretroviral therapy. J Pediatr 2011; 159(3): 484-9.
106. Prezotto KH, Chaves MM, Mathias TA. [Hospital admissions due to ambulatory care sensitive conditions among children by age group and health region]. Rev Esc Enferm USP 2015; 49(1): 44-53.
107. Punchak M, Hall K, Seni A, et al. Epidemiology of Disease and Mortality From a PICU in Mozambique. Pediatr Crit Care Med 2018; 19(11): e603-e10.
108. Ramtake AK, Mudda V, Garampalli SS, Umesh SR. A prospective study to ascertain the profile of unnatural deaths at Basaveshwara teaching and general hospital mortuary, Gulbarga (Kalaburagi) - A research paper. Medico-Legal Update 2017; 17(2): 17-21.
109. Rathore V, Bansal A, Singhi SC, Singhi P, Muralidharan J. Survival and neurological outcome following in-hospital paediatric cardiopulmonary resuscitation in North India. Paediatr Int Child Health 2016; 36(2): 141-7.
110. Rice B, Periyanayagam U, Chamberlain S, et al. Mortality in Children Under Five Receiving Nonphysician Clinician Emergency Care in Uganda. Pediatrics 2016; 137(3): e20153201.
111. Sahu SK, Das P, Behera JR. Managing pediatric haemophagocytic lymphohistiocytosis (HLH) in a resource limited setting-a 3 years experience. International Journal of Research in Pharmaceutical Sciences 2020; 11(4): 5965-70.
112. Salih KMA, Elfaki OA, Hamid YHM, Eldouch WMA, Diab M, Abdelgadir SO. Prevalence of Helicobacter Pylori among Sudanese children admitted to a specialized children hospital. Sudan 2017; 17(1): 14-8.
113. Sambany E, Pussard E, Rajaonarivo C, Raobijaona H, Barennes H. Childhood dysglycemia: prevalence and outcome in a referral hospital. PLoS ONE 2013; 8(5): e65193.
114. Samuels RJ, Moon TD, Starnes JR, et al. Lassa Fever among Children in Eastern Province, Sierra Leone: A 7-year Retrospective Analysis (2012-2018). Am J Trop Med Hyg 2020; 23: 23.
115. Sarangi R, Pradhan S, Dhanawat A, Patanayak R, Benia G. Thrombocytosis in children: Clinico-hematological profile from a single centre in Eastern India. J 2018; 10(1): 34-7.
116. Sears D, Mpimbaza A, Kigozi R, et al. Quality of inpatient pediatric case management for four leading causes of child mortality at six government-run Ugandan hospitals. PLoS ONE 2015; 10(5): e0127192.
117. Seni J, Mapunjo SG, Wittenauer R, et al. Antimicrobial use across six referral hospitals in Tanzania: a point prevalence survey. BMJ Open 2020; 10(12): e042819.
118. Siddharth V, Gupta SK, Lodha R, Satpathy S. Quality of care in a paediatric intensive care unit of a tertiary care teaching hospital in New Delhi. Journal of Nepal Paediatric Society 2017; 37(2): 168-73.
119. Singh HK, Gupte N, Kinikar A, et al. High Rates of All-cause and Gastroenteritis-related Hospitalization Morbidity and Mortality among HIV-exposed Indian Infants. BMC Infectious Diseases 2011; 11.
120. Singh K, Speizer I, Barker PM, et al. Evaluating the impact of a hospital scale-up phase of a quality improvement intervention in Ghana on mortality for children under five. Int J Qual Health Care 2019; 19: 19.

121. Sirachainan N, Chuansumrit A, Angchaisuksiri P, Pakakasama S, Hongeng S, Kadegasem P. Venous thromboembolism in Thai children. Pediatr Hematol Oncol 2007; 24(4): 245-56.
122. Sodemann M, Biai S, Jakobsen MS, Aaby P. Knowing a medical doctor is associated with reduced mortality among sick children consulting a paediatric ward in Guinea-Bissau, West Africa. Trop Med Int Health 2006; 11(12): 1868-77.
123. Sylverken J, Robison JA, Osei-Akoto A, et al. Decreased Mortality After Establishing a Pediatric Emergency Unit at an Urban Referral Hospital in Ghana. Pediatr Emerg Care 2019; 01: 01.
124. Tack B, Phoba MF, Barbe B, et al. Non-typhoidal Salmonella bloodstream infections in Kisantu, DR Congo: Emergence of O5-negative Salmonella Typhimurium and extensive drug resistance. PLoS Negl Trop Dis 2020; 14(4): e0008121.
125. Tarchouna M, Ferjani A, Tilouche S, et al. Screening at admission for carrier prevalence of multidrug-resistant organisms in a pediatric unit. American Journal of Infection Control 2014; 42(1): 88-9.
126. Tennant I, Harding H, Nelson M, Roye-Green K. Microbial isolates from patients in an intensive care unit, and associated risk factors. 2005; 1(4): 225-31.
127. Tfifha M, Ferjani A, Mallouli M, Mlika N, Abroug S, Boukadida J. Carriage of multidrug-resistant bacteria among pediatric patients before and during their hospitalization in a tertiary pediatric unit in Tunisia. Libyan j 2018; 13(1): 1419047.
128. Toussaint-Martínez de Castro G, Kaufer-Horwitz M, Carrillo-López HA, Klünder-Klünder M, Jarillo-Quijada A, García-Hernández HR. Nutritional status of children in critical condition at
admission to pediatric intensive care units. Boletin Medico del Hospital Infantil de Mexico 2013; 70(3): 216-21.
129. Ttendo SS, Was A, Preston MA, Munyarugero E, Kerry VB, Firth PG. Retrospective Descriptive Study of an Intensive Care Unit at a Ugandan Regional Referral Hospital. World J Surg 2016; 40(12): 2847-56.
130. Twomey M, Cheema B, Buys H, et al. Vital signs for children at triage: a multicentre validation of the revised South African Triage Scale (SATS) for children. Samj, S 2013; 103(5): 304-8.
131. Veirum JE, Biai S, Jakobsen M, et al. Persisting high hospital and community childhood mortality in an urban setting in Guinea-Bissau. Acta Paediatr 2007; 96(10): 1526-30.
132. Velazquez Cardona C, Rajah C, Mzoneli YN, et al. An audit of paediatric pain prevalence, intensity, and treatment at a South African tertiary hospital. Pain rep 2019; 4(6): e789.
133. Wunderlich J, Acuna-Soto R, Alonso WJ. Dengue hospitalisations in Brazil: annual wave from West to East and recent increase among children. Epidemiol Infect 2018; 146(2): 236-45.
134. Xu R, Zhao Q, Coelho MSZS, et al. Socioeconomic level and associations between heat exposure and all-cause and cause-specific hospitalization in 1,814 Brazilian cities: A nationwide case-crossover study. PLoS Medicine 2020; 17(10).

Ineligible population (n=298)

1. Ababulgu RZ, Tesfaye BT. Characteristics and Outcomes of Children with Type-I Diabetes Mellitus Hospitalized for Ketoacidosis. Curr Diabetes Rev 2019; 06: 06.
2. Abhilash KPP, Vincent D, George AS, Kalyaniwala K, Prajapathi A, Thomas SM. Pattern and outcome of unintentional pediatric trauma in the emergency department of a tertiary care hospital in South India. Journal of Medical Sciences (Taiwan) 2018; 38(6): 258-68.
3. ACCESS-SMC Partnership. Effectiveness of seasonal malaria chemoprevention at scale in west and central Africa: an observational study. Lancet 2020; 396 North American Edition(10265): 1829-40.
4. Accorsi S, Fabiani M, Nattabi B, et al. Differences in hospital admissions for males and females in northern Uganda in the period 1992-2004: a consideration of gender and sex differences in health care use. Trans R Soc Trop Med Hyg 2007; 101(9): 929-38.
5. Accorsi S, Kedir N, Farese P, et al. Poverty, inequality and health: the challenge of the double burden of disease in a non-profit hospital in rural Ethiopia. Trans R Soc Trop Med Hyg 2009; 103(5): 461-8.
6. Adam A. Gender Differentials and Disease-Specific Cause of Infant Mortality: A Case Study in an Urban Hospital in Accra, Ghana. Afr J Reprod Health 2016; 20(2): 104-10.
7. Adam S, Sama DH, Mouzou T, Akakpovi A, Tomta K, Bellier M. Emergency admissions in sub Saharan Africa: example of the surgical emergency admissions unit at the Sylvanus Olympio Teaching Hospital of Lome, Togo. Med 2018; 28(3): 327-30.
8. Adegoke SA, Oginni LM. Predictors of paediatric injury mortality. SAJCH South African Journal of Child Health 2011; 5(1): 15-8.
9. Adekunle O, Olatunde IO, Abdullateef RM. Causes and pattern of death in a tertiary health institution in South Western Nigeria. Niger Postgrad Med J 2008; 15(4): 247-50.
10. Adeniyi S, Awosan K. Pattern of noma (cancrum oris) and its risk factors in Northwestern Nigeria: A hospital-based retrospective study. Annals of African Medicine 2019; 18(1): 17-22.
11. Adeolu AA, Abiona TC, Komolafe EO, Adeolu JO, Adegbehingbe OO. Epidemiology of neurotrauma in Ife-Ijesha zone of Nigeria. World Neurosurg 2013; 80(3-4): 251-4.

12. Adeolu AA, Arowolo OA, Alatise OI, et al. Pattern of death in a Nigerian teaching hospital; 3-decade analysis. Afr Health Sci 2010; 10(3): 266-72.
13. Adeyeye EI, Iyoha AA, Ogunlade I. Analysis of medical admissions to Ekiti State specialist hospital, Ado-Ekiti, Nigeria, 1999 - 2001. Biosciences Biotechnology Research Asia 2006; 3(1 A): 63-72.
14. Adoga AA, Kokong DD, Mugu JG, Okwori ET, Yaro JP. Epistaxis: The demographics, etiology, management, and predictors of outcome in Jos, North-Central Nigeria. Ann Afr Med 2019; 18(2): 75-9.
15. Agossou-Voyeme AK, Fiogbe MA, Goundete J, Hounnou GM, Hodonou R. Paediatric urologic pathologies at the national teaching hospital in Cotonou: A etiological and therapeutic aspects. Afr J Paediatr Surg 2013; 10(2): 135-9.
16. Agrawal D, Ahmed S, Khan S, Gupta D, Sinha S, Satyarthee GD. Outcome in 2068 patients of head injury: Experience at a level 1 trauma centre in India. Asian J Neurosurg 2016; 11(2): 143-5.
17. Ahmed MAA, Al-Nafeesah A, Al-Wutayd O, Mahgoub HM, Adam I. Severe childhood anemia and emergency blood transfusion in Gadarif Hospital, eastern Sudan. PLoS ONE 2019; 14(12): e0225731.
18. Akech S, Ayieko P, Gathara D, et al. Risk factors for mortality and effect of correct fluid prescription in children with diarrhoea and dehydration without severe acute malnutrition admitted to Kenyan hospitals: an observational, association study. The Lancet Child and Adolescent Health 2018; 2(7): 516-24.
19. Akech S, Rotich B, Chepkirui M, Ayieko P, Irimu G, English M. The prevalence and management of dehydration amongst neonatal admissions to general paediatric wards in Kenya-a clinical audit. Journal of Tropical Pediatrics 2018; 64(6): 516-22.
20. Akhiwu WO, Nwafor CC, Igbe AP. A 20 year retrospective analysis of medicolegal deaths in a tertiary hospital setting in Nigeria. Niger J Clin Pract 2013; 16(4): 535-9.
21. Akhuemokhan OC, Ewah-Odiase RO, Akpede N, et al. Prevalence of Lassa Virus Disease (LVD) in Nigerian children with fever or fever and convulsions in an endemic area. PLoS Negl Trop Dis 2017; 11(7): e0005711.
22. Akinbami FO, Venugopalan P, Elnour IB, Nirmala V, Abiodun P, Azubuike JC. Pattern of chronic diarrhoea in children: a prospective analysis of causes, clinical features and outcome. Niger Postgrad Med J 2006; 13(1): 53-6.
23. Al-Okour K. Growth pattern of urinary pathogens in children with febrile UTI. Rawal Medical Journal 2014; 39(3): 319-22.
24. Alam A, Gupta S. Lactate Measurements and Their Association With Mortality in Pediatric Severe Sepsis in India: Evidence That 6-Hour Level Performs Best. J Intensive Care Med 2020: 885066620903231.
25. Alam MR, Haque M. An appraisal of mortality in intensive care unit of a level III Military Hospital of Bangladesh. Indian Journal of Critical Care Medicine 2017; 21(9): 594-8.
26. Alemu MH. Patterns of pre hospital fatal injuries in Mekelle, Ethiopia. Ethiop Med J 2008; 46(2): 179-83.
27. Ali M, Naureen H, Tariq MH, et al. Rational use of antibiotics in an intensive care unit: A retrospective study of the impact on clinical outcomes and mortality rate. Infection and Drug Resistance 2019; 12: 493-9.
28. Alonso-Canovas A, de Felipe-Mimbrera A, Gonzalez-Valcarcel J, Garcia-Barragan N, Corral I, Masjuan J. Neurology at the airport. J Neurol Neurosurg Psychiatry 2011; 82(9): 981-5.
29. Alvi AR. Impact of rural medical care in remote mountainous region of pakistan: Challenges and opportunities. Pakistan Journal of Medical Sciences 2009; 25(1): 131-6.
30. Alvi R. Experience of developing rural surgical care in a remote mountainous region of Pakistan: Challenges and opportunities. Annals of Tropical Medicine and Public Health 2011; 4(1): 57-63.
31. Amboko BI, Ayieko P, Ogero M, Julius T, Irimu G, English M. Malaria investigation and treatment of children admitted to county hospitals in western Kenya. Malaria Journal 2016; 15(1).
32. Anders KL, Nguyen HL, Nguyen NM, et al. Epidemiology and virology of acute respiratory infections during the first year of life: A birth cohort study in Vietnam. Pediatric Infectious Disease Journal 2015; 34(4): 361-70.
33. Anders KL, Thompson CN, Thuy NT, et al. The epidemiology and aetiology of diarrhoeal disease in infancy in southern Vietnam: a birth cohort study. Int J Infect Dis 2015; 35: 3-10.
34. Anderson JE, Erickson A, Funzamo C, et al. Surgical conditions account for the majority of admissions to three primary referral hospitals in rural Mozambique. World J Surg 2014; 38(4): 823-9.
35. Andrade SS, Jorge MH. Estimate of physical sequelae in victims of road traffic accidents hospitalized in the Public Health System. Rev 2016; 19(1): 100-11.
36. Andrade SS, Jorge MH. Hospitalization due to road traffic injuries in Brazil, 2013: hospital stay and costs. Epidemiol 2017; 26(1): 31-8.
37. Anochie IC, Eke FU. Acute renal failure in Nigerian children: Port Harcourt experience. Pediatr Nephrol 2005; 20(11): 1610-4.
38. Ansari I, Pokhrel Y. Culture proven bacterial meningitis in children: agents, clinical profile and outcome. Kathmandu Univ 2011; 9(33): 36-40.
39. Aregawi M, Lynch M, Bekele W, et al. Time series analysis of trends in malaria cases and deaths at hospitals and the effect of antimalarial interventions, 2001-2011, Ethiopia. PLoS ONE 2014; 9(11): e106359.
40. Ariyaratnam R, Palmqvist CL, Hider P, et al. Toward a standard approach to measurement and reporting of perioperative mortality rate as a global indicator for surgery. Surgery 2015; 158(1): 17-26.

41. Arul GS, Reynolds J, DiRusso S, et al. Paediatric admissions to the British military hospital at Camp Bastion, Afghanistan. 2012; 1(1): 52-7.
42. Asati DP, Sharma VK, Khandpur S, Khilnani GC, Kapil A. Clinical and bacteriological profile and outcome of sepsis in dermatology ward in tertiary care center in New Delhi. Indian J Dermatol Venereol Leprol 2011; 77(2): 141-7.
43. Assao Neino MM, Gagara Issoufou MA, Ouédraogo AR, Marcellin KA, Maizoumbou DA, Mamadou S. The current situation with sputum positive pulmonary tuberculosis in Niamey (Niger). Revue des Maladies Respiratoires 2019; 36(5): 578-82.
44. Asumanu E, Richardson R. Improving emergency attendance and mortality--the case for unit separation. West Afr J Med 2009; 28(1): 28-32.
45. Athwani V, Bhargava M, Chanchlani R, Mehta A, Mehta AJ. Incidence and Outcome of Acute Cardiorenal Syndrome in Hospitalized Children. Indian Journal of Pediatrics 2017; 84(6): 420-4.
46. Atiq H, Siddiqui E, Bano S, et al. The pediatric disease spectrum in emergency departments across Pakistan: data from a pilot surveillance system. BMC emerg 2015; 15 Suppl 2: S11.
47. Aurenty L, López D, Méndez O, et al. Osteomielitis aguda y crónica: 10 años de estudio en pacientes pediítricos: Hospital de Niños J.M. de Los Rí-os Caracas Venezuela. Bol venez infectol 2010; 21(1): 34-40.
48. Ayoola OO, Orimadegun AE, Akinsola AK, Osinusi K. A five-year review of childhood mortality at the University College Hospital, Ibadan. West Afr J Med 2005; 24(2): 175-9.
49. Ba DM, Mboup MC, Zeba N, et al. Infective endocarditis in Principal Hospital of Dakar: a retrospective study of 42 cases over 10 years. Pan Afr Med J 2017; 26: 40.
50. Bahloul M, Chelly H, Chaari A, et al. Isolated traumatic head injury in children: Analysis of 276 observations. J 2011; 4(1): 29-36.
51. Banerjee R, Prasad A, Gupta S. Clinical spectrum of acute abdomen in children admitted to pediatric emergency department: A prospective study. Current Medicine Research and Practice 2019; 9(2): 49-52.
52. Basu K, Mondal RK, Banerjee DP. Epidemiological aspects of acute childhood poisoning among patients attending a hospital at Kolkata. Indian J Public Health 2005; 49(1): 25-6.
53. Bazaraa HM, El Houchi S, Rady HI. Profile of patients visiting the pediatric emergency service in an Egyptian university hospital. Pediatr Emerg Care 2012; 28(2): 148-52.
54. Bejon P, Mohammed S, Mwangi I, et al. Fraction of all hospital admissions and deaths attributable to malnutrition among children in rural Kenya. Am J Clin Nutr 2008; 88(6): 1626-31.
55. Bella ME, Borgiattino V. Demand for hospitalization due to psychosocial causes in a pediatric hospital. Arch Argent Pediatr 2016; 114(3): 252-7.
56. Bellad R, Rao S, Patil VD, Mahantshetti NS. Outcome of intensive care unit patients using pediatric risk of mortality (PRISM) score. Indian Pediatrics 2009; 46(12): 1091-2.
57. Ben Fredj M, Sriha Belguith A, Abroug H, et al. Hospitalizations for communicable diseases in a developing country: prevalence and trends- Monastir, Tunisia, 2002-2013. International Journal of Infectious Diseases 2017; 55: 102-8.
58. Ben Salem K, El Mhamdi S, Letaief M, Bchir M, Soltani MS. Epidemiological profile of health-care-associated infections in the central-east area of Tunisia. East Mediterr Health J 2011; 17(6): 485-9.
59. Berends EA, Erasmus E, van Veenendaal NR, Mukonkole SN, Lahri S, Van Hoving DJ. Assessment of documented adherence to critical actions in paediatric emergency care at a district-level public hospital in South Africa. African Journal of Emergency Medicine 2021; 11(1): 98-104.
60. Berkeley J, Mwangi I, Griffiths K, et al. Assessment of severe malnutrition among hospitalized children in rural Kenya: comparison of weight for height and mid upper arm circumference. JAMA: Journal of the American Medical Association 2005; 294(5): 591-7.
61. Bhagavath P, Monteiro FNP, Gnanadev N. Epidemiology of Intentional Self Poisoning. Medico-Legal Update 2012; 12(1): 52-4.
62. Bhardwaj R, Rai SK, Yadav AK, et al. Epidemiology of Congenital Heart Disease in India. Congenit 2015; 10(5): 437-46.
63. Biai S, Rodrigues A, Nielsen J, Sodemann M, Aaby P. Vaccination status and sequence of vaccinations as risk factors for hospitalisation among outpatients in a high mortality country. Vaccine 2011; 29(20): 3662-9.
64. Borgman M, Matos RI, Blackbourne LH, Spinella PC. Ten years of military pediatric care in Afghanistan and Iraq. J Trauma Acute Care Surg 2012; 73(6 Suppl 5): S509-13.
65. Brent AJ, Ahmed I, Ndiritu M, et al. Incidence of clinically significant bacteraemia in children who present to hospital in Kenya: community-based observational study. Lancet 2006; 367 North American Edition(9509): 482-8.
66. Bundu I, Lowsby R, Vandy HP, et al. The burden of trauma presenting to the government referral hospital in Freetown, Sierra Leone: An observational study. Afr J Emerg Med 2019; 9(Suppl): S9-S13.
67. Burza S, Mahajan R, Marino E, et al. Community-based management of severe acute malnutrition in India: new evidence from Bihar. Am J Clin Nutr 2015; 101(4): 847-59.
68. Caballero A, Pinilla MI, Mendoza ICSr, Peña JRA. Frecuencia de reingresos hospitalarios y factores asociados en afiliados a una administradora de servicios de salud en Colombia. Cad saúde pública 2016; 32(7): e00146014-e.

69. Caldart RV, Marrero L, Basta PC, Orellana JD. Factors associated with pneumonia in Yanomami children hospitalized for Ambulatory Care sensitive conditions in the north of Brazil. Cienc 2016; 21(5): 1597-606.
70. Cardenas-Turanzas M, Tovalin-Ahumada H, Romo CG, Okhuysen-Cawley R. Assessing need for palliative care services for children in Mexico. J Palliat Med 2015; 18(2): 162-6.
71. Cardoso MP, Bourguignon DC, Gomes MM, Saldiva PH, Pereira CR, Troster EJ. Comparison between clinical diagnoses and autopsy findings in a pediatric intensive care unit in Sao Paulo, Brazil. Pediatr Crit Care Med 2006; 7(5): 423-7.
72. Cartmell E, Natalal H, Francois I, Ferreira MH, Grahnquist L. Nutritional and clinical status of children admitted to the malnutrition ward, Maputo central hospital: a comparison of data from 2001 and 1983. J Trop Pediatr 2005; 51(2): 102-5.
73. Carugati M, Zhang HL, Kilonzo KG, et al. Predicting Mortality for Adolescent and Adult Patients with Fever in Resource-Limited Settings. Am J Trop Med Hyg 2018; 99(5): 1246-54.
74. Carvalho PR, Feldens L, Seitz EE, Rocha TS, Soledade MA, Trotta EA. Prevalence of systemic inflammatory syndromes at a tertiary pediatric intensive care unit. J Pediatr (Rio J) 2005; 81(2): 143-8.
75. Carville KS, Lehmann D, Hall G, et al. Infection is the major component of the disease burden in aboriginal and non-aboriginal Australian children: a population-based study. 2007; 1(3): 210-6.
76. Chaio Sn, Toibaro J, Valicenti P, Saidón P. Reacciones adversas medicamentosas y errores de prescripción: morbi-mortalidad. Medicina (BAires) 2013; 73(2): 111-8.
77. Chamberlain S, Stolz U, Dreifuss B, et al. Mortality related to acute illness and injury in rural Uganda: task shifting to improve outcomes. PLoS ONE 2015; 10(4): e0122559.
78. Chukuezi AB, Nwosu JN. Mortality pattern in the surgical wards: a five year review at Federal Medical Centre, Owerri, Nigeria. Int J Surg 2010; 8(5): 381-3.
79. Cluet de Rodrí-guez I, Rossell-Pineda MadR, Ãlvarez de Acosta T, Chirinos R. Comparación entre los niveles de prolactina de madres en relactancia y madres con lactancia materna exclusiva. Arch latinoam nutr 2014; 64(1): 1-8.
80. Contreras Camarena C. Utilización hospitalaria de las especialidades médicas. Acta méd peru 2011; 28(3): 124-31.
81. Contreras-Camarena C. Factores asociados a hospitalizaciones inadecuadas a los servicios de medicina. Rev Soc Peru Med Interna 2011; 24(4): 177-85.
82. Crouse HL, Torres F, Vaides H, et al. Impact of an Emergency Triage Assessment and Treatment (ETAT)-based triage process in the paediatric emergency department of a Guatemalan public hospital. Paediatr Int Child Health 2016; 36(3): 219-24.
83. Da Silva ARA, Oliveira CVDS, Teixeira CH, Leal IA. Use of the World Health Organization Access, Watch, and Reserve Classification to Follow Trends in Prescription of Antibiotic Use in Two Pediatric Intensive Care Units in Rio de Janeiro, Brazil. Journal of Pediatric Infectious Diseases 2020; 15(3): 155-60.
84. da Silva I, de Almeida DS, Hashimoto EM, Martins LD. Risk assessment of temperature and air pollutants on hospitalizations for mental and behavioral disorders in Curitiba, Brazil. Environ Health 2020; 19(1): 79.
85. Davies NE, Karstaedt AS. Shigella bacteraemia over a decade in Soweto, South Africa. Trans R Soc Trop Med Hyg 2008; 102(12): 1269-73.
86. de Almeida CE, de Sousa Filho JL, Dourado JC, Gontijo PA, Dellaretti MA, Costa BS. Traumatic Brain Injury Epidemiology in Brazil. World Neurosurg 2016; 87: 540-7.
87. de Lima Mota M, De Melo D, Christyan Nunes Beserra F, et al. Clinical-epidemiological profile and factors related to the mortality of patients with nontuberculous mycobacteria isolated at a reference hospital in Cearí, Northeastern Brazil. International Journal of Mycobacteriology 2020; 9(1): 83-90.
88. Debnath DJ, Parulekar CV. Profile of under-five malnourished children admitted in a tertiary care teaching hospital in pune, India. Int J Prev Med 2014; 5(7): 882-6.
89. den Hollander D, Albert M, Strand A, Hardcastle TC. Epidemiology and referral patterns of burns admitted to the Burns Centre at Inkosi Albert Luthuli Central Hospital, Durban. Burns 2014; 40(6): 1201-8.
90. Deorari AK, Chellani H, Carlin JB, et al. Clinicoepidemiological profile and predictors of severe illness in young infants (< 60 days) reporting to a hospital in North India. Indian Pediatr 2007; 44(10): 739-48.
91. Dhaffala A, Longo-Mbenza B, Kingu JH, et al. Demographic profile and epidemiology of injury in Mthatha, South Africa. African Health Sciences 2013; 13(4): 1144-8.
92. Dhillon MS, Kumar D, Saini UC, Bhayana H, Gopinathan NR, Aggarwal S. Changing Pattern of Orthopaedic Trauma Admissions During COVID-19 Pandemic: Experience at a Tertiary Trauma Centre in India. Indian j 2020: 1-6.
93. Edaigbini SA, Eze JC, Anyanwu CH. Audit of cardiothoracic diseases seen at the National Cardiothoracic Centre Enugu Nigeria. Niger J Med 2011; 20(3): 376-9.
94. Einterz EM, Bates M. Causes and circumstances of death in a district hospital in northern Cameroon, 1993-2009. Rural Remote Health 2011; 11(3): 1623.
95. El Kassas M, Asem N, Abdelazeem A, et al. Clinical features and laboratory characteristics of patients hospitalized with COVID-19: single centre report from Egypt. J 2020; 14(12): 1352-60.
96. El Kholy AA, Mostafa NA, El-Sherbini SA, et al. Morbidity and outcome of severe respiratory syncytial virus infection. Pediatr Int 2013; 55(3): 283-8.

97. Elisante M, Geoffrey M, Noemi S, et al. Diagnoses made in an Emergency Department in rural sub-Saharan Africa. Swiss Medical Weekly 2019; 149(5-6).
98. Embu HY, Yiltok S, Isamade E, Nuhu S, Oyeniran O, Uba F. Paediatric admissions and outcome in a general intensive care unit. African Journal of Paediatric Surgery 2011; 8(1): 57-61.
99. Emodi IJ, Ikefuna AN, Obichukwu C. Incidence and outcome of neonatal tetanus in Enugu over a 10-year period. SAJCH South African Journal of Child Health 2011; 5(4): 117-9.
100. Emodi I, Ikefuna A, Ujunwa F, Chinawa J. Synopsis of non-communicable diseases in children admitted to the paediatric ward of the university of Nigeria teaching hospital (UNTH) Enugu, Nigeria: A ten year review. Niger Med J 2014; 55(6): 499-503.
101. Erhabor GE, Adewole OO, Ogunlade O. A five-year review of tuberculosis mortality amongst hospitalised patients in Ile-Ife. Indian J Chest Dis Allied Sci 2006; 48(4): 253-6.
102. Escandón-Vargas K, Tangua AR, Medina P, et al. Healthcare-associated infections in burn patients: Timeline and risk factors. Burns 2020; 46(8): 1775-86.
103. Ezeonwu B, Chima O, Oguonu T, Ikefuna A, Nwafor I. Morbidity and mortality pattern of childhood illnesses seen at the children emergency unit of federal medical center, asaba, Nigeria. ann 2014; 4(Suppl 3): S239-44.
104. Fedrizzi V, El Kik S, Testoni F, Nieto M. Anílisis retrospectivo de pacientes con traqueostomí-a nueva en 2010-2011. Med infant 2016; 23(4): 299-302.
105. Feikin DR, Adazu K, Obor D, et al. Mortality and health among internally displaced persons in western Kenya following post-election violence, 2008: novel use of demographic surveillance. Bull World Health Organ 2010; 88(8): 601-8.
106. Fengjun L, Mteta KA, Xuting Z, Nanhai S. Complex therapy for hepatic trauma. East African medical journal 2005; 82(1): 28-33.
107. Ferrer AP, Grisi SJ. Assessment of access to primary health care among children and adolescents hospitalized due to avoidable conditions. Rev Assoc Med Bras 2016; 62(6): 513-23.
108. Fetuga MB, Jokanma OF, Ogunfowora OB, Abiodun R. A ten-year study of measles admissions in a Nigerian teaching hospital. Niger J Clin Pract 2007; 10(1): 41-6.
109. Frank-Briggs AI, Nte AR. Trends in diarrhoeal disease in a southern Nigerian coastal city. West Afr J Med 2009; 28(4): 211-5.
110. Freitas JS, Chaves MMN, Raksa VP, Larocca LM. Internações de adolescentes por condições sensíveis à atenção primária em uma regional de saúde. Cogitare enferm 2018; 23(4): e56188-e.
111. Gaitín-Duarte H, Eslava-Schmalbach J, Rodrí-guez-Malagon N, Forero-Supelano V, Santofimio-Sierra D, Altahona H. Incidencia y Evitabilidad de Eventos Adversos en Pacientes Hospitalizados en tres Instituciones Hospitalarias en Colombia, 2006. Rev salud pública 2008; 10(2): 215-26.
112. Ghafoor T, Khalil S, Farah T, Ahmed S, Sharif I. Prognostic Factors in Childhood Acute Myeloid Leukemia; Experience from A Developing Country. Cancer Rep (Hoboken) 2020; 3(5): e1259.
113. Ghimire U, Aryal BK, Gupta AK, Sapkota S. Severe acute malnutrition and its associated factors among children under-five years: a facility-based cross-sectional study. BMC Pediatr 2020; 20(1): 249.
114. Giri S, Agarwal MP, Garg S, Aggarwal S, Ranga R, Sharma V. A single centre retrospective study to analyze and compare mortality amongst geriatric and non-geriatric population presenting to a tertiary care hospital in Delhi. Annals of Tropical Medicine and Public Health 2012; 5(5): 495-7.
115. Giri S, Halvas-Svendsen T, Rogne T, et al. Pediatric Patients in a Local Nepali Emergency Department: Presenting Complaints, Triage and Post-Discharge Mortality. Glob 2020; 7: 2333794X20947926.
116. Gladstone BP, Muliyil JP, Jaffar S, et al. Infant morbidity in an Indian slum birth cohort. Arch Dis Child 2008; 93(6): 479-84.
117. Goncalez TT, Sabino EC, Capuani L, et al. Blood transfusion utilization and recipient survival at Hospital das Clinicas in Sao Paulo, Brazil. 2012; 1(4): 729-38.
118. Grandin W, Westwood T, Lagerdien K, King MS. Deaths at Red Cross Children's Hospital, Cape Town 1999-2003--a study of death notification forms. Samj, S 2006; 96(9 Pt 2): 964-8.
119. Groome MJ, Albrich WC, Wadula J, Khoosal M, Madhi SA. Community-onset Staphylococcus aureus bacteraemia in hospitalised African children: high incidence in HIV-infected children and high prevalence of multidrug resistance. Paediatr Int Child Health 2012; 32(3): 140-6.
120. Guanche Garcell H, Martínez Quesada C, Peña Sandoval R, Gutiérrez Garcí-a F, Gonzílez López J, Sínchez Villalobo J. Hospitalizaciones por accidentes de trífico en Villa Clara (enero a junio de 2003). Rev cuba cir 2008; 47(3).
121. Guimarães, N. M.; de Oliveira, E. R.; Bohland, A. K. Revista Paulista de Pediatria 2020;38(): 2020
122. Gulati S, Kalra V, Sridhar MR. Status epilepticus in Indian children in a tertiary care center. 2005; 1(2): 105-8.
123. Hamid MH, Butt T, Baloch GR, Maqbool S. Acute poisoning in children. J Coll Physicians Surg Pak 2005; 15(12): 805-8.
124. Hanieh S, Ha TT, Simpson JA, et al. Exclusive breast feeding in early infancy reduces the risk of inpatient admission for diarrhea and suspected pneumonia in rural Vietnam: a prospective cohort study. BMC Public Health 2015; 15(1): 1-10.

125. Hapolo E, Ilai J, Francis T, du Cros P, Taune M, Chan G. TB treatment delay associated with drug resistance and admission at Daru General Hospital in Papua New Guinea. Public Health Action 2019; 9: S50-S6.
126. Harris JB, Faiz MA, Rahman MR, et al. Snake bite in Chittagong Division, Bangladesh: a study of bitten patients who developed no signs of systemic envenoming. Trans R Soc Trop Med Hyg 2010; 104(5): 320-7.
127. Hassan AA, Taha Z, Abdulla MA, Ali AA, Adam I. Causes and risk factors of hospitalization among under-five children in kassala, eastern sudan. Open Access Macedonian Journal of Medical Sciences 2020; 8(E): 451-7.
128. Hawkridge A, Hatherill M, Little F, et al. Efficacy of percutaneous versus intradermal BCG in the prevention of tuberculosis in South African infants: Randomised trial. BMJ 2008; 337(7681): 1275-8.
129. Hendrikse C, Parak M, Van Hoving DJ. A descriptive analysis of the effect of the national COVID-19 lockdown on the workload and case mix of patients presenting to a district-level emergency centre in Cape Town, South Africa. S Afr Med J 2020; 110(11): 1113-8.
130. Hodkinson PW, Wallis LA. Cross-sectional survey of patients presenting to a South African urban emergency centre. Emerg Med J 2009; 26(9): 635-40.
131. Hortiwakul T, Nagij S, Chusri S, Silpapojakul K. Nosocomial bloodstream infection in Songklanagarind Hospital: outcome and factors influencing prognosis. J Med Assoc Thai 2012; 95(2): 170-4.
132. House DR, Nyabera SL, Yusi K, Rusyniak DE. Descriptive study of an emergency centre in Western Kenya: Challenges and opportunities. African Journal of Emergency Medicine 2014; 4(1): 19-24.
133. Ibekwe RC, Muoneke VU, Nnebe-Agumadu UH, Amadife MA. Factors influencincing discharge against medical advice among paediatric patients in Abakaliki, Southeastern Nigeria. J Trop Pediatr 2009; 55(1): 39-41.
134. Idro R, Ndiritu M, Ogutu B, et al. Burden, features, and outcome of neurological involvement in acute falciparum malaria in Kenyan children. Jama 2007; 297(20): 2232-40.
135. Ilechie AA, Essuman VA, Enyionam S. Prevalence of congenital eye anomalies in a paediatric clinic in Ghana. Eastern Mediterranean Health Journal 2013; 19(SUPPL 3): S76-S80.
136. Ilori IU, Kalu QN. Intensive care admissions and outcome at the University of Calabar Teaching Hospital, Nigeria. J Crit Care 2012; 27(1): 105.e1-4.
137. Imsuwan I. Characteristics of unscheduled emergency department return visit patients within 48 hours in Thammasat University Hospital. J Med Assoc Thai 2011; 94 Suppl 7: S73-80.
138. Isamade ES, Yiltok SJ, Uba AF, Isamade EI, Daru PH. Intensive care unit admissions in the Jos University Teaching Hospital. Niger J Clin Pract 2007; 10(2): 156-61.
139. Islam MN, Siddika M, Hossain MA, Bhuiyan MK, Ali MA. Morbidity pattern and mortality of neonates admitted in a tertiary level teaching hospital in Bangladesh. Mymensingh Med J 2010; 19(2): 159-62.
140. Islam MR, Nuzhat S, Fahim SM, et al. Antibiotic exposure among young infants suffering from diarrhoea in Bangladesh. J Paediatr Child Health 2020; 27: 27.
141. Jayarajah U, Dissanayake U, Abeysuriya V, et al. Comparing the 2009 and 1997 world health organization dengue case classifications in a large cohort of South Asian patients. Journal of Infection in Developing Countries 2020; 14(7): 781-7.
142. Jayaraman S, Ozgediz D, Miyamoto J, et al. Disparities in injury mortality between Uganda and the United States: comparative analysis of a neglected disease. World J Surg 2011; 35(3): 505-11.
143. Jayasekera P, Dassanayake G, Bandara K, Jayawardhena L, Malkanthi KMS. A Study of the Pattern of Admissions to the Accident and Emergency (A&E) Department of a Tertiary Care Hospital in Sri Lanka. emerg 2020; 2020: 6327293.
144. Ji YJ, Duan XZ, Gao XD, et al. Clinical presentations and outcomes of patients with Ebola virus disease in Freetown, Sierra Leone. Infect 2016; 5(1): 101.
145. Jiménez-Ãlvarez A, Acosta-Gutiérrez P, León-Govea MA, et al. Frecuencia de antibioticoterapia en pacientes hospitalizados y factores de riesgo asociados. Rev salud pública 2009; 11(2): 247-55.
146. Jofiro G, Jemal K, Beza L, Bacha Heye T. Prevalence and associated factors of pediatric emergency mortality at Tikur Anbessa specialized tertiary hospital: A 5 year retrospective case review study 11 Medical and Health Sciences 1117 Public Health and Health Services. BMC Pediatrics 2018; 18(1).
147. John Kirubakaran J, Jena SK, Basaveswara Rao MV, Dhanaraju MD. A study on pattern and outcome of poisoning cases in a tertiary care hospital in andhra pradesh, India. Indian Journal of Public Health Research and Development 2019; 10(8): 575-80.
148. Junge S, Palmer A, Greenwood BM, Kim Mulholland E, Weber MW. The spectrum of hypoxaemia in children admitted to hospital in The Gambia, West Africa. Trop Med Int Health 2006; 11(3): 367-72.
149. Kadhim TM. Iatrogenic pulmonary edema as a cause of death in burns. International Journal of Research in Pharmaceutical Sciences 2020; 11(1): 635-8.
150. Kanan SO, Swar MO. Prevalence and outcome of severe malnutrition in children less than five-year-old in Omdurman Paediatric Hospital, Sudan. Sudan 2016; 16(1): 23-30.
151. Kanchan T, Menezes RG. Mortalities among children and adolescents in manipal, southern India. J Trauma 2008; 64(6): 1600-7.

152. Kanopa V, Cuadro MaN, de León A, De León L, Milovidov A, Giachetto G. Hospitalizaciones por ALTE en el Centro Hospitalario Pereira Rossell: (1/12/2010 - 1/12/2011). Rev méd Urug 2014; 30(4): 218-25.
153. Karachentsev S. Epidemiology and management of peritonitis at a rural hospital in Zambia. Annals of African Surgery 2020; 17(3): 120-5.
154. Kendig CE, Samuel JC, Varela C, et al. Pediatric surgical care in Lilongwe, Malawi: outcomes and opportunities for improvement. J Trop Pediatr 2014; 60(5): 352-7.
155. Kerber R, Krumkamp R, B DI, et al. Analysis of Diagnostic Findings from the European Mobile Laboratory in Guéckédou, Guinea, March 2014 Through March 2015. Journal of Infectious Diseases 2016; 214: S250-S7.
156. Kesah FC, Khan PV, Chrysanthus N. A retrospective study on community infections in Dschang, West Region, Cameroon. Canadian Journal of Infection Control 2012; 27(1): 23-8.
157. Khaliq MF, Noorani MM, Siddiqui UA, Al Ibran E, Rao MH. Factors associated with duration of hospitalization and outcome in burns patients: a cross sectional study from Government Tertiary Care Hospital in Karachi, Pakistan. Burns 2013; 39(1): 150-4.
158. Khan N, Malik MA. Presentation of burn injuries and their management outcome. JPMA J Pak Med Assoc 2006; 56(9): 394-7.
159. Khilnani P, Sarma D, Zimmerman J. Epidemiology and peculiarities of pediatric multiple organ dysfunction syndrome in New Delhi, India. Intensive Care Med 2006; 32(11): 1856-62.
160. Khokhar RA, Gowa MA, Bangash SK, Tahir A. The Spectrum of Pediatric Cardiac Procedures and Their Outcomes: A Six-month Report from the Largest Cardiac Facility in Sindh, Pakistan. Cureus 2019; 11(8): e5339.
161. Khoso N, Ghaffar WB, Abassi S, Khan FA. Pediatric Anesthesia Severe Adverse Events Leading to Anesthetic Morbidity and Mortality in a Tertiary Care Center in a Low- A nd Middle-Income Country: A 25-Year Audit. Anesthesia and Analgesia 2020: 217-22.
162. Khoso N, Ghaffar WB, Abassi S, Khan FA. Pediatric Anesthesia Severe Adverse Events Leading to Anesthetic Morbidity and Mortality in a Tertiary Care Center in a Low- and Middle-Income Country: A 25-Year Audit. Anesth Analg 2021; 132(1): 217-22.
163. Khuri-Bulos N, Lawrence L, Piya B, et al. Severe outcomes associated with respiratory viruses in newborns and infants: a prospective viral surveillance study in Jordan. BMJ Open 2018; 8(5): e021898.
164. Kortz TB, Sawe HR, Murray B, Enanoria W, Matthay MA, Reynolds T. Clinical Presentation and Outcomes among Children with Sepsis Presenting to a Public Tertiary Hospital in Tanzania. Front 2017; 5: 278.
165. Kubale J, Kuan G, Gresh L, et al. Assessing the Incidence of Symptomatic Respiratory Syncytial Virus Illness Within a Prospective Birth Cohort in Managua, Nicaragua. Clinical Infectious Diseases 2020; 79(10): 2029-35.
166. Kumar D, Pannu AK, Dhibar DP, Singh R, Kumari S. The epidemiology and clinical spectrum of infections of the central nervous system in adults in north India. Tropical Doctor 2021; 51(1): 48-57.
167. Kumar S, Ghouri A, Shaikh M, Shah I, Kumar D, Awais M. Realty or myth hyponatremia is common in admitted patients in medical wards LUMHS Hyderabad. Journal of the Liaquat University of Medical and Health Sciences 2014; 13(2): 83-6.
168. Kwizera A, Dunser M, Nakibuuka J. National intensive care unit bed capacity and ICU patient characteristics in a low income country. BMC Res Notes 2012; 5: 475.
169. Larru B, Molyneux E, Ter Kuile FO, Taylor T, Molyneux M, Terlouw DJ. Malaria in infants below six months of age: retrospective surveillance of hospital admission records in Blantyre, Malawi. Malar J 2009; 8: 310.
170. Levy SB, Zimmermann O, de Ciman R, et al. Bacteremia among Kenyan children...Berkley JA, Lowe BS, Mwangi I et al. Bacteremia among children admitted to a rural hospital in Kenya. N Engl J Med 2005;352:39-47. New England Journal of Medicine 2005; 352(13): 1379-81.
171. Lima ACMG, Nichiata LYI, Bonfim D. Emergency department visits for ambulatory care sensitive conditions. Rev Esc Enferm USP 2019; 53: e03414-e.
172. Liñán Ponce JY, Véliz Vilcapoma F. Características clínicas de los pacientes con sepsis severa admitidos a una Unidad de Cuidados Intensivos. Rev Soc Peru Med Interna 2008; 21(4): 139-42.
173. Lohanathan A, Hazra D, Jyothirmayi CA, Kundavaram AP. An elucidation of pattern of injuries in patients with fall from height. Indian Journal of Critical Care Medicine 2020; 24(8): 683-7.
174. Lopes M, de Aguiar Junior W, Whitaker IY. The association between burn and trauma severity and in-hospital complications. Burns 2020; 46(1): 83-9.
175. López Torres O, Lima Castro J, Berdalles Milián JD, García Montes de Oca R. Ahogamiento incompleto, valoración de la injuria cerebral al ingreso: terapia intensiva pediátrica. Rev medica electron 2009; 31(3).
176. Louangpradith, V.; Phoummalaysith, B.; Kariya, T.; Saw, Y. M.; Yamamoto, E.; Hamajima, N. Nagoya J Med Sci 2020;82(1):113-121 2020
177. Lupisan SP, Ruutu P, Erma Abucejo-Ladesma P, et al. Predictors of death from severe pneumonia among children 2-59 months old hospitalized in Bohol, Philippines: implications for referral criteria at a first-level health facility. Trop Med Int Health 2007; 12(8): 962-71.

178. M'Pemba Loufoua Lemay AB, Mabiala Babela JR, Bantsimba T, Nzingoula S. [Tuberculosis and HIV/AIDS co-infection in children: experience carried out in paediatric service of the teaching hospital of Brazzaville, Republic of Congo (1995-2004)]. Bull Soc Pathol Exot 2007; 100(1): 51-2.
179. Mabiala-Babela JR, Pandzou N, Moyen GM. The accidental pathology among infants in pediatric emergencies of the university hospital of Brazzaville (Congo). Journal de Pediatrie et de Puericulture 2010; 23(4): 185-90.
180. Macedo MP, Souza LCD, Corrêa RGCF, Lopes FF. Aspects of dental care for patients hospitalized in a medical clinic from a university hospital. ABCS health sci 2020; 45: [1-5].
181. Makworo D, Laving AM. Acute medical conditions in under five year old children at a public hospital in Kenya. East Afr Med J 2010; 87(8): 340-4.
182. Manongi R, Mtei F, Mtove G, et al. Inpatient child mortality by travel time to hospital in a rural area of Tanzania. Trop Med Int Health 2014; 19(5): 555-62.
183. Mantelo Cecilio HP, Molena-Fernandes CA, de Freitas Mathias TA, Marcon SS. Profile of hospital admissions and deaths from tuberculosis. Acta Paulista de Enfermagem 2013; 26(3): 250-5.
184. Mashreky SR, Rahman A, Chowdhury SM, et al. Burn injury: economic and social impact on a family. Public Health 2008; 122(12): 1418-24.
185. Mashreky SR, Rahman A, Khan TF, Faruque M, Svanström L, Rahman F. Hospital burden of road traffic injury: Major concern in primary and secondary level hospitals in Bangladesh. Public Health 2010; 124(4): 185-9.
186. Massaut J, Valles P, Ghismonde A, et al. The modified south African triage scale system for mortality prediction in resource-constrained emergency surgical centers: a retrospective cohort study. BMC Health Serv Res 2017; 17(1): 594.
187. Matheka DM, Kitonyi MN, Alkizim FO. Three-month pattern of road traffic injuries at a Kenyan level 4 hospital. Pan Afr Med J 2015; 20: 78.
188. Mazzi E, Bartos AE, Carlin J, Weber MW, Darmstadt GL. Clinical signs predicting severe illness in young infants (<60 days) in Bolivia. Journal of Tropical Pediatrics 2010; 56(5): 307-16.
189. Meera M, Rajarao M. Diphtheria in Andhra Pradesh-a clinical-epidemiological study. Int J Infect Dis 2014; 19: 74-8.
190. Mehmood A, He S, Zafar W, Baig N, Sumalani FA, Razzak JA. How vital are the vital signs? A multi-center observational study from emergency departments of Pakistan. BMC Emergency Medicine 2015; 15(2).
191. Mehta C, George JV, Mehta Y, Ali MT, Singh MK. Incidence and risk factors for thrombocytopenia in the intensive care units of a tertiary hospital in northern India. Southern African Journal of Critical Care 2016; 32(1): 28-31.
192. Mekaoui N, Razine R, Bassat Q, Benjelloun BS, Karboubi L. The Effect of COVID-19 on Paediatric Emergencies and Admissions in Morocco: Cannot See the Forest for the Trees? J Trop Pediatr 2020; 03: 03.
193. Mesquita Ramirez MN, Godoy LE, Alvarez Barrientos E. SNAP II and SNAPPE II as Predictors of Neonatal Mortality in a Pediatric Intensive Care Unit: Does Postnatal Age Play a Role? Int J Pediatr 2014; 2014: 298198.
194. Mhando S, Lyamuya S, Lakhoo K. Challenges in developing paediatric surgery in Sub-Saharan Africa. Pediatr Surg Int 2006; 22(5): 425-7.
195. Migaud P, Silverman M, Thistle P. HIV status and mortality of surgical inpatients in rural Zimbabwe: A retrospective chart review. South 2019; 20(1): 812.
196. Mills RP, Clarke DL, Kong VY. Appendicectomy in private practice in kwazulu-natal province, south africa. South African Medical Journal 2018; 108(10): 836-8.
197. Minja IK, Lowery Wilson M, Shaikh MA, Perea-Lowery L. Head and Neck Trauma in a Rapidly Growing African Metropolis: A Two-Year Audit of Hospital Admissions. Int J Environ Res Public Health 2019; 16(24): 05.
198. Miranda JJ, Davies AR, Smith GD, et al. Frequency of diarrhoea as a predictor of elevated blood pressure in children. J Hypertens 2009; 27(2): 259-65.
199. Miranda L, Rodrigues EC, Costa SF, et al. Candida parapsilosis candidaemia in a neonatal unit over 7 years: a case series study. BMJ Open 2012; 2(4).
200. Mishra MN, Kalra R, Rohatgi S. Clinical profile, common thrombophilia markers and risk factors in 85 young Indian patients with arterial thrombosis. Sao Paulo Med J 2013; 131(6): 384-8.
201. Mitangala Ndeba P, Hennart P, D'Alessandro U, et al. Proteoenergetic malnutrition and malaria-related morbidity in children under 59 months in the Kivu region of the Democratic Republic of the Congo. Medecine Tropicale 2008; 68(1): 51-7.
202. Moffatt SE, Kong VY, Weale RD, et al. The spectrum of self inflicted injuries managed at a major trauma centre in South Africa. S Afr J Surg 2019; 57(2): 65.
203. Mohammedahmed ASA, Koko AEA, Arabi AME, Ibrahim MA. Maternal depression, a hidden predictor for severe acute malnutrition in children aged 6-59 months: a case-control study at Omdurman Paediatrics Teaching Hospital, Sudan. Sudan 2020; 20(2): 111-21.
204. Monteiro MdFV, Vertamatti MAF, Alves MNM, Carvalho ACdO, Alves MNT, Barbosa CP. Regulation of access to the hospital care network for children in Cearí, Brazil. International Journal of Health Planning & Management 2018; 33(4): 934-40.

205. Mtove G, Amos B, von Seidlein L, et al. Invasive salmonellosis among children admitted to a rural Tanzanian hospital and a comparison with previous studies. PLoS ONE 2010; 5(2): e9244.
206. Mwaniki MK, Talbert AW, Njuguna P, et al. Clinical indicators of bacterial meningitis among neonates and young infants in rural Kenya. BMC Infect Dis 2011; 11: 301.
207. Nadjm B, Thuy PT, Trang VD, Ha LD, Kinh NV, Wertheim HF. Scrub typhus in the northern provinces of Vietnam: An observational study of admissions to a national referral hospital. Transactions of the Royal Society of Tropical Medicine and Hygiene 2014; 108(11): 739-40.
208. Nanzaluka FH, Davis WW, Mutale L, et al. Risk Factors for Epidemic Cholera in Lusaka, Zambia-2017. Am J Trop Med Hyg 2020; 103(2): 646-51.
209. Navoa-Ng JA, Berba R, Galapia YA, et al. Device-associated infections rates in adult, pediatric, and neonatal intensive care units of hospitals in the Philippines: International Nosocomial Infection Control Consortium (INICC) findings. Am J Infect Control 2011; 39(7): 548-54.
210. Negussie A, Getie A, Manaye E, Tekle T. Prevalence and outcome of injury in patients visiting the emergency Department of Yirgalem General Hospital, Southern Ethiopia. BMC emerg 2018; 18(1): 14.
211. Ngale K, Tia MA, Santos I, et al. Bed-sharing and risk of hospitalisation due to pneumonia and diarrhoea in infancy: the 2004 Pelotas Birth Cohort. Journal of Epidemiology & Community Health 2013; 67(3): 245-9.
212. Niekerk AV, Reimers A, Laflamme L. Area characteristics and determinants of hospitalised childhood burn injury: a study in the city of Cape Town. Public Health 2006; 120(2): 115-24.
213. Noblat ACB, Noblat LACB, Toledo LAKd, et al. Prevalência de admissão hospitalar por reação adversa a medicamentos em Salvador, BA. Rev Assoc Med Bras (1992) 2011; 57(1): 42-5.
214. Nokes DJ, Okiro EA, Ngama M, et al. Respiratory syncytial virus infection and disease in infants and young children observed from birth in Kilifi District, Kenya. Clin Infect Dis 2008; 46(1): 50-7.
215. Nunes AA, Caliani LS, Nunes MS, da Silva AS, de Mello LM. Profile analysis of patients with HIV/AIDS hospitalized after the introduction of antiretroviral therapy. Cienc 2015; 20(10): 3191-8.
216. Nunes AA, Caliani LS, Nunes MrS, Silva ASd, Mello LMd. Anílise do perfil de pacientes com HIV/Aids hospitalizados após introdução da terapia antirretroviral (HAART). Ciênc saúde coletiva 2015; 20(10): 3191-8.
217. Obiero CW, Mturi N, Mwarumba S, et al. Clinical features to distinguish meningitis among young infants at a rural Kenyan hospital. Arch Dis Child 2021; 106(2): 130-6.
218. Ogendi JO, Ayisi JG. Causes of injuries resulting in a visit to the emergency department of a Provincial General Hospital, Nyanza, western Kenya. Afr Health Sci 2011; 11(2): 255-61.
219. Ogundare EO, Olatunya OS, Oluwayemi IO, et al. Pattern and outcome of dog bite injuries among children in Ado-Ekiti, Southwest Nigeria. Pan Afr Med J 2017; 27: 81.
220. Ogunmola OJ, Olamoyegun MA. Patterns and outcomes of medical admissions in the accident and emergency department of a tertiary health center in a rural community of Ekiti, Nigeria. Journal of Emergencies, Trauma and Shock 2014; 7(4): 261-7.
221. Oguonu T, Adaeze Ayuk C, Edelu BO, Ndu IK. Pattern of respiratory diseases in children presenting to the paediatric emergency unit of the University of Nigeria Teaching Hospital, Enugu: a case series report. BMC polm 2014; 14: 101.
222. Okiro EA, Hay SI, Gikandi PW, et al. The decline in paediatric malaria admissions on the coast of Kenya. Malar J 2007; 6: 151.
223. Olotu AI, Mithwani S, Newton CR. Haemolytic uraemic syndrome in children admitted to a rural district hospital in Kenya. Trop Doct 2008; 38(3): 165-7.
224. Onyekwulu FA, Anya SU. Pattern of admission and outcome of patients admitted into the Intensive Care Unit of University of Nigeria Teaching Hospital Enugu: A 5-year review. Niger J Clin Pract 2015; 18(6): 775-9.
225. Onyemaechi N, Popoola S, Schuh A, Iorbo A, Elachi I, Oluwadiya K. Mortality Pattern of Hospitalized Surgical Patients in a Nigerian Tertiary Hospital. Indian Journal of Surgery 2015; 77: 881-5.
226. Onyiriuka AN, Ifebi E. Ketoacidosis at diagnosis of type 1 diabetes in children and adolescents: frequency and clinical characteristics. J 2013; 12(1): 47.
227. Oyedeji OA, Oluwayemi IO, Afolabi AA, Bolaji O, Fadero FF. Severe malaria at a tertiary paediatric emergency unit in South West Nigeria. Research Journal of Medical Sciences 2010; 4(6): 352-6.
228. Paez P, Suarez-Obando F, Zarante I. Genetic diseases in pediatric patients hospitalised in the town of Ubate, Colombia. Rev Salud Publica (Bogota) 2008; 10(3): 414-22.
229. Palaniswamy SR, Beniwal M, Venkataramaiah S, Srinivas D. Perioperative Management of Pediatric Giant Supratentorial Tumors: Challenges and Management Strategies. J 2019; 14(4): 211-7.
230. Pandey A, Ploubidis GB, Clarke L, Dandona L. Hospitalisation trends in India from serial cross-sectional nationwide surveys: 1995 to 2014. BMJ Open 2017; 7(12): e014188.
231. Parra T, Mujica L. Factores de riesgo psicosocial que afectan el comportamiento de escolares hospitalizados en la unidad de cuidados intermedios del Servicio Desconcentrado Hospital Pediítrico Dr. Agustí-n Zubillaga. Bol méd postgrado 2020; 36(1): 48-55.

232. Patel MR, Mushavi A, Balachandra S, et al. HIV-exposed uninfected infant morbidity and mortality within a nationally representative prospective cohort of mother-infant pairs in Zimbabwe. AIDS (02699370) 2020; 34(9): 1339-46.
233. Patra S, Pemde HK, Singh V, Chandra J, Dutta A. Profile of adolescents with severe anemia admitted in a tertiary care hospital in northern India. Indian J Pediatr 2011; 78(7): 863-5.
234. Paudel KM, Sharma S. Review of Paediatrics inpatient at a Zonal Hospital. Journal of Nepal Paediatric Society 2012; 32(3): 239-44.
235. Píez P, Suírez-Obando F, Zarante I. Enfermedades de origen genético en pacientes pediítricos hospitalizados en la Provincia de Ubate, Colombia. Rev salud pública 2008; 10(3): 414-22.
236. Perry HB, Ross AG, Fernand F. Assessing the causes of under-five mortality in the Albert Schweitzer Hospital service area of rural Haiti. 2005; 1(3): 178-86.
237. Piffer S, Demonti S, Ramponi C, Giustini M, Pitidis A. Home accidents in the province of Trento. Ten years of observations regarding admissions to the emergency and first aid department. Ann Ig 2021; 33(2): 152-62.
238. Prin M, Itaye T, Clark S, et al. Critical Care in a Tertiary Hospital in Malawi. World J Surg 2016; 40(11): 2635-42.
239. Ragoobirsingh D, McGrowder DA. A prospective study of children who were admitted for diabetes mellitus at a Jamaican hospital. Journal of Clinical and Diagnostic Research 2012; 6(5): 832-4.
240. Raihana S, Dunsmuir D, Huda T, et al. Development and Internal Validation of a Predictive Model Including Pulse Oximetry for Hospitalization of Under-Five Children in Bangladesh. PLoS ONE 2015; 10(11): e0143213.
241. Reyes L, Arvelo W, Estevez A, et al. Population-based surveillance for 2009 pandemic influenza A (H1N1) virus in Guatemala, 2009. Influenza other respi 2010; 4(3): 129-40.
242. Riyuzo MC, Silveira LV, Macedo CS, Fioretto JR. Predictive factors of mortality in pediatric patients with acute renal injury associated with sepsis. J Pediatr (Rio J) 2017; 93(1): 28-34.
243. Roberts T, Rattanavong S, Phommasone K, et al. Typhoid in Laos: An 18-Year Perspective. Am J Trop Med Hyg 2020; 102(4): 749.
244. Rocha LA, Froio SC, Silva CC, et al. Risk Factors for Mortality in Children with Congenital Heart Disease Delivered at a Brazilian Tertiary Center. Braz 2018; 33(6): 603-7.
245. Rodrigo GJ, Neffen H, Nannini LJ, Piñeyro L, Gené R, Plaza V. Hospitalizaciones por asma aguda durante el perí-odo 1994-2004 en Argentina y Uruguay: el estudio EAGLE. Rev am med respir 2009; 9(2): 41-8.
246. Rodriguez-Martinez CE, Acuna-Cordero R, Sossa-Briceno MP. Predictors of prolonged length of hospital stay or readmissions for acute viral lower respiratory tract infections among infants with a history of bronchopulmonary dysplasia. J Med Virol 2018; 90(3): 405-11.
247. Rooh ul M, Zareen M, Dilbag, Hayat M, Khan MI. Epidemiology and outcome of burns at Khyber Teaching Hospital Peshawar. Pakistan Journal of Medical Sciences 2007; 23(3): 420-4.
248. Rostagno G, Bonetto Gn, Saenz S. Escorpionismo en pacientes pediítricos internados en terapia intensiva: Serie de casos. Arch argent pediatr 2019; 117(4): 368-72.
249. Sadoh WE, Okperi B, Ikhurionan P, Monday P, Sadoh AE. Evaluation of Predictors of Mortality Associated With Childhood Heart Failure in Nigeria: A 2-Center Study. Pediatr Emerg Care 2018; 05: 05.
250. Saha S, Uddin MJ, Islam M, Das RC, Garrett D, Saha SK. Enteric Fever Cases in the Two Largest Pediatric Hospitals of Bangladesh: 2013-2014. J Infect Dis 2018; 218(suppl_4): S195-S200.
251. Sakita FM, Sawe HR, Mwafongo V, Mfinanga JA, Runyon MS, Murray BL. The Burden and Outcomes of Abdominal Pain among Children Presenting to an Emergency Department of a Tertiary Hospital in Tanzania: A Descriptive Cohort Study. emerg 2018; 2018: 3982648.
252. Samake BM, Coulibaly Y, Diani N, et al. Epidemiologic profile of acute intoxications at the Gabriel Toure University Hospital. Mali med 2011; 26(3): 34-6.
253. Sastry UMK, Jayranganath M, Kumar RK, et al. Thiamine-responsive acute severe pulmonary hypertension in exclusively breastfeeding infants: A prospective observational study. Archives of Disease in Childhood 2021; 106(3): 241-6.
254. Sawadogo N, Garanet F, Traore D, Some/Bere C, Mare Z, Dabire G. Morbidity of adolescents at the Kaya Regional Hospital Center (Burkina Faso). Mali med 2018; 33(1): 29-34.
255. Seale AC, Davies MR, Anampiu K, et al. Invasive Group A Streptococcus Infection among Children, Rural Kenya. Emerg Infect Dis 2016; 22(2): 224-32.
256. Shah K, Kandre Y, Mavalankar D. Secondary attack rate in household contacts of COVID-19 Paediatric index cases: a study from Western India. J Public Health (Oxf) 2021; 18: 18.
257. Shahid S, Tikmani SS, Nayani K, et al. Clinical signs predictive of severe illness in young Pakistani infants. BMC Res Notes 2021; 14(1): 71.
258. Shankar PR, Upadhyay DK, Subish P, Dubey AK, Mishra P. Prescribing patterns among paediatric inpatients in a teaching hospital in western Nepal. Singapore Med J 2006; 47(4): 261-5.
259. Sharma D, Panta P, Amgain K. An epidemiological study of injuries in Karnali, Nepal. Journal of Emergencies, Trauma & Shock 2020; 13(1): 30-4.

260. Sheikh AM, Rwegerera GM, Godman B, Habte D. Adjustments of medication dosages in patients with renal impairment in Botswana; findings and implications to improve patient care. Hosp Pract (Minneap) 2019; 47(5): 231-40.
261. Shusil MPK, Agarwal A, Rastogi AK. Demographic profile and pattern of presentation of organophosphorus poisoning at tertiary care hospital agra. Indian Journal of Forensic Medicine and Toxicology 2020; 14(3): 39-44.
262. Sigauque B, Roca A, Sanz S, et al. Acute bacterial meningitis among children, in Manhica, a rural area in Southern Mozambique. Acta Trop 2008; 105(1): 21-7.
263. Simmerman JM, Chittaganpitch M, Levy J, et al. Incidence, seasonality and mortality associated with influenza pneumonia in Thailand: 2005-2008. PLoS ONE 2009; 4(11): e7776.
264. Singh K, Speizer I, Barker PM, et al. Evaluating the impact of a hospital scale-up phase of a quality improvement intervention in Ghana on mortality for children under five. International Journal for Quality in Health Care 2019; 31(10): 752-8.
265. Singhal T, Kothari V. Clinical and Laboratory Profile of Fatal Dengue Cases at a Tertiary Care Private Hospital in Mumbai, India. Am J Trop Med Hyg 2020; 103(3): 1223-7.
266. Sirikarn P, Pattanittum P, Sawanyawisuth K, Tiamkao S. Causes of death in patients with status epilepticus. Epilepsy Behav 2019; 101(Pt B): 106372.
267. Smith Z, Ayele Y, McDonald P, Smith ZA. Outcomes in critical care delivery at Jimma University Specialised Hospital, Ethiopia. Anaesthesia & Intensive Care 2013; 41(3): 363-8.
268. Sornsrivichai V, Chongsuvivatwong V, Mo-suwan L, Intusoma U. Hospitalized infant morbidity in the Prospective Cohort Study of Thai Children Project. J Med Assoc Thai 2008; 91(6): 882-8.
269. Studart-Botto P, Martins-Junior DF, Sarmento S, Argolo L, Galvao-de-Almeida A, Miranda-Scippa A. Self-injurious behavior and related mortality in children under 10 years of age: a retrospective health record study in Brazil. Rev Bras Psiquiatr 2019; 05: 05.
270. Sutra S, Chirawatkul A, Leelapanmetha P, Sirisuwan S, Thepsuthammarat K. Evaluation of causes-of-death: which statistics should we rely on, hospital deaths or vital statistics? J Med Assoc Thai 2012; 95 Suppl 7: S262-73.
271. Tabish A, Lone NA, Afzal WM, Salam A. The incidence and severity of injury in children hospitalised for traumatic brain injury in Kashmir. Injury 2006; 37(5): 410-5.
272. Tak V, Mathur P, Kumar S, et al. Impact of an intensive surveillance on central line associated blood stream infections at an Indian trauma center. Journal of Patient Safety and Infection Control 2014; 2(2): 38-41.
273. Talbert AW, Mwaniki M, Mwarumba S, Newton CR, Berkley JA. Invasive bacterial infections in neonates and young infants born outside hospital admitted to a rural hospital in Kenya. Pediatr Infect Dis J 2010; 29(10): 945-9.
274. Teixeira Lopes MCB, de Aguiar W, Yamaguchi Whitaker I. In-hospital Complications in Trauma Patients According to Injury Severity. Journal of trauma nursing : the official journal of the Society of Trauma Nurses 2019; 26(1): 10-6.
275. Thai PQ, Toan DTT, Son DT, et al. Factors associated with the duration of hospitalisation among COVID-19 patients in Vietnam: A survival analysis. Epidemiol Infect 2020; 148: e114.
276. Thang VV, Bao TQQ, Tuyen HD, et al. Incidence of snakebites in Can Tho Municipality, Mekong Delta, South Vietnam-Evaluation of the responsible snake species and treatment of snakebite envenoming. PLoS Negl Trop Dis 2020; 14(6): e0008430.
277. Thanni LO, Kehinde OA. Trauma at a Nigerian teaching hospital: pattern and docu-mentation of presentation. Afr Health Sci 2006; 6(2): 104-7.
278. Thompson DC, Crooks RJ, Clasper JC, Lupu A, Stapley SA, Cloke DJ. The pattern of paediatric blast injury in Afghanistan. J R Army Med Corps 2017; 21: 21.
279. Tshimangani T, Pongo J, Bodi Mabiala J, Yotebieng M, O'Brien NF. Pediatric Acute Severe Neurologic Illness and Injury in an Urban and a Rural Hospital in the Democratic Republic of the Congo. Am J Trop Med Hyg 2018; 98(5): 1534-40.
280. Tumusiime G, Was A, Preston MA, Riesel JN, Ttendo SS, Firth PG. The Quality and Utility of Surgical and Anesthetic Data at a Ugandan Regional Referral Hospital. World J Surg 2017; 41(2): 370-9.
281. Turbau Valls M, Gil Olivas E, Lopez Garcia T, Piedade D, Pessela A, Nicasio MM. Epidemiological and clinical features of the emergency visits in a rural hospital in Cubal, Angola. Pan Afr Med J 2018; 29: 143.
282. Uche EO, Ezomike UO, Chukwu JC, Ituen MA. Intensive care unit admissions in Federal Medical Centre Umuahia south east Nigeria. Niger J Med 2012; 21(1): 70-3.
283. Ugwu GIM, Okperi BO, Chinemelu UCO. Pattern and outcome of presentation at the children emergency unit of a tetiary institution in the Niger Delta region of Nigeria: A one year prospective study. Journal of Medicine (Bangladesh) 2012; 13(2): 170-3.
284. Valadas E, Gomes A, Sutre A, et al. Tuberculosis with malaria or HIV co-infection in a large hospital in Luanda, Angola. J 2013; 7(3): 269-72.
285. van Deursen B, Lenglet A, Ariti C, et al. Risks and seasonal pattern for mortality among hospitalized infants in a conflict-affected area of Pakistan, 2013-2016. A retrospective chart review. [version 1; peer review: 2 approved, 1 approved with reservations]. F1000Research 2019; 8.

286. van Immerzeel TD, Mukasa O, Schmitt TS, Moorman PW. Morbidity and mortality on a paediatric ward in rural Tanzania. Trop Doct 2006; 36(3): 171-3.
287. Vanderburg S, Wijayaratne G, Danthanarayana N, et al. Outbreak of severe acute respiratory infection in Southern Province, Sri Lanka in 2018: a cross-sectional study. BMJ Open 2020; 10(11): e040612.
288. Veirum JE, Sodemann M, Biai S, et al. Routine vaccinations associated with divergent effects on female and male mortality at the paediatric ward in Bissau, Guinea-Bissau. Vaccine 2005; 23(9): 1197-204.
289. Von Dessauer GB, Ortiz FP, Hinostroza CT, et al. Intento de suicidio vía ingesta de fármacos en niños. Rev chil pediatr 2011; 82(1): 42-8.
290. Wain H, Kong V, Bruce J, Laing G, Clarke D. Analysis of Surgical Adverse Events at a Major University Hospital in South Africa. World J Surg 2019; 43(9): 2117-22.
291. Wessels EU, Kong VY, Buitendag J, et al. The spectrum of animal related injuries managed at a major trauma centre in South Africa. South African journal of surgery Suid-Afrikaanse tydskrif vir chirurgie 2019; 57(3): 54.
292. Wiens MO, Gan H, Barigye C, et al. A cohort study of morbidity, mortality and health seeking behavior following rural health center visits by children under 12 in southwestern Uganda. PLoS ONE 2015; 10(1): e0118055.
293. Yaich S, Jdidi J, Karray A, Fki H, Kassis M, Damak J. Adolescent's hospital morbidity in Sfax - Tunisia. Tunis Med 2010; 88(6): 399-403.
294. Yeboah-Antwi K, Addo-Yobo E, Adu-Sarkodie Y, et al. Clinico-epidemiological profile and predictors of severe illness in young infants (0-59 days) in Ghana. Annals of Tropical Paediatrics 2008; 28(1): 35-43.
295. Yore MA, Strehlow MC, Yan LD, et al. Characteristics and outcomes of pediatric patients presenting at Cambodian referral hospitals without appointments: an observational study. Int J Emerg Med 2018; 11(1): 17.
296. Young Infants Clinical Signs Study Group. Clinical signs that predict severe illness in children under age 2 months: a multicentre study. Lancet 2008; 371 North American Edition(9607): 135-42.
297. Zarif P, Uddin SZ, Bashir N. Analytical study of acute poisoning cases admitted in Lahore General Hospital, Lahore, Pakistan. Medical Forum Monthly 2018; 29(6): 102-5.
298. Zeidan ZA, Kojal EM, Habour AB, Nowary KA, Mohammed FH, Awadelkareem MA. Severe malaria in sudanese children: clinical aspects and prognosis in hospitilized patients. J Family Community Med 2005; 12(3): 127-32.

Ineligible setting (N=125)

1. Adil E, Tarshish Y, Roberson D, Jang J, Licameli G, Kenna M. The Public Health Impact of Pediatric Deep Neck Space Infections. Otolaryngology-Head & Neck Surgery 2015; 153(6): 1036-41.
2. Ahmed AS, Halabi Z, Antoun J. The effect of the waste disposal crisis on the rates of hospitalization due to acute diarrheal illness in a middle-income country: Retrospective chart review. Int J Infect Dis 2020; 90: 65-70.
3. Al Subaie SS, Al Saadi MA, Al Subaie SS, Al Saadi MA. Features associated with severe disease in hospitalized children with 2009 influenza A (H1N1) infection at a university hospital in Riyadh, Saudi Arabia. Annals of Saudi Medicine 2012; 32(1): 53-8.
4. Al-Busaidi M, Al Maamari K, Al'Adawi B, Ba Alawi F, Al-Wahaibi A, Belkhair A. Pandemic influenza a H1N1 in Oman: Epidemiology, clinical features, and outcome of patients admitted to sultan qaboos university hospital in 2009. Oman Medical Journal 2016; 31(4): 290-7.
5. Al-Dulimi HKH, Abosalah S, Abdulaziz A, Mohammed T, Bener AB. Trauma mortality in the State of Qatar, 2006-2007. Journal of Emergency Medicine, Trauma and Acute Care 2009; 9(1): 19-26.
6. Albert BD, Zurakowski D, Bechard LJ, et al. Enteral Nutrition and Acid-Suppressive Therapy in the PICU: Impact on the Risk of Ventilator-Associated Pneumoniaâˆ—. Pediatric Critical Care Medicine 2016; 17(10): 924-9.
7. Alhabdan S, Zamakhshary M, AlNaimi M, et al. Epidemiology of traumatic head injury in children and adolescents in a major trauma center in Saudi Arabia: implications for injury prevention. Ann Saudi Med 2013; 33(1): 52-6.
8. Ali Z, Harastani H, Hammadi M, et al. Rotavirus Genotypes and Vaccine Effectiveness from a Sentinel, Hospital-Based, Surveillance Study for Three Consecutive Rotavirus Seasons in Lebanon. PLoS ONE 2016; 11(8): e0161345.
9. Aminzadeh M, Navidi N, Valavi E, Aletayeb SMH. Childhood onset type 1 diabetes at a tertiary hospital in south-western Iran during 2000-2015: Rapid increase in admissions and high prevalence of DKA at diagnosis. Prim Care Diabetes 2019; 13(1): 43-8.
10. Andrade Filho VS, Artaxo P, Hacon S, Carmo CN, Cirino G. Aerosols from biomass burning and respiratory diseases in children, Manaus, Northern Brazil. Rev Saude Publica 2013; 47(2): 239-47.
11. André-von Arnim A, Brogan TV, Hertzig J, et al. Intensive care for infants and children in Haiti in April 2010. Pediatric Critical Care Medicine 2011; 12(4): 393-7.
12. Araujo da Silva AR, de Souza CV, Viana ME, Sargentelli G, de Andrada Serpa MJ, Gomes MZ. Health care-associated infection and hospital readmission in a home care service for children. Am J Infect Control 2012; 40(3): 282-3.

13. Arias P Hc, Peña R K. Pronóstico de mortalidad al ingreso en la unidad de cuidados intensivos pediítricos del Hospital Regional de Talca. Rev méd Maule 2005; 23(1/2): 4-7.
14. Arias P Hc, Selamé R MaE, Espina S Is. Influenza A H1N1: caracterí-sticas clínico-epidemiológicas de 26 pacientes hospitalizados en Hospital Regional de Talca. Rev méd Maule 2010; 26(1): 11-5.
15. Ashshi AM. Is rotavirus infection still responsible for acute gastroenteritis and severe diarrhea among children in Holy Makkah? Research Journal of Medical Sciences 2012; 6(4): 170-4.
16. Atıcı, S, Soysal A, Kadayifci EK, et al. Healthcare-associated infections in a newly opened pediatric intensive care unit in turkey: Results of four-year surveillance. Journal of Infection in Developing Countries 2016; 10(3): 254-9.
17. Ayar G, Yakut HI, Men Atmaca Y, Emeksiz S, Özkaya Parlakay A. Evaluation of Healthcare-Acquired Infection Rates in a Pediatric Intensive Care Unit in Turkey. Journal of Pediatric Infectious Diseases 2019; 14(5): 235-41.
18. Ballot DE, Ramdin T, White DA, Lipman J. A comparison between raw and predicted mortality in a paediatric intensive care unit in South Africa. BMC Res Notes 2018; 11(1): 829.
19. Banasiak NC, Meadows-Oliver M. Prevalence of asthma at a school-based health clinic in Nicaragua. Journal for Specialists in Pediatric Nursing 2020; 25(3): 1-5.
20. Bar-On E, Lebel E, Blumberg N, Sagi R, Kreiss Y. Pediatric Orthopedic Injuries Following an Earthquake: Experience in an Acute-Phase Field Hospital. Journal of trauma nursing : the official journal of the Society of Trauma Nurses 2015; 22(4): 223-8.
21. Bar-On E, Lebel E, Kreiss Y, et al. Orthopaedic management in a mega mass casualty situation. The Israel Defence Forces Field Hospital in Haiti following the January 2010 earthquake. Injury 2011; 42(10): 1053-9.
22. Baykan N, Aslaner MA. The use of emergency department and outpatient clinics by Syrian refugees. J 2019; 9(2): 020404.
23. Bhalla K, Naghavi M, Shahraz S, Bartels D, Murray CJ. Building national estimates of the burden of road traffic injuries in developing countries from all available data sources: Iran. Inj Prev 2009; 15(3): 150-6.
24. Blanc J, Locatelli I, Rarau P, et al. Retrospective study on the usefulness of pulse oximetry for the identification of young children with severe illnesses and severe pneumonia in a rural outpatient clinic of Papua New Guinea. PLoS ONE 2019; 14(4): e0213937.
25. Brimkulov N, Louton L, Sydykova S, Vinnikov D, Imanalieva F. Morbidity in the Mountainous Province of Kyrgyzstan: Results from a Population-Based Cross-Sectional Study. High Alt Med Biol 2017; 18(4): 338-42.
26. Brondex A, Viant E, Trendel D, Puidupin M. Medical activity in the Conventional Hospitalization Unit in Kabul NATO Role 3 Hospital: a 3-month-long experience. Mil Med 2014; 179(2): 197-202.
27. Burnett MW, Spinella PC, Azarow KS, Callahan CW. Pediatric care as part of the US Army medical mission in the global war on terrorism in Afghanistan and Iraq, December 2001 to December 2004. Pediatrics 2008; 121(2): 261-5.
28. Carcillo JA, Dean JM, Holubkov R, et al. Inherent Risk Factors for Nosocomial Infection in the Long Stay Critically Ill Child Without Known Baseline Immunocompromise: A Post Hoc Analysis of the CRISIS Trial. Pediatric Infectious Disease Journal 2016; 35(11): 1182-6.
29. Carmo GMID, Yen C, Cortes J, et al. Decline in diarrhea mortality and admissions after routine childhood rotavirus immunization in Brazil: A time-series analysis. PLoS Medicine 2011; 8(4): 11.
30. Castro MS, Carvalho MS, Travassos C. Factors associated with readmission to a general hospital in Brazil. Cadernos de saúde pública / Ministério da Saúde, FundaÃ§Ã£o Oswaldo Cruz, Escola Nacional de Saúde Pública 2005; 21(4): 1186-200.
31. Cha WC, Shin SD, Cho JS, Song KJ, Singer AJ, Kwak YH. The association between crowding and mortality in admitted pediatric patients from mixed adult-pediatric emergency departments in Korea. Pediatric Emergency Care 2011; 27(12): 1136-41.
32. Collard Borsotti MV. Evaluación de la adecuación de ingresos y estadías de los pacientes internados en el hospitla de niños de la Santísima Trinidad de Córdoba: Argentina. 2012: 59-.
33. De Moraes Silveira CR, De Mello ED, Antonacci Carvalho PR. Evolution of nutritional status of pediatric in patients of a tertiary care general hospital in Brazil. Nutr Hosp 2008; 23(6): 599-606.
34. Duek OS, Ben Naftali Y, Pikkel YY, et al. Coffee break - Epidemiologic analysis, traditions & holidays that increase burn risk of pediatric patients. Burns 2021.
35. Edwards MJ, Lustik M, Eichelberger MR, Elster E, Azarow K, Coppola C. Blast injury in children: an analysis from Afghanistan and Iraq, 2002-2010. J Trauma Acute Care Surg 2012; 73(5): 1278-83.
36. Ee Lin T, Shaun Wen Huey L, Jamaluddin SF, et al. The epidemiology of childhood brain injury in the state of Selangor and Federal Territory of Kuala Lumpur, Malaysia. BMC Pediatrics 2016; 16: 1-6.
37. El Desoky S, Mashat S, Bana S, et al. Efficiency of Using Pediatrics Emergency Services and Triage Evaluation. Pediatr Emerg Care 2018; 34(6): 417-21.
38. El-Menyar A, Consunji R, Al-Thani H, Mekkodathil A, Jabbour G, Alyafei KA. Pediatric Traumatic Brain Injury: a 5-year descriptive study from the National Trauma Center in Qatar. World J Emerg Surg 2017; 12: 48.
39. Farias JA, Ferníndez A, Monteverde E, et al. Mechanical ventilation in pediatric intensive care units during the season for acute lower respiratory infection: a multicenter study. Pediatric Critical Care Medicine 2012; 13(2): 158-64.

40. Ferníndez A, Alonso L, Baldovino R, et al. Rendimiento del score PIM 2 en una Unidad de Cuidados Intensivos Pediítricos. Arch pediatr Urug 2016; 87(3): 234-9.
41. Field-Ridley A, Dharmar M, Steinhorn D, McDonald C, Marcin JP. ICU-Acquired Weakness Is Associated With Differences in Clinical Outcomes in Critically Ill Children. Pediatric Critical Care Medicine 2016; 17(1): 53-7.
42. Foong Ng K, Kee Tan K, Hong Ng B, Nair P, Ying Gan W. Epidemiology of adenovirus respiratory infections among hospitalized children in Seremban, Malaysia. Trans R Soc Trop Med Hyg 2015; 109(7): 433-9.
43. Giangioppo S, Bijelic V, Barrowman N, Radhakrishnan D. Emergency department visit count: a practical tool to predict asthma hospitalization in children. Journal of Asthma 2020; 57(10): 1043-52.
44. Gianserra CV, Aguero AP, Chapelet AG, Paradiso B, Spanevello VA, Del Pino MA. Anemia intrahospitalaria y descenso de hemoglobina en pacientes internados. Medicina (BAires) 2011; 71(3): 201-6.
45. Giraldo D, Sant'Anna C, Perisse AR, et al. Characteristics of children hospitalized with dengue fever in an outbreak in Rio de Janeiro, Brazil. Trans R Soc Trop Med Hyg 2011; 105(10): 601-3.
46. Gold DL, Mihalov LK, Cohen DM. Evaluating the Pediatric Early Warning Score (PEWS) system for admitted patients in the pediatric emergency department. Acad Emerg Med 2014; 21(11): 1249-56.
47. Gomila AA, De Grandis ES, Visconti GB, et al. Nutritional status in children hospitalized in minimum care wards. Hospital de Niños de la Santísima Trinidad. Córdoba. Archivos Argentinos de Pediatria 2009; 107(1): 37-42.
48. Göngör A, Čatak AI, Čakir BC, et al. Evaluation of Syrian refugees who received inpatient treatment in a tertiary pediatric hospital in Turkey between January 2016 and August 2017. International Health 2018; 10(5): 371-5.
49. Haghparast-Bidgoli H, Saadat S, Bogg L, Yarmohammadian MH, Hasselberg M. Factors affecting hospital length of stay and hospital charges associated with road traffic-related injuries in Iran. BMC Health Serv Res 2013; 13: 281.
50. Han T, Han K, Kim J, et al. Pediatric hand injury induced by treadmill. Burns (03054179) 2005; 31(7): 906-9.
51. Han T, Kim J, Yang M, et al. A retrospective analysis of 19,157 burns patients: 18-year experience from Hallym Burn Center in Seoul, Korea. Burns (03054179) 2005; 31(4): 465-70.
52. Hariharan S, Krishnamurthy K, Grannum D. Validation of Pediatric Index of Mortality-2 scoring system in a pediatric intensive care unit, Barbados. J Trop Pediatr 2011; 57(1): 9-13.
53. Hekimoglu HC, Demirci NA. Evaluation of cases with a preliminary diagnosis of Crimean- Congo hemorrhagic fever and comparison of characteristics in patients admitted to a secondary care hospital in Kastamonu, Turkey. Afr Health Sci 2014; 14(4): 873-81.
54. Herbert HK, van As AB, Bachani AM, et al. Patterns of pediatric injury in South Africa: an analysis of hospital data between 1997 and 2006. J Trauma Acute Care Surg 2012; 73(1): 168-74.
55. Hwang J, Chang H. Understanding non-emergency patients admitted to hospitals through the emergency department for efficient ED functions. JEN: Journal of Emergency Nursing 2010; 36(3): 196-202.
56. Iloh GU, Ofoedu JN, Njoku PU, Amadi AN, Godswill-Uko EU. The Magnitude of Under-five Emergencies in a Resource-poor Environment of a Rural Hospital in Eastern Nigeria: Implication for
57. Strengthening the House-hold and Community-integrated Management of Childhood Illnesses. N A J Med Sci (Hamilt) 2012; 4(8): 344-9.
58. Jacobs C, Vacek J, Many B, Bouchard M, Abdullah F. An Analysis of Factors Associated with Burn Injury Outcomes in Low- and Middle-Income Countries. J Surg Res 2021; 257: 442-8.
59. Jatau AI, Aung MMT, Kamauzaman THT, Rahman AFA. Prevalence of Drug-Related Emergency Department Visits at a Teaching Hospital in Malaysia. Drugs - Real World Outcomes 2015; 2(4): 387-95.
60. Junqueira RMP, Duarte EC. Hospitalizations due to ambulatory care-sensitive conditions in the Federal District, Brazil, 2008. Revista de Saude Publica 2012; 46(5): 761-8.
61. Kanoksil M, Jatapai A, Peacock SJ, Limmathurotsakul D. Epidemiology, microbiology and mortality associated with community-acquired bacteremia in northeast Thailand: a multicenter surveillance study. PLoS ONE 2013; 8(1): e54714.
62. Kazemian H, Pourmand MR, Pourramezan N, et al. Evaluation of healthcare-associated infections in Ardabil hospitals, Iran, 2014-2015. Research Journal of Pharmaceutical, Biological and Chemical Sciences 2016; 7(5): 898-903.
63. Kelishadi R, Barakati SH, Lornejad HR, Amiri M, Motlagh ME. Mortality-related factors disparity among Iranian deceased children aged 1-59 months according to the medical activities in emergency units: National mortality surveillance system. J 2012; 17(12): 1096-101.
64. Khajeh A, Narouie B, Noori NM, et al. Patterns of acute poisoning in childhood and relative factors in Zahedan, Southeast Iran. Shiraz E Medical Journal 2012; 13(1): 19-27.
65. Khajeh A, Mohammad Noori N, Reisi M, Fayyazi A, Mohammadi M, Miri-Aliabad G. Mortality Risk Prediction by Application of Pediatric Risk of Mortality Scoring System in Pediatric Intensive Care Unit. Iranian Journal of Pediatrics 2013; 23(5): 546-50.
66. Kim J, Lee MU, Kim T-H, Mayr J. Nationwide epidemiologic study for pediatric osteomyelitis and septic arthritis in South Korea: A cross-sectional study of national health insurance review and assessment service. Medicine 2019; 98(17): e15355-e.

67. Kim KW, Ahn K, Yang HJ, et al. Humidifier disinfectant-associated children's interstitial lung disease. American Journal of Respiratory & Critical Care Medicine 2014; 189(1): 48-56.
68. Kim SA, Kilgore PE, Lee SY, et al. Trends in pneumonia and influenza-associated hospitalizations in South Korea, 2002-2005. Journal of Health, Population & Nutrition 2011; 29(6): 574-82
69. Kim YK, Nyambat B, Hong YS, Lee CG, Lee JW, Kilgore PE. Burden of viral respiratory disease hospitalizations among children in a community of Seoul, Republic of Korea, 1995 - 2005. Scandinavian Journal of Infectious Diseases 2008; 40(11/12): 946-53.
70. Kim YA, Ha E-J, Jhang WK, Park SJ. Early blood lactate area as a prognostic marker in pediatric septic shock. Intensive Care Medicine 2013; 39(10): 1818-23.
71. Lee CH, Won YK, Roh EJ, Suh DI, Chung EH. A nationwide study of children and adolescents with pneumonia who visited Emergency Department in South Korea in 2012. Korean J 2016; 59(3): 132-8.
72. Lee E, Kim C-H, Lee YJ, et al. Annual and seasonal patterns in etiologies of pediatric community-acquired pneumonia due to respiratory viruses and Mycoplasma pneumoniae requiring hospitalization in South Korea. BMC Infectious Diseases 2020; 20(1): 1-10.
73. Lee WS, Ahmad Z. The prevalence of undernutrition upon hospitalization in children in a developing country: A single hospital study from Malaysia. Pediatr neonatol 2017; 58(5): 415-20.
74. Linsuwanon P, Payungporn S, Samransamruajkit R, et al. High prevalence of human rhinovirus C infection in Thai children with acute lower respiratory tract disease. J Infect 2009; 59(2): 115-21.
75. Liu J, Ai H, Xiong Y, et al. Prevalence and correlation of infectious agents in hospitalized children with acute respiratory tract infections in Central China. PLoS ONE 2015; 10(3): e0119170.
76. Lucion MaF, Juarez MV, Viegas M, et al. Virus respiratorio sincicial: Patrón clínico epidemiológico en niños internados en un hospital pediátrico durante los años 2000-2013. Arch argent pediatr 2014; 112(5): 397-404.
77. Lum LC, Abdel-Latif ME, de Bruyne JA, Nathan AM, Gan CS. Noninvasive ventilation in a tertiary pediatric intensive care unit in a middle-income country. Pediatr Crit Care Med 2011; 12(1): e7-13.
78. Machado K, Pérez W, Pé-rez C, Stoll M. Hospitalizaciones por IRAB de probable etiologí-a viral en niños durante el año 2012: estrategia asistencial. Arch pediatr Urug 2016; 87(1): 5-11.
79. Matijasevich A, Cesar JA, Santos IS, et al. Hospitalizations during infancy in three population-based studies in Southern Brazil: trends and differentials. 2008; 1: S437-43.
80. Mírquez S, Vomero A, Tapie A, Famolaro Ma, Raggio V, Giachetto G. Hospitalización relacionada con defectos congénitos en el Centro Hospitalario Pereira Rossell. Arch pediatr Urug 2015; 86(3): 167-75.
81. Mianling Z, Yuge H, Tufeng L, et al. Performance of the pediatric sequential organ failure assessment score in assessing the prognosis of children with sepsis in a PICU of a developing country: A single-center retrospective observational study. Iranian Journal of Pediatrics 2019; 29(5).
82. Miftode EG, Dorneanu OS, Leca DA, et al. Tuberculous Meningitis in Children and Adults: A 10-Year Retrospective Comparative Analysis. PLoS ONE 2015; 10(7): e0133477.
83. Modaghegh MH, Saremi E, Mohamadian M, Jafarzadeh R. Characteristics of trauma in North East Iran and the prevention strategies. Arch Iran Med 2013; 16(10): 576-9.
84. Moitinho de Almeida M, Schluter BS, van Loenhout JAF, et al. Changes in patient admissions after the 2015 Earthquake: a tertiary hospital-based study in Kathmandu, Nepal. Sci 2020; 10(1): 4956.
85. Morosini Pérez FP, Dalgalarrondo Reistaino A, Gerolami Dondo A, et al. Estrategia de hospitalización abreviada en el síndrome bronquial obstructivo del lactante. Experiencia de tres años en Unidades de Terapia Inhalatoria. Arch pediatr Urug 2012; 83(2): 103-10.
86. Movahedi Z, Mahmoudi S, Pourakbari B, et al. Epidemiology of children with acquired immune deficiency syndrome (stage 3): A referral hospital-based study in Iran. J Med Virol 2016; 88(1): 64-8.
87. Naaman O, Yulevich A, Sweed Y. Syria civil war pediatric casualties treated at a single medical center. J Pediatr Surg 2019; 02: 02.
88. Nathan AM, Rani F, Lee RJ, et al. Clinical risk factors for life-threatening lower respiratory tract infections in children: a retrospective study in an urban city in Malaysia. PLoS ONE 2014; 9(10): e111162.
89. Naveda Romero OE, Naveda Melendez AF. Fluid overload and kidney failure in children with severe sepsis and septic shock: A cohort study. Arch Argent Pediatr 2017; 115(2): 118-24.
90. Naylor JF, April MD, Thronson EE, Hill GJ, Schauer SG. U.S. Military Medical Evacuation and Prehospital Care of Pediatric Trauma Casualties in Iraq and Afghanistan. Prehosp Emerg Care 2020; 24(2): 265-72.
91. Neff LP, Cannon JW, Morrison JJ, Edwards MJ, Spinella PC, Borgman MA. Clearly defining pediatric massive transfusion: Cutting through the fog and friction with combat data. Journal of Trauma and Acute Care Surgery 2015; 78(1): 22-8.
92. Neyro SE, Ferolla FM, Molise C, et al. Impacto clí-nico y epidemiológico de las infecciones por varicela en niños previo a la introducción de la vacuna al Calendario Nacional de Argentina. Arch argent pediatr 2019; 117(1): 12-8.
93. Notejane M, Pandolfo S, Garcí-a L, et al. Gastroenteritis aguda: formas de presentación clínica y etiología en niños hospitalizados en el Hospital Pediátrico, Centro Hospitalario Pereira Rossell, año 2012. Arch pediatr Urug 2015; 86(2): 91-7.

94. Oprescu F, Peek-Asa C, Wallis A, Young T, Nour D, CherecheÅŸ R. Pediatric Poisonings and Risk Markers for Hospital Admission in a Major Emergency Department in Romania. Maternal & Child Health Journal 2012; 16(2): 495-500.
95. Park JW, Jung JH, Kwak YH, Jung JY, Wang S-M. Epidemiology of pediatric visits to the emergency department due to foreign body injuries in South Korea: Nationwide cross-sectional study. Medicine 2019; 98(22): e15838-e.
96. Plakht Y, Shiyovich A, Francine L, et al. The emergency department in a region under missile attack: utilization patterns during Operation Cast Lead. Isr Med Assoc J 2011; 13(2): 69-75.
97. Poo MI, Lee WS. Admission to hospital with childhood acute gastroenteritis in Kuala Lumpur, Malaysia. Medical Journal of Malaysia 2007; 62(3): 189-93.
98. Prerna A, Lim JY, Tan NW, et al. Neurology of the H1N1 pandemic in Singapore: a nationwide case series of children and adults. J Neurovirol 2015; 21(5): 491-9.
99. Rey C, Sínchez-Arango D, López-Herce Js, et al. Vitamin D deficiency at pediatric intensive care admission. J pediatr (Rio J) 2014; 90(2): 135-42.
100. Ronaldi M, Suwibawa IBG, Kadafi KT, Dewi NE, Effendi JA, Pulungan AB. The impacts of 2018 earthquakes to the pediatric population in north lombok field hospital, West Nusa Tenggara, Indonesia. Current Pediatric Research 2020; 24(1): 172-4.
101. Roodpeyma S, Hekmat M, Dordkhar M, Rafieyian S, Hashemi A. A prospective observational study of paediatric cardiac surgery outcomes in a postoperative intensive care unit in Iran. JPMA J Pak Med Assoc 2013; 63(1): 55-9.
102. Rosenthal VD, Bat-Erdene I, Gupta D, et al. Six-year multicenter study on short-term peripheral venous catheters-related bloodstream infection rates in 727 intensive care units of 268 hospitals in 141 cities of 42 countries of Africa, the Americas, Eastern Mediterranean, Europe, South East Asia, an. Infection Control and Hospital Epidemiology 2020; 41(5): 553-63.
103. Royal C, McKerrow NH. A retrospective review of the transfer of critically ill children to tertiary care in KwaZulu-Natal, South Africa. SAJCH South African Journal of Child Health 2015; 9(4): 112-8.
104. Safari S, Hashemi B, Forouzanfar MM, Shahhoseini M, Heidari M. Epidemiology and outcome of patients with acute kidney injury in emergency department; a cross-sectional study. Emergency 2018; 6(1).
105. Santos E, Goncalves de Sousa A, Marques AP, Bussamra MH, Zukerman E, Schvartsman C. Reduction in hospitalizations of children. Acta Paediatr 2008; 97(12): 1712-6.
106. Schauer SG, Hill GJ, Naylor JF, April MD, Borgman M, Bebarta VS. Emergency department resuscitation of pediatric trauma patients in Iraq and Afghanistan. Am J Emerg Med 2018; 36(9): 1540-4.
107. Secmeer G, Ciftci AO, Kanra G, et al. Community-acquired pneumonia and parapneumonic effusions in developing countries. Turk J Pediatr 2008; 50(1): 51-7.
108. Shiraki A, Yasui M, Kidokoro H, et al. Initial treatment of seizures in children in an emergency department in rural Japan. Brain Dev 2021; 43(2): 288-93.
109. Sorup S, Benn CS, Stensballe LG, Aaby P, Ravn H. Measles-mumps-rubella vaccination and respiratory syncytial virus-associated hospital contact. Vaccine 2015; 33(1): 237-45.
110. Sutra S, Kosuwon P, Chirawatkul A, Thepsuthammarat K. Burden of acute, persistent and chronic diarrhea, Thailand, 2010. J Med Assoc Thai 2012; 95 Suppl 7: S97-107.
111. Tempia S, Walaza S, Moyes J, et al. Risk Factors for Influenza-Associated Severe Acute Respiratory Illness Hospitalization in South Africa, 2012-2015. Open forum infect 2017; 4(1): ofw262.
112. Thrane N, Søndergaard C, Schønheyder HC, Sørensen HT. Socioeconomic factors and risk of hospitalization with infectious diseases in 0- to 2-year-old Danish children. European Journal of Epidemiology 2005; 20(5): 467-74.
113. Torabian S, Saba MS. Epidemiology of paediatric burn injuries in Hamadan, Iran. Burns (03054179) 2009; 35(8): 1147-51.
114. Totapally A, Martinez P, Raszynski A, Alkhoury F, Totapally BR. Do Racial/Ethnic and Economic Factors Affect the Rate of Complicated Appendicitis in Children? Surgery Research and Practice 2020; 2020.
115. Vahdati SS, Karzar BH, Momen N. Independent predictive factors of hospitalization in a north-west Burn center of Iran: An epidemiologic study. Emergency 2015; 3(1): 40-4.
116. Wang Y, Sun B, Yue H, et al. An epidemiologic survey of pediatric sepsis in regional hospitals in China. Pediatr Crit Care Med 2014; 15(9): 814-20.
117. Wehrmeister FC, Victora CG, Horta BL, et al. Hospital admissions in the first year of life: Inequalities over three decades in a southern Brazilian city. International Journal of Epidemiology 2019; 48: I63-I71.
118. Wu PS, Chang IS, Tsai FY, et al. Epidemiology and impacts of children hospitalized with pneumonia from 1997 to 2004 in Taiwan. Pediatr Pulmonol 2009; 44(2): 162-6.
119. Wu Z, Liang Y, Li Z, et al. Accuracy Comparison Between Age-Adapted SOFA and SIRS in Predicting in-Hospital Mortality of Infected Children at China's PICU. Shock 2019; 52(3): 347-52.
120. Xu X, Nie S, Zhang A, et al. Acute Kidney Injury among Hospitalized Children in China. Clin J Am Soc Nephrol 2018; 13(12): 1791-800.

121. Yurtseven A, Turan C, Kilinc MA, Ulas Saz E. Frequency and outcomes of endotracheal intubation in the pediatric emergency department. Turk J Pediatr 2017; 59(5): 524-30.
122. Zaidi AA, Dixon J, Lupez K, et al. The burden of trauma at a district hospital in the Western Cape Province of South Africa. African Journal of Emergency Medicine 2019; 9: S14-S20.
123. Zhang QL, Wan CM, MacDonald NE. Vaccine preventable infections and multiple organ dysfunction syndrome in critically ill children in China. Pediatr Infect Dis J 2009; 28(3): 182-5.
124. Zhang Q, Guo Zx, MacDonald NE. Vaccine preventable community-acquired pneumonia in hospitalized children in Northwest China. Pediatr Infect Dis J 2011; 30(1): 7-10.
125. Zhao Q, Li S, Abramson MJ, et al. The association between heatwaves and risk of hospitalization in Brazil: A nationwide time series study between 2000 and 2015. PLoS Medicine 2019; 16(2): 1-16.

Not original research (N=118)

1. Abebe MW, Tefera T, Mengesha MG, Mengesha MW, Teshome S. Case series of hyena bite injuries and their surgical management in a resource-limited setup: 1-year experience. J 2020; 2020(10): rjaa446.
2. Accorsi S, Fabiani M, Nattabi B, et al. The disease profile of poverty: morbidity and mortality in northern Uganda in the context of war, population displacement and HIV/AIDS. Trans R Soc Trop Med Hyg 2005; 99(3): 226-33.
3. Afifi S, Wasfy MO, Azab MA, et al. Laboratory-based surveillance of patients with bacterial meningitis in Egypt (1998-2004). Eur J Clin Microbiol Infect Dis 2007; 26(5): 331-40.
4. Ahmed BW, Hanoudi BM, Ibrahim BA. Risk factors in children with febrile seizures and their iron status. JPMA J Pak Med Assoc 2019; 69(Suppl 3)(8): S22-S5.
5. Al-Mendalawi MD. Mortality patterns among critically ill children in a pediatric intensive care unit of a developing country. Indian J 2015; 19(5): 293-4.
6. Al-Tehewy M, Shehad E, Al Gaafary M, Al-Houssiny M, Nabih D, Salem B. Appropriateness of hospital admissions in general hospitals in Egypt. East Mediterr Health J 2009; 15(5): 1126-34.
7. Alazab R. Children Below 5 years of Employed Mothers Are Less Exposed to Acute Poisoning in Alexandria, Egypt. Occupational & Environmental Medicine 2014; 71: A63-A.
8. Alfaro Rojas AK, Atria Machuca RP. Factores ambientales y su incidencia en la experiencia emocional del niño hospitalizado. Pediatrí-a (Santiago de Chile) 2009; 6(1).
9. Allison WE, Kiromat M, Vince J, et al. Development of a clinical algorithm to prioritise HIV testing of hospitalised paediatric patients in a low resource moderate prevalence setting. Archives of Disease in Childhood 2011; 96(1): 67-72.
10. Alvarez P AM, Galvez R SM. Accidente cerebrovascular: revisión de la literatura: etiologí-a, diagnóstico, tratamiento general y anílisis pediítrico. Rev colomb enferm 2011; 6(6): 102-20.
11. Anderson M, Luangxay K, Sisouk K, et al. Epidemiology of bacteremia in young hospitalized infants in Vientiane, Laos, 2000-2011. J Trop Pediatr 2014; 60(1): 10-6.
12. Andrade AL, Afonso ET, Minamisava R, et al. Direct and indirect impact of 10-valent pneumococcal conjugate vaccine introduction on pneumonia hospitalizations and economic burden in all age-groups in Brazil: A time-series analysis. PLoS ONE 2017; 12(9).
13. Anh DD, Riewpaiboon A, Le Tho H, et al. Treatment costs of pneumonia, meningitis, sepsis, and other diseases among hospitalized children in Viet Nam. Journal of Health, Population & Nutrition 2010; 28(5): 436-42.
14. Arslankoylu AE, Bayrakci B, Oymak Y. Admission time and mortality rates. Indian J Pediatr 2008; 75(7): 691-4.
15. Ascarza Atau LP. Factores de riesgo asociados a mortalidad en niños con traumatismo encefalocraneano en la Unidad de Cuidados Intensivos del Hospital de Emergencias Pediátricas, periodo 2006-2010. 2013: 63-.
16. Asl HM, Nateghian A. Epidemiology of nosocomial infections in a pediatric intensive care unit (PICU). Iranian Journal of Clinical Infectious Diseases 2009; 4(2): 83-6.
17. Awotiwon OF, Pillay- van Wyk V, Dhansay A, Day C, Bradshaw D. Diarrhoea in children under five years of age in South Africa (1997-2014). Tropical Medicine & International Health 2016; 21(9): 1060-70.
18. Ayad A, Malih M, Agadr A. SFP PC-27 - Epidemiological profile of pediatric hospitalizations at the Moroccan military field hospital in the ZAATARI refugee camp (Jordan 2013). Archives de Pediatrie 2014; 21(5): 917.
19. Ayieko P, Ntoburi S, Wagai J, et al. A multifaceted intervention to implement guidelines and improve admission paediatric care in Kenyan district hospitals: a cluster randomised trial. PLoS Med 2011; 8(4): e1001018.
20. Bahl R, Ray P, Subodh S, et al. Incidence of severe rotavirus diarrhea in New Delhi, India, and G and P types of the infecting rotavirus strains. Journal of Infectious Diseases 2005; 192: S114-9.
21. Barasa EW, Ayieko P, Cleary S, English M. Out-of-pocket costs for paediatric admissions in district hospitals in Kenya. Trop Med Int Health 2012; 17(8): 958-61.
22. Becerra MR, Tantalean JA, Suarez VJ, Alvarado MC, Candela JL, Urcia FC. Epidemiologic surveillance of nosocomial infections in a Pediatric Intensive Care Unit of a developing country. BMC Pediatr 2010; 10: 66.
23. Behera CK, Jain MK, Mishra R, Jena PK, Dash SK, Sahoo RK. Clinico-radiological Profile of Posterior Reversible Encephalopathy Syndrome and Its Associated Risk Factors in PICU: A Single-center Experience from a Tertiary Care Hospital in Bhubaneswar, Odisha. Indian J 2020; 24(12): 1223-9.

24. Berkley JA, Lowe BS, Mwangi I, et al. Bacteremia among children admitted to a rural hospital in Kenya. N Engl J Med 2005; 352(1): 39-47.
25. Berkley JA, Maitland K, Mwangi I, et al. Use of clinical syndromes to target antibiotic prescribing in seriously ill children in malaria endemic area: observational study. Bmj 2005; 330(7498): 995.
26. Bharti B. Pediatric Hospital Readmissions: An Emerging Metric of Healthcare Quality. Indian Journal of Pediatrics 2019; 86(3): 220-1.
27. Bhatta NK, Shrestha P, Budathoki S, et al. Profile of renal diseases in Nepalese children. Kathmandu University Medical Journal 2008; 6(22): 191-4.
28. Bills C, Newberry JA, Matheson L, Rao R, Mahadevan SV, Strehlow MC. 317 Acute Respiratory Illness among a Prospective Cohort of Pediatric Patients Using Emergency Medical Services in India: Demographic, Environmental, and Out-of-Hospital Clinical Predictors of Mortality in a High-Risk Population. Annals of Emergency Medicine 2018; 72: S124-S5.
29. Bonkoungou IJO, Aliabadi N, Leshem E, et al. Impact and effectiveness of pentavalent rotavirus vaccine in children <5years of age in Burkina Faso. Vaccine 2018; 36(47): 7170-8.
30. Branco RG, Xavier L, Garcia PC, et al. Prospective operationalization and feasibility of a glycemic control protocol in critically ill children. Pediatr Crit Care Med 2011; 12(3): 265-70.
31. Broughton EI, Gomez I, Nuñez O, Wong Y. Cost-effectiveness of improving pediatric hospital care in Nicaragua. Rev panam salud pública 2011; 30(5): 453-60.
32. Cavalcante SS, Mota E, Silva LR, Teixeira LF, Cavalcante LB. Risk factors for developing nosocomial infections among pediatric patients. Pediatr Infect Dis J 2006; 25(5): 438-45.
33. Cavalini LT, Ponce de Leon ACM. Correction approach for underreporting of deaths and hospital admissions due to ill-defined causes. Revista de Saude Publica 2007; 41(1): 85-93.
34. Collaborators, G. B. D. Global, regional, and national incidence, prevalence, and years lived with disability for 354 diseases and injuries for 195 countries and territories, 1990-2017: a systematic analysis for the Global Burden of Disease Study 2017. Lancet 2018; 392 North American Edition(10159): 1789-858.
35. Corten L, As AB, Rahim S, et al. Physiotherapy in children hospitalized with traumatic brain injury in a South African tertiary paediatric hospital. Physiotherapy Research International 2020; 25(4): 1-9.
36. de Sandes-Freitas TV, Canito Brasil IR, Oliveira Sales M, et al. Lessons from SARS-CoV-2 screening in a Brazilian organ transplant unit. Transpl Infect Dis 2020; 22(6): e13376.
37. De Vos P, Barroso I, Rodriguez A, Bonet M, Van der Stuyft P. The functioning of the Cuban home hospitalization programme: a descriptive analysis. BMC Health Serv Res 2007; 7: 76.
38. Dewan MC, Mummareddy N, Wellons JC, 3rd, Bonfield CM. Epidemiology of Global Pediatric Traumatic Brain Injury: Qualitative Review. World Neurosurg 2016; 91: 497-509.e1.
39. Dulal S, Ale JB, Sapkota YD. Profile of pediatric ocular trauma in mid western hilly region of Nepal. Nepal J Ophthalmol 2012; 4(1): 134-7.
40. Fabiansen C, Cichon B, Yameogo CW, et al. Association between admission criteria and body composition among young children with moderate acute malnutrition, a cross-sectional study from Burkina Faso. Sci 2020; 10(1): 13266.
41. Fell DB, Johnson J, Mor Z, et al. Incidence of laboratory-confirmed influenza disease among infants under 6 months of age: a systematic review. BMJ Open 2017; 7(9): e016526.
42. Ferrer Machín M, Jiménez Bodib JR, Ferníndez Romo F. Ventilación mecánica en UCIP: año 2006. Mediciego 2007; 13(supl.2).
43. Fusheini A, Gyawu SK. Prevalence of typhoid and paratyphoid fever in the hohoe municipality of the volta region, ghana: A five-year retrospective trend analysis. Annals of Global Health 2020; 86(1): 1-10.
44. Garcia PCR, Piva JP. Pediatric Index of Mortality 2 (PIM2)--a prognostic tool for developing countries: easy, efficient, and free! Pediatric Critical Care Medicine 2007; 8(1): 77-8.
45. Gonzílez AL, Martí-nez RA, Villar Lín. Evolución clínica de pacientes hospitalizados por dengue en una institución de salud de Bucaramanga, Colombia. Biomédica (Bogotí) 2008; 28(4): 531-43.
46. Guiraud I, Post A, Diallo SN, et al. Population-based incidence, seasonality and serotype distribution of invasive salmonellosis among children in Nanoro, rural Burkina Faso. PLoS ONE 2017; 12(7).
47. Gyenwali D, Vaidya A, Tiwari S, Khatiwada P, Lamsal DR, Giri S. Pesticide poisoning in Chitwan, Nepal: a descriptive epidemiological study. BMC Public Health 2017; 17(1): 619.
48. Halstead SB, Suaya JA, Shepard DS, Halstead SB, Suaya JA, Shepard DS. The burden of dengue infection. Lancet 2007; 369 North American Edition(9571): 1410-1.
49. Haque A, Ahmed SA, Rafique Z, Abbas Q, Jurair H, Ali SA. Device-associated infections in a paediatric intensive care unit in Pakistan. Journal of Hospital Infection 2017; 95(1): 98-100.
50. Harris S, Jack S. Home-based treatment of acute malnutrition in Cambodian urban poor communities. Food Nutr Bull 2011; 32(4): 333-9.
51. Hassam I, Kisenge R, Aboud S, Manji K. Association of vitamin D and diarrhoea in children aged less than five years at Muhimbili national hospital, Dar es Salaam: an unmatched case control study. BMC Pediatr 2019; 19(1): 237.

52. Hassan RH, Eldegla H, Elmorsy F, Eldars WM. Clinical and microbiological characteristics of healthcare-associated infections in a tertiary care pediatric hospital. Egyptian Pediatric Association Gazette 2017; 65(4): 127-31.
53. Hayakawa LY, Marcon SS, Waidman MAP. Using the group as a support strategy for mothers of children admitted to Pediatric Intensive Care unit: an experience report. Online Brazilian Journal of Nursing 2009; 8(3): 19p-p.
54. Hegazi M, Abdelkader A, Zaki M, El-Deek B. Characteristics and risk factors of candidemia in pediatric intensive care unit of a tertiary care children's hospital in Egypt. J 2014; 8(5): 624-34.
55. Hertz JT, Munishi OM, Sharp JP, Reddy EA, Crump JA. Comparing actual and perceived causes of fever among community members in a low malaria transmission setting in northern Tanzania. Trop Med Int Health 2013; 18(11): 1406-15.
56. Homaira N, Luby SP, Alamgir AS, et al. Influenza-associated mortality in 2009 in four sentinel sites in Bangladesh. Bull World Health Organ 2012; 90(4): 272-8.
57. Ibinda F, Zarnack HC, Newton CR. Sodium Disturbances in Children Admitted to a Kenyan Hospital: Magnitude, Outcome and Associated Factors. PLoS ONE 2016; 11(9): e0161320.
58. Itaka MB, Omole OB. Prevalence and factors associated with malnutrition among under 5-year-old children hospitalised in three public hospitals in South Africa. Afr 2020; 12(1): e1-e7.
59. Iyer P, Iyer PU. Management Issues in Intensive Care Units for Infants and Children with Heart Disease. Indian Journal of Pediatrics 2015; 82(12): 1164-71.
60. Jofré ML, Vega-Briceño L. Impacto de la infección por virus influenza en niños: costos, morbilidad y mortalidad asociada. Neumol pediítr 2009; 4(1): 10-3.
61. Kanyina EW. Characterization of visceral leishmaniasis outbreak, Marsabit County, Kenya, 2014. BMC Public Health 2020; 20(1): 446.
62. Khanal A, Sharma A, Basnet S. Current State of Pediatric Intensive Care and High Dependency Care in Nepal. Pediatr Crit Care Med 2016; 17(11): 1032-40.
63. Kissoon N, Carcillo JA, Espinosa V, et al. World federation of pediatric intensive care and critical care societies: Global sepsis initiative. Pediatric Critical Care Medicine 2011; 12(5): 494-503.
64. Lum LCS. Reducing PICU Mortality in Resource-Limited Settings. Pediatric Critical Care Medicine 2020: 1007-8.
65. Luong LMT, Phung D, Dang TN, Sly PD, Morawska L, Thai PK. Seasonal association between ambient ozone and hospital admission for respiratory diseases in Hanoi, Vietnam. PLoS ONE 2018; 13(9): e0203751.
66. Luong LMT, Phung D, Sly PD, Dang TN, Morawska L, Thai PK. Effects of temperature on hospitalisation among pre-school children in Hanoi, Vietnam. Environ Sci Pollut Res Int 2019; 26(3): 2603-12.
67. Luong LM, Phung D, Sly PD, Morawska L, Thai PK. The association between particulate air pollution and respiratory admissions among young children in Hanoi, Vietnam. Sci Total Environ 2017; 578: 249-55.
68. Maitland K, Kiguli S, Olupot-Olupot P, et al. Immediate transfusion in African children with uncomplicated severe anemia. New England Journal of Medicine 2019; 381(5): 407-19.
69. Maitland K, Kiguli S, Opoka RO, et al. Mortality after fluid bolus in African children with severe infection. New England Journal of Medicine 2011; 364(26): 2483-95.
70. Martins CL, Benn CS, Andersen A, et al. A randomized trial of a standard dose of Edmonston-Zagreb measles vaccine given at 4.5 months of age: effect on total hospital admissions. Journal of Infectious Diseases 2014; 209(11): 1731-8.
71. Masukawa Mde L, Moriwaki AM, Uchimura NS, Souza EM, Uchimura TT. Intervention analysis of introduction of rotavirus vaccine on hospital admissions rates due to acute diarrhea. 2014; 1(10): 2101-11.
72. McAllister DA, Liu L, Shi T, et al. Global, regional, and national estimates of pneumonia morbidity and mortality in children younger than 5 years between 2000 and 2015: a systematic analysis. Lancet Glob Health 2019; 7(1): e47-e57.
73. McAuley CF, Webb C, Makani J, et al. High mortality from Plasmodium falciparum malaria in children living with sickle cell anemia on the coast of Kenya. Blood 2010; 116(10): 1663-8.
74. Mejí-a-Santos HM, Couto P, Palekar R, et al. Hospitalizaciones y mortalidad asociada a influenza, Honduras. 2011-2015. Rev fac cienc méd (Impr) 2019; 16(2,n.esp): 11-22.
75. Menezes RAM, Pavanitto DR, Nascimento LFC. Spatial distribution of hospitalization rates of children due to pneumonia in the Unified Health System, in the municipalities of the state of Sao Paulo. Rev 2019; 22: e190053.
76. Morrow BM, Feldman C, Green RJ. Acute viral bronchiolitis in South Africa: Intensive care management for severe disease. South African Medical Journal 2016; 106(5): 446-8.
77. Mosser P, Schmutzhard E, Winkler AS. The pattern of epileptic seizures in rural Tanzania. J Neurol Sci 2007; 258(1-2): 33-8.
78. Motta FA, Dalla-Costa LM, Muro MD, et al. Risk factors for candidemia mortality in hospitalized children. J Pediatr (Rio J) 2017; 93(2): 165-71.
79. Mulenga V, Ford D, Walker AS, et al. Effect of cotrimoxazole on causes of death, hospital admissions and antibiotic use in HIV-infected children. AIDS 2007; 21(1): 77-84.
80. Muloiwa R, Kagina BM, Engel ME, Hussey GD. The burden of laboratory-confirmed pertussis in low- and middle-income countries since the inception of the Expanded Programme on Immunisation (EPI) in 1974: a systematic review and meta-analysis. BMC Medicine 2020; 18(1): N.PAG-N.PAG.

81. Muzigaba M, Kigozi G, Puoane T. Short-term and sustained effects of a health system strengthening intervention to improve mortality trends for paediatric severe malnutrition in rural South African hospitals. SAJCH South African Journal of Child Health 2017; 11(1): 38-45.
82. Nair H, Simoes EA, Rudan I, et al. Global and regional burden of hospital admissions for severe acute lower respiratory infections in young children in 2010: a systematic analysis. Lancet 2013; 381(9875): 1380-90.
83. Nathoo N, Nadvi SS, Narotam PK, van Dellen JR. Brain abscess: management and outcome analysis of a computed tomography era experience with 973 patients. World Neurosurg 2011; 75(5-6): 716-26; discussion 612.
84. Nielsen KR, Arones Rojas R, Tantalean da Fieno J, Huicho L, Roberts JS, Zunt J. Emergency department risk factors for serious clinical deterioration in a paediatric hospital in Peru. J Paediatr Child Health 2018; 54(8): 866-71.
85. Njeru EK, Mutiso VM, Saidi H, Mak'Anyengo K, Gakuru JN. Firearm injuries at selected hospitals in Kenya. Annals of African Surgery 2008; 3: 3-9.
86. Nteziyaremye J, Paasi G, Burgoine K, Sadiq Balyejjusa J, Tegu C, Olupot-Olupot P. Perspectives on aetiology, pathophysiology and management of shock in African children. Afr J Emerg Med 2017; 7(Suppl): S20-S6.
87. Ogero M, Sarguta RJ, Malla L, et al. Prognostic models for predicting in-hospital paediatric mortality in resource-limited countries: a systematic review. BMJ Open 2020; 10(10): e035045.
88. Olola CH, Missinou MA, Issifou S, et al. Medical informatics in medical research - the Severe Malaria in African Children (SMAC) Network's experience. Methods Inf Med 2006; 45(5): 483-91.
89. Palekar RS, Rolfes MA, Arriola CS, et al. Burden of influenza-associated respiratory hospitalizations in the Americas, 2010-2015. PLoS ONE 2019; 14(9).
90. Pedraza DF, Araujo EM. Hospitalizations of Brazilian children under fiver years old: a systematic review. Epidemiol 2017; 26(1): 169-82.
91. Procter C, Morrow B, Pienaar G, Shelton M, Argent A. Outcomes following admission to paediatric intensive care: A systematic review. J Paediatr Child Health 2021; 12: 12.
92. Puumalainen T, Quiambao B, Abucejo-Ladesma E, et al. Clinical case review: a method to improve identification of true clinical and radiographic pneumonia in children meeting the World Health Organization definition for pneumonia. BMC Infectious Diseases 2008; 8(1): 95-.
93. Raihana S, Dunsmuir D, Huda T, et al. Erratum: Development and internal validation of a predictive model including pulse oximetry for hospitalization of under-five children in Bangladesh (PLoS ONE (2016) 11:1). PLoS ONE 2016; 11(1).
94. Ramos Miraval PD. Factores de riesgo asociados en menores de 5 años hospitalizados con infección respiratoria aguda baja por virus respiratorios en el Hospital de Emergencias Pediítricas 2011-2012. 2013: 84-.
95. Ranjit S, Kissoon N, Jayakumar I. Aggressive management of dengue shock syndrome may decrease mortality rate: a suggested protocol. Pediatr Crit Care Med 2005; 6(4): 412-9.
96. Remtullah AZ, Sirili N, Anaeli A, Massawe A, Manji K, Sunguya BF. Quality of Healthcare in Acute Pediatric Care Unit in a Tertiary Hospital in Tanzania: A Case of Muhimbili National Hospital. Front 2020; 8: 496.
97. Roine I, Pelkonen T, Bernardino L, et al. Factors affecting time to death from start of treatment among children succumbing to bacterial meningitis. Pediatr Infect Dis J 2014; 33(8): 789-92.
98. Rojjanadumrongkul K, Kumthip K, Khamrin P, Ukarapol N, Ushijima H, Maneekarn N. Enterovirus infections in pediatric patients hospitalized with acute gastroenteritis in Chiang Mai, Thailand, 2015-2018. Peerj 2020; 8: e9645.
99. Roy SK, Raqib R, Khatun W, et al. Zinc supplementation in the management of shigellosis in malnourished children in Bangladesh. Eur J Clin Nutr 2008; 62(7): 849-55.
100. Sakyi SA, Enimil A, Adu DK, et al. Individual and combined bioscore model of presepsin, procalcitonin, and high sensitive C - reactive protein as biomarkers for early diagnosis of paediatric sepsis. Heliyon 2020; 6(9): e04841.
101. Sankar J, Singh A, Sankar MJ, Joghee S, Dewangan S, Dubey N. Pediatric Index of Mortality and PIM2 scores have good calibration in a large cohort of children from a developing country. Biomed Res Int 2014; 2014: 907871.
102. Senarathna L, Buckley NA, Jayamanna SF, Kelly PJ, Dibley MJ, Dawson AH. Validity of referral hospitals for the toxicovigilance of acute poisoning in Sri Lanka. Bull World Health Organ 2012; 90(6): 436-43A.
103. Seni J, Mwakyoma AA, Mashuda F, et al. Deciphering risk factors for blood stream infections, bacteria species and antimicrobial resistance profiles among children under five years of age in North-Western Tanzania: a multicentre study in a cascade of referral health care system. BMC Pediatr 2019; 19(1): 32.
104. Shiva F, Ghotbi F, Padyab M. Infant feeding and hospitalization during the first six months of life. JPMA J Pak Med Assoc 2007; 57(12): 599-603.
105. Sinimole KR. Emerging Patterns of Morbidity and Hospitalization- A Comparison of Kerala and Bihar. Illness, Crisis & Loss 2020; 28(4): 321-46.
106. Southeast Asia Infectious Disease Clinical Research. Causes and outcomes of sepsis in southeast Asia: a multinational multicentre cross-sectional study. Lancet Glob Health 2017; 5(2): e157-e67.
107. Suarez V, Michel F, Toscano CM, et al. Impact of pneumococcal conjugate vaccine in children morbidity and mortality in Peru: Time series analyses. Vaccine 2016; 34(39): 4738-43.
108. Sudarmono P, Aman AT, Arif M, et al. Causes and outcomes of sepsis in southeast Asia: a multinational multicentre cross-sectional study. The Lancet Global Health 2017; 5(2): e157-e67.

109. Suzuki M, Yoshida LM, Ariyoshi K, Suzuki M, Yoshida L-M, Ariyoshi K. Severe pneumonia among Kenyan infants and children. Chicago, Illinois: American Medical Association; 2010. p. 964-5.
110. Tadepalli D, Srinadh A, Balaji K, Thomas D. Acute diarrhea demanding hospitalization in rural India. Rural & Remote Health 2014; 14(3): 1-3.
111. Teixeira MG. Few characteristics of dengue's fever epidemiology in Brazil. Rev Inst Med Trop São Paulo 2012; 54(supl.18): 1-4.
112. Tette EMA, Nyarko MY, Nartey ET, et al. Under-five mortality pattern and associated risk factors: a case-control study at the Princess Marie Louise Children's Hospital in Accra, Ghana. BMC Pediatr 2016; 16(1): 148.
113. Tovar Ibarra I. Caracterí-sticas clí-nico epidemiológicas y pertinencia de la referencia de los pacientes derivados para hospitalización al Servicio de Emergencia del Instituto Nacional de Salud del Niño durante el año 2013. 2014: 42-.
114. Winkler AS, Salmhofer G, Helbok R, Kalokola F, Matuja W, Schmutzhard E. In-hospital risk estimation in children with malaria--early predictors of morbidity and mortality. J Trop Pediatr 2008; 54(3): 184-91.
115. Winkler AS, Tluway A, Schmutzhard E. Febrile seizures in rural Tanzania: hospital-based incidence and clinical characteristics. J Trop Pediatr 2013; 59(4): 298-304.
116. Yadav SP, Shah GS, Mishra OP, Baral N. Pattern of renal diseases in children: A developing country experience. Saudi J Kidney Dis Transpl 2016; 27(2): 371-6.
117. Yameogo TM, Kyelem CG, Bamba S, et al. Management of suspected cases of malaria before admission to a district hospital in Burkina Faso. Med 2014; 24(3): 301-6.
118. World Health Organization. Estimating meningitis hospitalization rates for sentinel hospitals conducting surveillance of invasive bacterial vaccine preventable diseases. Weekly epidemiological record / Health Section of the Secretariat of the League of Nations 2013; 88(43): 471.

Overlapping populations (N=4)

1. de Barros Hatisuka MF, Oliveira de Arruda G, Molena Fernandes CA, Silva Marcon S. Tendency analysis of admission rates for bacterial pneumonia in children and adolescents. 294-300.
2. Ferrer AP, Sucupira AC, Grisi SJ. Causes of hospitalization among children ages zero to nine years old in the city of São Paulo, Brazil. Clinics (Sao Paulo) 2010; 65(1): 35-44.
3. Maior MDCL, Osorio-de-Castro CGS, Andrade CLT. Hospitalizations due to drug poisoning in under-five-year-old children in Brazil, 2003-2012. Epidemiol Serv Saude 2017; 26(4): 771-82.
4. Sartori AM, Novaes CG, de Soárez PC, Toscano CM, Novaes HM. Estimating health service utilization for treatment of pneumococcal disease: the case of Brazil. Vaccine 2013; 31 Suppl 3: C63-71.
